# Supplementary material for: Vitruvian binders in Venice: First evidence of Phlegraean pozzolans in an underwater Roman construction in the Venice Lagoon
Source: PLoS One. 2024 Nov 22;19(11):e0313917. doi: 10.1371/journal.pone.0313917 (PMC11584134; doi:10.1371/journal.pone.0313917)
Supplement: S1 File — (DOCX) [file pone.0313917.s001.docx]

**S1 Analytical methods.** Instrumental equipment and standards

*Polarized Light Optical Microscopy (PLM)*

All mortar samples were analyzed by means of Polarized Light Microscopy (PLM) on 30 μm thin sections under a Leica DM790 P equipped with an integrated digital camera FLEXACAM C1.

*Quantitative Phase Analysis - X-Ray Powder Diffraction (QPA-XRPD)*

QPA-XRPD analyses were performed on the bulk composition of the four mortar samples from TSF structure, mechanically grinded on an agate mortar, and on the binder-concentrated fractions of the TSF_T9 sample. The binder fraction of this sample was separated in water solution following the separation procedure according to [60].

XRPD profiles were collected using a Bragg–Brentano θ-θ diffractometer (PANalytical X’Pert PRO, Cu Kα radiation, 40 kV and 40 mA) equipped with a real-time multiple strip (RTMS) detector (PIXcel by Panalytical). Data acquisition was performed by operating a continuous scan in the 3–85 [◦2θ] range, with a virtual step scan of 0.02 [◦2θ]. Diffraction patterns were interpreted with X’Pert HighScore Plus 3.0 software by PANalytical, qualitatively reconstructing mineral profiles of the compounds by comparison with PDF databases from the International Centre for Diffraction Data (ICDD).

Qantitative phase analysis (QPA) was performed using the Rietveld method [58]. Refinements were carried out with TOPAS software (version 4.1) by Bruker AXS. The quantification of both crystalline and amorphous content was obtained through the addition of 20 wt% of zincite to the powders as internal standard. The observed Bragg peaks in the powder patterns have been modelled through a pseudo-Voigt function, fitting the background with a 12 coefficients Chebyshev polynomial. For each mineral phase, lattice parameters, Lorentzian crystal sizes and scale factors have been refined. Although samples were prepared with the backloading technique to minimize preferred orientation of crystallites a priori, any residual preferred orientation effect was modelled during the refinement with the March Dollase algorithm. The starting structural models for the refinements were taken from the International Crystal Structure Database (ICSD).

*Scanning Electron Microscopy (SEM) coupled with Energy-Dispersive X-Ray Spectroscopy (EDS)*

SEM-EDS analyses were performed to investigate locally the chemical composition of the binder and aggregates and the reaction zones in the samples. The analytical instrument used for this analysis was a FEI Quanta 200 microscope, equipped with an Energy Dispersive X-ray detector (EDX) EDAX Element- C2B. Standarless semiquantitative analysis by Team EDAX software (based on ZAF correction and factory standardization data implemented in the software) was previously tested on two NIST certificated reference materials: SRM 2066 K411 and SRM 620. These materials are glasses with a chemical composition compatible with the volcanic tephra analyzed in this study. In particular, SRM 2066 K411 is a specific reference material for SEM-EDX analysis. NIST standards were adopted to verify the EDS accuracy.

| **Standard NIST-620 - Soda-Lime Flat Glass (NIST certificated data)** | | | | |  | **Results of EDS analysis of standard NIST-620 (mean of 5 spot analyses)** | | | |
| --- | --- | --- | --- | --- | --- | --- | --- | --- | --- |
| **Element** | **wt%** | **Uncertainty** |  |  |  | **Element** | **mean (wt%)** | **SD** |  |
| SiO_2_ | 72.08 | 0.08 |  |  |  | SiO_2_ | 71.6 | 0.11 |  |
| Na_2_O | 14.39 | 0.06 |  |  |  | Na_2_O | 14.7 | 0.14 |  |
| CaO | 7.11 | 0.05 |  |  |  | CaO | 8.1 | 0.20 |  |
| MgO | 3.69 | 0.05 |  |  |  | MgO | 3.4 | 0.19 |  |
| Al_2_O_3_ | 1.8 | 0.03 |  |  |  | Al_2_O_3_ | 1.6 | 0.08 |  |
| K_2_O | 0.41 | 0.03 |  |  |  | K_2_O | 0.5 | 0.01 |  |
| SO_3_ | 0.28 | 0.02 |  |  |  | SO_3_ | 0.2 | 0.05 |  |
| As_2_O_3_ | 0.056 | 0.003 |  |  |  |  |  |  |  |
| Fe_2_O_3_ | 0.043 | 0.004 |  |  |  |  |  |  |  |
| TiO_2_ | 0.018 | 0.002 |  |  |  |  |  |  |  |
|  |  |  |  |  |  |  |  |  |  |
|  |  |  |  |  |  |  |  |  |  |
| **Standard NIST-2066-k411 - Glass microspheres (NIST certificated data)** | | | | | | **Results of EDS analysis of standard NIST-2066-k411 (mean of 5 spot analyses)** | | | |
| **Element** | **wt%** | **Uncertainty** |  |  |  | **Element** | **mean (wt%)** | **SD** |  |
| Si | 25.6 | 1.7 |  |  |  | Si | 24.7 | 0.15 |  |
| Ca | 11.2 | 2.3 |  |  |  | Ca | 11.7 | 0.09 |  |
| Mg | 9.2 | 1.4 |  |  |  | Mg | 9.7 | 0.10 |  |
| Fe | 11.2 | 2.3 |  |  |  | Fe | 10.3 | 0.13 |  |
| Oxygen | 42.9 | 1.2 |  |  |  | Oxygen | 43.6 | 0.07 |  |

*Laser Ablation-Inductively Coupled Plasma-Mass Spectrometry (LA-ICP-MS)*

Trace elements were analysed on selected pumice clasts from ~ 1 mm thick polished sections by Laser-Ablation Inductively-Coupled-Plasma Mass-Spectrometry (LA-ICP-MS) at the Laboratory of the Centro Inter-dipartimentale Grandi Strumenti (CIGS) of the University of Modena and Reggio Emilia. Analyses were carried out using a Thermo Fisher ICP-MS iCAP-TQ coupled with a laser ablation NewWave UP 213. Laser spot size was calibrated at 55 μm and laser beam fluency at 10 microJoule for cm^2^. Instrument was calibrated using NIST-612 reference material checking ^139^La, ^238^U and the ^238^U/^232^Th ratio. The oxide production within the plasma was monitored through the ^232^Th^16^O/^232^Th ratio, constantly kept below 0.01%. Spot analyses consist of 30 s of background acquisition followed by 60 s of signal acquisition during ablation and 40 s of washout. During the analytical session, reference materials NIST-610, NIST-612, NIST-614 and ML3-B were analysed (see table in the bottom of the paragraph). NIST-612 was used as known standard for the data reduction while NIST-610, NIST-614 and ML3B were used as unknown to check accuracy and precision. Reference material ML3B was used as unknown in a primary step of correction and then it was used to recalculate correction factors for the analytical session to perform a 2-standard correction during the data reduction. The data reduction was performed with an in-house Excel spreadsheet. ^44^Ca from SEM analyses was used as internal standard for the pumice clasts. Standard values here reported in the table below from the analytical session are in ppm.

| **Standard** | ML3B | ML3B | ML3B | ML3B | ML3B | ML3B | ML3B | ML3B | ML3B | ML3B | ML3B | ML3B | ML3B | ML3B | ML3B | ML3B | ML3B | ML3B | ML3B | ML3B |
| --- | --- | --- | --- | --- | --- | --- | --- | --- | --- | --- | --- | --- | --- | --- | --- | --- | --- | --- | --- | --- |
| **Li** | 4.4 | 4.8 | 4.2 | 4.6 | 4.4 | 4.6 | 4.6 | 4.5 | 4.5 | 4.4 | 4.5 | 4.4 | 4.4 | 4.5 | 4.6 | 4.7 | 4.4 | 4.5 | 4.6 | 4.5 |
| **Be** | 0.514 | 0.675 | 0.674 | 0.671 | 0.408 | 0.480 | 0.652 | 0.563 | 0.608 | 0.675 | 0.507 | 0.653 | 0.637 | 0.570 | 0.566 | 0.923 | 0.669 | 0.657 | 0.733 | 0.566 |
| **B** | 3.00 | 2.28 | 2.16 | 2.39 | 6.2 |  | 2.60 | 2.69 | 2.22 | 2.36 | 2.48 | 2.70 | 2.02 | 2.78 | 2.67 | 2.59 | 2.41 | 2.49 | 2.66 | 2.50 |
| **Sc** | 33 | 31 | 33 | 31 | 31 | 33 | 32 | 32 | 31 | 32 | 31 | 32 | 31 | 32 | 31 | 32 | 31 | 31 | 32 | 31 |
| **V** | 268 | 269 | 271 | 267 | 259 | 271 | 269 | 268 | 268 | 269 | 267 | 267 | 267 | 269 | 264 | 275 | 267 | 268 | 269 | 266 |
| **Cr** | 178 | 175 | 180 | 177 | 172 | 182 | 179 | 177 | 175 | 178 | 177 | 177 | 177 | 178 | 173 | 177 | 177 | 178 | 178 | 176 |
| **Co** | 42 | 40 | 42 | 41 | 39 | 46 | 42 | 41 | 41 | 41 | 41 | 41 | 41 | 42 | 40 | 40 | 40 | 41 | 42 | 41 |
| **Ni** | 110 | 105 | 109 | 106 | 104 | 109 | 106 | 108 | 107 | 108 | 107 | 108 | 105 | 108 | 105 | 110 | 105 | 107 | 108 | 105 |
| **Cu** | 115 | 108 | 115 | 111 | 109 | 114 | 112 | 113 | 111 | 112 | 112 | 114 | 110 | 114 | 109 | 112 | 112 | 112 | 113 | 111 |
| **Zn** | 107 | 106 | 104 | 109 | 119 | 107 | 108 | 109 | 109 | 110 | 107 | 106 | 112 | 113 | 106 | 104 | 102 | 106 | 100 | 114 |
| **Rb** | 6.0 | 5.7 | 5.8 | 5.8 | 5.8 | 6.1 | 5.8 | 5.8 | 5.8 | 5.9 | 5.7 | 5.8 | 5.7 | 5.9 | 5.7 | 5.5 | 5.8 | 5.8 | 5.9 | 5.8 |
| **Sr** | 318 | 304 | 312 | 312 | 297 | 318 | 313 | 313 | 311 | 315 | 307 | 316 | 310 | 316 | 308 | 316 | 318 | 311 | 314 | 312 |
| **Y** | 25 | 23 | 24 | 24 | 23 | 25 | 24 | 24 | 24 | 24 | 24 | 24 | 23 | 24 | 24 | 24 | 25 | 24 | 24 | 24 |
| **Zr** | 124 | 119 | 123 | 120 | 115 | 125 | 123 | 123 | 121 | 124 | 120 | 124 | 121 | 125 | 121 | 126 | 123 | 122 | 124 | 120 |
| **Nb** | 8.5 | 8.1 | 8.3 | 8.2 | 8.5 | 8.5 | 8.3 | 8.3 | 8.3 | 8.4 | 8.2 | 8.4 | 8.2 | 8.3 | 8.4 | 8.0 | 8.2 | 8.3 | 8.3 | 8.3 |
| **Mo** | 17 | 16 | 17 | 16 | 15 | 17 | 16 | 17 | 17 | 17 | 16 | 17 | 17 | 17 | 16 | 17 | 17 | 17 | 17 | 16 |
| **Cs** | 0.155 | 0.133 | 0.145 | 0.134 | 0.148 | 0.088 | 0.131 | 0.149 | 0.139 | 0.137 | 0.138 | 0.145 | 0.127 | 0.138 | 0.143 | 0.179 | 0.145 | 0.141 | 0.145 | 0.140 |
| **Ba** | 82 | 77 | 80 | 81 | 76 | 81 | 80 | 80 | 80 | 82 | 79 | 81 | 79 | 81 | 78 | 81 | 83 | 80 | 81 | 79 |
| **La** | 9.2 | 8.9 | 9.1 | 8.9 | 8.7 | 9.1 | 9.1 | 8.9 | 8.9 | 9.1 | 8.8 | 9.1 | 8.8 | 9.2 | 8.8 | 9.1 | 9.1 | 9.0 | 9.1 | 8.9 |
| **Ce** | 23 | 23 | 23 | 23 | 22 | 24 | 23 | 23 | 23 | 24 | 22 | 24 | 23 | 23 | 23 | 23 | 23 | 23 | 23 | 23 |
| **Pr** | 3.6 | 3.3 | 3.4 | 3.4 | 3.1 | 3.6 | 3.4 | 3.4 | 3.4 | 3.5 | 3.3 | 3.5 | 3.3 | 3.5 | 3.4 | 3.5 | 3.6 | 3.4 | 3.5 | 3.4 |
| **Nd** | 17 | 16 | 17 | 17 | 17 | 18 | 17 | 17 | 17 | 17 | 16 | 17 | 16 | 17 | 16 | 17 | 16 | 17 | 17 | 16 |
| **Sm** | 4.9 | 4.5 | 4.7 | 4.6 | 4.4 | 4.7 | 4.8 | 4.7 | 4.7 | 5.0 | 4.6 | 4.9 | 4.7 | 4.9 | 4.9 | 4.7 | 5.2 | 4.7 | 4.8 | 4.5 |
| **Eu** | 1.69 | 1.66 | 1.72 | 1.67 | 1.50 | 1.82 | 1.64 | 1.67 | 1.63 | 1.70 | 1.60 | 1.71 | 1.62 | 1.71 | 1.58 | 1.63 | 1.73 | 1.73 | 1.70 | 1.68 |
| **Gd** | 5.4 | 5.1 | 5.1 | 5.3 | 5.0 | 4.8 | 5.3 | 5.2 | 5.4 | 5.4 | 5.1 | 5.4 | 5.1 | 5.5 | 5.2 | 6.1 | 5.2 | 5.2 | 5.3 | 5.2 |
| **Tb** | 0.792 | 0.771 | 0.828 | 0.786 | 0.756 | 0.801 | 0.800 | 0.810 | 0.787 | 0.806 | 0.764 | 0.841 | 0.784 | 0.811 | 0.763 | 0.798 | 0.833 | 0.791 | 0.837 | 0.781 |
| **Dy** | 5.0 | 4.6 | 4.8 | 4.8 | 4.6 | 4.8 | 4.8 | 4.8 | 4.9 | 4.9 | 4.7 | 5.0 | 4.8 | 5.0 | 4.8 | 4.9 | 5.0 | 4.9 | 5.0 | 4.7 |
| **Ho** | 0.926 | 0.906 | 0.920 | 0.887 | 0.910 | 0.904 | 0.932 | 0.897 | 0.898 | 0.932 | 0.887 | 0.915 | 0.876 | 0.923 | 0.894 | 0.891 | 0.919 | 0.897 | 0.931 | 0.874 |
| **Er** | 2.54 | 2.31 | 2.47 | 2.32 | 2.36 | 2.56 | 2.49 | 2.41 | 2.42 | 2.50 | 2.36 | 2.53 | 2.38 | 2.53 | 2.44 | 2.40 | 2.44 | 2.43 | 2.51 | 2.39 |
| **Tm** | 0.317 | 0.306 | 0.322 | 0.311 | 0.317 | 0.360 | 0.321 | 0.332 | 0.324 | 0.329 | 0.316 | 0.340 | 0.334 | 0.345 | 0.318 | 0.279 | 0.340 | 0.320 | 0.336 | 0.315 |
| **Yb** | 2.09 | 1.96 | 2.02 | 2.11 | 2.09 | 1.76 | 2.10 | 2.08 | 2.06 | 2.08 | 2.01 | 2.13 | 2.06 | 2.07 | 2.05 | 2.41 | 1.98 | 2.01 | 2.11 | 2.05 |
| **Lu** | 0.289 | 0.265 | 0.287 | 0.270 | 0.251 | 0.252 | 0.289 | 0.276 | 0.286 | 0.294 | 0.281 | 0.299 | 0.291 | 0.302 | 0.286 | 0.307 | 0.334 | 0.293 | 0.285 | 0.283 |
| **Hf** | 3.4 | 3.1 | 3.2 | 3.2 | 3.2 | 3.1 | 3.3 | 3.2 | 3.1 | 3.3 | 3.2 | 3.2 | 3.1 | 3.3 | 3.2 | 3.3 | 3.3 | 3.3 | 3.3 | 3.1 |
| **Ta** | 0.570 | 0.523 | 0.574 | 0.543 | 0.480 | 0.517 | 0.566 | 0.549 | 0.551 | 0.587 | 0.542 | 0.578 | 0.550 | 0.541 | 0.563 | 0.614 | 0.609 | 0.555 | 0.551 | 0.540 |
| **Pb** | 1.36 | 1.37 | 1.34 | 1.39 | 1.19 | 1.39 | 1.35 | 1.41 | 1.37 | 1.46 | 1.35 | 1.43 | 1.36 | 1.45 | 1.34 | 1.43 | 1.51 | 1.39 | 1.33 | 1.38 |
| **Th** | 0.550 | 0.505 | 0.570 | 0.545 | 0.518 | 0.606 | 0.536 | 0.551 | 0.553 | 0.574 | 0.538 | 0.587 | 0.550 | 0.542 | 0.535 | 0.567 | 0.501 | 0.553 | 0.562 | 0.518 |
| **U** | 0.451 | 0.425 | 0.437 | 0.429 | 0.474 | 0.413 | 0.416 | 0.437 | 0.458 | 0.457 | 0.428 | 0.464 | 0.429 | 0.470 | 0.432 | 0.437 | 0.443 | 0.457 | 0.450 | 0.433 |

| **Standard** | NIST610 | NIST610 | NIST610 | NIST610 | NIST610 | NIST610 | NIST610 | NIST610 | NIST610 | NIST610 | NIST614 | NIST614 | NIST614 | NIST614 | NIST614 | NIST614 | NIST614 | NIST614 |
| --- | --- | --- | --- | --- | --- | --- | --- | --- | --- | --- | --- | --- | --- | --- | --- | --- | --- | --- |
| **Li** | 422 | 425 | 403 | 440 | 443 | 411 | 419 | 403 | 437 | 432 | 1.81 | 1.76 | 2.18 | 2.22 | 1.97 | 1.87 | 2.32 | 2.20 |
| **Be** | 452 | 442 | 300 | 456 | 460 | 436 | 436 | 296 | 458 | 454 | 0.653 | 0.802 | 0.733 | 0.740 | 0.556 | 0.808 | 0.691 | 0.806 |
| **B** | 131 | 169 | 266 | 163 | 192 | 131 | 165 | 245 | 163 | 187 | 5.3 | 3.7 | 3.7 | 3.3 | 5.9 | 3.4 | 3.4 | 3.4 |
| **Sc** | 504 | 543 | 499 | 529 | 548 | 492 | 550 | 510 | 526 | 550 | 11.3 | 6.2 | 7.7 | 6.6 | 11.5 | 6.7 | 7.7 | 6.9 |
| **V** | 416 | 417 | 421 | 427 | 423 | 411 | 418 | 416 | 423 | 425 | 1.02 | 1.07 | 1.27 | 1.35 | 1.08 | 1.06 | 1.27 | 1.38 |
| **Cr** | 475 | 472 | 469 | 481 | 479 | 467 | 474 | 464 | 476 | 478 | 2.15 | 2.21 | 2.33 | 2.32 | 2.24 | 2.06 | 2.33 | 2.28 |
| **Co** | 394 | 388 | 374 | 397 | 402 | 382 | 388 | 367 | 400 | 396 | 0.848 | 0.873 | 1.00 | 0.974 | 0.850 | 0.840 | 0.985 | 1.00 |
| **Ni** | 463 | 453 | 433 | 470 | 477 | 449 | 453 | 427 | 475 | 471 | 1.51 | 1.21 | 1.62 | 1.76 | 2.07 | 1.26 | 1.51 | 1.69 |
| **Cu** | 430 | 427 | 397 | 436 | 449 | 419 | 427 | 388 | 440 | 439 | 5.3 | 4.7 | 5.9 | 5.8 | 5.1 | 4.7 | 6.0 | 6.0 |
| **Zn** | 433 | 415 | 338 | 449 | 429 | 421 | 419 | 340 | 448 | 419 | 3.5 | 2.29 | 3.5 | 3.4 | 3.8 | 2.40 | 4.0 | 3.1 |
| **Rb** | 414 | 409 | 395 | 422 | 435 | 405 | 418 | 394 | 425 | 425 | 1.03 | 0.98 | 1.21 | 1.23 | 1.08 | 1.01 | 1.24 | 1.26 |
| **Sr** | 546 | 541 | 536 | 523 | 531 | 536 | 550 | 543 | 525 | 522 | 49 | 49 | 50 | 51 | 50 | 49 | 50 | 51 |
| **Y** | 613 | 625 | 609 | 591 | 599 | 592 | 634 | 602 | 597 | 595 | 0.901 | 0.885 | 0.795 | 0.893 | 0.913 | 0.890 | 0.786 | 0.849 |
| **Zr** | 567 | 576 | 562 | 555 | 576 | 548 | 587 | 565 | 561 | 566 | 0.897 | 0.966 | 0.847 | 0.922 | 0.945 | 0.965 | 0.847 | 0.902 |
| **Nb** | 488 | 487 | 476 | 486 | 480 | 478 | 495 | 472 | 485 | 472 | 0.824 | 0.865 | 0.869 | 0.871 | 0.873 | 0.857 | 0.828 | 0.842 |
| **Mo** | 348 | 353 | 334 | 374 | 372 | 339 | 358 | 338 | 377 | 366 | 0.729 | 0.872 | 1.09 | 1.04 | 0.759 | 0.817 | 1.05 | 0.955 |
| **Cs** | 370 | 311 | 311 | 356 | 352 | 367 | 316 | 313 | 361 | 344 | 0.794 | 0.654 | 0.944 | 0.901 | 0.853 | 0.652 | 0.926 | 0.941 |
| **Ba** | 458 | 454 | 456 | 460 | 460 | 449 | 459 | 449 | 458 | 451 | 3.4 | 3.4 | 3.6 | 3.6 | 3.6 | 3.1 | 3.8 | 3.6 |
| **La** | 500 | 501 | 490 | 479 | 491 | 486 | 506 | 486 | 483 | 478 | 0.768 | 0.750 | 0.741 | 0.756 | 0.761 | 0.763 | 0.716 | 0.758 |
| **Ce** | 462 | 459 | 456 | 434 | 449 | 447 | 456 | 451 | 433 | 437 | 0.856 | 0.882 | 0.909 | 0.925 | 0.903 | 0.849 | 0.964 | 0.939 |
| **Pr** | 486 | 488 | 480 | 460 | 472 | 474 | 492 | 480 | 456 | 466 | 0.838 | 0.865 | 0.860 | 0.848 | 0.871 | 0.813 | 0.865 | 0.841 |
| **Nd** | 497 | 498 | 483 | 481 | 493 | 480 | 496 | 484 | 479 | 481 | 0.790 | 0.850 | 0.800 | 0.780 | 0.768 | 0.737 | 0.797 | 0.758 |
| **Sm** | 528 | 537 | 533 | 516 | 538 | 517 | 534 | 528 | 514 | 525 | 0.840 | 0.887 | 0.832 | 0.826 | 0.861 | 0.840 | 0.806 | 0.815 |
| **Eu** | 496 | 495 | 476 | 478 | 469 | 483 | 489 | 463 | 473 | 464 | 0.840 | 0.841 | 0.807 | 0.793 | 0.821 | 0.792 | 0.800 | 0.786 |
| **Gd** | 573 | 587 | 520 | 557 | 585 | 555 | 582 | 521 | 557 | 574 | 0.763 | 0.851 | 0.796 | 0.799 | 0.905 | 0.761 | 0.798 | 0.799 |
| **Tb** | 547 | 569 | 554 | 511 | 551 | 528 | 564 | 547 | 514 | 539 | 0.814 | 0.866 | 0.741 | 0.784 | 0.831 | 0.838 | 0.760 | 0.794 |
| **Dy** | 555 | 566 | 554 | 547 | 569 | 537 | 560 | 549 | 545 | 557 | 0.834 | 0.908 | 0.745 | 0.816 | 0.795 | 0.847 | 0.761 | 0.842 |
| **Ho** | 590 | 588 | 572 | 532 | 573 | 567 | 585 | 572 | 533 | 559 | 0.805 | 0.822 | 0.755 | 0.776 | 0.881 | 0.883 | 0.759 | 0.794 |
| **Er** | 584 | 591 | 577 | 573 | 619 | 557 | 590 | 571 | 576 | 604 | 0.777 | 0.880 | 0.728 | 0.855 | 0.844 | 0.847 | 0.749 | 0.827 |
| **Tm** | 591 | 594 | 533 | 537 | 574 | 566 | 591 | 521 | 536 | 557 | 0.811 | 0.846 | 0.759 | 0.793 | 0.826 | 0.792 | 0.738 | 0.776 |
| **Yb** | 618 | 584 | 629 | 568 | 600 | 594 | 587 | 618 | 563 | 584 | 0.909 | 0.827 | 0.779 | 0.805 | 0.908 | 0.839 | 0.763 | 0.829 |
| **Lu** | 568 | 610 | 570 | 534 | 611 | 546 | 621 | 568 | 531 | 591 | 0.786 | 0.857 | 0.769 | 0.825 | 0.831 | 0.880 | 0.734 | 0.798 |
| **Hf** | 545 | 557 | 503 | 531 | 576 | 521 | 565 | 493 | 532 | 560 | 0.750 | 0.758 | 0.723 | 0.797 | 0.817 | 0.773 | 0.705 | 0.776 |
| **Ta** | 583 | 575 | 560 | 550 | 591 | 556 | 585 | 552 | 542 | 574 | 0.891 | 0.881 | 0.833 | 0.857 | 0.907 | 0.882 | 0.815 | 0.846 |
| **Pb** | 462 | 449 | 395 | 466 | 449 | 449 | 446 | 395 | 464 | 446 | 3.0 | 3.0 | 3.7 | 3.6 | 3.1 | 2.80 | 3.8 | 3.7 |
| **Th** | 558 | 582 | 513 | 507 | 557 | 533 | 590 | 503 | 503 | 543 | 0.772 | 0.844 | 0.722 | 0.822 | 0.816 | 0.798 | 0.728 | 0.813 |
| **U** | 414 | 413 | 418 | 411 | 432 | 404 | 425 | 408 | 406 | 420 | 0.889 | 0.857 | 1.07 | 1.07 | 1.07 | 0.859 | 1.17 | 1.08 |
