# Supplementary material for: Vitruvian binders in Venice: First evidence of Phlegraean pozzolans in an underwater Roman construction in the Venice Lagoon
Source: PLoS One. 2024 Nov 22;19(11):e0313917. doi: 10.1371/journal.pone.0313917 (PMC11584134; doi:10.1371/journal.pone.0313917)

**S1 Fig. Roman well-cisterns**.

Examples of Roman well-cistern documented in the site of Ca’ Ballarin in the Northern Lagoon of Venice (up) and from Aquileia (down). Images taken from [49] (see references in the main text).


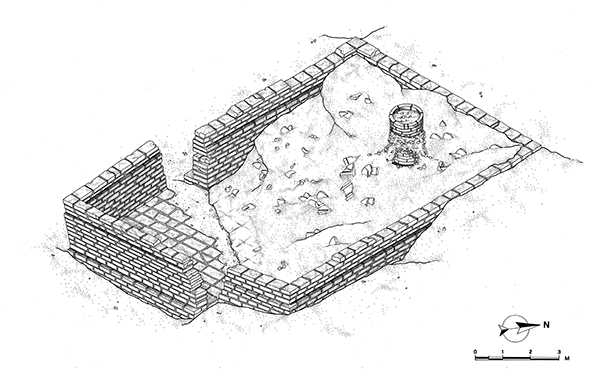


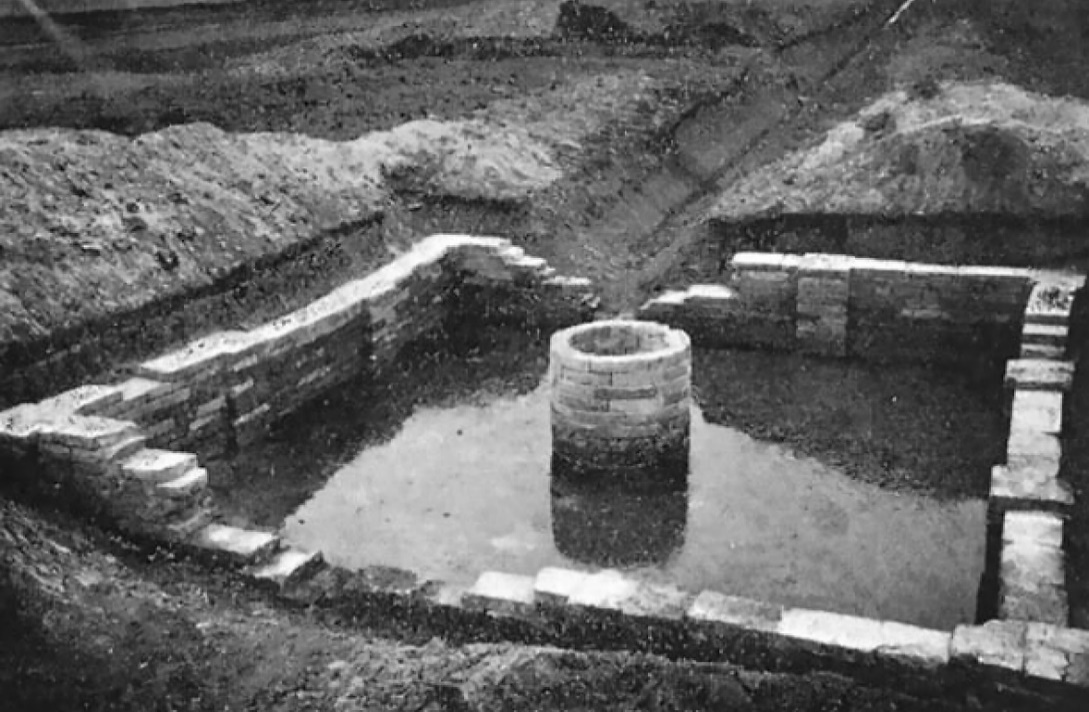

Supplement: S1 Fig — Examples of Roman well-cistern documented in the site of Ca’ Ballarin in the Northern Lagoon of Venice (up) and from Aquileia (down). Images taken from [49] (see references in the main text). (DOCX) [file pone.0313917.s002.docx]
