# Supplementary figures and images for: Vitruvian binders in Venice: First evidence of Phlegraean pozzolans in an underwater Roman construction in the Venice Lagoon
Source: PLoS One. 2024 Nov 22;19(11):e0313917. doi: 10.1371/journal.pone.0313917 (PMC11584134; doi:10.1371/journal.pone.0313917)

**S2 Fig.** **SEM-EDS investigation of** **mineral chemistry of volcanic tephra and iron slags**.


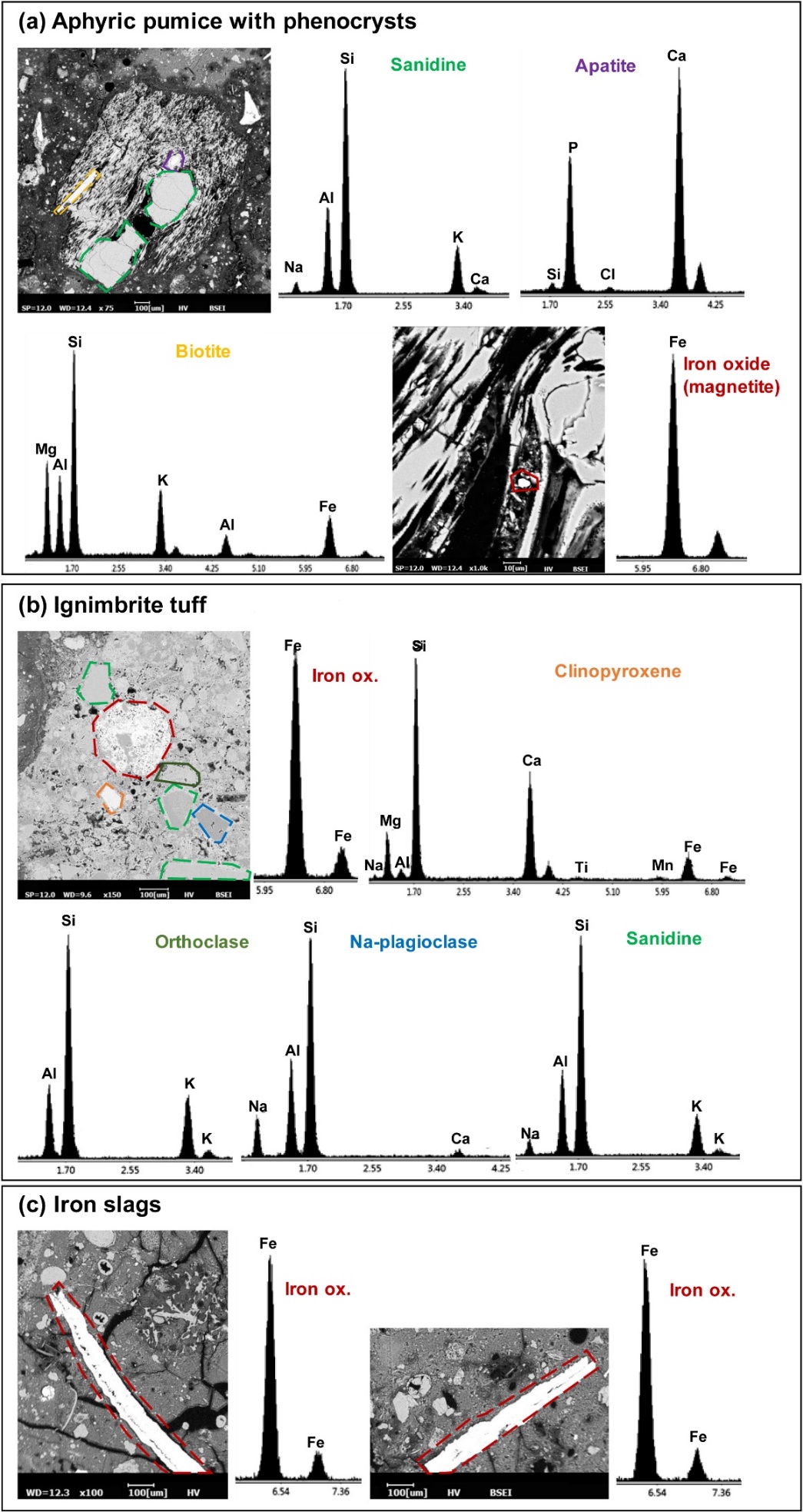

Supplement: S2 Fig — (DOCX) [file pone.0313917.s003.docx]
