# Supplementary material for: Vitruvian binders in Venice: First evidence of Phlegraean pozzolans in an underwater Roman construction in the Venice Lagoon
Source: PLoS One. 2024 Nov 22;19(11):e0313917. doi: 10.1371/journal.pone.0313917 (PMC11584134; doi:10.1371/journal.pone.0313917)

**S3 Fig.** **Trace elements’ scatterplots of the pumice clasts in TSF_T9A and TSF_T9B analyzed by LA-ICP-MS**

**a)** Nb/Zr vs Th/Ta scatterplot of pumice clasts in relation to the fields occupied by the Roman, Tuscan and Campanian magmatic provinces and Aeolian Arc Islands’ volcanic products (compositional fields from [42] and references therein); **b)** Nb/Y vs Zr/Y scatterplot of clasts’ samples in relation to the fields occupied by the Roman, Tuscan and Campanian magmatic provinces and the Aeolian Arc Island's products (compositional fields from [42] and references therein).


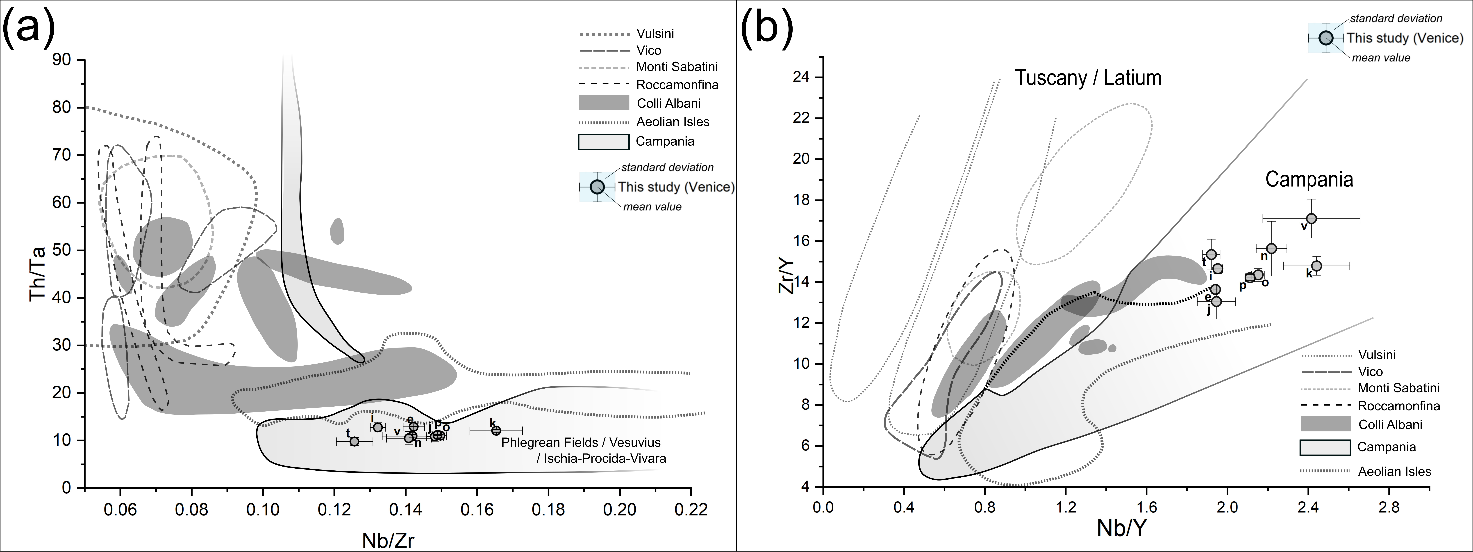

Supplement: S3 Fig — a) Nb/Zr vs Th/Ta scatterplot of pumice clasts in relation to the fields occupied by the Roman, Tuscan and Campanian magmatic provinces and Aeolian Arc Islands’ volcanic products (compositional fields from [42] and references therein); b) Nb/Y vs Zr/Y scatterplot of clasts’ samples in relation to the fields occupied by the Roman, Tuscan and Campanian magmatic provinces, and the Aeolian Arc Island’s products (compositional fields from [42] and references therein). (DOCX) [file pone.0313917.s004.docx]
