# Supplementary material for: Vitruvian binders in Venice: First evidence of Phlegraean pozzolans in an underwater Roman construction in the Venice Lagoon
Source: PLoS One. 2024 Nov 22;19(11):e0313917. doi: 10.1371/journal.pone.0313917 (PMC11584134; doi:10.1371/journal.pone.0313917)
Supplement: S1 Table — Multiple areas were analyzed by SEM-EDS and described as %Ox (average values and standard deviation); b.d. = Components below the detection limit. (DOCX) [file pone.0313917.s005.docx]

**S1 Table**. **Major elements composition of the binder matrix and binder-filled pumice vesicles**

Multiple areas were analyzed by SEM-EDS and described as %Ox (average values and standard deviation); b.d. = Components below the detection limit.

| **Area** | **Na_2_O** | **MgO** | **Al_2_O_3_** | **SiO_2_** | **P_2_O_5_** | **SO_3_** | **Cl_2_O** | **K_2_O** | **CaO** | **TiO_2_** | **Fe_2_O_3_** | **MgO/**  **SiO_2_+ Al_2_O_3_** | **SiO_2_/Al_2_O_3_** |
| --- | --- | --- | --- | --- | --- | --- | --- | --- | --- | --- | --- | --- | --- |
| *Binder Matrix* | | | | | | | | | | | | | |
| Area 1 | 1.65 | 28.50 | 13.00 | 44.23 | b.d. | 0.37 | 0.61 | 0.61 | 6.07 | 0.49 | 4.48 | 0.50 | 3.40 |
| Area 2 | 0.96 | 27.17 | 13.78 | 45.77 | b.d. | 0.18 | 0.23 | 0.62 | 5.95 | b.d. | 5.34 | 0.46 | 3.32 |
| Area 3 | b.d. | 26.53 | 16.40 | 47.00 | b.d. | b.d. | b.d. | 0.81 | 5.61 | b.d. | 3.64 | 0.42 | 2.87 |
| Area 4 | b.d. | 27.30 | 17.13 | 45.05 | b.d. | 0.53 | 0.37 | b.d. | 4.70 | 0.47 | 4.46 | 0.44 | 2.63 |
| Area 5 | b.d. | 25.51 | 15.93 | 41.29 | 0.04 | 0.28 | 0.36 | b.d. | 1.86 | 2.56 | 12.17 | 0.45 | 2.59 |
| Area 6 | b.d. | 27.08 | 17.06 | 44.96 | 0.10 | 0.36 | 0.58 | 0.51 | 5.52 | 0.85 | 2.98 | 0.44 | 2.64 |
| Area 7 | 0.50 | 29.33 | 12.46 | 52.12 | b.d. | b.d. | 0.60 | b.d. | 3.39 | b.d. | 1.60 | 0.45 | 4.18 |
| Area 8 | b.d. | 26.64 | 12.58 | 51.46 | b.d. | b.d. | 0.46 | b.d. | 4.57 | b.d. | 4.29 | 0.42 | 4.09 |
| Area 9 | 3.23 | 23.60 | 15.97 | 44.80 | 0.19 | 0.73 | 2.67 | 0.80 | 5.46 | b.d. | 2.55 | 0.39 | 2.81 |
| Area 10 | 2.99 | 23.34 | 15.75 | 43.42 | b.d. | 1.52 | 2.99 | 0.60 | 6.55 | b.d. | 2.84 | 0.39 | 2.76 |
| Area 11 | 5.76 | 25.37 | 13.29 | 41.52 | b.d. | 0.44 | 6.60 | b.d. | 4.32 | b.d. | 2.69 | 0.46 | 3.12 |
| MEAN | 1.37 | 26.40 | 14.85 | 45.60 | 0.03 | 0.40 | 1.41 | 0.36 | 4.91 | 0.40 | 4.28 | 0.44 | 3.13 |
| SD | 1.80 | 1.76 | 1.75 | 3.33 | 0.06 | 0.42 | 1.89 | 0.34 | 1.29 | 0.74 | 2.70 | 0.03 | 0.54 |
| *Binder-filled pumice vesicles* | | | | | | | | | | | | | |
| Area 1 | b.d. | 31.44 | 12.28 | 50.40 | b.d. | b.d. | 0.53 | b.d. | 1.79 | 0.79 | 2.77 | 0.50 | 4.10 |
| Area 2 | 1.84 | 28.50 | 10.96 | 47.39 | b.d. | b.d. | 1.08 | 0.89 | 1.75 | 1.39 | 6.20 | 0.49 | 4.32 |
| Area 3 | b.d. | 28.21 | 11.07 | 52.60 | 0.16 | b.d. | 1.70 | 0.40 | 0.92 | 1.75 | 3.19 | 0.44 | 4.75 |
| Area 4 | 1.72 | 28.59 | 11.79 | 50.67 | b.d. | b.d. | 0.71 | 0.34 | 1.55 | 0.94 | 3.68 | 0.46 | 4.30 |
| Area 5 | 1.08 | 29.03 | 11.15 | 48.29 | b.d. | b.d. | 0.49 | 0.47 | 1.42 | b.d. | 8.08 | 0.49 | 4.33 |
| Area 6 | 1.23 | 29.16 | 10.63 | 52.81 | b.d. | b.d. | 0.76 | 0.47 | 0.96 | b.d. | 3.99 | 0.46 | 4.97 |
| Area 7 | 1.39 | 25.63 | 12.52 | 49.49 | b.d. | b.d. | 0.81 | b.d. | 1.28 | 1.10 | 7.78 | 0.41 | 3.95 |
| Area 8 | 1.41 | 30.26 | 10.88 | 50.47 | b.d. | b.d. | 0.75 | b.d. | 1.37 | 1.12 | 3.74 | 0.49 | 4.64 |
| MEAN | 1.08 | 28.85 | 11.41 | 50.27 | 0.02 | b.d. | 0.85 | 0.32 | 1.38 | 0.89 | 4.93 | 0.47 | 4.42 |
| SD | 0.67 | 1.57 | 0.65 | 1.76 | 0.05 | b.d. | 0.36 | 0.29 | 0.30 | 0.58 | 1.98 | 0.03 | 0.32 |
