# Supplementary material for: Vitruvian binders in Venice: First evidence of Phlegraean pozzolans in an underwater Roman construction in the Venice Lagoon
Source: PLoS One. 2024 Nov 22;19(11):e0313917. doi: 10.1371/journal.pone.0313917 (PMC11584134; doi:10.1371/journal.pone.0313917)
Supplement: S2 Table — Clasts were analyzed by SEM-EDS and described as %wt in oxides (average values and standard deviation); b.d. = Components below the detection limit. (DOCX) [file pone.0313917.s006.docx]

**S2 Table**. **Major elements composition of the** **volcanic tephra**

Clasts were analyzed by SEM-EDS and described as %Ox (average values and standard deviation); b.d. = Components below the detection limit.

| **Sample** | ***Clast*** | **Type** | **%ox**  **st.dev.** | **Na_2_O** | **MgO** | **Al_2_O_3_** | **SiO_2_** | **P_2_O_5_** | **SO_3_** | **Cl_2_O** | **K_2_O** | **CaO** | **TiO_2_** | **MnO** | **Fe_2_O_3_** | **Na_2_O + K_2_O** |
| --- | --- | --- | --- | --- | --- | --- | --- | --- | --- | --- | --- | --- | --- | --- | --- | --- |
| TSF_T9A | *01* | Aph. pumice | %ox | 4.65 | 0.96 | 19.68 | 55.75 | 0.01 | 0.03 | 0.11 | 9.43 | 3.35 | 0.96 | 0.25 | 4.80 | 14.08 |
|  |  |  | *st.dev.* | *0.60* | *0.51* | *0.38* | *1.81* | *0.02* | *0.06* | *0.32* | *0.42* | *0.21* | *0.57* | *0.37* | *1.48* | *0.77* |
|  | *02* | Aph. pumice | %ox | 6.63 | 1.11 | 19.36 | 57.50 | 0.04 | 0.04 | b.d. | 8.03 | 2.96 | 0.59 | 0.32 | 3.42 | 14.66 |
|  |  |  | *st.dev.* | *0.24* | *0.17* | *0.29* | *0.56* | *0.08* | *0.10* | *b.d.* | *0.22* | *0.28* | *0.30* | *0.27* | *0.23* | *0.36* |
|  | *03* | Aph. pumice | %ox | 5.94 | 1.22 | 19.52 | 56.91 | b.d. | b.d. | 0.18 | 8.14 | 3.16 | 0.84 | 0.23 | 3.85 | 14.08 |
|  |  |  | *st.dev.* | *0.25* | *0.18* | *0.19* | *0.51* | *b.d.* | *b.d.* | *0.46* | *0.21* | *0.18* | *0.14* | *0.29* | *0.14* | *0.32* |
|  | *04* | Aphyric pumice | %ox | 6.90 | 1.00 | 19.50 | 56.30 | b.d. | b.d. | 1.20 | 8.10 | 2.50 | 0.90 | b.d. | 3.60 | 14.90 |
|  |  |  | *st.dev.* | *0.30* | *0.30* | *0.20* | *1.80* | *b.d.* | *b.d.* | *0.10* | *0.40* | *0.20* | *0.30* | *b.d.* | *0.30* | *0.60* |
|  | *05* | Weakly-lithified Tuff | %ox | 4.10 | 5.20 | 19.50 | 58.20 | b.d. | b.d. | 0.40 | 8.40 | 1.10 | 0.50 | b.d. | 2.70 | 12.50 |
|  |  |  | *st.dev.* | *1.20* | *2.40* | *1.10* | *2.00* | *b.d.* | *b.d.* | *0.30* | *1.40* | *0.40* | *0.10* | *b.d.* | *1.20* | *1.10* |
|  | *06* | Aphyric pumice | %ox | 6.40 | 0.50 | 19.70 | 58.70 | b.d. | b.d. | 0.90 | 8.50 | 2.10 | 0.50 | b.d. | 2.80 | 14.90 |
|  |  |  | *st.dev.* | *1.10* | *0.40* | *0.50* | *2.00* | *b.d.* | *b.d.* | *0.40* | *1.90* | *0.60* | *0.40* | *b.d.* | *1.30* | *1.00* |
|  | *07* | Obsidian | %ox | 6.60 | 1.10 | 19.50 | 56.70 | b.d. | b.d. | 1.10 | 8.10 | 2.60 | 0.70 | b.d. | 3.60 | 14.70 |
|  |  |  | *st.dev.* | *0.20* | *0.10* | *0.20* | *0.80* | *b.d.* | *b.d.* | *0.10* | *0.10* | *0.20* | *0.30* | *b.d.* | *0.20* | *0.20* |
|  | *09* | Weakly-lithified Tuff | %ox | 5.60 | 1.20 | 19.20 | 62.40 | b.d. | b.d. | 0.40 | 9.00 | 0.50 | b.d. | b.d. | 2.00 | 14.70 |
|  |  |  | *st.dev.* | *1.10* | *0.20* | *0.50* | *0.30* | *b.d.* | *b.d.* | *0.60* | *1.50* | *0.20* | *b.d.* | *b.d.* | *0.60* | *0.50* |
| TSF_T9B | *a* | Weakly-lithified Tuff | %ox | 5.78 | 4.54 | 17.89 | 60.78 | b.d. | 0.04 | 0.36 | 5.94 | 0.92 | 0.87 | 0.23 | 2.68 | 11.73 |
|  |  |  | *st.dev.* | *1.20* | *1.40* | *0.47* | *1.11* | *b.d.* | *0.09* | *0.18* | *0.76* | *0.64* | *0.29* | *0.38* | *0.53* | *1.47* |
|  | *b* | Aph. pumice | %ox | 6.96 | 0.77 | 19.11 | 57.12 | 0.01 | 0.06 | 1.01 | 7.92 | 2.42 | 0.66 | 0.38 | 3.57 | 14.88 |
|  |  |  | *st.dev.* | *0.28* | *0.20* | *0.27* | *0.81* | *0.02* | *0.07* | *0.05* | *0.20* | *0.13* | *0.21* | *0.20* | *0.38* | *0.35* |
|  | *c* | Porp. pumice | %ox | 6.24 | 2.31 | 18.76 | 59.61 | 0.10 | 0.14 | 0.64 | 6.06 | 1.66 | 0.68 | 0.56 | 3.24 | 12.30 |
|  |  |  | *st.dev.* | *0.34* | *0.51* | *0.31* | *0.53* | *0.10* | *0.09* | *0.12* | *0.25* | *0.17* | *0.10* | *0.15* | *0.26* | *0.33* |
|  | *d* | Aph. pumice | %ox | 8.09 | 0.64 | 18.40 | 59.29 | 0.02 | 0.16 | 0.84 | 6.29 | 2.00 | 0.71 | 0.57 | 2.99 | 14.37 |
|  |  |  | *st.dev.* | *0.37* | *0.07* | *0.17* | *1.21* | *0.03* | *0.05* | *0.17* | *0.09* | *0.18* | *0.12* | *0.21* | *0.27* | *0.41* |
|  | *e* | Aph. pumice | %ox | 4.80 | 1.00 | 19.20 | 56.50 | 0.00 | 0.20 | 0.80 | 8.80 | 3.20 | 0.80 | 0.30 | 4.30 | 13.60 |
|  |  |  | *st.dev.* | *0.50* | *0.20* | *0.20* | *1.50* | *0.00* | *0.20* | *0.20* | *0.30* | *0.30* | *0.40* | *0.20* | *0.80* | *0.30* |
|  | *f* | Porp. pumice | %ox | 5.54 | 2.68 | 18.94 | 59.44 | 0.01 | 0.04 | 0.19 | 8.07 | 1.31 | 0.81 | 0.44 | 2.51 | 13.61 |
|  |  |  | *st.dev.* | *0.91* | *0.66* | *0.41* | *1.33* | *0.03* | *0.09* | *0.25* | *1.42* | *0.33* | *0.43* | *0.23* | *0.65* | *0.92* |
|  | *j* | Aph. pumice | %ox | 6.05 | 0.94 | 19.17 | 56.27 | 0.03 | 0.04 | 0.84 | 8.72 | 2.93 | 0.89 | 0.51 | 3.62 | 14.78 |
|  |  |  | *st.dev.* | *0.17* | *0.16* | *0.28* | *0.88* | *0.05* | *0.09* | *0.09* | *0.20* | *0.16* | *0.24* | *0.25* | *0.18* | *0.20* |
|  | *k* | Porp. pumice | %ox | 6.66 | 0.91 | 19.42 | 57.22 | 0.02 | b.d. | 0.58 | 8.09 | 2.44 | 0.67 | 0.43 | 3.56 | 14.75 |
|  |  |  | *st.dev.* | *0.80* | *0.43* | *0.69* | *2.15* | *0.05* | *b.d.* | *0.36* | *0.65* | *0.28* | *0.39* | *0.28* | *1.33* | *0.32* |
|  | *g* | Aph. pumice | %ox | 6.31 | 1.31 | 18.87 | 55.97 | b.d. | 0.15 | 0.93 | 8.27 | 2.64 | 1.20 | 0.56 | 3.80 | 14.58 |
|  |  |  | *st.dev.* | *0.46* | *0.69* | *0.48* | *1.17* | *b.d.* | *0.14* | *0.13* | *0.30* | *0.12* | *0.34* | *0.28* | *0.33* | *0.50* |
|  | *l* | Aph. pumice | %ox | 8.59 | 2.85 | 20.51 | 58.98 | 0.08 | b.d. | 0.07 | 5.45 | 0.80 | 0.54 | 0.08 | 2.08 | 14.04 |
|  |  |  | *st.dev.* | *1.68* | *1.46* | *0.58* | *1.60* | *0.12* | *b.d.* | *0.16* | *1.88* | *0.25* | *0.53* | *0.19* | *0.78* | *1.01* |
|  | *h* | Aph. pumice | %ox | 6.45 | 1.28 | 19.57 | 56.41 | 0.07 | 0.05 | 0.66 | 7.91 | 2.70 | 0.76 | 0.45 | 3.68 | 14.35 |
|  |  |  | *st.dev.* | *1.02* | *0.38* | *0.36* | *0.98* | *0.11* | *0.08* | *0.12* | *0.71* | *0.31* | *0.25* | *0.25* | *0.39* | *0.39* |

*Continues*

| **Sample** | ***Clast*** | **Type** | **%ox**  **st.dev.** | **Na_2_O** | **MgO** | **Al_2_O_3_** | **SiO_2_** | **P_2_O_5_** | **SO_3_** | **Cl_2_O** | **K_2_O** | **CaO** | **TiO_2_** | **MnO** | **Fe_2_O_3_** | **Na_2_O + K_2_O** |
| --- | --- | --- | --- | --- | --- | --- | --- | --- | --- | --- | --- | --- | --- | --- | --- | --- |
| TSF_T9C | *i* | Aph. pumice | %ox | 5.29 | 0.99 | 19.01 | 57.11 | 0.03 | 0.10 | 0.86 | 8.00 | 2.72 | 0.93 | 0.56 | 3.73 | 13.96 |
|  |  |  | *st.dev.* | *0.56* | *0.15* | *0.28* | *0.64* | *0.01* | *0.12* | *0.09* | *0.23* | *0.14* | *0.12* | *0.07* | *0.40* | *0.53* |
|  | *m* | Porp. pumice | %ox | 6.40 | 1.11 | 19.52 | 55.44 | 0.08 | 0.10 | 1.07 | 8.32 | 2.69 | 0.95 | 0.39 | 3.97 | 14.72 |
|  |  |  | *st.dev.* | *0.48* | *0.23* | *0.56* | *0.83* | *0.07* | *0.06* | *0.24* | *0.25* | *0.21* | *0.27* | *0.26* | *0.38* | *0.25* |
|  | *n* | Aph. pumice | %ox | 6.26 | 0.92 | 19.40 | 57.68 | b.d. | 0.08 | 1.09 | 8.40 | 2.35 | 0.36 | 0.20 | 3.26 | 14.67 |
|  |  |  | *st.dev.* | *0.32* | *0.14* | *0.25* | *0.72* | *b.d.* | *0.08* | *0.12* | *0.15* | *0.20* | *0.21* | *0.21* | *0.60* | *0.35* |
|  | *o* | Aph. pumice | %ox | 5.70 | 1.01 | 19.45 | 57.95 | 0.02 | 0.08 | 1.11 | 9.10 | 2.81 | 0.39 | 0.33 | 2.04 | 14.80 |
|  |  |  | *st.dev.* | *0.87* | *0.24* | *0.29* | *0.38* | *0.04* | *0.08* | *0.06* | *0.60* | *0.27* | *0.09* | *0.09* | *0.28* | *0.45* |
|  | *p* | Aph. pumice | %ox | 6.21 | 1.03 | 19.61 | 58.06 | 0.01 | 0.07 | 1.08 | 8.83 | 2.48 | 0.38 | 0.25 | 2.00 | 15.03 |
|  |  |  | *st.dev.* | *0.41* | *0.15* | *0.19* | *0.41* | *0.02* | *0.09* | *0.09* | *0.21* | *0.10* | *0.06* | *0.12* | *0.10* | *0.39* |
|  | *r* | Porp. pumice | %ox | 7.44 | 0.72 | 19.51 | 58.87 | b.d. | b.d. | 0.63 | 7.27 | 2.09 | 0.69 | 0.30 | 2.48 | 14.71 |
|  |  |  | *st.dev.* | *0.36* | *0.19* | *1.22* | *1.69* | *b.d.* | *b.d.* | *0.48* | *0.56* | *0.48* | *0.28* | *0.38* | *1.67* | *0.43* |
|  | *s* | Weakly-lithified Tuff | %ox | 7.99 | 2.02 | 18.60 | 58.99 | b.d. | 0.02 | 0.32 | 6.58 | 1.17 | 0.76 | 0.53 | 3.03 | 14.56 |
|  |  |  | *st.dev.* | *1.14* | *0.19* | *0.42* | *1.16* | *b.d.* | *0.05* | *0.15* | *1.00* | *0.52* | *0.25* | *0.27* | *0.48* | *0.41* |
|  | *t* | Aph. pumice | %ox | 6.86 | 1.05 | 19.27 | 57.09 | 0.01 | 0.09 | 1.05 | 7.91 | 2.33 | 0.54 | 0.34 | 3.46 | 14.77 |
|  |  |  | *st.dev.* | *0.43* | *0.43* | *0.45* | *1.00* | *0.02* | *0.14* | *0.12* | *0.47* | *0.20* | *0.25* | *0.24* | *0.38* | *0.44* |
|  | *v* | Porp. pumice | %ox | 6.94 | 1.08 | 18.96 | 56.64 | 0.02 | 0.05 | 1.17 | 7.74 | 2.42 | 0.73 | 0.40 | 3.87 | 14.68 |
|  |  |  | *st.dev.* | *0.37* | *0.25* | *0.40* | *1.11* | *0.05* | *0.06* | *0.28* | *0.26* | *0.20* | *0.35* | *0.21* | *0.32* | *0.41* |
|  | *z* | Weakly-lithified Tuff | %ox | 7.91 | 2.01 | 17.86 | 58.88 | b.d. | b.d. | 0.25 | 4.70 | 5.80 | 0.29 | 0.62 | 1.69 | 12.61 |
|  |  |  | *st.dev.* | *0.76* | *1.00* | *1.02* | *2.46* | *b.d.* | *b.d.* | *0.15* | *0.50* | *4.05* | *0.22* | *0.35* | *0.49* | *0.43* |
