# Supplementary material for: Vitruvian binders in Venice: First evidence of Phlegraean pozzolans in an underwater Roman construction in the Venice Lagoon
Source: PLoS One. 2024 Nov 22;19(11):e0313917. doi: 10.1371/journal.pone.0313917 (PMC11584134; doi:10.1371/journal.pone.0313917)
Supplement: S3 Table — Chemical results of the selected clasts of pumice analyzed by LA-ICP-MS (average values of multiple spot-size analyses are reported and related standard deviation). Values are described as ppm. (DOCX) [file pone.0313917.s007.docx]

**S3 Table. Trace elements composition of the** **volcanic tephra**

Chemical results of the selected clasts of pumice analyzed by LA-ICP-MS (average values of multiple spot-size analyses are reported and related standard deviation). Values are described as ppm.

| **Clast** | ***e*** | | ***j*** | | ***k*** | | ***l*** | | ***n*** | | ***o*** | | ***p*** | | ***t*** | | ***v*** | |
| --- | --- | --- | --- | --- | --- | --- | --- | --- | --- | --- | --- | --- | --- | --- | --- | --- | --- | --- |
| **Type** | *Aphyric*  *Pumice* | | *Aphyric*  *Pumice* | | *Porphyritic*  *Pumice* | | *Aphyric*  *Pumice* | | *Aphyric*  *Pumice* | | *Aphyric*  *Pumice* | | *Aphyric*  *Pumice* | | *Aphyric*  *Pumice* | | *Porphyritic*  *Pumice* | |
| ppm/st.dev. | ppm | *st.dev.* | ppm | *st.dev.* | ppm | *st.dev.* | ppm | *st.dev.* | ppm | *st.dev.* | ppm | *st.dev.* | ppm | *st.dev.* | ppm | *st.dev.* | ppm | *st.dev.* |
| Li | 62.3 | *7.9* | 71.4 | *16.3* | 69.2 | *18.0* | 63.1 | *3.5* | 72.1 | *1.5* | 64.2 | *1.6* | 61.1 | *0.2* | 71.6 | *7.1* | 61.0 | *5.5* |
| Be | 13.8 | *0.2* | 15.3 | *1.1* | 19.3 | *2.1* | 16.0 | *0.5* | 17.6 | *0.6* | 16.9 | *0.8* | 15.7 | *0.7* | 12.5 | *1.6* | 13.4 | *1.6* |
| B | 70.7 | *12.9* | 80.4 | *21.3* | 67.3 | *6.7* | 49.7 | *3.8* | 49.4 | *4.3* | 43.1 | *2.1* | 40.5 | *1.5* | 96.0 | *5.4* | 102.7 | *19.4* |
| Sc | 9.2 | *0.5* | 8.5 | *1.0* | 7.6 | *0.3* | 9.4 | *0.3* | 9.1 | *0.5* | 10.2 | *0.3* | 9.5 | *0.3* | 7.6 | *3.0* | 8.5 | *1.1* |
| V | 76.8 | *1.9* | 70.9 | *2.5* | 41.2 | *5.6* | 56.4 | *2.7* | 35.4 | *1.0* | 54.9 | *2.5* | 46.5 | *1.2* | 34.9 | *1.7* | 37.6 | *8.5* |
| Cr | 10.2 | *1.9* | 6.1 | *1.6* | 2.5 | *2.4* | 11.3 | *1.4* | 4.2 | *2.4* | 1.6 | *0.5* | 1.4 | *0.4* | 9.0 | *3.9* | 10.9 | *3.3* |
| Co | 6.8 | *0.7* | 5.0 | *1.4* | 3.6 | *0.4* | 4.8 | *0.4* | 3.4 | *0.7* | 4.0 | *0.2* | 3.4 | *0.1* | 3.1 | *0.4* | 2.9 | *0.6* |
| Ni | 9.4 | *2.8* | 10.2 | *4.6* | 6.5 | *11.7* | 13.8 | *1.4* | 2.7 | *2.3* | 0.6 | *0.5* | 0.3 | *0.1* | 4.5 | *2.9* | 4.0 | *1.8* |
| Cu | 14.6 | *2.7* | 12.9 | *6.2* | 6.8 | *4.3* | 17.3 | *1.2* | 8.0 | *2.3* | 7.2 | *0.8* | 6.9 | *0.2* | 7.7 | *1.6* | 8.8 | *1.6* |
| Zn | 67.8 | *6.5* | 79.8 | *6.1* | 136.5 | *25.8* | 55.8 | *2.0* | 102.3 | *26.2* | 124.1 | *7.2* | 120.2 | *1.6* | 54.0 | *23.7* | 67.1 | *14.8* |
| Rb | 396.9 | *3.8* | 410.5 | *24.8* | 544.9 | *36.5* | 348.6 | *7.7* | 468.5 | *41.0* | 486.9 | *11.1* | 448.2 | *8.4* | 459.4 | *27.8* | 480.4 | *43.8* |
| Sr | 475.0 | *2.1* | 345.5 | *23.6* | 110.5 | *42.5* | 268.0 | *6.5* | 118.5 | *29.8* | 216.6 | *30.6* | 177.7 | *21.5* | 88.9 | *3.6* | 151.6 | *114.0* |
| Y | 48.6 | *1.5* | 46.3 | *1.4* | 53.0 | *4.2* | 52.1 | *2.4* | 48.8 | *2.2* | 44.6 | *1.7* | 42.2 | *1.2* | 59.8 | *2.8* | 58.9 | *9.8* |
| Zr | 663.1 | *31.0* | 604.6 | *44.7* | 782.5 | *41.7* | 764.7 | *43.7* | 765.7 | *98.5* | 641.0 | *33.3* | 600.4 | *16.9* | 917.0 | *50.1* | 998.0 | *110.4* |
| Nb | 94.2 | *2.4* | 90.0 | *5.7* | 129.1 | *9.9* | 101.8 | *5.9* | 108.2 | *7.5* | 96.1 | *4.3* | 89.1 | *1.9* | 114.7 | *4.8* | 140.0 | *11.0* |
| Mo | 5.7 | *0.1* | 6.1 | *0.5* | 4.9 | *1.4* | 5.1 | *0.3* | 6.3 | *0.6* | 7.5 | *0.3* | 7.3 | *0.1* | 6.5 | *1.0* | 5.2 | *2.2* |
| Cs | 22.1 | *0.3* | 22.8 | *1.3* | 40.1 | *10.5* | 22.5 | *0.7* | 41.6 | *2.5* | 40.4 | *1.8* | 38.6 | *0.8* | 36.7 | *1.8* | 34.8 | *4.7* |
| Ba | 574.2 | *29.2* | 274.5 | *51.6* | 35.7 | *10.6* | 119.6 | *1.9* | 40.7 | *18.5* | 86.7 | *26.4* | 62.0 | *12.9* | 19.6 | *2.2* | 68.3 | *78.2* |
| La | 105.2 | *4.2* | 102.8 | *3.7* | 134.7 | *13.1* | 111.2 | *3.7* | 113.8 | *1.3* | 109.9 | *3.3* | 103.5 | *2.1* | 127.7 | *6.4* | 128.4 | *20.1* |
| Ce | 184.8 | *3.5* | 188.4 | *10.2* | 278.3 | *31.6* | 199.8 | *9.2* | 217.3 | *9.1* | 218.4 | *6.3* | 203.9 | *5.7* | 232.4 | *9.2* | 241.3 | *38.3* |
| Pr | 21.3 | *0.8* | 21.2 | *0.8* | 28.2 | *3.0* | 22.3 | *0.7* | 22.7 | *0.2* | 22.3 | *0.6* | 21.0 | *0.6* | 25.9 | *1.3* | 26.5 | *3.9* |
| Nd | 76.4 | *3.3* | 74.7 | *2.6* | 94.3 | *10.2* | 81.3 | *2.8* | 77.3 | *1.7* | 75.3 | *2.2* | 70.9 | *1.7* | 89.0 | *4.1* | 92.1 | *16.0* |
| Sm | 14.2 | *0.3* | 13.4 | *0.5* | 16.0 | *1.4* | 14.6 | *0.9* | 13.5 | *0.6* | 13.1 | *0.4* | 12.3 | *0.3* | 15.9 | *1.6* | 17.7 | *3.2* |
| Eu | 2.8 | *0.1* | 2.5 | *0.1* | 2.0 | *0.2* | 2.7 | *0.2* | 1.9 | *0.1* | 2.2 | *0.0* | 2.0 | *0.0* | 1.8 | *0.2* | 2.1 | *0.4* |
| Gd | 11.3 | *0.1* | 10.8 | *0.3* | 12.7 | *1.0* | 12.4 | *0.4* | 11.1 | *0.4* | 10.7 | *0.3* | 9.9 | *0.2* | 12.1 | *0.7* | 12.3 | *2.4* |
| Tb | 1.6 | *0.0* | 1.5 | *0.1* | 1.7 | *0.1* | 1.7 | *0.1* | 1.5 | *0.1* | 1.4 | *0.1* | 1.3 | *0.1* | 1.8 | *0.1* | 1.9 | *0.3* |
| Dy | 9.1 | *0.5* | 8.4 | *0.3* | 9.7 | *0.8* | 10.0 | *0.5* | 8.5 | *0.6* | 7.9 | *0.3* | 7.4 | *0.2* | 10.3 | *0.7* | 11.5 | *2.0* |
| Ho | 1.7 | *0.0* | 1.6 | *0.1* | 1.8 | *0.1* | 2.0 | *0.1* | 1.7 | *0.1* | 1.5 | *0.1* | 1.4 | *0.1* | 1.9 | *0.2* | 2.1 | *0.4* |
| Er | 4.9 | *0.2* | 4.6 | *0.2* | 5.2 | *0.3* | 5.5 | *0.3* | 4.8 | *0.4* | 4.4 | *0.2* | 4.1 | *0.1* | 6.1 | *0.6* | 5.9 | *0.6* |
| Tm | 0.8 | *0.0* | 0.7 | *0.0* | 0.8 | *0.1* | 0.9 | *0.1* | 0.8 | *0.1* | 0.7 | *0.0* | 0.6 | *0.0* | 0.8 | *0.1* | 0.9 | *0.1* |
| Yb | 4.9 | *0.1* | 4.6 | *0.2* | 5.3 | *0.4* | 5.9 | *0.3* | 5.4 | *0.4* | 4.8 | *0.2* | 4.6 | *0.1* | 6.6 | *0.5* | 6.6 | *0.7* |
| Lu | 0.7 | *0.0* | 0.7 | *0.0* | 0.8 | *0.1* | 0.8 | *0.1* | 0.7 | *0.0* | 0.7 | *0.0* | 0.6 | *0.0* | 0.9 | *0.1* | 0.9 | *0.2* |
| Hf | 14.7 | *0.7* | 13.8 | *1.6* | 16.3 | *0.9* | 16.5 | *1.1* | 16.0 | *2.6* | 13.3 | *0.8* | 12.5 | *0.3* | 18.8 | *2.0* | 20.5 | *1.7* |
| Ta | 6.5 | *0.4* | 6.0 | *0.7* | 6.9 | *0.5* | 7.1 | *0.6* | 7.2 | *1.7* | 5.5 | *0.6* | 5.0 | *0.1* | 8.4 | *0.9* | 10.7 | *1.2* |
| W | 6.7 | *0.2* | 6.9 | *0.3* | 6.6 | *1.6* | 5.9 | *0.1* | 8.0 | *0.6* | 9.7 | *0.3* | 9.1 | *0.2* | 9.3 | *0.6* | 8.5 | *1.3* |
| Pb | 138.0 | *8.8* | 155.2 | *28.7* | 142.2 | *33.5* | 114.6 | *9.5* | 142.7 | *51.6* | 96.9 | *9.2* | 87.0 | *3.5* | 114.0 | *17.0* | 171.6 | *25.3* |
| Th | 83.4 | *5.5* | 65.0 | *5.5* | 83.6 | *6.9* | 90.7 | *11.0* | 78.8 | *19.5* | 60.8 | *5.0* | 55.9 | *1.8* | 81.5 | *8.0* | 111.1 | *11.3* |
| U | 10.7 | *0.0* | 12.4 | *0.7* | 20.6 | *2.0* | 10.5 | *0.4* | 16.8 | *1.3* | 17.7 | *0.6* | 16.8 | *0.6* | 18.2 | *0.9* | 17.3 | *2.9* |

*Comment to the data*. Most of the clasts presents low standard deviation of the average values, indicative for the substantial efficacy of the LA-ICP MS acquisitions of the glassy matrix of the samples.

Yb is comprised between 4.6 and 6.6, with low SD, always < 0.5 with only clast *v* exceeding this (0.7); Y between 42.2 and 59.8 with st.dev. always < 5, except for clast v (9.8); Nb between 90 and 140, with st.dev. < 10 except clast *v* (11); Nd between 70.9 and 92.1 with very low st.dev., always < 3.5, apart for clasts *k* and *v* where it is > 10; Ta between 5.0 and 10.7, with st.dev. < 1 apart from clasts *n* and *v*, where it is 1.7 and 1.2 respectively.

Slightly greater but still acceptable variability distinguishes Zr, comprised between 604.6 and 998.0, with st.dev. between 31.0 and 50.1, while it is around 100 in clasts *n* and *v*; Rb, comprised between 396.9 and 544.9, whereas st.dev. is variable between 3.8 and 43.8 in clast *v*; Th, between 60.8 and 111.1, with st.dev. < 10 apart from clasts *v*, *i* and *n* where it is between 11 and 19.5.
