# Supplementary material for: Vitruvian binders in Venice: First evidence of Phlegraean pozzolans in an underwater Roman construction in the Venice Lagoon
Source: PLoS One. 2024 Nov 22;19(11):e0313917. doi: 10.1371/journal.pone.0313917 (PMC11584134; doi:10.1371/journal.pone.0313917)
Supplement: S4 Table — (DOCX) [file pone.0313917.s008.docx]

**S4 Table**. **Coefficients of the discriminant analysis**

**Classification Function Coefficients for Volcanic Activity of the Phlegraean Fields**

|  | ***CI*** | ***NYT*** | ***Post-NYT*** | ***Pre-CI*** | ***post CI*** | ***pre-NYT*** |
| --- | --- | --- | --- | --- | --- | --- |
| **Zr** | -0.0424407 | -0.0567724 | -0.000496821 | -0.036857 | -0.0740453 | -0.028277 |
| **Nb** | -0.28933 | -0.480032 | -0.514558 | -0.222188 | -0.559942 | -0.44933 |
| **Th** | -0.637694 | -0.22148 | -0.21731 | -0.559715 | 0.0896236 | -0.274493 |
| **Rb** | 0.248593 | 0.284685 | 0.235973 | 0.199206 | 0.246364 | 0.236843 |
| **Y** | 0.559243 | 1.02266 | 0.552035 | 0.900891 | 0.633252 | 0.683229 |
| **La** | -0.105198 | -0.209921 | -0.420803 | -0.0336309 | 0.240622 | -0.328778 |
| **Nd** | 1.02054 | 1.26601 | 1.38445 | 1.03359 | -0.0597009 | 1.14622 |
| **Yb** | -3.72899 | -11.2424 | -7.68178 | -9.61448 | 1.52445 | -4.97379 |
| **CONSTANT** | -49.2563 | -54.8751 | -42.532 | -41.127 | -40.7156 | -40.6222 |

This pane shows the functions used to classify observations. There is a function for each of the 6 main events

of “Volcanic Activity of the Phlegraean Fields”. These functions are used to predict which event of the Phlegraean Volcanic activities new observations belong to. For example, the function used for the first event is:

-49.2563 - 0.0424407*Zr - 0.28933*Nb - 0.637694*Th + 0.248593*Rb + 0.559243*Y - 0.105198*La +

1.02054*Nd - 3.72899*Yb

**Discriminant Function Coefficients for Volcanic Activity of the Phlegraean Fields**

|  | ***1*** | ***2*** | ***3*** | ***4*** | ***5*** |
| --- | --- | --- | --- | --- | --- |
| **Zr** | 0.348661 | 0.501376 | -4.13285 | 0.335731 | -1.30802 |
| **Nb** | 2.04147 | -0.647742 | 1.0852 | -0.500872 | -0.30709 |
| **Th** | -1.87032 | 0.0302289 | -0.443995 | -1.09264 | 0.748365 |
| **Rb** | -0.913987 | 1.01296 | 0.917646 | 1.28893 | -0.0971118 |
| **Y** | 0.304996 | 0.881747 | 1.93887 | -1.97401 | 0.935655 |
| **La** | 0.606471 | -2.74321 | 4.21516 | -2.34276 | -2.69696 |
| **Nd** | 0.712383 | 4.18357 | -2.72138 | 1.24082 | 0.0927708 |
| **Yb** | -0.948459 | -3.04727 | -0.846443 | 3.26228 | 2.21176 |

*Unstandardized Coefficients*

|  | ***1*** | ***2*** | ***3*** | ***4*** | ***5*** |
| --- | --- | --- | --- | --- | --- |
| **Zr** | 0.00214519 | 0.0030848 | -0.025428 | 0.00206564 | -0.00804782 |
| **Nb** | 0.0747434 | -0.0237155 | 0.0397321 | -0.0183382 | -0.0112434 |
| **Th** | -0.119185 | 0.00192632 | -0.0282934 | -0.0696282 | 0.0476893 |
| **Rb** | -0.0132559 | 0.0146914 | 0.013309 | 0.0186939 | -0.00140845 |
| **Y** | 0.0263893 | 0.0762918 | 0.167758 | -0.170799 | 0.0809561 |
| **La** | 0.0231756 | -0.104829 | 0.161078 | -0.0895262 | -0.103062 |
| **Nd** | 0.0448957 | 0.263656 | -0.171506 | 0.0781985 | 0.00584659 |
| **Yb** | -0.806983 | -2.59272 | -0.720184 | 2.77566 | 1.88184 |
| **CONSTANT** | 0.388522 | -5.33298 | -2.79889 | -3.66552 | 1.615 |

This pane shows the coefficients of the functions used to discriminate amongst the different levels of

Volcanic Activity of the Phlegraean Fields. The first standardized discriminating function is

0.348661*Zr + 2.04147*Nb - 1.87032*Th - 0.913987*Rb + 0.304996*Y + 0.606471*La + 0.712383*Nd - 0.948459*Yb
