# Supplementary material for: Vitruvian binders in Venice: First evidence of Phlegraean pozzolans in an underwater Roman construction in the Venice Lagoon
Source: PLoS One. 2024 Nov 22;19(11):e0313917. doi: 10.1371/journal.pone.0313917 (PMC11584134; doi:10.1371/journal.pone.0313917)
Supplement: Attachment — Submitted filename: PONE-D-23-36360_PDF Review.pdf [file pone.0313917.s009.pdf]

PLOS ONE

Vitruvian binders in Venice: first evidence of Phlegraean pozzolans in an underwater Roman construction in the Venice Lagoon

--Manuscript Draft--

**Manuscript Number:**	PONE-D-23-36360
**Article Type:**	Research Article
**Full Title:**	Vitruvian binders in Venice: first evidence of Phlegraean pozzolans in an underwater Roman construction in the Venice Lagoon
**Short Title:**	Phlegraean pozzolans in mortars from an underwater Roman construction in the Venice Lagoon
**Corresponding Author:**	Simone Dilaria<br>University of Padova: Universita degli Studi di Padova<br>Padova, ITALY
**Keywords:**	Phlegraean pozzolans; Roman mortars; Underwater archaeology; LA-ICP-MS; QPA-XRPD; M-S-H; Venice Lagoon
**Abstract:**	Four mortar samples were collected from a submerged Roman well-cistern in the norther part of the Lagoon of Venice, recently investigated during underwater surveys promoted by the team of maritime archaeology of the University Ca' Foscari of Venice. Samples were preliminary described following a standardized protocol of analytical techniques, including Polarized Light Optical Microscopy (PLM), Quantitative Phase Analysis - X-Ray Powder Diffraction (QPA-XRPD) and Scanning Electron Microscopy (SEM) coupled with Energy-Dispersive X-Ray Spectroscopy (EDS). Archaeometric analyses allowed the samples to be identified as lime-based mortars enriched with ceramic fragments and local sands in variable concentrations. Moreover, pyroclastic aggregates from the Phlegraean fields (pulvis puteolana) were added to the mortars to enhance the hydraulic capabilities of the compounds. Provenance determination of the volcanic pozzolans was determined geochemically by analysing via SEM-EDS and Laser Ablation-Inductively Coupled Plasma-Mass Spectrometry (LA-ICP-MS) unreacted cores of volcanic glass and by comparing the results with the geochemical profiles of pyroclastic products of the Italian Plio-Quaternary magmatic districts. This operation was complicated by the significant reaction processes the analysed pozzolans were object of, seriously affecting their geogenic chemical and petro-mineralogical features. The strongly alkaline anoxic underwater environment of the Venetian lagoon likely fostered the kinetics, as the matrices showed a relevant development of M-S-H hydrates replacing the pristine Ca-bearing phases of the binder, suggesting that the carbonation of the lime was almost null. The perfect blend of local sands, with ceramic fragments, imported volcanic rocks and brackish water promoted the development of pozzolanic and para-pozzolanic processes that influenced the durability and the perfect preservation of the analysed mortars over time to the present day. This evidence shows, once again, as much as the Vitruvian mention to the prodigious effects of pulvis puteolana used in seawater concrete (Vitr., 5.12.2-3) was thoroughly grounded in the outcomes of ancient engineering knowledge.
**Order of Authors:**	Simone Dilaria
	Giulia Ricci
	Michele Secco
	Carlo Beltrame
	Elisa Costa
	Tommaso Giovanardi
	Jacopo Bonetto
	Gilberto Artioli
**Additional Information:**	
**Question**	**Response**

Powered by Editorial Manager® and ProduXion Manager® from Aries Systems Corporation

**Financial Disclosure**<br><br>Enter a financial disclosure statement that describes the sources of funding for the work included in this submission. Review the submission guidelines for detailed requirements. View published research articles from *PLOS ONE* for specific examples.<br><br>This statement is required for submission and **will appear in the published article** if the submission is accepted. Please make sure it is accurate.<br><br>**Funded studies**<br>Enter a statement with the following details:<br>• Initials of the authors who received each award<br>• Grant numbers awarded to each author<br>• The full name of each funder<br>• URL of each funder website<br>• Did the sponsors or funders play any role in the study design, data collection and analysis, decision to publish, or preparation of the manuscript?<br><br>Did you receive funding for this work?	Yes
Please add funding details.<br>as follow-up to "**Financial Disclosure**<br><br>Enter a financial disclosure statement that describes the sources of funding for the work included in this submission. Review the submission guidelines for detailed requirements. View published research articles from *PLOS ONE* for specific examples.<br><br>This statement is required for submission and **will appear in the published article** if the submission is accepted. Please make sure it is accurate.	This project was partially implemented within the scope of the "Exceptional Laboratory Practices in Cultural Heritage: Upgrading Infrastructure and Extending Research Perspectives of the Laboratory of Archaeometry", co-financed by Greece and the European Union project under the auspices of the program "Competitiveness, Entrepreneurship and Innovation" NSRF 2014–2020 (https://ec.europa.eu/regional_policy/in-your-country/programmes/2014-2020/el/2014gr16m2op001_en). The investigation of the well-cistern of Canale San Felice in 2023 was financed by the PNRR project CHANGES - Cultural Heritage Active Innovation for Sustainable Society (project code: PE00000020; https://sites.google.com/uniroma1.it/changes/). The analyses on materials were financed from the project "Trade and use of volcanic pozzolans in the Roman world. A natural material for the production of eco-sustainable concrete of antiquity" (Principal investigator: Simone Dilaria, BIRD 2023 of the Department of Cultural Heritage of the University of Padova, project code BIRD230232/23). This work is funded by the University of Padova under the World Class Research Infrastructures (WCRI) programme - SYCURI (Synergic Strategies for Culture Heritage at Risk).

Powered by Editorial Manager® and ProduXion Manager® from Aries Systems Corporation

**Funded studies**<br>Enter a statement with the following details:<br>• Initials of the authors who received each award<br>• Grant numbers awarded to each author<br>• The full name of each funder<br>• URL of each funder website<br>• Did the sponsors or funders play any role in the study design, data collection and analysis, decision to publish, or preparation of the manuscript?<br><br>Did you receive funding for this work?"	
Please select the country of your main research funder (please select carefully as in some cases this is used in fee calculation).<br>as follow-up to "**Financial Disclosure**<br><br>Enter a financial disclosure statement that describes the sources of funding for the work included in this submission. Review the submission guidelines for detailed requirements. View published research articles from *PLOS ONE* for specific examples.<br><br>This statement is required for submission and **will appear in the published article** if the submission is accepted. Please make sure it is accurate.<br><br>**Funded studies**<br>Enter a statement with the following details:<br>• Initials of the authors who received each award<br>• Grant numbers awarded to each author<br>• The full name of each funder<br>• URL of each funder website<br>• Did the sponsors or funders play any role in the study design, data collection and analysis, decision to publish, or preparation of the manuscript?<br><br>Did you receive funding for this work?"	ITALY - IT
**Competing Interests**	The authors have declared that no competing interests exist.

Use the instructions below to enter a competing interest statement for this submission. On behalf of all authors, disclose any competing interests that could be perceived to bias this work—acknowledging all financial support and any other relevant financial or non-financial competing interests.<br><br>This statement is required for submission and **will appear in the published article** if the submission is accepted. Please make sure it is accurate and that any funding sources listed in your Funding Information later in the submission form are also declared in your Financial Disclosure statement.<br><br>View published research articles from *PLOS ONE* for specific examples.<br><br>**NO authors have competing interests**<br><br>Enter: *The authors have declared that no competing interests exist.*<br><br>**Authors with competing interests**<br><br>Enter competing interest details beginning with this statement:<br><br>*I have read the journal's policy and the authors of this manuscript have the following competing interests: [insert competing interests here]*<br><br>* typeset	
**Ethics Statement**<br><br>Enter an ethics statement for this submission. This statement is required if the study involved:<br><br>• Human participants<br>• Human specimens or tissue<br>• Vertebrate animals or cephalopods<br>• Vertebrate embryos or tissues	N/A

Powered by Editorial Manager® and ProduXion Manager® from Aries Systems Corporation

• Field research<br><br>Write "N/A" if the submission does not require an ethics statement.<br><br>General guidance is provided below. Consult the submission guidelines for detailed instructions. **Make sure that all information entered here is included in the Methods section of the manuscript.**	

**Format for specific study types**<br><br>**Human Subject Research (involving human participants and/or tissue)**<br>• Give the name of the institutional review board or ethics committee that approved the study<br>• Include the approval number and/or a statement indicating approval of this research<br>• Indicate the form of consent obtained (written/oral) or the reason that consent was not obtained (e.g. the data were analyzed anonymously)<br><br>**Animal Research (involving vertebrate animals, embryos or tissues)**<br>• Provide the name of the Institutional Animal Care and Use Committee (IACUC) or other relevant ethics board that reviewed the study protocol, and indicate whether they approved this research or granted a formal waiver of ethical approval<br>• Include an approval number if one was obtained<br>• If the study involved *non-human primates*, add *additional details* about animal welfare and steps taken to ameliorate suffering<br>• If anesthesia, euthanasia, or any kind of animal sacrifice is part of the study, include briefly which substances and/or methods were applied<br><br>**Field Research**<br><br>Include the following details if this study involves the collection of plant, animal, or other materials from a natural setting:<br>• Field permit number<br>• Name of the institution or relevant body that granted permission	
**Data Availability**<br><br>Authors are required to make all data underlying the findings described fully available, without restriction, and from the time of publication. PLOS allows rare exceptions to address legal and ethical concerns. See the PLOS Data Policy and FAQ for detailed information.	Yes - all data are fully available without restriction

A Data Availability Statement describing where the data can be found is required at submission. Your answers to this question constitute the Data Availability Statement and **will be published in the article**, if accepted.<br><br>**Important:** Stating 'data available on request from the author' is not sufficient. If your data are only available upon request, select 'No' for the first question and explain your exceptional situation in the text box.<br><br>Do the authors confirm that all data underlying the findings described in their manuscript are fully available without restriction?	
**Describe where the data may be found in full sentences. If you are copying our sample text, replace any instances of XXX with the appropriate details.**<br><br>• If the data are **held or will be held in a public repository**, include URLs, accession numbers or DOIs. If this information will only be available after acceptance, indicate this by ticking the box below. For example: *All XXX files are available from the XXX database (accession number(s) XXX, XXX.)*.<br>• If the data are all contained **within the manuscript and/or Supporting Information files**, enter the following: *All relevant data are within the manuscript and its Supporting Information files.*<br>• If neither of these applies but you are able to provide **details of access elsewhere**, with or without limitations, please do so. For example:<br><br>*Data cannot be shared publicly because of [XXX]. Data are available from the XXX Institutional Data Access / Ethics Committee (contact via XXX) for researchers who meet the criteria for access to confidential data.*<br><br>*The data underlying the results presented in the study are available from (include the name of the third party*	All relevant data are within the manuscript and its Supporting Information files.

and contact information or URL).<br>• This text is appropriate if the data are owned by a third party and authors do not have permission to share the data.<br><br>* typeset	
Additional data availability information:	Tick here if the URLs/accession numbers/DOIs will be available only after acceptance of the manuscript for publication so that we can ensure their inclusion before publication.

Powered by Editorial Manager® and ProduXion Manager® from Aries Systems Corporation

Manuscript

Click here to access/download;Manuscript;Vitruvian binders in Venice_Def.docx

Vitruvian binders in Venice: first evidence of Phlegraean pozzolans in an underwater Roman construction in the Venice Lagoon

Simone Dilaria[1,2,*], Giulia Ricci[2,3], Michele Secco[1,2], Carlo Beltrame[4], Elisa Costa[4], Tommaso Giovanardi[5], Jacopo Bonetto[1], Gilberto Artioli[2,3]

[1] Department of Cultural Heritage (DBC), University of Padova, Piazza Capitaniato 7, 35139 Padua, Italy.

[2] Inter-Departmental Research Centre for the Study of Cement Materials and Hydraulic Binders (CIRCe), University of Padova, Via Giovanni Gradenigo 6, 35131 Padua, Italy.

[3] Department of Geosciences, University of Padova, Via Gradenigo 6, 35131 Padua, Italy.

[4] Department of Humanities, Ca' Foscari University Venice, Dorsoduro 3484/D, 30123 Venice, Italy

[5] Department of Chemical and Geological Sciences, University of Modena and Reggio Emilia, Via Campi 103, 411251 Modena, Italy

[*] Corresponding author (simone.dilaria@unipd.it)

Abstract

Four mortar samples were collected from a submerged Roman well-cistern in the norther part of the Lagoon of Venice, recently investigated during underwater surveys promoted by the team of maritime archaeology of the University Ca' Foscari of Venice. Samples were preliminary described following a standardized protocol of analytical techniques, including Polarized Light Optical Microscopy (PLM), Quantitative Phase Analysis - X-Ray Powder Diffraction (QPA-XRPD) and Scanning Electron Microscopy (SEM) coupled with Energy-Dispersive X-Ray Spectroscopy (EDS). Archaeometric analyses allowed the samples to be identified as lime-based mortars enriched with ceramic fragments and local sands in variable concentrations. Moreover, pyroclastic aggregates from the Phlegraean fields (*pulvis puteolana*) were added to the mortars to enhance the hydraulic capabilities of the compounds. Provenance determination of the volcanic pozzolans was determined geochemically by analysing via SEM-EDS and Laser Ablation-Inductively Coupled Plasma-Mass Spectrometry (LA-ICP-MS) unreacted cores of volcanic glass and by comparing the results with the geochemical profiles of pyroclastic products of the Italian Plio-Quaternary magmatic districts. This operation was complicated by the significant reaction processes the analysed pozzolans were object of, seriously affecting their geogenic chemical and petro-

mineralogical features. The strongly alkaline anoxic underwater environment of the Venetian lagoon likely fostered the kinetics, as the matrices showed a relevant development of M-S-H hydrates replacing the pristine Ca-bearing phases of the binder, suggesting that the carbonation of the lime was almost null. The perfect blend of local sands, with ceramic fragments, imported volcanic rocks and brackish water promoted the development of pozzolanic and para-pozzolanic processes that influenced the durability and the perfect preservation of the analysed mortars over time to the present day. This evidence shows, once again, as much as the Vitruvian mention to the prodigious effects of *pulvis puteolana* used in seawater concrete (Vitr., 5.12.2-3) was thoroughly grounded in the outcomes of ancient engineering knowledge.

1. Introduction

The technological expertise achieved by humankind in the formulation of binding products from the Prehistory throughout time represents one of the most intriguing aspects in contemporary research on ancient mortar-based materials [1–4]. A specific interest was devoted by scholars to the parametrization of the hydraulic capabilities of ancient recipes, in order to unveil the secrets of the extreme durability and longevity of such composite construction materials.

In this framework, the use of certain additives, such as crushed ceramics, highly-amorphous volcanic rocks, organic ashes, metal slags or calcined clays [5] to improve the hydraulic and cohesive behaviour of aerial lime-based mortars [6–11] represents one of the most brilliant achievements of the ancient craftsmanship. Under the term “pozzolanic reaction” we usually refer to the full set of chemical processes occurring when these materials are mixed with calcium hydroxide (lime) in liquid solutions. During the reaction, the silica and alumina compounds present in these pozzolanic aggregates react with the calcium hydroxide of the lime pastes. This results in the formation of anthropogenic phases ordinarily detected in ancient hydraulic mortars [12], having very variable stoichiometries, ranging from pure calcium silicate hydrates (C–S–H) to calcium aluminate hydrates (C-A-H) or complex C–A–S–H phases at increased Al activity. Such newformed phases are the main binding agents responsible for the strength and durability of the resulting composite materials [13–18].

Nevertheless, the environmental conditions in which the compounds are embedded also play an essential role in the development of reaction processes, as the latent reactivity of pozzolanic aggregates appears to be favored in saltwater environments, since the alkaline and anoxic conditions promote the reaction, whereas lime carbonation is minimized.

Less thoroughly investigated in literatures is the fact that, under certain conditions of alkali, magnesium, and chlorine activity, the hydraulic reaction can deviate from the typical lime-silica-alumina ternary diagram to other types of para-pozzolanic hydrate products, based on the interaction of magnesium with silica or, sometimes, also with alumina. These processes usually develop into M-S-H/M-A-S-H phases (where M stands for MgO in cement chemistry notation), having a variable phyllosilicate to quasi gel-like structure [19–22], whose formation in ancient mortars is being properly recognized and parameterized only in the last years' research [12,23–28].

The full set of reactions occurring in mortar-based materials enriched with pozzolanic aggregates in submerged environments were empirically known by ancient builders. Both Vitruvius (Vitr. 2.6.1-2; 5.12.2) and Pliny (Pliny 16.202; 35.166) exalted the outstanding properties of the *pulvis puteolana*, referred to a prodigious powder outcropping around the modern town of Pozzuoli (Bay of Naples) that in geological terms relates to the mainly unlithified pyroclastic ash deposits (namely pumices and feebly lithified tuffs) of the Neapolitan volcanic district, comprising both the last Phlegraean (Neapolitan Yellow Tuff and post-Neapolitan Yellow Tuff events) and Mt. Somma-Vesuvius pre-79 CE eruptive events [29]. These Latin authors indicated the use of *pulvis puteolana* for enhancing the longevity of seawater concrete [30,31]. In perfect agreement with the literary source, from the Augustan Age (beginning of the 1st c. CE) onwards, this product obtained the monopoly in the markets as the topmost material for the manufacture of long-lasting hydraulic mortar-based materials for underwater port infrastructures [28,32–36].

In this research, new evidence of mortars enriched with Neapolitan volcanic pozzolans in submerged environments is reported. Samples under study were collected from a Roman well-cistern nowadays preserved in the Norther Lagoon of Venice, recently investigated by the team of maritime Archaeology of the University Ca' Foscari Venice, led by C. Beltrame [37]. The samples were preliminary analysed adopting a multi-analytical protocol for the characterization of the main petro-mineralogical features of

the compounds, including Polarized Optical Microscopy (PLM), Quantitative Phase Analysis - X-Ray Powder Diffraction (QPA-XRPD) and Scanning Electron Microscopy (SEM) coupled with Energy Dispersive Spectroscopy (EDS). Indeed, archaeometric analyses allowed the compounds to be identified as lime-based mortars enriched with ceramic fragments (*cocciopesto*), local sand and volcanic pozzolans in variable concentrations. Great interest concerned the investigation of the extraordinarily advanced reaction phenomena that significantly perturbed the original geochemical and petro-mineralogical characteristics of the pozzolanic aggregates. High aliquots of paracrystalline Mg-rich hydrated phases (M-S-H/M-A-S-H), likely related to para-pozzolanic reactions between the lime-based binder and Mg-rich reactive aggregates [38] through a liquid medium [39], were detected. However, these complex reaction kinetics seriously complicated the determination of the provenance of the volcanic pozzolans. Their compatibility with the composition of the pyroclastic products of the Phlegraean Fields was geochemically established by analysing via SEM-EDS and Laser Ablation-Inductively Coupled Plasma-Mass Spectrometry (LA-ICP-MS) unreacted glassy cores of a selection of pozzolanic aggregates and by comparing the results with over 950 geochemical profiles of pyroclastic rocks of the Neapolitan volcanic districts selected from literature.

Our outcomes represent the first proof of Phlegraean pozzolans in the Veneto region during the Roman era, which, not coincidentally, has been reported in an extremely fragile geomorphological context, still barely archaeologically explored, such as the Lagoon of Venice.

2. The archaeological context and mortar sampling

The so-called 'tower' situated along the San Felice Channel (**Fig[illegible] 1**), in the northern lagoon of Venice, lies at a depth ranging from -4.5 to -3.0 m MSL, rising around 1.5 m above the channel bottom. Based on the photogrammetric survey and the quoted DEM of the structure (Digital Elevation Model), the depth of the structure suggests that it was partially placed underwater also in Roman times. In fact, even considering that the Roman relative sea level (RSL) in this part of the northern lagoon was about 2.15 m lower [40], the base of the well-cistern was surely positioned at a depth of around – 2.0 m MSL [37].

The geomorphological reconstruction of the coast of the northern lagoon, proposed in **Figure 1**, shows that, compared with nowadays, the Torrione San Felice (TSF), located by side of the S. Felice Channel in antiquity, was much closer to the edge of the lagoon and with the Adriatic Sea.

The historical framework and constructive technique of the TSF structure was already accurately described in [37]. This is a flat box-like construction measuring 6.7 × 7.7 m, entirely made of local bricks, both used in masonries and floors, measuring around 43.5–46 × 29.5–29 × 6 cm, which is the typical size of the *sesquipedali* of the Po Valley [41,42]. Due to its shape, in previous studies (for example [43]) the structure was interpreted as a 'defensive tower' dating to the Roman Imperial period. In the summer 2020, after the underwater surveys conducted by the team of Ca' Foscari University assisted by Venice's Idra company, the structure was reinterpreted as a Roman well-cistern, a peculiar system for collecting and storing freshwater, an ancestor of the Venetian-style well ("Pozzo alla Veneziana") developed in Venice during the Medieval and Modern Ages [44], but originating in Roman times with various pieces of evidence from the North-Eastern Adriatic territory (Aquileia, Musile di Piave and Ca' Ballarin/Treporti, **S1 Figure**). This new interpretation relies on several relevant elements, including a preliminary identification of the use of waterproofing *cocciopesto* mortar renders, the assessment of the structure's base depth in relation to the relative sea level, the absence of groundwork poles in the soft lagoon mud that are recurrent for building foundations of Roman period and the strong similarities with the much better preserved well-cistern of Ca' Ballarin, located along the same channel not far from this site. The dating of the TSF structure in the 1st c. CE, or little later, was determined on the basis of associated pottery finds [37].

In order to characterize the properties and composition of the peculiar hydraulic mortars employed in the structures, four mortar samples were collected from coating renders and masonry joints of the structure during two underwater campaigns in 2020 and 2023 (**Figure 2).** All necessary permits were obtained for the described study, which complied with all relevant regulations. The sampling activity and the underwater archaeological survey in the Lio Piccolo site was authorized by the Soprintendenza Archeologia, Belle Arti e Paesaggio per l'area metropolitana di Venezia e le province di Belluno, Padova e Treviso. Samples were collected and stored in the laboratories of the Department of Geosciences of the University of Padova.

Figure 1. Geomorphological map of the northern lagoon of Venice (modified from [37]) with indication of the site of the Torrione San Felice well-cistern. In the inset map, the site is georeferenced on a satellite sketch-map taken from Google Earth.

Figure 2. Orthophoto of the submerged well-cistern at the end of 2020 survey, with indication of the mortars under study and their sampling points.

3. Multi-analytical protocol

Samples collected from the TSF structure were analysed according to a multi-analytical protocol designed for defining the raw materials employed in the compounds, the peculiar reaction phenomena occurring in submerged environment and, finally, the provenance of the volcanic pozzolans included in the mortars (**S1 Analytical methods**).

All samples were firstly subjected to a full petro-mineralogical characterization, through detailed Polarized Light Optical Microscopy (PLM) investigations of mortars prepared on 30 μm thin sections. The characterization was accomplished in accordance with the standard UNI 11176:2006 "Cultural heritage - Petrographic description of a mortar" [45]. Furthermore, samples were mineralogically investigated by Quantitative Phase Analysis - X-Ray Powder Diffraction (QPA-XRPD). Samples were analysed after the addition to the bulk fraction of 20 wt% zincite as internal standard. Subsequently, the XRPD profiles were refined through the Rietveld method [46].

After this first screening, the most representative sample (TSF_T9) was subjected to further in-detail investigations aimed at parametrizing the reaction phenomena and the provenance of the natural pozzolans included in the compound.

Scanning Electron Microscopy (SEM) coupled with Energy-Dispersive X-Ray Spectroscopy (EDS) analyses of the binder matrix and aggregate's reaction rims of the sample were functional at determining the chemism of the secondary hydrate products occurring in the peculiar alkali-rich marsh-water environment of the Lagoon of Venice. Moreover, QPA-XRPD analysis on the sole binder fraction of the sample allowed the reaction products of the material to be characterized also at a mineralogical level. Binder-concentrated aliquots were separated from the bulk mortar following the wet separation procedure [47,48]. The quantification of the mineralogical phases was determined by the addition to the

separated-binder fraction of 20 wt% zincite as internal standard. Then, the profiles were processed adopting the same procedure previously defined for the bulk mortars.

The provenance of the volcanic pozzolans was determined adopting a geochemical approach. After a detailed mineral-petrographic characterization of the pozzolanic specimens under PLM, the investigation of the geochemistry of pyroclastic rocks was performed according to the analytical protocol reported in [49]. Firstly, their major elements' profiles were determined by analysing unreacted areas of volcanic glass. The average values and standard deviations were calculated on the basis of 5 up to 10 SEM-EDS semi-quantitative punctual analyses, performed on a 30 μm thin section (TSF_T9A) and on two 1 mm thick sections (TSF_T9B and TSF_T9C), prepared in order to be investigated via LA-ICP-MS analysis. The sections were polished and carbon-coated before the analysis. LA-ICP-MS analyses were performed in order to collect the trace element profiles of a selection of volcanic pozzolans already investigated by SEM-EDS. In order to avoid, as much as possible, the contamination with non-geogenic chemical elements (i.e. pozzolanic products), a set of clasts preserving portions of sufficiently unaltered volcanic glass to fit within the 55 μm laser ablation spot-size were selected for this part of the research. For each clast, 4 up to 9 punctual LA-ICP-MS analyses were acquired, and the average values and standard deviations calculated and reported.

The actual provenance of the clasts was determined by comparing on discriminant scatterplots a selection of major and trace elements collected from the observed pozzolans with the distribution of already-analysed geological samples relating to the main Plio-Quaternary magmatic activities of the Italian peninsula, according to an already scientifically-established methodology [29,36,50,51]. Moreover, by plotting in explorative scatterplots and spider-diagrams the chemical elements one-by-one, an updated pattern of elements was selected among REE and HFSE to discriminate among the differentiated magmatic activities of the Neapolitan volcanoes. In addition, in order to check and verify the results inferred from scatterplot and spider-diagram distributions, we processed the geochemical data by discriminant analysis (DA). This is a multivariate statistical data reduction method frequently adopted to validate the classification of known data into categories or to assign unknown observations into such categories. This useful technique has been already applied in archaeology for assessing the

provenance of artifacts of unknown origin by matching their descriptive variables with already classified markers [52–56].

4. Results and Discussion

4.1 Characterization of raw materials

Through PLM investigations, the TSF samples were characterized as lime-based mortars enriched with ceramic fragments and dust (*cocciopesto*), mixed with local sands and volcanic pozzolans in variable concentrations (**Tab. 1**). Sample TSF_T9, collected from the joint coating between one wall and the floor of the well-cistern (socle), has the highest content of volcanic pozzolans and ceramic fragments, easily detectable even at naked eye (**Figure 3, a**). This is followed by sample TSF_LN, where the concentration of pozzolanic aggregates appears less abundant than the previous one (**Figure 3, b**). Finally, the last two samples, TSF_SS and SN, show few traces of pozzolanic aggregates and a higher concentration of alluvial sand and other non-pozzolanic elements (**Figure 3, c-d**), mainly comprising angular monocrystalline quartz, micritic limestone and dolostones clasts, small granitoids and scattered plagioclases and micas of the muscovite type.

QPA-XRPD analyses highlighted these differences through a significant shift in the concentration of the phases correlated with the silicate fraction of the sands, such as quartz and plagioclases (**Figure 4** and **Tab. 1**). Another distinguishing marker is represented by the abundance of diopside, that is almost twice in TSF_T9 (11.4 wt%) in respect to the other samples. Its occurrence is related to the ceramic inclusions and it demonstrates the firing of Ca-rich clays at high temperature (>850 °C) [57].

Among the newformed pozzolanic phases, hydrated calcic ferro-aluminates of the C-A-H type (AFm phases) [58] were detected in all the samples apart from TSF_T9. Its absence in the most hydraulic-suspected sample of the dataset is intriguing and probably rely on the fact that most of Si and Al ions did not react with Ca in a traditional Ca-based pozzolanic system, but with Mg, precipitating into Mg-rich hydrated compounds (M-S-H and M-A-S-H), as detailed in paragraph 4.2. In fact, broad low-angle peaks ascribable to poorly crystalline M-S-H/M-A-S-H phases presenting the peculiar phyllosilicate-like structure of turbostratically-disordered smectite clays [12,19,26,38,59] were detected in high

concentration in the sample TSF_T9. Finally, the presence of pyrite, detected by XRPD in most of the samples, is to be ascribed to post-depositional alteration phenomena of the materials, as a consequence of the action of sulfate-reducing bacteria, which promoted the dissolution of oxides and hydroxides in sulfur-rich alkaline aqueous solutions. The conditions for the proliferation of these bacteria are coherent with that of buried contexts of anoxic lagoon-like environments, having abundant organic matter and poor water circulation [60–62].

~~As already stated,~~ sample TSF_T9 was selected for a thorough analytical characterization, being the mortar with the highest concentration of pozzolanic aggregates and that showing the more complex reaction kinetics of the pozzolanic aggregates with the binder.

Under PLM observation, the binder of TSF_T9 appears extremely zoned with a structure varying from micritic to microsparitic, with diffused areas exhibiting very low birefringence colours (**Figure 5, b**), likely ascribable to the relevant precipitation of hydrate phases.

Lime lumps are few and scattered (**Figure 5, c**), demonstrating a good mixing of the binding component. The aggregate is mainly constituted of poorly sorted ceramic elements (**Figure 5, d**), with a grain-size distribution ranging from angular pluri-millimetric fragments to finely dispersed micrometric powder, less than 40 μm in size. The relevant milling probably contributed to the reactivity of the ceramic fraction [63,64].

Volcanic rock clasts constitute a slightly subordinate fraction of the aggregate in respect to the ceramic ones. They primarily consist in rounded-subrounded vesicular pumices with elongated or round pores and weak to compact density (**Figure 5, e**). Under specific conditions, the reaction with the binder is pervasive to the extent that pozzolans become barely discernible, appearing as *ghosts* fully reacted within the matrix. Two families of pumices were distinguished, based on the texture of their groundmass: completely aphyric (glassy) juveniles (**Figure 5, f-g**) fairly prevailing over feeble porphyritic ones (**Figure 5, h-i**), presenting sparse feldspars microlites and biotite occasionally filling the intraclasts. The phenocryst assemblage is constituted by large minerals of K-feldspars (sanidine) prevailing over plagioclases, biotite and – occasionally – clinopyroxenes. Accessory minerals are composed of opaque minerals (iron oxides) and apatite (**S2 Figure, a**). These juveniles are characterized by high porosity, deriving from the quick outgassing of magmatic volatiles in the course of explosive

events [65]. Angular chips of ignimbrite tuffs (**Figure 5, j**) and eutaxitic ignimbrites (**Figure 5, k**) constitute a totally subordinate fraction of the volcanic aggregates. They are rich in mineral inclusions of biotite, k-feldspars (sanidine/orthoclase) and plagioclases, clay minerals, iron oxides, opaque minerals, glass shards and fragmented pumices embedded in a cineritic matrix (**S2 Figure, b**). Finally, elements of compact vitrophyric obsidians with scattered feldspars microlites (**Figure 5, l**) were very occasionally observed together with dense porphyritic lavas with k-feldspars and clinopyroxene phenocrysts (**Figure 5, m**). It must be remarked that in the other samples from the well-cistern, only pumices were detected among the volcanic pozzolan components.

From a minero-petrographic point of view, the volcanic pozzolans detected in TSF samples appear incompatible with the local volcanic rocks of the Veneto Region, substantially constituted by the dense effusive lava domes, dikes and explosive breccias of the Euganean Hills volcanic units [66]. Therefore, this component of the aggregate is not local, and it was imported from other regions, whose geographic position was tracked in paragraph 4.3.

Opaque millimetric shards and ground microclasts of pure-Fe slags (**Figure 5, n**), chemically characterized via SEM-EDS microanalysis (**S2 Figure, c**), were detected uniquely in TSF_T9 sample, while they are absent in the others. They could act as tensile strengthening agents of the binding composite, and they were likely added only to this sample to enhance the mechanical behaviour of the compound [67], according to a practice already attested in ancient Greek and Roman times [68,69]. Their abundance suggests an intentional reuse of discarded processing debris of metalwork that probably took place in the nearness of the site, but on which there are no pieces of evidence in the archaeological record.

Finally, fragments of carbonate rocks were identified and they consist in sub-millimetric to pluri-millimetric angular or sub-rounded chips of crystalline limestones (**Figure 5, o**), metamorphosed white marbles (**Figure 5, p**), and occasional altered dolostones/dolomitic limestone (**Figure 5, q**), indicating de-dolomitization processes [70]. Furthermore, apart from monomineralic quartz/carbonate sandy clasts, scattered monomineralic feldspars, clinopyroxenes, biotite and opaque minerals, probably deriving from the weathering of volcanic aggregates, were detected.

Among the additives, dispersed charcoal (**Figure 5, r**) and organic unburned fibres (**Figure 5, s**) were occasionally detected, both in samples TSF_T9 as well as in the other ones.

Figure 3. Low-magnification PLM micrographs of TSF samples (transmitted light, plane polars). (**a**) TSF_T9; (**b**) TSF_LN; (**c**) TSF_SN; (**d**) TSF_SS. Legend: Vp = Volcanic pozzolan; Cf = Ceramic fragments.

Figure 4. XRPD patterns of the four analysed TSF mortar samples. Mineral phases labelled according to [71] when mentioned: Clc = clinochlore; Bt = Biotite; Ms = Muscovite; AFm = AFm phases; Php = Phillipsite; Anl = Analcime; Qz = Quartz; Kfs = K-Feldspar; Pl = Plagioclase; Cal = Calcite; Cpx = Clinopyroxenes; Hem = Hematite; Ilm= Ilmenite; Py = Pyrite; Znc = Zincite (used as internal standard).

Figure 5. In-detail mineral-petrographic characterization of TSF_T9. (a) Cross section of the sample, with identification of the main aggregate components; PLM images of TSF_T9 sample, acquired in plane nicols (PN) and crossed nicols (XN); (b) low-birefringence lime binder in an area rich in poorly sorted ceramic fragments (XN); (c) a lime lump surrounded by an outer rim showing low birefringence (PN); (d) the ceramic fragments' assemblage, mainly constituted by poorly sorted grains (PN); (e) feebly sorted sub-rounded clasts of pumice, with evident reaction edges (PN); (f) and (g) a clast of aphyric pumice, with scattered phenocrysts of k-feldspars (sanidine) and plagioclase (XN and PN respectively); (h) and (i) a clast of pumice with a feebly-porphyritic texture, containing scattered feldspar microlites in a primarily glassy groundmass (XN and PN respectively); (j) an angular chip of tuff with cineritic groundmass (PN); (k) a coarse fragment of eutaxitic ignimbrite including volcanic shards, fragmented pumices and scattered biotite and feldspars (PN); (l) a small angular fragment of very dense vitrophyric obsidian, with sharp reaction rims (XN); (m) a clast of dense porphyritic lava with k-feldspars and clinopyroxenes phenocrysts (XN); (n) concentration of opaque fine-grained iron slags (PN); (o) an angular chip of crystalline limestone (PN); (p) a coarse clast of marble, composed of anhedral metamorphized crystals of calcite (PN); (q) a chip of dolostone with clear de-dolomitization edges (PN); (r) an organic charcoal inclusion (PN); (s) a small fragment of unburned organic fibre (PN). Legend – rock clasts and main aggregates: P = pumice; aP = aphyric pumice; pP = porphyritic pumice; Lv = volcanic lava; Obs = obsidian; Tu = tuffs; Ig = ignimbrite; Sh = volcanic shard; Mb = marble; Dol = dolostone; Cc = crystalline limestone; Cf = ceramic fragments; Lime binder and reactions: L = lime lump; Rr = reaction rim; Minerals: Bt = biotite; Fls = feldspar; Kfs = k-feldspar; Pl = plagioclase; Qz = quartz; Cpx = clinopyroxene; Op = opaque minerals; Other inclusions: Ch = charcoal; Of = organic fibre; Is = iron slag.

Table 1. Cumulative results of PLM and bulk QPA-XRPD analyses on the four mortar samples from Torrione San Felice well-cistern. Legend: b.d. = below detection; **- -** very low presence (<2%); **-** low presence (2-7.5%); **+** =

moderate occurrence (7.5-20%); **++** = relevant occurrence (20-40%); **+++** = abundant occurrence (> 40%); LB = Lime binder.

**Sample**	**TSF_T9**	**TSF_LN**	**TSF_SN**	**TSF_SS**
**Function**	Coating (socle)	Joint mortar	Linings (?)	Linings (?)
**Description**	junction between masonry and floor	between two *sesquipedali* of the masonry	Flooring	Flooring
PLM				
**Binder texture**	LB	LB	LB	LB
**Lime Lumps**	-	+	-	-
**Voids**	-	+	++	+
**Local sands**	-	++	+++	+++
**Ceramic fragments**	+++	++	+	-
**Volcanic pozzolans**	++	+	-	-
**Cr. Limestones chips**	+	+	-	-
**Iron slags**	+	b.d.	b.d.	b.d.
**Shells**	- -	-	- -	- -
**Fibers and charchoal**	- -	- -	-	- -
**estimated B:A prop.**	1:2	1:2	1:3	1:2
QPA-XRPD				
**Calcite**	19.5	19.2	11.7	26.1
**AFm**	b.d.	b.d.	0.3	1.3
**Dolomite**	b.d.	3.5	2.5	4.96
**Quartz**	2.4	21.7	19.4	25.8
**Plagioclase**	3.9	10.4	8.5	7.2
**Sanidine**	4.2	4.7	4.1	3.5
**Microline**	4.0	3.6	2.1	2.1
**Analcime**	0.9	0.4	0.4	0.2
**Phillipsite**	0.1	0.5	b.d.	0.6

**Clinopyroxene**	11.4	5.4	6.2	4.4
**Hematite**	0.5	0.3	0.3	0.3
**Ilmenite**	0.2	0.1	0.2	0.2
**Muscovite**	b.d.	2.3	2.3	3.0
**Biotite**	0.7	1.4	b.d.	0.7
**Clinochlore**	b.d.	3.7	2.2	4.1
**Pyrite**	b.d.	0.6	0.2	0.1
**Smectite/M-S-H**	26.0	b.d.	13.6	b.d.
**Amorphous**	26.1	22.4	26.1	15.6

4.2 Binder Characterization and Reaction in Submerged Environment

The reaction processes involving pozzolanic aggregates and the lime matrix in TSF_T9 were investigated in-detail by SEM-EDS and QPA-XRPD analyses of the separated binder fraction.

The backscattered electron images, as presented in **Figure 6**, reveal that most of the pozzolanic aggregates are profoundly reacted with the binder. The chemical composition of the binder matrix clearly indicates the presence of Si, Mg, Al, and, subordinately, Ca and Fe (**Figure 6, a- c3**), which are primarily attributed to a complex array of hydraulic phases that ultimately formed after pozzolanic reaction. In general terms, the factors triggering the widespread reactivity of these materials derive from a complex interplay of parameters that are influenced by the intrinsic physicochemical properties of the pozzolanic aggregates.

QPA-XRPD analyses of the binder fraction showed the occurrence of very high aliquots of paracrystalline Mg-rich hydrated phases (M-S-H/M-A-S-H) as principal crystalline phase, up to 37.0 %wt (**Figure 6, g**). These were mineralogically described using the structure of a turbostratically disordered smectite, as described at paragraph 4.1. Also, the relevant amorphous phase (up to 52.5 %wt), displaying no diffraction reflexes, may be mainly related to development of these hydrate phases. It has been already noticed that salt-water plays a crucial role in facilitating the formation of M-S-H/M-A-S-H products. This is achieved by increasing the pH, which, in turn, promotes the dissolution of silicates and carbonates releasing Mg, Si, Al, ions in the system, which then combine to form Si-Al-Mg hydraulic phases

[23,25,26,28,72]. As a matter of fact, the significant formation of M–S–H/M–A–S–H phyllosilicate gels is likely occurring when high concentrations of active magnesium, silica and alumina are present in the system. Whereas Al and Si were provided by the natural and artificial pozzolans, the sources of Mg in TSF_T9 within the aggregate fraction are less consistent and rely on the de-dolomitized dolostones and on certain Mg-rich ceramic fragments, exhibiting partially vitrified and reacted siliceous-aluminous matrices (**Figure 6, d-d1)**. However, the amount of active Mg occurring within the aggregate fraction is not sufficient to satisfy the abundant magnesium content detected by EDS analysis of matrices. The possible utilization of a magnesian binder must be rejected as confirmed by EDS analyses of the scattered lumps and calcination relicts, proving its original chemistry as eminently calcic (**Figure 6, e-e1, f-f1**).

Therefore, as already suggested in [39], it must be assumed that another relevant source of Mg was provided by the prolonged exposure to brackish water, having a significant concentration of magnesium ions (up to 3.7 %wt) in the overall amount of soluble salts [73].

Regarding the remaining phases, the QPA-XRPD shows a very low amount of calcite (1.6 %wt), to be ascribed to the anthropogenic $CaCO_3$ included in the binder. This demonstrates that carbonatation of the binder was minimized whereas the available portlandite primarily undergone pozzolanic interchanges, being the reactivity of the pozzolanic aggregates fostered in the alkaline and oxygen-depleted subaqueous conditions. Moreover, the sample appears clearly Ca-depleted. In fact, the low-Ca rates returned by EDS analyses in the matrixes prove that the development of ordinary Ca-based hydraulic phases (C-S-H; C-A-S-H) was completely subordinated to Mg-based ones in such chemical environment. Therefore, under this condition, the hydraulic reaction occurred in TSF_T9 sample deviated from the conventional calcium-silica-alumina ternary diagram due to specific conditions of alkali, magnesium and chlorine activity.

The remaining phases detected with the QPA-XRPD analysis of the TSF_T9 binder fraction are negligible and mainly consist in mineral residues from the aggregate, as goethite (2.3 %wt), representing fine liquid-suspended particles deriving from the disaggregation of the iron shards included in the sample.

Figure 6. Backscattered electron images of the sample TSF_T9. (a) SEM acquisition of an area of sample TSF_T9; (b) enlarged picture of the area highlighted by the white dashed line in figure (a); (b1) EDS analysis of an unreacted core of a pumice clast; (b2) Binder matrix of an area close to the pumice clast enriched in M-A-S-H; (c) pumice aggregate within the matrix of the sample; (c1) unreacted pumice clast; (c2 and c3) binder matrix enriched in M-A-S-H; (d) ceramic fragment; (d1) EDS analysis of the ceramic fragment; (e) lime lump; (e1) EDS analyses of the lime lump; (f) calcination relict; (f1) EDS analyses of the calcination relict; (g) XRPD pattern of the separated binder fraction of sample TSF_T9. Mineral phases labelled according to [71] when mentioned: Ms = Muscovite; AFm = AFM phases; Cal = Calcite; Cpx = Clinopyroxenes; Gth = Goethite. The quantification of mineral phases after QPA analysis is shown to the right of the XRPD profile.

4.3 Provenance of volcanic pozzolans

Preliminary minero-petrographic and microchemical investigation of the volcanic pozzolans included in TSF samples allowed to establish their incompatibility with the geology of the Veneto region.

In order to determine the provenance of these extra-regional pozzolans on a geochemical basis, 29 volcanic clasts, comprising 23 aphyric and slightly porphyritic pumices, 4 ignimbrite-tuffs, 1 vitrophiric obsidian and 1 eutaxitic ignimbrite (**S1 Table**), were preliminary investigated via EDS microanalysis on unreacted glassy/cineritic areas, still preserving the geogenic chemical fingerprint. This technique has been already adopted as a rapid and efficient tool for a preliminary assessment of volcanic pozzolans' geochemistry included in ancient mortar-based materials [50,54,74,75].

For a first comparison between the analysed clasts and the geochemical intervals of the volcanic products of the main Italian Plio-Quaternary magmatic districts [76,77], the resulting major elements' profiles were plotted in the TAS (Total Alkali vs Silica) diagram [78], reporting the relation between alkaline elements (Na_2O + K_2O) and silica (SiO_2). In this diagram, most of the TSF_T9 volcanic pozzolans can be chemically categorized as phonolites. However, some clasts (*a, c, z, 05*), primarily comprising ignimbrites and ignimbrite tuffs, exhibit compositions that fall between phonolites and trachytes. Specifically, the Si values in the pumice clasts range from 55.1 to 60.8 wt% with a standard deviation (st.dev.) typically less than 1 wt% in aphyric samples and ≤ 1.5 in porphyritic ones. The concentration of Na + K in the pumice fragments varies around 14 ± 0.5 wt%, except for clast *c* (12.3 wt%), confirming their uniform geochemical composition.

On the other hand, st.dev. values exceeding 1.5 in Si concentration and 1 in Na + K concentrations were registered for tuffs and ignimbrite clasts. This could be symptomatic of a higher heterogeneity, possibly determined by the occurrence of microcrystalline minerals in the cineritic matrices of these stone chips, whose presence can be inferred also by the inhomogeneous interference colours observed in the SEM backscattered images (see **S2 Figure, b**).

The geochemical profile of the clasts is compatible with most of the volcanic products of the Campanian magmatic province, including the alkaline and highly-alkaline series of the main Phlegraean eruptions (pyroclastic products) [76,79] comprising the pre- and Campanian Ignimbrite (pre-CI/CI), pre- and Neapolitan Yellow Tuff (pre-NYT/NYT), post-NYT as well as the Phlegraean-correlated volcanoes of Ischia and Procida-Vivara islands (**Figure 7, a**). However, some of the clasts, characterized by low-Si concentrations, fall outside the intervals of the Phlegraean Fields. The Si-depletion could be attributable to a slight alteration of the clasts after pozzolanic/para-pozzolanic reactions that may have affected even the innermost cores of the less-reacted clasts.

On the basis of the TAS, all the observed profiles except clast 09 appear geochemically compatible with the Somma-Vesuvius explosive events older than 79 CE (**Figure 7, b**). On the other hand, they are incompatible with the highly-alkaline scoriaceous ~~cinerites~~ of the Colli Albani (*harenae fossiciae*) and with most of the other products of the Roman and Tuscan magmatic provinces [76,80,81], as well as with the rhyolites and rhyolitic breccias of the Oligocene eruptions of the Euganean Hills in the Veneto region [82] (**Figure 7, c**). Finally, a ~~very feeble~~ overlap can be tracked with certain pyroclastic phonolites of Vulcano in the Aeolian Arc Isles [83] (**Figure 7, d**).

These results demonstrate that TAS is non-conclusive for provenance determination of the volcanic pozzolans. Therefore, trace elements, collected via LA-ICP-MS, were crucial to exactly pinpoint their origin. Glassy/cineritic areas of 9 pumices and 2 tuffs from sub-samples TSF_T9B and TSF_T9C (**S2 Table**), presenting unreacted areas wide enough to be comprised within the 55-µm laser ablation's spot size, were selected and analysed avoiding as much as possible the altered portions (**Figure 8**).

Good results were collected for most of the analysed pumices (both aphyric and porphyritic). In these clasts, the quantification of REE and HFSE returned a low st.dev. (see the comments to the data reported in the **S2 Table**), that, in particular, is coherent for the concentrations of Zr, Y, Nb, Yb, La, Th and Ta,

usually considered in literature for provenance discrimination [29,49–51,55,84–86]. Conversely, the two analysed tuff samples (clasts *a*, and *s*) exhibited a significantly high st.dev. and unusual average values, confirming the geochemical inconsistency already evidenced after the EDS investigations. Therefore, while the LA-ICP-MS technique proved effective for measuring the geochemistry of pure volcanic glass, it is not suitable for analysing such heterogenous volcanic materials. Thus, these clasts were removed from the dataset considered in the following analytical steps.

As suggested by the TAS, the discordancy with the volcanic products of Roman district (comprising Colli Albani's *harenae fossiciae*) and Aeolian Isles, is confirmed also by trace elements, as demonstrated by Th/Ta vs Nb/Zr and Zr/Y vs Nb/Y scatterplots [50,87]. In the first one the analysed clasts plot within the field of the Neapolitan volcanoes (**S3. Figure, a**), characterized by the low ratio of Th/Ta (usually < 15, except from Pomici di Mercato eruption of Mt Somma-Vesuvius), whereas, in the Zr/Y vs Nb/Y scatterplot (**S3 Figure, b**) the clasts, connoted by the high Nb/Y ratios (always > 1.7), overlap the field of Campania and they are incompatible with both Tuscan/Roman Magmatic districts and Aeolian Isles.

To establish the provenance of the volcanic pozzolans at higher resolution within the Neapolitan district, the fingerprint of the analysed clast was compared to that of around 950 pyroclastic products selected from literature, comprising the main eruptive events of the Phlegraean Fields, Mt. Somma-Vesuvius, Ischia and Procida-Vivara.

In the Zr/Y vs Nb/Y diagram (**Figure 9, a**), most of the samples overlap the Phlegraean Fields' intervals, showing strong correlations with the products of the post-NYT activities. However, some clasts with Nb/Y values < 2.0 (in particular *e* and *j*) present certain affinities with geological samples of the Ischia's pre- and post-Monte Epomeo Green Tuffs (MEGT). Additionally, other pumices (especially *k*) fall close to the intervals associated with certain pre-79 CE Mt. Somma-Vesuvius juveniles.

The Nb/Zr vs La/Yb scatterplot, adopted in [29] for discrimination, does not provide relevant information (**Figure 9, b**).

Both the scatterplots indicate that the compatibility with the Phlegraean Fields of the analysed clasts is plausible, but it cannot be considered as ubiquitous. Consequently, to enhance the resolution of the analysis, non-fractional HSFE and REE from the 950 geological markers of our in-house geochemical dataset were compared in exploratory bivariate scatterplots trace-by-trace. Notably, Yb concentrations

show a relevant and coherent variability within the volcanic units of the Gulf of Naples, resulting extremely informative when plotted versus Zr, Nb and Th. A similar pattern is observed when Nd is plotted versus Rb.

In fact, in the Yb vs Zr, Yb vs Nb, Yb vs Th and Nd vs Rb scatterplots (**Figure 9, c-f**), the 9 analysed pumices systematically overlap the intervals of distribution of the Phlegraean fields, connoted by positive correlation between Yb with Zr, Nb and Th as well as between Nd with Rb. All the TSF_T9 clasts have Yb and Nd values higher than 4 and 60 ppm respectively, making them incompatible with Mt. Somma-Vesuvius eruptions, characterized by Nd values < 50 ppm and Yb values < 2 ppm except for the older series, where Yb values can reach 5 ppm. The distribution of the Ischia pre-MEGT, MEGT and post-MEGT products appears somewhat inconstant. Although Yb and Nd are positively correlated with the other trace elements, when compared to the other Phlegraean products, these trends show slightly higher ratios, particularly evident in the Yb vs Th scatterplot (see **Figure 9, e**).

Having proved a ubiquitous compatibility with the Phlegraean Fields, the investigated clasts appear to align in particular with the post-NYT eruptive events, characterized by high Zr, Th and Yb. However, in most of the diagrams, the analysed pumices show a latent compatibility with pre-NYT, NYT and CI juveniles too.

To prove with the highest degree of resolution the origin of the clasts within the Phlegraean domain, discriminant analysis (DA) was carried out. The geological markers, subdivided according to the major Phlegraean eruptive events, were used as classification factors, and the most informative trace elements adopted for provenance discrimination in the scatterplots (Zr, Nb, Y, Th, Ta, Yb, La, Nd and Rb) were selected as independent variables. The compatibility with possible sources was established considering two probabilistic terms.

For all the analysed pumices, the DA reported a 1st probability correlation, ranging from 75 to 100%, with the post-NYT volcanic products (see **Tab. 2**). This compatibility was eventually confirmed through a spider-diagram reporting the trace element profiles of the analysed pumices perfectly overlapping the intervals of the geological marker samples associated with the post-NYT eruptions (**Figure 10**).

These outcomes confirm that the weakly-lithified deposits of the late Phlegraean eruptions, outcropping around the Gulf of Naples' coastline, are the likely source of the volcanic material included in TSF samples.

Figure 7. TAS scatterplots of volcanic pozzolans (pumices, obsidians, tuffs and ignimbrites) included in TSF_T9 sample overlapped to the geochemical intervals of the main Plio-Quaternary volcanic districts of the Italian peninsula (reference for geochemical fields taken from [49] and references therein), distributed as it follows: (a) the main eruptive events of the Phlegraean Fields, comprising pre- and Campanian Ignimbrite (pre-CI/CI), pre- and Neapolitan Yellow Tuff (pre-NYT/NYT), post-NYT, and Phlegraean-correlated volcanoes of Ischia and Procida-Vivara; (b) the three main eruptive *facies* of Somma-Vesuvius; (c) the products of the Roman, Tuscan and Euganean Magmatic provinces; (d) pyroclastic products of the Aeolian Arc Isles.

Figure 8. Identification of the volcanic pozzolans in the polished sections TSF_T9A, TSF_T9B and TSF_T9C samples analysed by SEM-EDS (in yellow and light-blue dashed line) and the nine pumices analysed by both SEM-EDS and LA-ICP MS (in light-blue dashed line). In the lower part the BSE acquisitions of the nine LA-ICP-MS analysed pumices with indication of the Laser Ablation spot of analysis (the dimension of the red dots is scaled down to the instrumental 55 μm spot size).

Figure 9. Trace elements' scatterplot distribution of the pumices in TSF_T9A and TSF_T9B samples, in relation with that of juveniles' geological markers from the main eruptive *facies* of the volcanic units of the Gulf of Naples. A selection of discriminant trace elements for provenance recognition was adopted: (a) Nb/Y vs Zr/Y; (b) La/Yb vs Nb/Zr; (c) Yb vs Zr; (d) Yb vs Nd; (e) Yb vs Th; (f) Nd vs Rd. Geochemical data of reference geological markers are taken from literature, as it follows. Phlegraean Fields (eruptive activities distinguished according to [88,89]: Pre-CI [88,90–93]; CI [88,90,93–95]; post-CI [90]; Pre-NYT [88,92,93]; NYT [88,96,97]; Post-NYT [50,88,96,98–103]; comprising Epoch I, II and III according to [103]; Somma-Vesuvius (eruptive activities distinguished according to [104]: Older series – Pomici di Base < 20 k.a. BP [91,105–110]; Greenish pumice [106,108,111]; Pomici di Mercato [106,108,110,112,113]; Avellino [105,106,110,113,114]; Protohistorical series – AP1/AP6 [106,108–110]; 79 CE [105,106,108,110,113,115–117]; post-79 CE – 472 CE [105,106,108–110]; Ischia, eruptive activities distinguished according to [118]: pre-MEGT [118–122]; MEGT [118,119]; post-MEGT [118–122]; Procida-Vivara [123].

Figure 10 – Comparative spider-diagram of trace elements (average values of single clast) between the pumice glass measured by LA-ICP-MS in this study and the field of Post-NYT juveniles from literature (references for post-NYT are reported in the main text and captions of figure 9). Data were normalized to the Primitive Mantle recalculated from pyrolite (PM, data from: [124]).

Table 2. Results of the discriminant analysis of pumice samples with probabilistic association to sources within the main Phlegraean eruptions (Classification Function Coefficients and Discriminant Function Coefficients are reported at the **S3. Table**).

Clast	Provenance (1st prob.)	Highest Group	Squared distance	Prob. (%)	Provenance (2nd prob.)	2nd Highest group	Squared distance	Prob. (%)	TOT prob. (%)
j	Post-NYT	44.3087	4.61778	90.32	pre-NYT	42.0199	9.1953	9.16	99.48
p	Post-NYT	47.6212	5.88543	75.17	pre-NYT	46.4381	8.25164	23.03	98.2
o	Post-NYT	54.886	8.51028	80.03	pre-NYT	53.3371	11.6079	17.01	97.04
e	Post-NYT	35.2316	20.086	97.76	pre-NYT	31.4485	27.6522	2.22	99.98
k	Post-NYT	63.3567	12.6565	91.23	NYT	60.8802	17.6097	7.67	98.9
n	Post-NYT	39.832	17.359	96.96	pre-NYT	36.3686	24.286	3.04	100
i	Post-NYT	16.6407	57.7087	98.12	pre-NYT	12.6858	65.6184	1.88	100
t	Post-NYT	40.453	46.5635	98.34	pre-NYT	36.3725	54.7244	1.66	100
v	Post-NYT	29.2316	76.88	99.92	pre-NYT	22.1365	91.0701	0.08	100

5. Archaeological inferences

The data obtained from this research are extremely interesting from an archaeological point of view, as they offer important information regarding the building technique and the environment in which the TSF well-cistern was placed, providing a new piece of evidence on the commerce and exploitation of the Vitruvian *pulvis puteolana* in the ancient Mediterranean.

5.1 The construction technique and environment of 'Torrione' San Felice Cistern

In a context-related perspective, the building technique of the well-cistern appears extremely complex. Nowadays, due to eustatic and subsidence phenomena, the first course of bricks positioned at the

bottom of the structure lies at a depth of – 4.20/3.75 m MSL, but even in Roman times the base of the well-cistern was placed at a depth of around – 2.0 m MSL [37]. This implies that at least the foundations of the cistern, but more probably most of the structure, had to be realized underwater. Therefore, it seems difficult to think that the Romans were able to build a structure made of local *sesquipedali* bricks laid on mortar joints and mortar-coated without using cofferdams [35,125], possibly constituted of a double bulkhead (Vitr., 5.12.5). In order to work in an adequately dry space, it is even probable that machines for the lifting of the liquid infiltrations, as those described by Vitruvius (10.6.1-4) [126,127], were employed avoiding as much as possible the intrusion of saltwater during construction. This technique was historically used in Venice to build underwater, but some pieces of evidence of the use of wooden scaffoldings to allow the setting of mortar-based materials underwater, dating back to the Roman times, were documented in other sites along the Northern Adriatic coast [128]. However, this solution does not deny the opportunity to use, whereas available, hydraulic mortars, in order to fasten the bricks, improving the grip and bonding of the building materials. The natural and artificial pozzolans (volcanic ashes and crushed ceramics in particular) were processed and finely milled by the crafts to maximize their interfaces with the lime binder, thus increasing the overall hydraulic capability of the resulting mortars. In addition, the reactivity was certainly fostered by the brackish liquid medium the mortar was presumably mixed with and that came into contact with the binding materials shortly after the cofferdams were dismantled. In fact, when Phlegraean pozzolan-rich mortars are applied in masonry joints or *opus caementicium* casts for on-land buildings, where carbonation of lime-based binder is favoured by air circulation, the development of reaction processes in the matrices appears almost null and limited to the interface edges with the reactive elements [49,129]. Therefore, it is eventually clear how the buried, alkaline-rich submerged anoxic environment favoured the latent reactivity of certain aggregates over aerial carbonation, influencing the development of pozzolanic or para-pozzolanic reaction processes. This complex reaction kinetics significantly contributed to the strength and durability of the compounds along time [7,26,38,85], as already recognized for underwater concrete piers of Roman port infrastructure [7,39,85,130].

This result confirms – once again – the brilliant intuition of the Roman engineers, epitomized in the well-known Vitruvian book, regarding the prodigious strength granted by *pulvis puteolana* once employed in

the making of underwater concretes. These important findings were firstly assimilated by the indigenous workers and then reinterpreted and adapted to the construction methods and materials of the local tradition.

5.2 Commercialization and use of *pulvis puteolana*

In general terms, the results obtained in this research offer a new piece on the diffusion of the Neapolitan pozzolans in the ancient Mediterranean. The ship-trading of the "prodigious powder" from Campania up to the northernmost Adriatic route seems to be substantiated in an ever-expanding landscape of attestations, that now range from the High Imperial Age [49] to the Late Antiquity [86]. New scenarios are opened on the wide distribution of the Neapolitan pozzolans in this region, that were even employed in a context hitherto considered as marginal in the Roman world, such as the Lagoon of Venice, that, in the light of these data, now appears as deeply rooted in the commercial networks of one of the most esteemed and specialized building materials of the genuine Roman tradition. Indeed, from Aquileia, located around 11 km inland from the Adriatic coastline and around 80 km north of the Lagoon of Venice, another crucial proof of expoitation of Neapolitan pozzolans was evidenced by recent research [49]. This endorses the hypothesis that the transport of the material was operated probably as ship-ballast along the endolagunar route connecting Ravenna with *Altinum* and Aquileia [131].

Considering the dimension of the well-cistern (6.7 × 7.7 m), which may have been intended to serve fresh water to ships passing throughout the lagoon route, the quantity of mortar needed for the making of the structure was surely significant and, as a consequence, the amount of volcanic pozzolan exploited. Therefore, its supply was probably massive and intentionally-commissioned from Campania, but its use in the making of the mortars was not left to chance. As demonstrated by the different concentrations of volcanic aggregates in the analyzed samples, the local builders carefully dosed the imported material, employing the largest amounts of pozzolans in the nodal joints of the well-cistern, to ensure an optimal waterproofing. Crushed ceramics were also added for this purpose, according to the common *modus operandi* of Roman crafts of combining natural and artificial pozzolanas (pyroclastic rocks and crushed ceramics, respectively) for waterproofing the coatings of hydraulic infrastructures [26,132–135]. On the other hand, volcanic pozzolans-enriched mortars, having no ceramic additions, were mainly employed

in masonry joints and structural mortars and concretes [12,54,75,135,136], possibly for improving the strength and the cohesive properties of the compounds, as recalled by Vitruvius regarding both *pulvis puteolana* (Vitr. 2.6.1) and *harenae fossiciae* (Vitr. 2.4.1-2).

6. Conclusions

This study provides many insights in the current field of research on ancient hydraulic mortars. The main results, summarized below, can be split into three categories, comprising: (a) highlights on the kinetics of pozzolanic and para-pozzolanic reaction processes; (b) novel tools for the provenance determination of volcanic pozzolans, and (c) crucial advances on the technology of ancient pozzolanic mortars and on the trading of *pulvis puteolana* in Northern Italy in Roman times.

- **Kinetics of pozzolanic and para-pozzolanic reaction processes**. The study has provided new insights regarding the development of the peculiar reaction processes in ancient mortar-based materials in submerged anoxic environments. Being clear the role of saltwater in fostering the latent "pozzolanicity" of certain materials, the prevalent formation of Mg-based hydrate products (M-S-H and M-A-S-H) over traditional Ca-based pozzolanic ones (C-S-H and C-A-S-H) was promoted by the long-term evolution of the reactions, as the binders appear clearly Ca-depleted and feebly carbonated. This opens new perspectives on the complex kinetics of reactions occurring in ancient underwater mortars and on the crucial role played by Mg ions in these processes.
- **Tools for provenance determination of volcanic pozzolans**. A nodal element of the study was the determination of the provenance of natural pozzolans present in the composites. From a methodological point of view, the work offers new tools for measuring the geochemical fingerprint of volcanic aggregates in ancient mortar-based materials and for tracking their origin. In particular, an enriched set of discriminant diagrams was proposed, outlining the crucial role of Yb and Nd for a geochemical differentiation of juveniles within the Neapolitan magmatic district. The statistical processing of the descriptive geochemical elements with multivariate techniques, such as the discriminant analysis (DA), can constitute another useful tool to validate and crosscheck the information derived from scatterplot observations.

- **Technology of ancient pozzolanic mortars and trading of *pulvis puteolana*.** From a historical-archaeological point of view, this study documented a peculiar case of utilization of Phlegraean volcanic pozzolans in Roman times and the first attestation of this material in the lagoon of Venice. This evidence allows to reconsider this territory as strictly connected in the ancient Northen Adriatic economic-commercial routes and deeply embedded in the Roman technological awareness of genuine central-Italic tradition, thought redesigned according to the forms, methods and construction materials of the local built environment.

7. Supporting information

S1 Analytical methods. Instrumental equipment and standards.

S1 Figure Roman well-cisterns. Examples of Roman well-cistern documented in the site of Ca' Ballarin in the Northern Lagoon of Venice (up) and from Aquileia (down). Images taken from [37] (see references in the main text).

S2 Figure SEM-EDS investigation of volcanic pozzolans' mineral chemistry and iron slags.

S3 Figure TAS diagram. Trace elements' scatterplots of the pumice clasts in TSF_T9B and TSF_T9C subsamples analyzed by LA-ICP-MS; **a)** Nb/Zr vs Th/Ta scatterplot of pumice clasts in relation to the fields occupied by the Roman, Tuscan and Campanian magmatic provinces and Aeolian Arc Islands' volcanic products (compositional fields from [49] and references therein); **b)** Nb/Y vs Zr/Y scatterplot of clasts' samples in relation to the fields occupied by the Roman, Tuscan and Campanian magmatic provinces, and the Aeolian Arc Island's products (compositional fields from [49] and references therein).

S1 Table. **Major elements composition of the volcanic pozzolans**. Clasts were analyzed by SEM-EDS and described as %wt in oxides (average values and standard deviation); b.d. = Components below the detection limit.

S2 Table. Trace elements of the volcanic pozzolans. Chemical results of the selected clasts of pumice analyzed by LA-ICP-MS (average values of multiple spot-size analyses are reported and related standard deviation). Values are described as ppm

S3 Table. Coefficients of the discriminant analysis.

8. Acknowledgments

We are grateful to Dr. Alessandro Asta (Soprintendenza Archeologia, Belle Arti e Paesaggio per l'area metropolitana di Venezia e le province di Belluno, Padova e Treviso) for the support in the underwater archaeological research and endorsement to excavation activities.

9. Author contributions

Conceptualization: Simone Dilaria, Michele Secco, Jacopo Bonetto, Gilberto Artioli

Data curation: Simone Dilaria, Giulia Ricci, Michele Secco, Tommaso Giovanardi

Formal analysis: Simone Dilaria, Giulia Ricci

Funding acquisition: Simone Dilaria, Carlo Beltrame, Jacopo Bonetto, Gilberto Artioli

Investigation: Simone Dilaria, Giulia Ricci

Methodology: Simone Dilaria, Michele Secco

Project administration: Simone Dilaria, Jacopo Bonetto, Carlo Beltrame

Resources: Carlo Beltrame, Elisa Costa

Software: Simone Dilaria

Supervision: Jacopo Bonetto, Gilberto Artioli

Validation: Simone Dilaria

Visualization: Simone Dilaria (figures 1, 3, 4, 5, 6, 7, 8, 9, tables 1 and 2 and all the figures and tables in the supplementary materials), Elisa Costa (figure 2), Tommaso Giovanardi (figure 10)

Writing – original draft: Simone Dilaria (paragraphs 1, 3, 4.1, 4.3, 5.1, 5.2); Carlo Beltrame (paragraphs 2 and 5.1), Elisa Costa (paragraph 2), Giulia Ricci (paragraph 3 and 4.2), Michele Secco (paragraph 4.2), Jacopo Bonetto (paragraph 5.2)

Writing – review & editing: all the authors contributed in the review and editing of the manuscript

10. References

1. Hobbs LW, Siddall R. Cementitious materials of the ancient world. Build Roma aeterna Curr Res Rom mortar Concr. 2011;3:34–58.

2. Artioli G, Secco M, Addis A. The vitruvian legacy: Mortars and binders before and after the Roman world. Eur Mineral Union Notes Mineral. 2019;20:151–202.

3. Dilaria S, Secco M. Mortar recipes through the ages. A brief review of data from prehistory to late antiquity. In: Korać M, Golubović S, editors. Archaeol Sci. Center for New Technology Viminacium Institute of Archaeology Belgrade; 2022. p. 113.

4. Pachta V, Stefanidou M, Konopisi S, Papayianni I. Technological Evolution of Historic Structural Mortars. J Civ Eng Archit. 2014;8:846–54.

5. Lancaster LC. Pozzolans in Mortar in the Roman Empire: An Overview and Thoughts on Future Work. Mortiers Hydraul. en Méditerranée Antiq. 2019. p. 31–9.

6. Jackson MD, Logan JM, Scheetz BE, Deocampo DM, Cawood CG, Marra F, et al. Assessment of material characteristics of ancient concretes, Grande Aula, Markets of Trajan, Rome. J Archaeol Sci. 2009;36:2481–92.

7. Jackson MD, Mulcahy SR, Chen H, Li Y, Li Q, Cappelletti P, et al. Phillipsite and Al-tobermorite mineral cements produced through low-temperature water-rock reactions in Roman marine concrete. Am Mineral. 2017;102:1435–50.

8. Borges C, Santos Silva A, Veiga R. Durability of ancient lime mortars in humid environment. Constr Build Mater [Internet]. 2014;66:606–20. Available from: http://dx.doi.org/10.1016/j.conbuildmat.2014.05.019

9. Seymour LM, Maragh J, Sabatini P, Di Tommaso M, Weaver JC, Masic A. Hot mixing: Mechanistic insights into the durability of ancient Roman concrete. Sci Adv [Internet]. 2023;9. Available from: https://www.science.org/doi/10.1126/sciadv.add1602

10. MacFarlane J, Vanorio T, Monteiro PJM. Multi-scale imaging, strength and permeability measurements: Understanding the durability of Roman marine concrete. Constr Build Mater. 2021;272:12812.

11. Jackson MD, Vola G, Všianský D, Oleson JP, Scheetz BE, Brandon C, et al. Cement Microstructures and Durability in Ancient Roman Seawater Concretes. RILEM Bookseries [Internet]. 2012. p. 49–76. Available from: https://link.springer.com/10.1007/978-94-007-4635-0_5

12. Dilaria S, Secco M, Bonetto J, Ricci G, Artioli G. Making Ancient Mortars Hydraulic. How to Parametrize Type and Crystallinity of Reaction Products in Different Recipes [Internet]. RILEM Bookseries. Springer Nature Switzerland; 2023. Available from: http://dx.doi.org/10.1007/978-3-031-31472-8_4

13. Richardson IG. The nature of C-S-H in hardened cements. Cem Concr Res. 1999;29:1131–47.

14. Richardson IG. Tobermorite/jennite- and tobermorite/calcium hydroxide-based models for the structure of C-S-H: Applicability to hardened pastes of tricalcium silicate, β-dicalcium silicate, Portland cement, and blends of Portland cement with blast-furnace slag, metakaol. Cem Concr Res. 2004;34:1733–77.

15. Arizzi A, Cultrone G. Mortars and plasters—how to characterise hydraulic mortars. Archaeol Anthropol Sci. 2021;13.

16. L'Hôpital E, Lothenbach B, Le Saout G, Kulik D, Scrivener K. Incorporation of aluminium in calcium-silicate-hydrates. Cem Concr Res [Internet]. 2015;75:91–103. Available from: https://linkinghub.elsevier.com/retrieve/pii/S0008884615001131

17. L'Hôpital E, Lothenbach B, Scrivener K, Kulik DA. Alkali uptake in calcium alumina silicate hydrate (C-A-S-H). Cem Concr Res [Internet]. 2016;85:122–36. Available from: https://linkinghub.elsevier.com/retrieve/pii/S0008884616303088

18. Hong S-Y, Glasser F. Alkali sorption by C-S-H and C-A-S-H gels. Cem Concr Res [Internet]. 2002;32:1101–11. Available from: https://linkinghub.elsevier.com/retrieve/pii/S0008884602007536

19. Roosz C, Grangeon S, Blanc P, Montouillout V, Lothenbach B, Henocq P, et al. Crystal structure of magnesium silicate hydrates (M-S-H): The relation with 2:1 Mg-Si phyllosilicates. Cem Concr Res [Internet]. 2015;73:228–37. Available from: http://dx.doi.org/10.1016/j.cemconres.2015.03.014

20. Nied D, Enemark-Rasmussen K, L'Hopital E, Skibsted J, Lothenbach B. Properties of magnesium silicate hydrates (M-S-H). Cem Concr Res [Internet]. 2016;79:323–32. Available from: http://dx.doi.org/10.1016/j.cemconres.2015.10.003

21. Bernard E, Lothenbach B, Rentsch D, Pochard I, Dauzères A. Formation of magnesium silicate hydrates (M-S-H). Phys Chem Earth. 2017;99:142–57.

22. Brew DRM, Glasser FP. Synthesis and characterisation of magnesium silicate hydrate gels. Cem Concr Res [Internet]. 2005;35:85–98. Available from: https://linkinghub.elsevier.com/retrieve/pii/S000888460400256X

23. Bechor B, Sivan D, Miko S, Hasan O, Grisonic M, Rossi IR, et al. Salt pans as a new archaeological sea-level proxy: A test case from Dalmatia, Croatia. Quat Sci Rev. 2020;250:106680.

24. Alonso-Olazabal A, Ortega LA, Zuluaga MC, Ponce-Antón G, Echevarría JJ, Fernández CA. Compositional characterization and chronology of roman mortars from the archaeological site of arroyo de la dehesa de velasco (Burgo de osma-ciudad de osma, Soria, Spain). Minerals. 2020;10:393.

25. Ponce-Antón G, Zuluaga MC, Ortega LA, Mauleon JA. Petrographic and chemical-mineralogical characterization of mortars from the cistern at amaiur castle (Navarre, Spain). Minerals. 2020;10.

26. Secco M, Dilaria S, Bonetto J, Addis A, Tamburini S, Preto N, et al. Technological transfers in the Mediterranean on the verge of Romanization: Insights from the waterproofing renders of Nora (Sardinia, Italy). J Cult Herit [Internet]. 2020;44:63–82. Available from: https://doi.org/10.1016/j.culher.2020.01.010

27. Ricci G, Secco M, Addis A, Pistilli A, Preto N, Brogiolo G Pietro, et al. Integrated multi□analytical screening approach for reliable radiocarbon dating of ancient mortars. Sci Rep. 2022;12:3339.

28. Secco M, Asscher Y, Ricci G, Tamburini S, Preto N, Sharvit J, et al. Cementation processes of Roman pozzolanic binders from Caesarea Maritima (Israel). Constr Build Mater [Internet]. 2022;355:129128. Available from: https://doi.org/10.1016/j.conbuildmat.2022.129128

29. Brandon CJ, Hohlfelder RL, Jackson MD, Oleson JP. Building for Eternity: The History and Technology of Roman Concrete Engineering in the Sea. Oleson JP, editor. Oxford: OXOBOW BOOKS; 2014.

30. Lancaster LC. Mortars and plasters—How mortars were made. The literary sources. Archaeol Anthropol Sci [Internet]. 2021;13:1–20. Available from: https://doi.org/10.1007/s12520-021-01395-0

31. Greco A V. "Virtutes materiae." Il contributo delle fonti latine nello studio di malte, intonaci e rivestimenti nel

mondo romano. Sandhi, Or. 2011.
32. Felici E. Ricerche sulle tecniche costruttive dei porti romani. Atti del V Congr di Topogr Antica I porti del Mediterr età Class (Rome 2004), J Anc Topogr. 2006. p. 59–84.
33. Stanislao C, Rispoli C, Vola G, Cappelletti P, Morra V, De Gennaro M. Contribution to the knowledge of ancient Roman seawater concretes: Phlegrean pozzolan adopted in the construction of the harbour at Soli-Pompeiopolis (Mersin, Turkey). Period di Mineral. 2011;80:471–88.
34. Vola G, Gotti E, Brandon C, Oleson JP, Hohlfelder RL. Chemical, mineralogical and petrographic characterization of Roman ancient hydraulic concretes cores from Santa Liberata, Italy, and Caesarea Palestinae, Israel. Period di Mineral. 2011;80:317–38.
35. Brandon CJ, Hohlfelder RL, Jackson MD, Oleson JP. Building for Eternity. The History and Technology of Roman Concrete Engineering in the Sea. Oxford & Pholadelphia: Oxbow Books; 2014.
36. Marra F, Anzidei M, Benini A, D'Ambrosio E, Gaeta M, Ventura G, et al. Petro-chemical features and source areas of volcanic aggregates used in ancient Roman maritime concretes. J Volcanol Geotherm Res [Internet]. 2016;328:59–69. Available from: http://dx.doi.org/10.1016/j.jvolgeores.2016.10.005
37. Beltrame C, Medas S, Mozzi P, Ricci G. Roman 'Well-cisterns', Navigational Routes, and Landscape Modifications in the Venice Lagoon and Northeastern Adriatic. Int J Naut Archaeol [Internet]. 2023;52:133–57. Available from: https://doi.org/10.1080/10572414.2023.2182984
38. Bernard E, Lothenbach B, Pochard I, Cau-Dit-Coumes C. Alkali binding by magnesium silicate hydrates. J Am Ceram Soc. 2019;102:6322–36.
39. Secco M, Asscher Y, Ricci G, Tamburini S, Preto N, Sharvit J, et al. Cementation processes of Roman pozzolanic binders from Caesarea Maritima (Israel). Constr Build Mater. 2022;355.
40. Serandrei Barbero R, Albani AD, Zecchetto S. Palaeoenvironmental significance of a benthic foraminiferal fauna from an archaeological excavation in the Lagoon of Venice, Italy. Palaeogeogr Palaeoclimatol Palaeoecol. 1997;136:41–52.
41. Previato C. Modi d'uso e sistemi di messa in opera del laterizio in Italia settentrionale in età repubblicana. In: Bonetto J, Bukowiecki E, Volpe R, editors. Alle Orig del laterizio Rom Nasc e Diffus del mattone cotto nel Mediterr tra IV e I secolo aC Atti del II Convegno internazionale "laterizio" Padova April 2016. Quasar; 2019. p. 369–382.
42. Bonetto J. Maestranze greche e laterizio cotto: alle origini dell'architettura della Cisalpina. In: Bonetto J, Bukowiecki E, Vole R, editors. Alle Orig del laterizio Rom Nasc e Diffus del mattone cotto nel Mediterr tra IV e I secolo aC Atti del II Convegno internazionale "laterizio" Padova April 2016. Edizioni Quasar; 2019. p. 317–334.

43. Canal E. Archeologia della laguna di Venezia, 1960-2010. Caselle, S. 2013.

44. Ciancioisi A. Il “pozzo alla veneziana”: una soluzione complessa per un’esigenza quotidiana. Revisione dei dati archeologici sull’approvvigionamento idrico nei centri lagunari durante il medioevo. Il caso di Jesolo (VE). Archeol Mediev. 2021;48:371–88.

45. Pecchioni E, Fratini F, Cantisani E. Atlante Malte Antiche. In sezione sottile al microscopio ottico. Nardini Editore; 2014.

46. Rietveld HM. A profile refinement method for nuclear and magnetic structures. J Appl Crystallogr. 1969;2:65–71.

47. Addis A, Secco M, Marzaioli F, Artioli G, Chavarría Arnau A, Passariello I, et al. Selecting the most reliable 14C dating material inside mortars: The origin of the Padua Cathedral. Radiocarbon. 2019;61:375–93.

48. Ricci G, Secco M, Marzaioli F, Passariello I, Terrasi F, Artioli G. New Strategies in Mortar Characterization and Radiocarbon Dating. 2019 IMEKO TC-4 Int Conf Metrol Archaeol Cult Herit. Florence; 2019. p. 95–9.

49. Dilaria S, Secco M, Ghiotto AR, Furlan G, Giovanardi T, Zorzi F, et al. Early exploitation of Neapolitan pozzolan (pulvis puteolana) in the Roman theatre of Aquileia, Northern Italy. Sci Rep [Internet]. 2023;13:1–19. Available from: https://doi.org/10.1038/s41598-023-30692-y

50. Marra F, D’Ambrosio E, Sottili G, Ventura G. Geochemical fingerprints of volcanic materials: Identification of a pumice trade route from Pompeii to Rome. Bull Geol Soc Am. 2013;125:556–77.

51. D’Ambrosio E, Marra F, Cavallo A, Gaeta M, Ventura G. Provenance materials for Vitruvius’ harenae fossiciae and pulvis puteolanis: Geochemical signature and historical-archaeological implications. J Archaeol Sci Reports [Internet]. 2015;2:186–203. Available from: http://dx.doi.org/10.1016/j.jasrep.2015.01.012

52. Dilaria S, Bonetto J, Germinario L, Previato C, Girotto C, Mazzoli C. The stone artifacts of the National Archaeological Museum of Adria (Rovigo, Italy): a noteworthy example of heterogeneity. Archaeol Anthropol Sci.

53. Montana G, Cau Ontiveros MÁ, Polito AM, Azzaro E. Characterisation of clayey raw materials for ceramic manufacture in ancient Sicily. Appl Clay Sci [Internet]. 2011;53:476–88. Available from: http://dx.doi.org/10.1016/j.clay.2010.09.005

54. Columbu S, Garau AM, Lugliè C. Geochemical characterisation of pozzolanic obsidian glasses used in the ancient mortars of Nora Roman theatre (Sardinia, Italy): provenance of raw materials and historical–archaeological implications. Archaeol Anthropol Sci. 2019;11:2121-2150.

55. Dilaria S, Previato C, Bonetto J, Secco M, Zara A, De Luca R, et al. Volcanic Pozzolan from the Phlegraean Fields in the Structural Mortars of the Roman Temple of Nora (Sardinia). Heritage. 2023;6:567–86.

56. Prochaska W. The use of geochemical methods to pinpoint the origin of ancient white marbles. Mineral Petrol [Internet]. 2023; Available from: https://doi.org/10.1007/s00710-023-00833-2

57. Maritan L, Nodari L, Mazzoli C, Milano a., Russo U. Influence of firing conditions on ceramic products: Experimental study on clay rich in organic matter. Appl Clay Sci [Internet]. 2006 [cited 2014 Feb 13];31:1–15. Available from: http://linkinghub.elsevier.com/retrieve/pii/S0169131705001122

58. Matschei T, Lothenbach B, Glasser FP. The AFm phase in Portland cement. Cem Concr Res. 2007;37:118–30.

59. Bernard E. Magnesium silicate hydrate (M-S-H) characterization: temperature, calcium, aluminum and alkali. Phd thesis. Université Bourgogne Franche-Comté; 2017.

60. Secco M, Maritan L, Mazzoli C, Lampronti GI, Zorzi F, Nodari L, et al. Alteration Processes of Pottery in Lagoon-Like Environments. Archaeometry [Internet]. 2011 [cited 2014 Feb 13];53:809–29. Available from: http://doi.wiley.com/10.1111/j.1475-4754.2010.00571.x

61. Secco M, Dilaria S, Addis A, Bonetto J, Artioli G, Salvadori M. The Evolution of the Vitruvian Recipes over 500Years of Floor-Making Techniques: The Case Studies of the Domus delle Bestie Ferite and the Domus di Tito Macro (Aquileia, Italy). Archaeometry. 2018;60:185–206.

62. Dilaria S, Secco M. Analisi archeometriche sulle miscele leganti (malte e calcestruzzi). L'anfiteatro di Aquil Ric d'archivio e nuove indagini di scavo, Scavi di Aquil V, Quingentole. 2018. p. 177–86.

63. Teutonico JM, Ashall G, Garrod E, Yates T. A comparative study of hydraulic lime-based mortars. Int RILEM Work Hist Mortars Charact Tests. 1999;339–49.

64. Coutelas A. L'hydraulicité des mortiers antiques, entre préconçus et réalité: I'exemple des mor-tiers de tuileau et autres matériaux de Gaule romaine. In: Ortega F, Bouffier S, editors. Mortiers Hydraul en Méditerranée Antiq Archéologies Mé-diterranéennes 6, Aix-en-Provence. 2019. p. 17–30.

65. Houghton BF, Wilson CJN. A vesicularity index for pyroclastic deposits. Bull Volcanol [Internet]. 1989;51:451–62. Available from: http://link.springer.com/10.1007/BF01078811

66. Astolfi G, Colombara F. La geologia dei Colli Euganei. Programma. Padova; 1990.

67. Wu R, Zhang Y, Zhang G, An S. Enhancement effect and mechanism of iron tailings powder on concrete strength. J Build Eng [Internet]. 2022;57:104954. Available from: https://doi.org/10.1016/j.jobe.2022.104954

68. Papadimitriou G, Kordatos J. The brown waterproofing plaster of the ancient cisterns in Laurion and its weathering and degradation. In: Maniatis Y, Herz N, Basiakos Y, editors. Asmosia III Athens Trans 3rd Int Symp Assoc Study Marble other Stones used Antiq. 1995. p. 277–84.

69. Böttger KG, Thiedig F, Knöfel D. Keltisch-römische Mörtel vom Magdalensberg in Kärnten / Celtic-Roman

Mortars from Magdalensberg in Carinthia. Restor Build Monum [Internet]. 2002;8:19–42. Available from: https://www.degruyter.com/document/doi/10.1515/rbm-2002-5638/html

70. Katayama T. The So-called Alkali-carbonate Reaction (ACR): Its Mineralogical and Geochemical Details, With Special Reference to ASR. Cem Concr Res. 2010;40:643–75.

71. Whitney DL, Evans BW. Abbreviations for names of rock-forming minerals. Am Mineral. 2010;95:185–7.

72. Prinčič T, Štukovnik P, Pejovnik S, De Schutter G, Bokan Bosiljkov V. Observations on dedolomitization of carbonate concrete aggregates, implications for ACR and expansion. Cem Concr Res. 2013;54:151–60.

73. Dickson AG, Goyet C. Handbook of methods for the analysis of the various parameters of the carbon dioxide system in sea water. Oak Ridge. 1994.

74. Miriello D, Barca D, Bloise A, Ciarallo A, Crisci GM, De Rose T, et al. Characterisation of archaeological mortars from Pompeii (Campania, Italy) and identification of construction phases by compositional data analysis. J Archaeol Sci [Internet]. 2010;37:2207–23. Available from: http://dx.doi.org/10.1016/j.jas.2010.03.019

75. Rispoli C, De Bonis A, Guarino V, Graziano SF, Di Benedetto C, Esposito R, et al. The ancient pozzolanic mortars of the Thermal complex of Baia (Campi Flegrei, Italy). J Cult Herit. 2019;40:143–54.

76. Peccerillo A. Plio-Quaternary Volcanism in Italy: Petrology, Geochemistry. Berlin; 2005.

77. Branca S, Cinquegrani A, Cioni R, Conte AM, Conticelli S, De Astis G, et al. The Italian Quaternary Volcanism. Alp Mediterr Quat. 2023;36:221–84.

78. Le Bas MJL, Maitre RWL, Streckeisen A, Zanettin B. A Chemical Classification of Volcanic Rocks Based on the Total Alkali-Silica Diagram. J Petrol [Internet]. 1986;27:745–50. Available from: https://academic.oup.com/petrology/article-lookup/doi/10.1093/petrology/27.3.745

79. Morra V, Calcaterra D, Cappelletti P, Colella A, Fedele L, de Gennaro R, et al. Urban geology: Relationships between geological setting and architectural heritage of the Neapolitan area. J Virtual Explor. 2010;36.

80. Avanzinelli R, Lustrino M, Mattei M, Melluso L, Conticelli S. Potassic and ultrapotassic magmatism in the circum-Tyrrhenian region: Significance of carbonated pelitic vs. pelitic sediment recycling at destructive plate margins. Lithos [Internet]. 2009;113:213–27. Available from: https://linkinghub.elsevier.com/retrieve/pii/S0024493709000978

81. Boari E, Tommasini S, Laurenzi MA, Conticelli S. Transition from Ultrapotassic Kamafugitic to Sub-alkaline Magmas: Sr, Nd, and Pb Isotope, Trace Element and 40Ar–39Ar Age Data from the Middle Latin Valley Volcanic Field, Roman Magmatic Province, Central Italy. J Petrol [Internet]. 2009;50:1327–57. Available from: https://academic.oup.com/petrology/article-lookup/doi/10.1093/petrology/egp003

82. Milani L, Beccaluva L, Coltorti M. Petrogenesis and evolution of the Euganean Magmatic Complex, Veneto Region, North-East Italy. Eur J Mineral [Internet]. 1999;11:379–400. Available from: http://www.schweizerbart.de/papers/ejm/detail/11/83149/Petrogenesis_and_evolution_of_the_Euganean_Magmati?af=crossref

83. Albert PG, Tomlinson EL, Smith VC, Di Traglia F, Pistolesi M, Morris A, et al. Glass geochemistry of pyroclastic deposits from the Aeolian Islands in the last 50 ka: A proximal database for tephrochronology. J Volcanol Geotherm Res [Internet]. 2017;336:81–107. Available from: http://dx.doi.org/10.1016/j.jvolgeores.2017.02.008

84. Lancaster L, Sottili G, Marra F, Ventura G. Provenancing of lightweight volcanic stones used in ancient Roman concrete vaulting: Evidence from Rome. Archaeometry. 2011;53:707–27.

85. Jackson MD, Chae SR, Mulcahy SR, Meral C, Taylor R, Li P, et al. Unlocking the secrets of Al-tobermorite in Roman seawater concrete. Am Mineral. 2013;98:1669–87.

86. Dilaria S, Secco M, Rubinich M, Bonetto J, Miriello D, Barca D, et al. High-performing mortar-based materials from the late imperial baths of Aquileia: An outstanding example of Roman building tradition in Northern Italy. Geoarchaeology [Internet]. 2022;37:637–57. Available from: https://onlinelibrary.wiley.com/doi/10.1002/gea.21908

87. Marra F, D'Ambrosio E, Gaeta M, Mattei M. Petrochemical Identification and Insights on Chronological Employment of the Volcanic Aggregates Used in Ancient Roman Mortars. Archaeometry. 2016;58:177–200.

88. Tomlinson EL, Arienzo I, Civetta L, Wulf S, Smith VC, Hardiman M, et al. Geochemistry of the Phlegraean Fields (Italy) proximal sources for major Mediterranean tephras: Implications for the dispersal of Plinian and co-ignimbritic components of explosive eruptions. Geochim Cosmochim Acta [Internet]. 2012;93:102–28. Available from: https://linkinghub.elsevier.com/retrieve/pii/S0016703712003535

89. Peccerillo A. Campania volcanoes. Vesuvius, Campi Flegrei, Camp Volcanism [Internet]. Elsevier; 2020. p. 79–120. Available from: https://linkinghub.elsevier.com/retrieve/pii/B9780128164549000055

90. Di Renzo V, Di Vito MA, Arienzo I, Carandente A, Civetta L, D'antonio M, et al. Magmatic History of Somma–Vesuvius on the Basis of New Geochemical and Isotopic Data from a Deep Borehole (Camaldoli della Torre). J Petrol [Internet]. 2007;48:753–84. Available from: https://academic.oup.com/petrology/article-lookup/doi/10.1093/petrology/egl081

91. Di Vito MA, Sulpizio R, Zanchetta G, D'Orazio M. The late Pleistocene pyroclastic deposits of the Campanian Plain: New insights into the explosive activity of Neapolitan volcanoes. J Volcanol Geotherm Res [Internet]. 2008;177:19–48. Available from: https://linkinghub.elsevier.com/retrieve/pii/S0377027307003824

92. Pabst S, Wörner G, Civetta L, Tesoro R. Magma chamber evolution prior to the Campanian Ignimbrite and Neapolitan Yellow Tuff eruptions (Campi Flegrei, Italy). Bull Volcanol [Internet]. 2008;70:961–76. Available from: https://link.springer.com/10.1007/s00445-007-0180-z

93. Pappalardo L, Civetta L, D'Antonio M, Deino A, Di Vito M, Orsi G, et al. Chemical and Sr-isotopical evolution of the Phlegraean magmatic system before the Campanian Ignimbrite and the Neapolitan Yellow Tuff eruptions. J Volcanol Geotherm Res [Internet]. 1999;91:141–66. Available from: https://linkinghub.elsevier.com/retrieve/pii/S0377027399000335

94. Civetta L, Orsi G, Pappalardo L, Fisher RV, Heiken G, Ort M. Geochemical zoning, mingling, eruptive dynamics and depositional processes — the Campanian Ignimbrite, Campi Flegrei caldera, Italy. J Volcanol Geotherm Res [Internet]. 1997;75:183–219. Available from: https://linkinghub.elsevier.com/retrieve/pii/S0377027396000273

95. Pappalardo L, Civetta L, de Vita S, Di Vito M, Orsi G, Carandente A, et al. Timing of magma extraction during the Campanian Ignimbrite eruption (Campi Flegrei Caldera). J Volcanol Geotherm Res [Internet]. 2002;114:479–97. Available from: https://linkinghub.elsevier.com/retrieve/pii/S037702730100302X

96. Civetta L, Gallo G, Orsi G. Sr- and Nd-isotope and trace-element constraints on the chemical evolution of the magmatic system of Ischia (Italy) in the last 55 ka. J Volcanol Geotherm Res [Internet]. 1991;46:213–30. Available from: https://linkinghub.elsevier.com/retrieve/pii/037702739190084D

97. Orsi G, Civetta L, D'Antonio M, Di Girolamo P, Piochi M. Step-filling and development of a three-layer magma chamber: the Neapolitan Yellow Tuff case history. J Volcanol Geotherm Res [Internet]. 1995;67:291–312. Available from: https://linkinghub.elsevier.com/retrieve/pii/0377027394001192

98. Arienzo I, Mazzeo FC, Moretti R, Cavallo A, D'Antonio M. Open-system magma evolution and fluid transfer at Campi Flegrei caldera (Southern Italy) during the past 5 ka as revealed by geochemical and isotopic data: The example of the Nisida eruption. Chem Geol [Internet]. 2016;427:109–24. Available from: https://linkinghub.elsevier.com/retrieve/pii/S0009254116300535

99. Cannatelli C, Lima A, Bodnar RJ, De Vivo B, Webster JD, Fedele L. Geochemistry of melt inclusions from the Fondo Riccio and Minopoli 1 eruptions at Campi Flegrei (Italy). Chem Geol [Internet]. 2007;237:418–32. Available from: https://linkinghub.elsevier.com/retrieve/pii/S0009254106005213

100. Di Vito MA, Arienzo I, Braia G, Civetta L, D'Antonio M, Di Renzo V, et al. The Averno 2 fissure eruption: a recent small-size explosive event at the Campi Flegrei Caldera (Italy). Bull Volcanol [Internet]. 2011;73:295–320. Available from: http://link.springer.com/10.1007/s00445-010-0417-0

101. Smith VC, Isaia R, Pearce NJG. Tephrostratigraphy and glass compositions of post-15 kyr Campi Flegrei eruptions: implications for eruption history and chronostratigraphic markers. Quat Sci Rev [Internet]. 2011;30:3638–60. Available from: https://linkinghub.elsevier.com/retrieve/pii/S0277379111002162

102. Tonarini S, D'Antonio M, Di Vito MA, Orsi G, Carandente A. Geochemical and B–Sr–Nd isotopic evidence for mingling and mixing processes in the magmatic system that fed the Astroni volcano (4.1–3.8 ka) within the Campi Flegrei caldera (southern Italy). Lithos [Internet]. 2009;107:135–51. Available from: https://linkinghub.elsevier.com/retrieve/pii/S0024493708002223

103. Fedele L, Insinga DD, Calvert AT, Morra V, Perrotta A, Scarpati C. 40Ar/39Ar dating of tuff vents in the Campi Flegrei caldera (southern Italy): Toward a new chronostratigraphic reconstruction of the Holocene volcanic activity. Bull Volcanol. 2011;73:1323–36.

104. Santacroce R, Cioni R, Marianelli P, Sbrana A, Sulpizio R, Zanchetta G, et al. Age and whole rock-glass compositions of proximal pyroclastics from the major explosive eruptions of Somma-Vesuvius: A review as a tool for distal tephrostratigraphy. J Volcanol Geotherm Res. 2008;177:1–18.

105. Ayuso RA, De Vivo B, Rolandi G, Seal RR, Paone A. Geochemical and isotopic (Nd–Pb–Sr–O) variations bearing on the genesis of volcanic rocks from Vesuvius, Italy. J Volcanol Geotherm Res [Internet]. 1998;82:53–78. Available from: https://linkinghub.elsevier.com/retrieve/pii/S0377027397000577

106. Paone A. The geochemical evolution of the Mt. Somma-Vesuvius volcano. Mineral Petrol [Internet]. 2006;87:53–80. Available from: http://link.springer.com/10.1007/s00710-005-0103-7

107. Sparice D, Scarpati C, Mazzeo FC, Petrosino P, Arienzo I, Gisbert G, et al. New proximal tephras at Somma-Vesuvius: evidences of a pre-caldera, large (?) explosive eruption. J Volcanol Geotherm Res [Internet]. 2017;335:71–81. Available from: https://linkinghub.elsevier.com/retrieve/pii/S0377027316303973

108. Piochi M, Ayuso RA, De Vivo B, Somma R. Crustal contamination and crystal entrapment during polybaric magma evolution at Mt. Somma–Vesuvius volcano, Italy: Geochemical and Sr isotope evidence. Lithos [Internet]. 2006;86:303–29. Available from: https://linkinghub.elsevier.com/retrieve/pii/S0024493705001532

109. Somma R, Ayuso RA, De Vivo B, Rolandi G. Major, trace element and isotope geochemistry (Sr-Nd-Pb) of interplinian magmas from Mt. Somma-Vesuvius (Southern Italy). Mineral Petrol. 2001;73:121–43.

110. Joron J-L, Metrich N, Rosi M, Santacroce R, Sbrana A. Chapter 3. Chemistry and Petrography of Somma-Vesuvius. In: Santacroce R, editor. Monogr Final Somma-Vesuvius. 1987. p. 105–74.

111. Cioni R, Sulpizio R, Garruccio N. Variability of the eruption dynamics during a Subplinian event: the Greenish Pumice eruption of Somma–Vesuvius (Italy). J Volcanol Geotherm Res [Internet]. 2003;124:89–114. Available

from: https://linkinghub.elsevier.com/retrieve/pii/S0377027303000702

112. Aulinas M, Civetta L, Di Vito MA, Orsi G, Gimeno D, Férnandez-Turiel JL. The “Pomici di mercato” Plinian eruption of Somma-Vesuvius: magma chamber processes and eruption dynamics. Bull Volcanol [Internet]. 2008;70:825–40. Available from: http://link.springer.com/10.1007/s00445-007-0172-z

113. Wotzlaw J-F, Bastian L, Guillong M, Forni F, Laurent O, Neukampf J, et al. Garnet petrochronology reveals the lifetime and dynamics of phonolitic magma chambers at Somma-Vesuvius. Sci Adv [Internet]. 2022;8. Available from: https://www.science.org/doi/10.1126/sciadv.abk2184

114. Sulpizio R, Cioni R, Di Vito MA, Mele D, Bonasia R, Dellino P. The Pomici di Avellino eruption of Somma-Vesuvius (3.9 ka bp). Part I: stratigraphy, compositional variability and eruptive dynamics. Bull Volcanol [Internet]. 2010;72:539–58. Available from: http://link.springer.com/10.1007/s00445-009-0339-x

115. Cioni R, Marianelli P, Sbrana A. Dynamics of the A.D. 79 eruption: stratigraphic, sedimentologic and geochemical data on the successions of the Somma-Vesuvius southern sector. Acta Vulcanol. 1992;2:109–23.

116. Cioni R, Civetta L, Marianelli P, Metrich N, Santacroce R, Sbrana A. Compositional Layering and Syn-eruptive Mixing of a Periodically Refilled Shallow Magma Chamber: the AD 79 Plinian Eruption of Vesuvius. J Petrol [Internet]. 1995;36:739–76. Available from: https://academic.oup.com/petrology/article-lookup/doi/10.1093/petrology/36.3.739

117. Marturano A, Aiello G, Barra D, Fedele L, Grifa C, Morra V, et al. Evidence for Holocenic uplift at Somma-Vesuvius. J Volcanol Geotherm Res [Internet]. 2009;184:451–61. Available from: https://linkinghub.elsevier.com/retrieve/pii/S0377027309002297

118. Brown SK, Crosweller HS, Sparks RSJ, Cottrell E, Deligne NI, Guerrero NO, et al. Characterisation of the Quaternary eruption record: analysis of the Large Magnitude Explosive Volcanic Eruptions (LaMEVE) database. J Appl Volcanol [Internet]. 2014;3:5. Available from: https://appliedvolc.biomedcentral.com/articles/10.1186/2191-5040-3-5

119. Tomlinson EL, Albert PG, Wulf S, Brown RJ, Smith VC, Keller J, et al. Age and geochemistry of tephra layers from Ischia, Italy: constraints from proximal-distal correlations with Lago Grande di Monticchio. J Volcanol Geotherm Res [Internet]. 2014;287:22–39. Available from: https://linkinghub.elsevier.com/retrieve/pii/S0377027314002856

120. Melluso L, Morra V, Guarino V, de’ Gennaro R, Franciosi L, Grifa C. The crystallization of shoshonitic to peralkaline trachyphonolitic magmas in a H2O–Cl–F-rich environment at Ischia (Italy), with implications for the feeder system of the Campania Plain volcanoes. Lithos [Internet]. 2014;210–211:242–59. Available from:

https://linkinghub.elsevier.com/retrieve/pii/S0024493714003685
121. Brown RJ, Orsi G, De Vita S. New insights into Late Pleistocene explosive volcanic activity and caldera formation on Ischia (southern Italy). Bull Volcanol. 2008;70:583–603.
122. Poli S, Chiesa S, Gillot PY, Gregnanin A, Guichard F. Chemistry versus time in the volcanic complex of Ischia (Gulf of Naples, Italy): evidence of successive magmatic cycles. Contrib to Mineral Petrol. 1987;95:322–35.
123. De Astis G, Pappalardo L, Piochi M. Procida volcanic history: new insights into the evolution of the Phlegraean Volcanic District (Campania region, Italy). Bull Volcanol [Internet]. 2004;66:622–41. Available from: http://link.springer.com/10.1007/s00445-004-0345-y
124. McDonough WF, Sun S -s. The composition of the Earth. Chem Geol [Internet]. 1995;120:223–53. Available from: https://linkinghub.elsevier.com/retrieve/pii/0009254194001404
125. Giuliani CF. L'edilizia nell'antichità. Ia ed. 199. Roma; 2006.
126. Wikander Ö. Handbook of Ancient Water Technology. Brill, Lei. 2000.
127. Antico Gallina M. Le pompe idrauliche di Mediolanum: dalle particolarità costruttive alla relazione con i caratteri ambientali , n. In: Minonzio F, editor. Probl di Macch ambito Rom Macch Idraul nella Lett Tec nelle fonti storiografiche e nelle evidenze Archeol di età Imp. Archeologi. Como; 2004. p. 125–42.
128. Magnani S, Bianchig P, P. V. Il ponte romano alla Mainizza e la via Aquileia-Emona. Aquielia Nostra LXXVI. 2005;81–136.
129. Dilaria S, Marinello A, Zara A. Analisi archeometriche delle malte aeree e pozzolaniche del tempio di Esculapio. Risult Prelim Quad Norensi. 2022;9:225–38.
130. Jackson M, Vola G, Gotti E, Zanga B. Roman Sea-Water Concretes and their Material Characteristics. In: Oleson JP, editor. Build Eternity Hist Technol Rom Concr Eng Sea. Oxbow Books; 2014. p. 141–87.
131. Rosada G. La direttrice endolagunare e per acque interne della Decima Regio Maritima: tra risorsa naturale e organizzazione antropica. La *Venetia* nell'area padano-danubiana Le vie di Comun Atti del convegno internazionale (Venezia, 6-10 April 1988). Padova; 1990. p. 153–82.
132. Bugini R, Salvatori A, Capannesi G, Sedda AF, D'agostini C, Giuliani CF. Investigation of the characteristics and properties of the "cocciopesto" from the ancient Roman period, in Conservation of Stone and Other Materials. In: Thiel M-J, editor. Proc Int RILEM/ UNESCO Congr. London; 1993. p. 386–93.
133. Chiocchini U. The Roman cistern of road Cristoforo Colombo, Rome, Italy: operating mode and connection with the old Appian Way. Archaeol Anthropol Sci [Internet]. 2019;11:321–36. Available from: http://link.springer.com/10.1007/s12520-018-0606-x

134. Rispoli C, De Bonis A, Esposito R, Graziano SF, Langella A, Mercurio M, et al. Unveiling the secrets of Roman craftsmanship: mortars from Piscina Mirabilis (Campi Flegrei, Italy). Archaeol Anthropol Sci. 2020;12.

135. Seymour LM, Tamura N, Jackson MD, Masic A. Reactive binder and aggregate interfacial zones in the mortar of Tomb of Caecilia Metella concrete, 1st C. BCE, Rome. J Am Ceram Soc. 2021;1–16.

136. Dilaria S, Previato C, Secco M, Busana MS, Bonetto J, Cappellato J, et al. Phasing the history of ancient buildings through PCA on mortars' mineralogical profiles: the example of the Sarno Baths (Pompeii). Archaeometry. 2022;1–17.

Figure 1

Click here to access/download;Figure;Fig_1.jpg

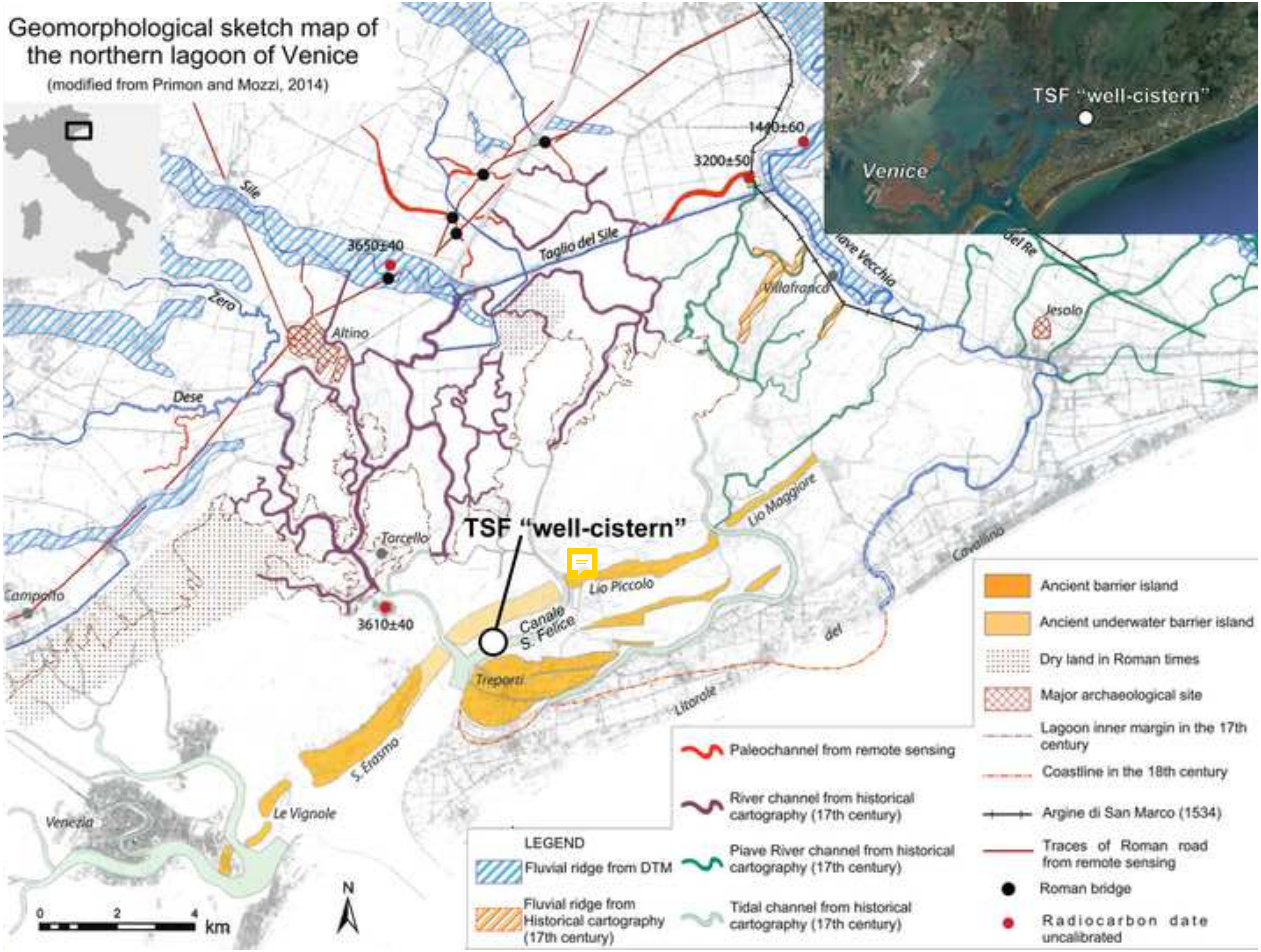

Figure 2

Click here to access/download;Figure;Fig_2.jpg

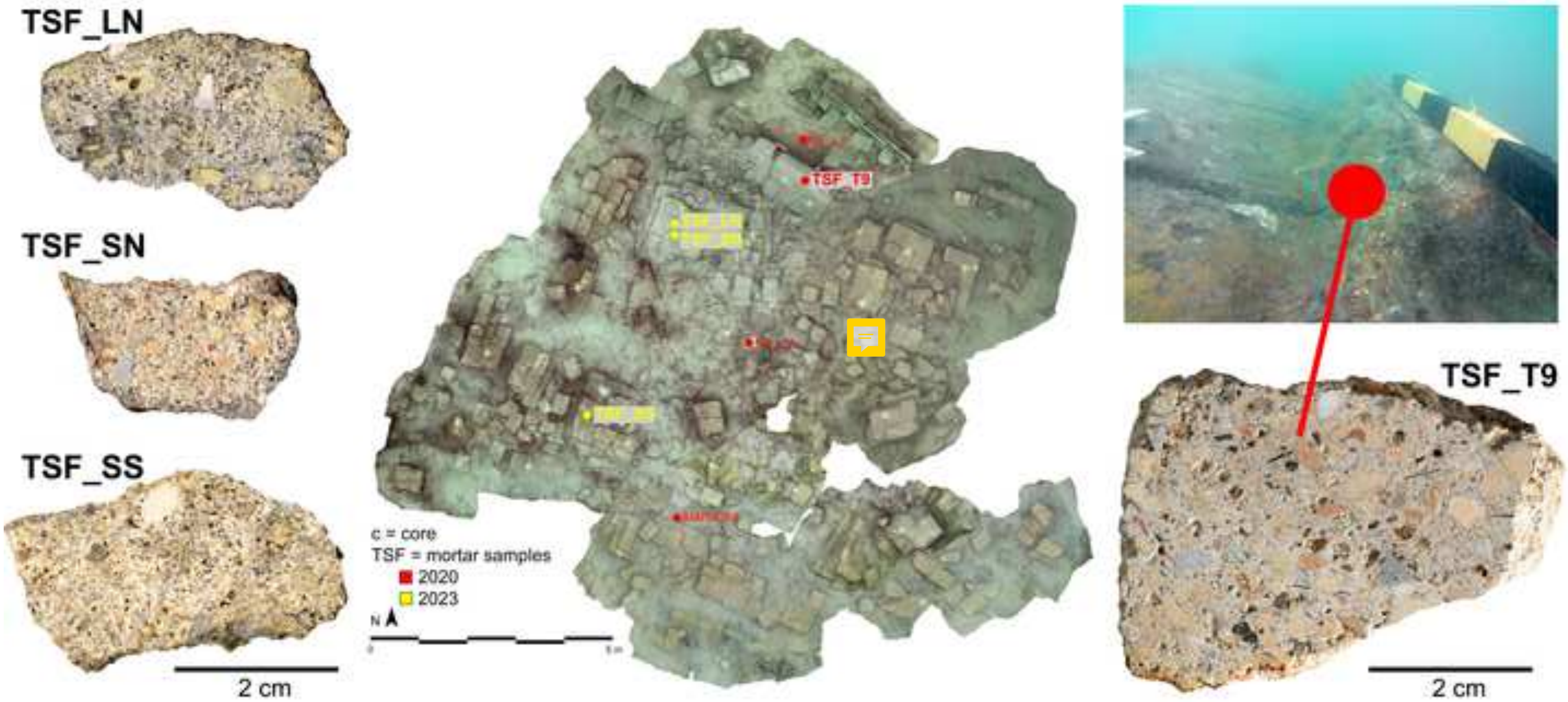

Figure 3

Click here to access/download;Figure;Fig_3.jpg

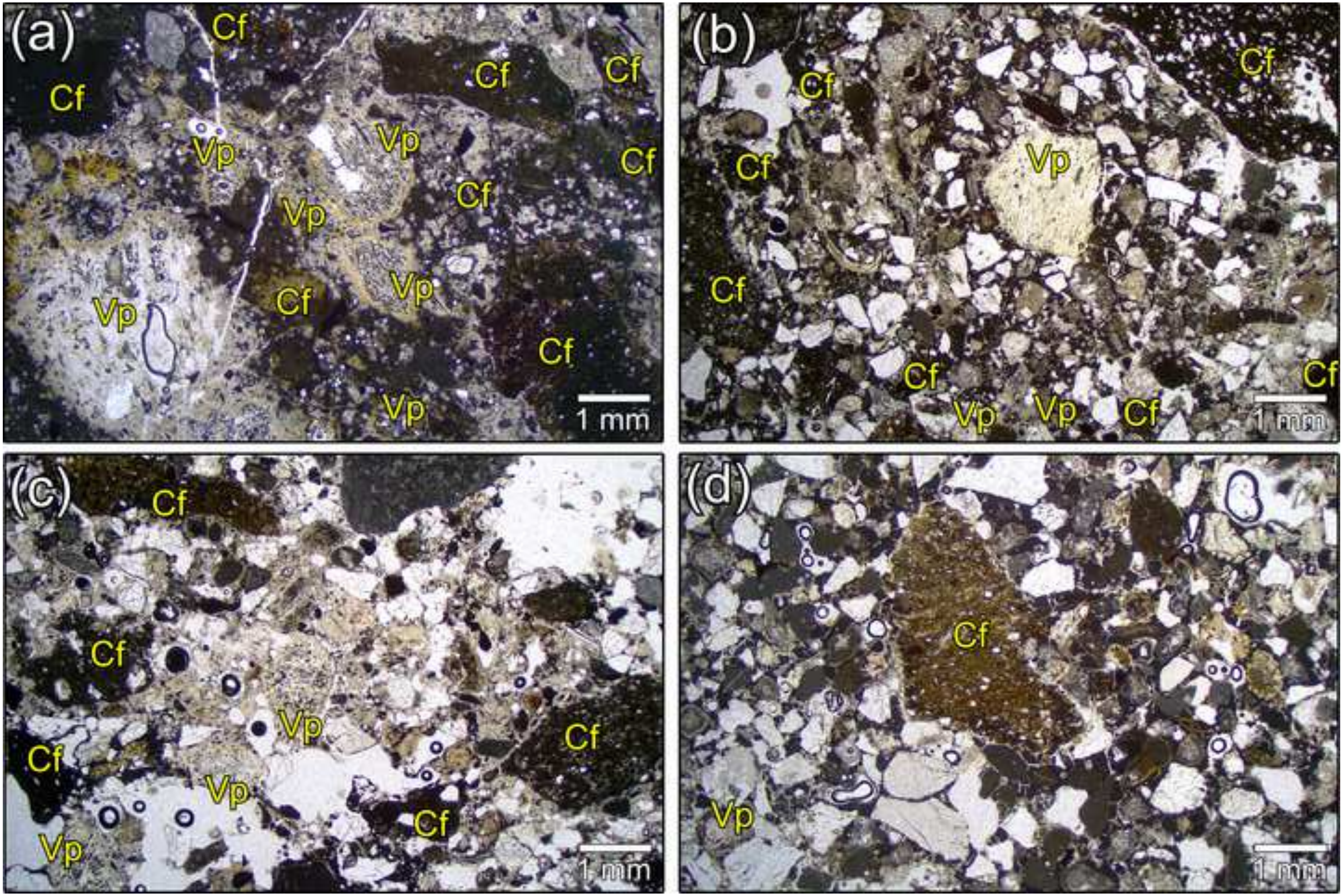

Figure 4

Click here to access/download;Figure;Fig_4.jpg

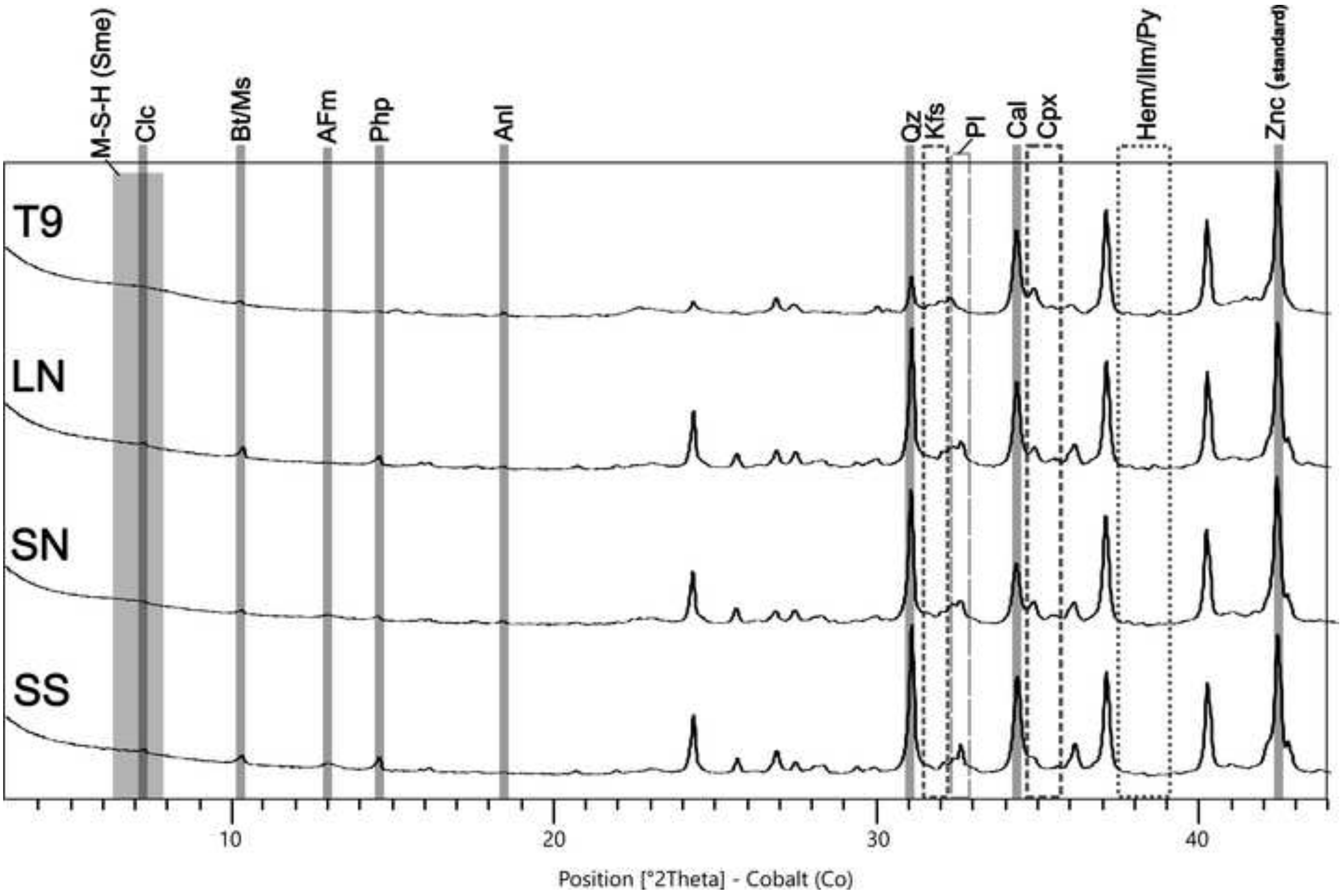

Figure 5

Click here to access/download;Figure;Fig_5.jpg

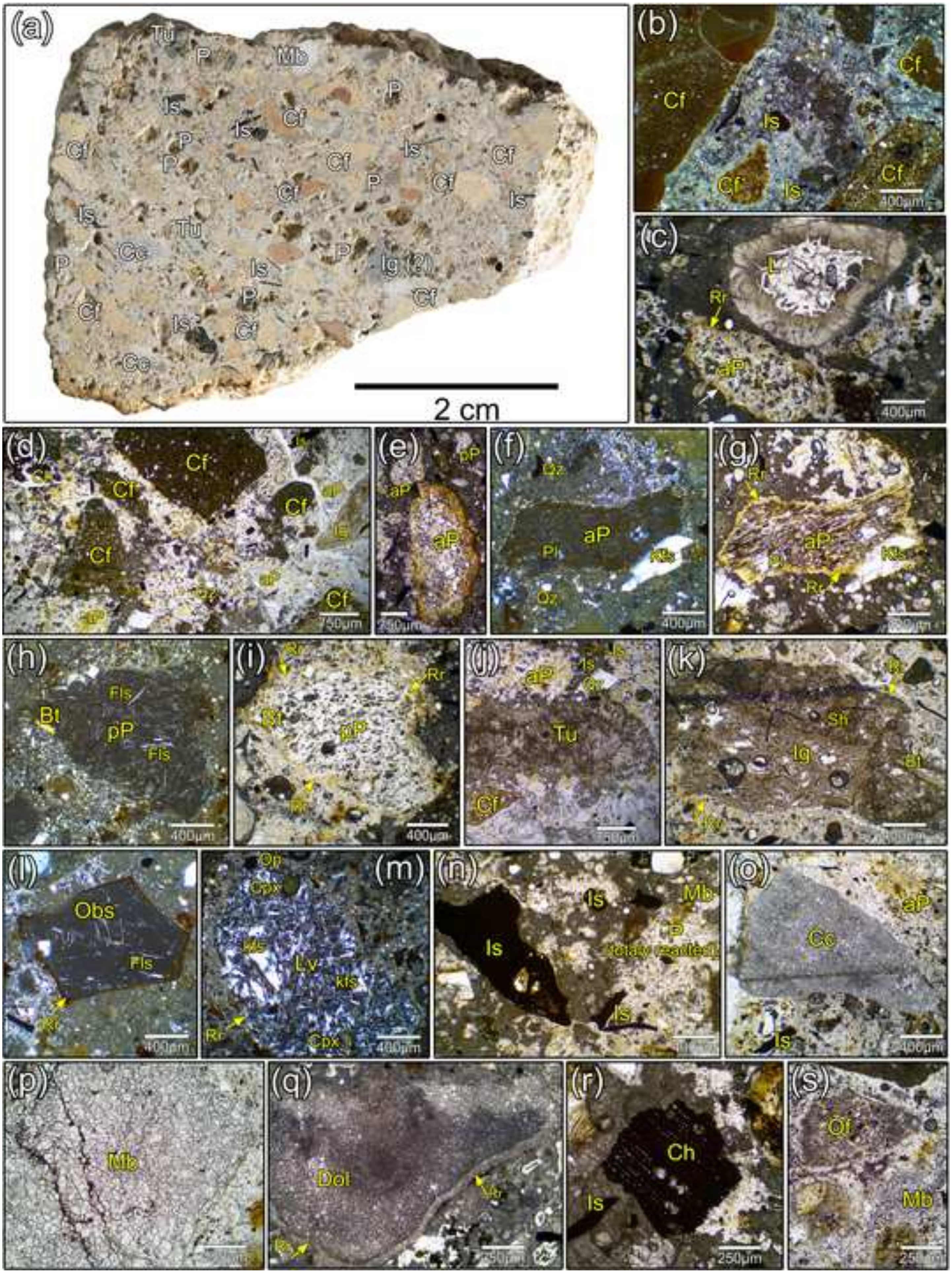

Figure 6
Click here to access/download;Figure;Fig_6.jpg

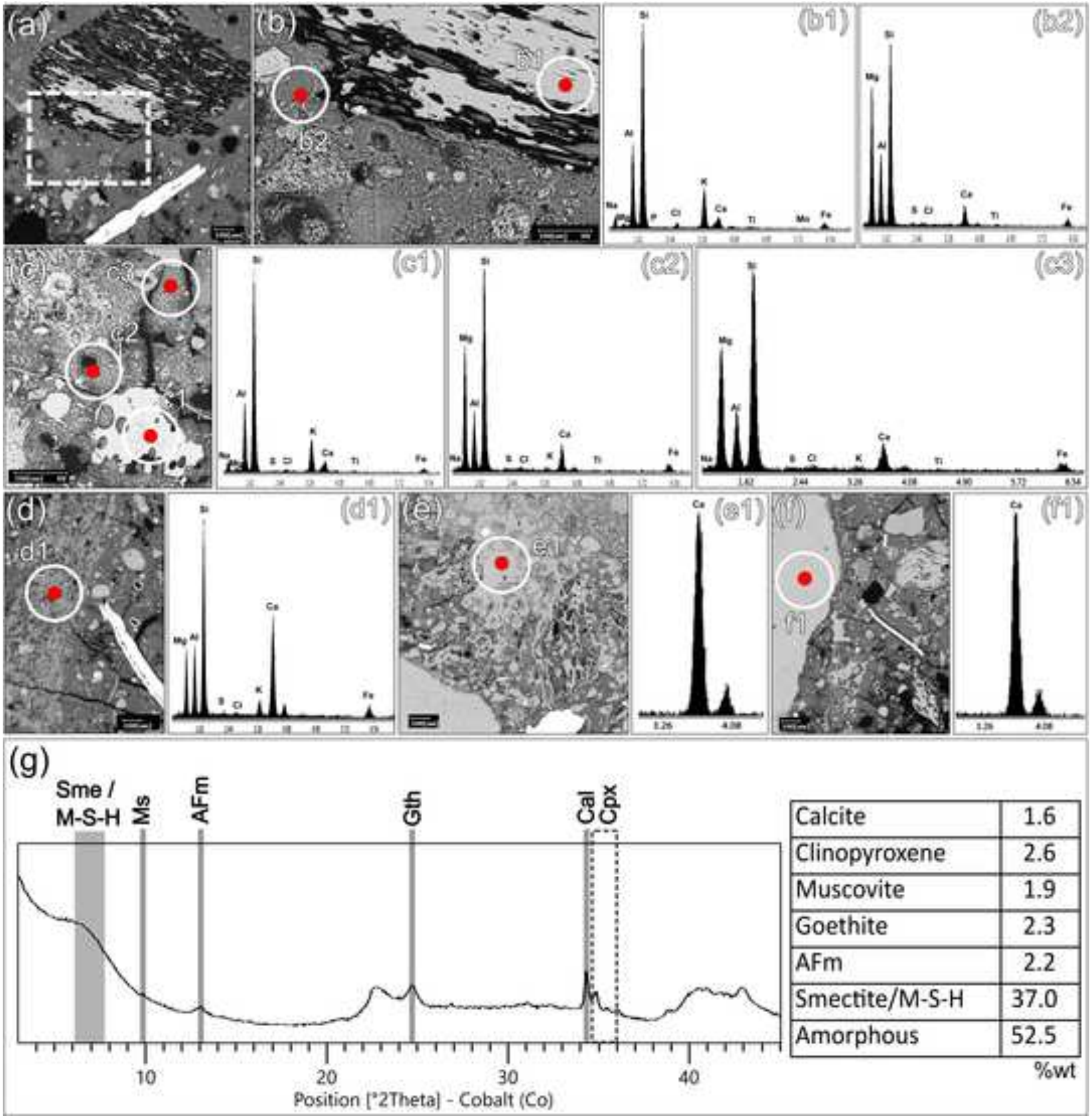

Figure 7

Click here to access/download;Figure;Fig_7.jpg

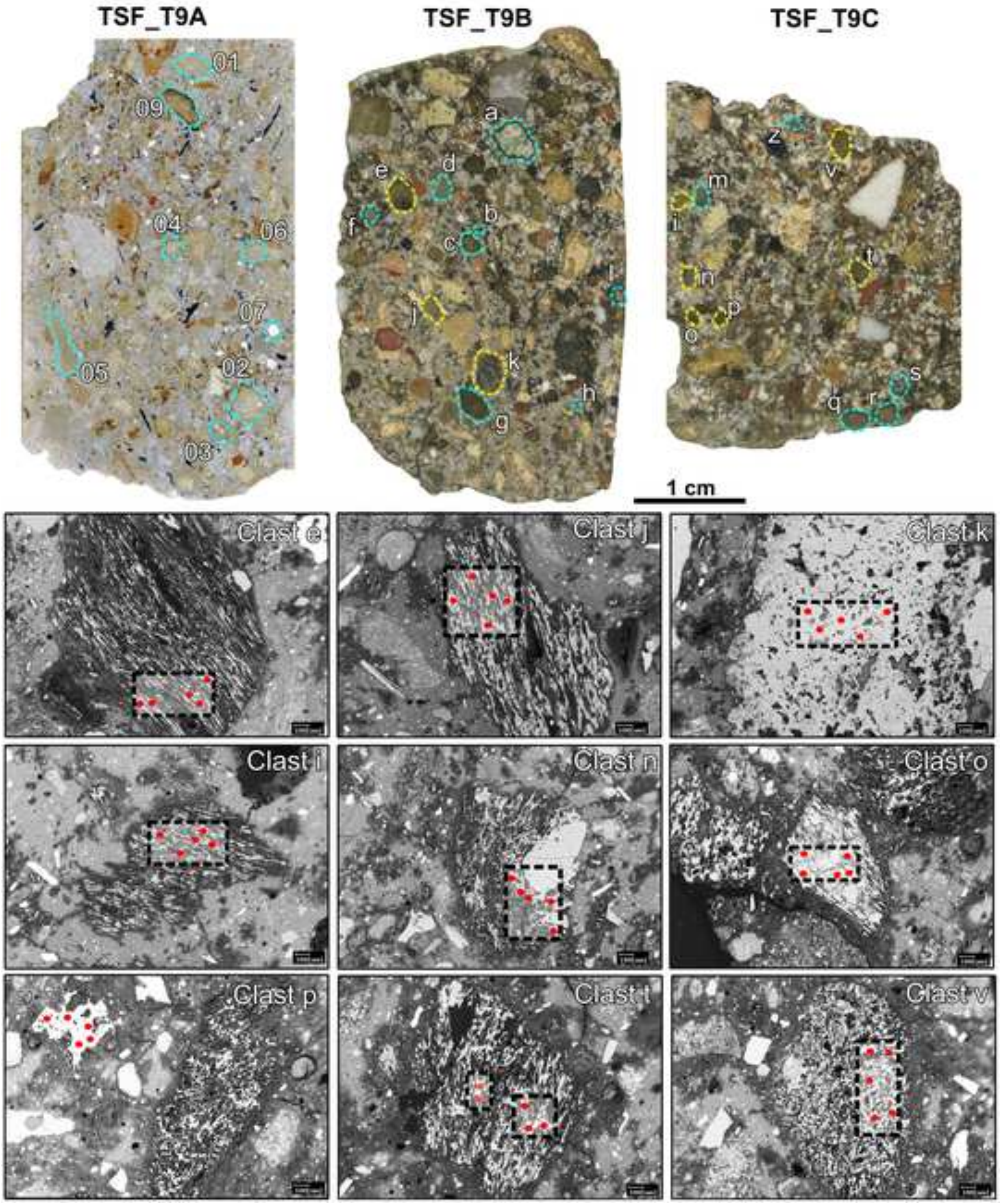

Figure 8 Click here to access/download;Figure;Fig_8.jpg

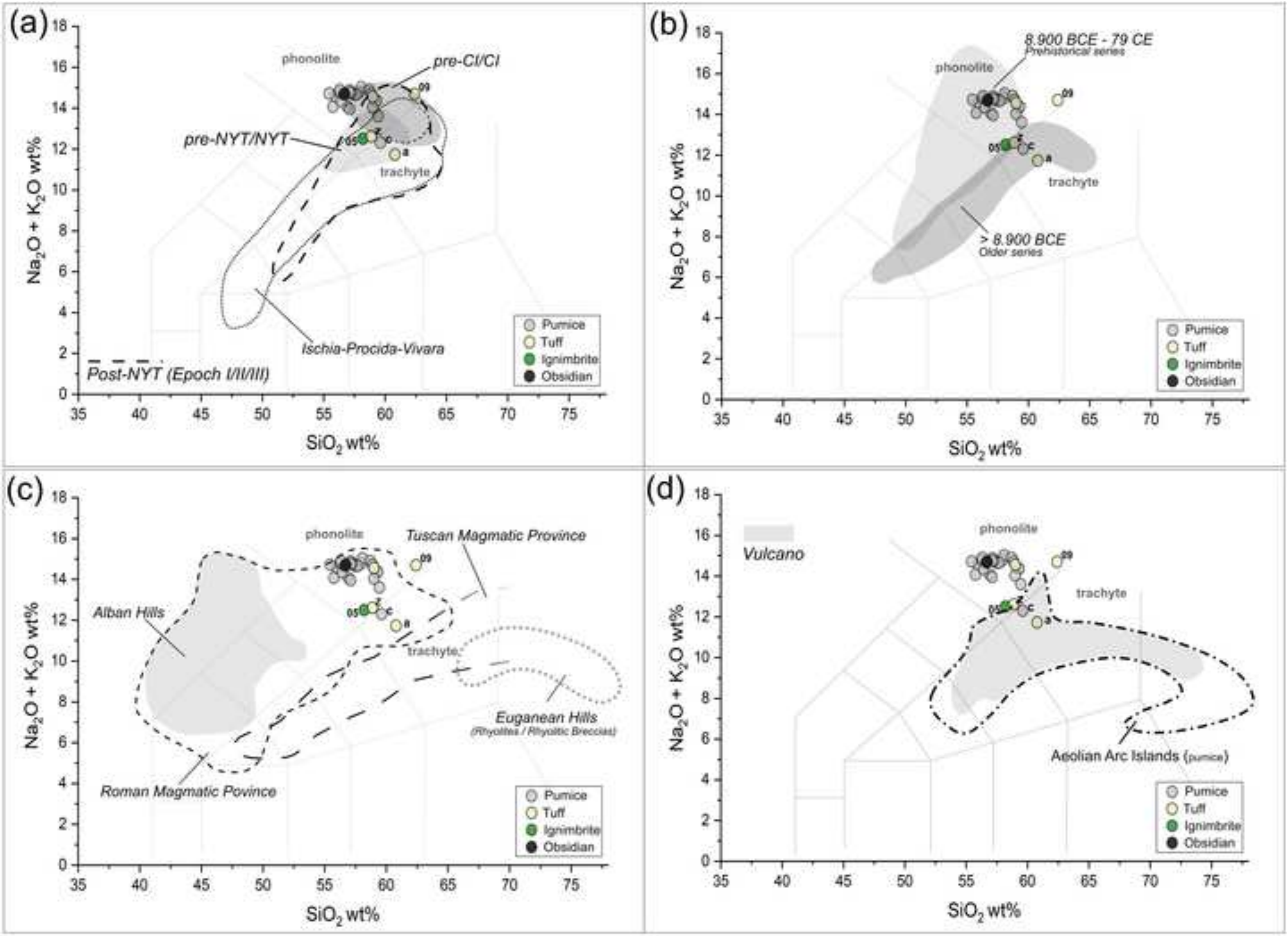

Figure 9

Click here to access/download;Figure;Fig_9.jpg

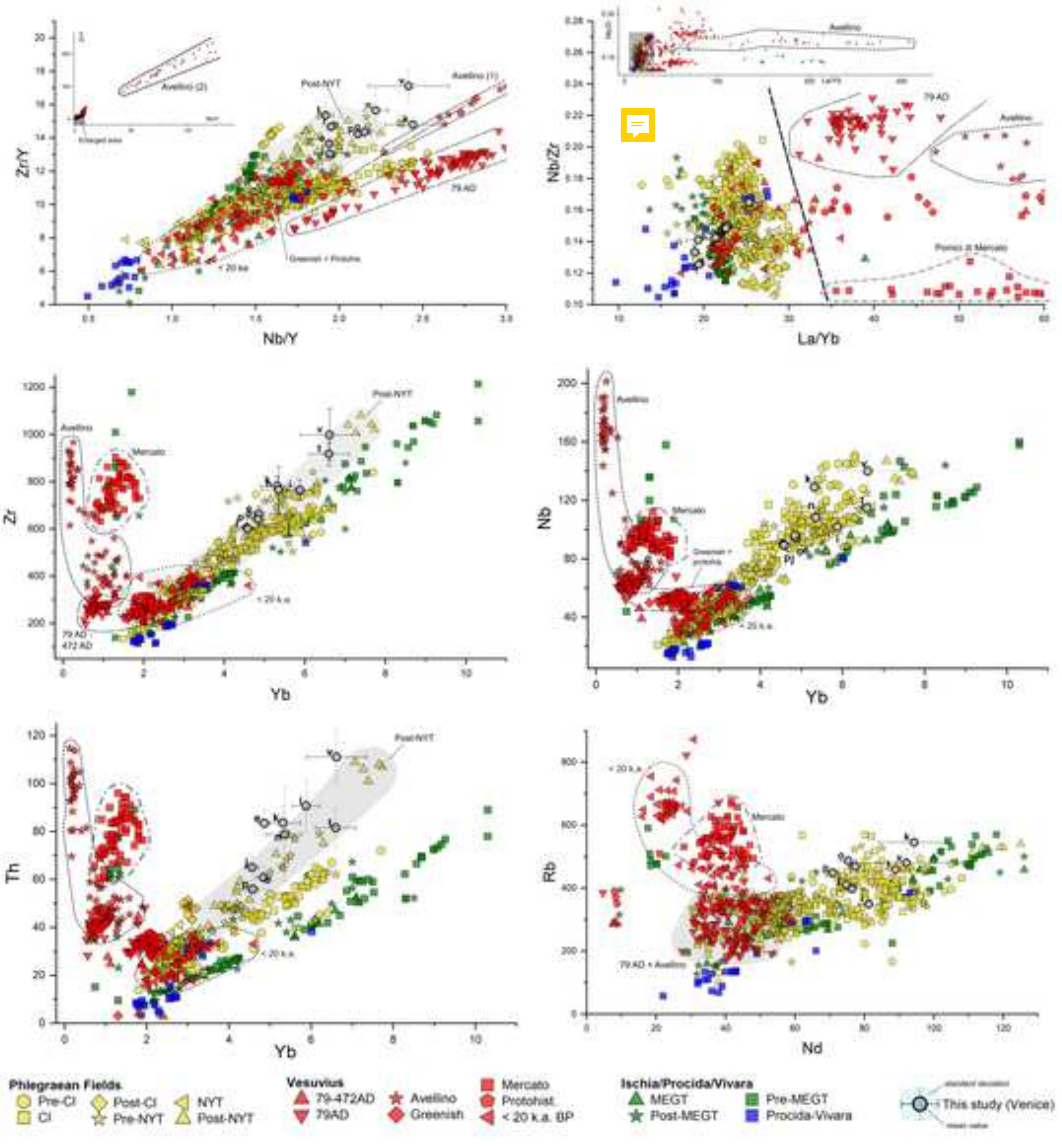

Figure 10

Click here to access/download;Figure;Fig_10.jpg

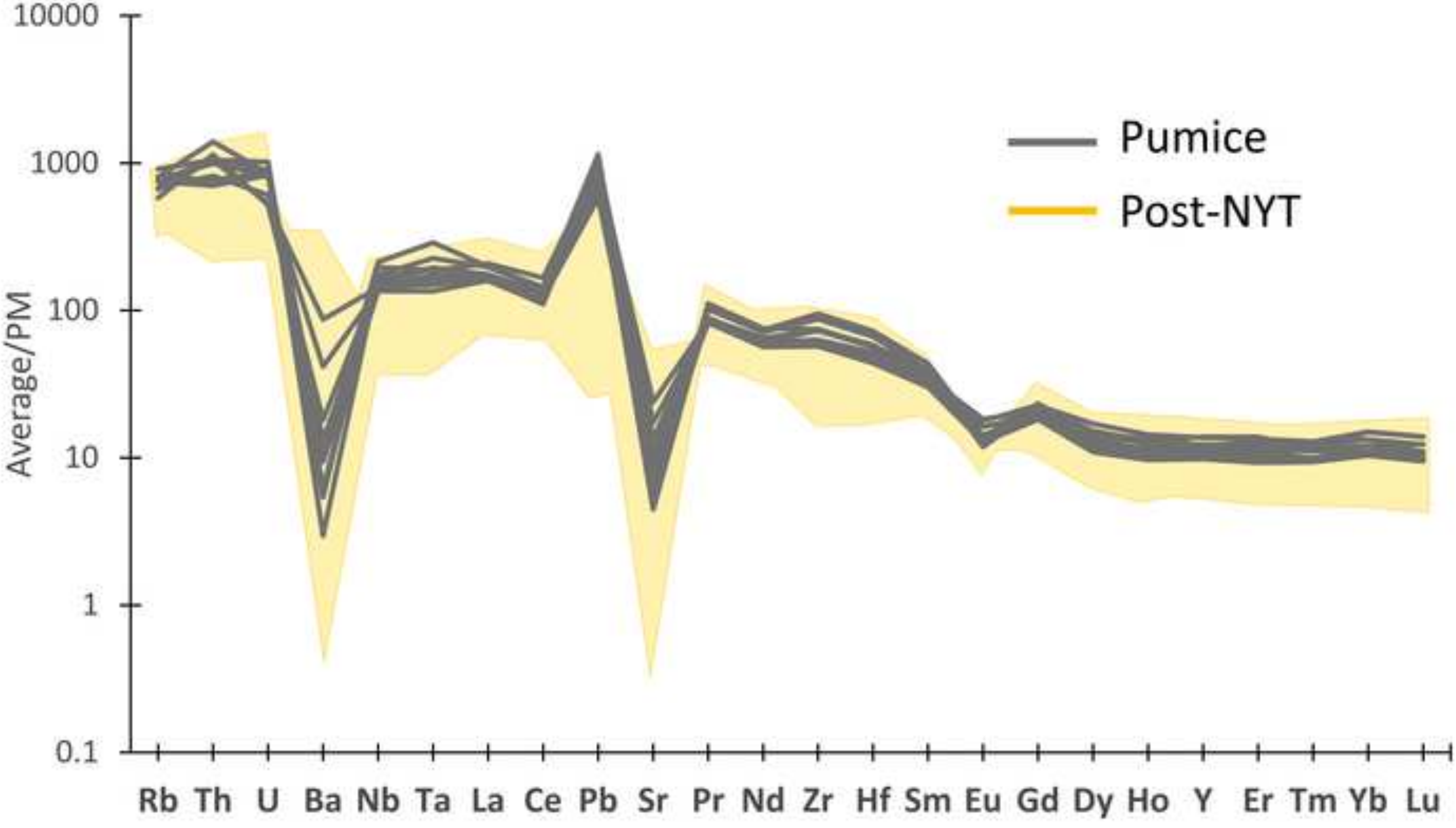

Click here to access/download
Supporting Information
Supporting_Information.docx
