# Supplementary material for: Vitruvian binders in Venice: First evidence of Phlegraean pozzolans in an underwater Roman construction in the Venice Lagoon
Source: PLoS One. 2024 Nov 22;19(11):e0313917. doi: 10.1371/journal.pone.0313917 (PMC11584134; doi:10.1371/journal.pone.0313917)
Supplement: Attachment — Submitted filename: PONE-D-23-36360_Review_Comments.pdf [file pone.0313917.s010.pdf]

Vitruvian binders in Venice: first evidence of Phlegraean pozzolans in an underwater Roman construction in the Venice Lagoon
PONE-D-23-36360

The principal contribution of this manuscript is a chemical evaluation intended to demonstrate that volcanic glass compositions of pumice fragments in the mortar of an underwater "well-cistern" structure in a lagoon in Venice indicate a lithologic provenance from the Campi Flegrei volcanic district in the Gulf of Naples. An orthophoto image (Figure 2) identifies the sampling sites, yet it is difficult to decipher the actual well-cistern and its history of submersion in the lagoon.

Despite the title, "Vitruvian binders in Venice", the actual passages from *de Architectura* are not given – nor is the relevance of these passages to the Venice lagoon mortars directly justified by the material analyses. Instead, there are oblique references that leave the reader confused as to the nature of a "prodigious powder", misleading in several respects, as described below and in the comments in the pdf.

Petrographic and SEM-EDS microscopy and X-ray diffraction analyses describe the mortar fabrics. An emphasis is placed on the development of magnesium-enriched binders and their relevance to fluids in the lagoonal environment, yet this discussion is based on only four EDS point analyses in low magnification images (Figure 6 b2, c2, c3, d1) and no direct information regarding fluid compositions in the lagoon. To accurately describe "kinetics of pozzolanic and para-pozzolanic reaction processes" more data is needed, such as high resolution SEM-EDS maps of relevant microstructures, high magnification images of the phase morphologies, and a potential resolution of the AFm phases in X-ray diffraction analyses, as hydrotalcite, for example. Section 5.1 is difficult to follow since there are no diagrams outlining the well-cistern structure (possibly shown in Fig. S1?)and relationships with changing water level from ancient to modern time.

The Introduction, unfortunately, contains many inaccuracies. Vitruvius did not use the term "pulvis puteolana" in *de Architectura* and never referred to Puteoli. There is also confusion over the use of chemical terms, such as "compound", "liquid solutions (and "aliquot" in the Results); aerial lime-based mortars; and archaeological context. (Vitruvius wrote *de Architectura* in 30 BCE, for Octavian (not Augustus in 1st C CE)). Results presented in the Introduction should be moved to the Conclusions section and statements questioning the validity of the LA-ICP-MS analyses of "unreacted glass" should be fully resolved.

Comments in the pdf identify many other issues in the text. Each of these should be carefully addressed by the authors. Some further explanations are included here. I read quickly through the last sections of this discussion and this will require a more complete review.

I have great respect for the publications of these researchers and their identifications of pumiceous aggregate from the Gulf of Naples volcanic district(s) in distant concrete structures. Here, however, the errors and inconsistencies are sufficiently complex and pervasive so as to require a thorough re-conception of the manuscript. My recommendation is for major revision, but this is at the discretion of the editor. All co-authors should carefully read the re-formulated manuscript and verify that all statements are fully validated and internally consistent (so that this work does not fall to a reviewer). I hope that these comments will provide assistance in improving the publication for future submission.

Marie Jackson

Specific Comments

1 "Vitruvius" and "Vitruvian" appear many times in the manuscript yet the text of the actual passages from *de Architectura* are not given – nor is the relevance of these passages to the Venice lagoon mortars directly justified by the material analyses. Instead, there are oblique references that leave the reader confused as to the nature of a "prodigious powder":

Line 35: "Vitruvian mention to the prodigious effects of *pulvis puteolana* used in seawater concrete (Vitr., 5.12.2-3)"

Line 68: Both Vitruvius (Vitr. 2.6.1-2; 5.12.2) and Pliny (Pliny 16.202; 35.166) exalted the outstanding properties of the *pulvis puteolana*, referred to a prodigious powder outcropping around the modern town of Pozzuoli (Bay of Naples
Line 505: "exploitation of the "Vitruvian *pulvis puteolana*" in the ancient Mediterranean.
Line 537: "Vitruvian book, regarding the prodigious strength granted by *pulvis puteolana* once employed in the making of underwater concretes"

a) In fact, Vitruvius never used the term "pulvis puteolana". He simply states "pulvis" (or dust). The Latin text of 2.6.1-2 and 5.12.2-3 appears at the end of this review. Attribution of an origin from Puteoli should correctly go to Seneca, *Q Nat*, or *Pliny, HN*:
p. 2, *Building for Eternity*, 2014, Oxbow Books.
In Latin, Vitruvius' term for the pumiceous, poorly consolidated volcanic ash that crops out "in the vicinity of Baiae and the territory of the municipalities around Mount Vesuvius" in the northwest sector of the Gulf of Naples was pulvis, "powder" or "dust" (De arch 2.6.1). This term refers to its finer grain size distribution, as compared with the granular scoriaceous ash or excavated sands (harenae fossiciae) of the region around Rome. Vitruvius thus indicates that the powdery ash used in first century BC came from either the Flegrean Fields near Puteoli or the Somma-Vesuvius volcanic districts (Fig. 7.2). The term, Puteolanus pulvis, or "dust (or powder) from Puteoli," occurs in two of the three passages by ancient authors that mention pulvis (Seneca, Q Nat 3.20.3; p. 26, Passage 14; Pliny, HN 16.202; pp. 26–27, Passage 15). In Pliny HN 35.167 (p. 27, Passage 16) the phrase is a pulvere Puteolano. Vitruvius does not attach a locative adjective, but simply states pulvis.

b) The hypothesis stating the relevance of the materials to the descriptions in *de Architectura* is not directly tested and validated.

These issues create a highly problematic foundation for the manuscript. Substantial revision is needed to resolve the textural errors, add the relevant descriptions from *de Architectura*, and state explicitly how these are validated by the analytical results.

2 Section 4.2 has a lengthy discussion of magnesium-enriched phases in the binding matrix. This is of interest, yet there seem to be only four EDS point analyses in low magnification images related to this process (Figure 6). The XRD analyses of Figure 4 state AFm phases, but these are not identified separately, e.g. as hydrotalcite. More data is needed – for example, high resolution SEM-EDS maps of instructive microstructural environments distinguishing pozzolanic reaction processes from subsequent authigenic processes, occurring possibly after the full consumption of calcium-hydroxide. In its current form, this hypothesis remains speculative.

3 Line 27, Abstract
This statement, "significant reaction processes the analysed pozzolans were object of, seriously affecting their geogenic chemical and petro-mineralogical features", calls into question preceding sentence, a "conclusion", "pyroclastic aggregates from the Phlegraean fields (pulvis puteolana) were added to the mortars". Please revise and clarify.
First, present the analytical data, Second, temper the conclusion: for example, "even so, the lithological origin of the tephra seems to the Campi Flegrei volcanic district."
A clarification is needed in the discussion section, stating quantitatively why the LA-ICP-MS data can be considered as representative of volcanic glass compositions.

4 Line 32
"perfect" and "perfectly" appear 6 times in the manuscript, with no justification for what "perfect" actually means: "having all the required or desirable elements, qualities, or characteristics; as good as it is possible to be; without fault, faultless, flawless."
"Perfect" is a qualitative assessment – not a scientific descriptor.
The mortars are not flawless materials, please use a different descriptor. Neither is the preservation "perfect", since there has apparently been much "decay" of the components of the original mortar fabric. Jackson et al. use the term "beneficial corrosion."

“Preservation”: the activity or process of keeping something valued alive, intact, or free from damage or decay.

5 Line 47
This is not correct. "Aerial lime-based mortars" develop their binding characteristics through carbonation. That process is antithetical to mortars with pozzolans that develop mainly silicate binding components.
Do you mean lime-based mortars in sub-aerial construction? or architectural applications?
Revision is needed. See:
https://www.researchgate.net/publication/358625402_AERIAL_LIME_MORTARS_AN_INTRODUCTION_AND_A_BRIEF_DISCUSSION

6 Line 68
“Both Vitruvius (Vitr. 2.6.1-2; 5.12.2) and Pliny (Pliny 16.202; 35.166) exalted the outstanding properties of the *pulvis puteolana*, referred to a prodigious powder outcropping around the modern town of Pozzuoli (Bay of Naples).”
Puteoli and “pulvis puteolana” is never referenced in *de Architectura*.
Also,
1) "exalted": definition involves a person and is not justified by the text passages: (of a person or their rank or status) placed at a high or powerful level; held in high regard; in a state of extreme happiness. Please revise.
2) "prodigious", "powder", "outcropping" : the definitions of these words are at odds with the actual behavior of the pyroclastic deposits. Without the text passages the reader will not have a context for this oblique description.

Although the Gulf of Naples tephra (and tuff) deposits have a prominent fine-ashed size fraction (de’Gennaro et al 1999, https://www.sciencedirect.com/science/article/abs/pii/S0377027399000402) they also contain a substantial coarse ash to lapilli sized fraction. Therefore, referring to these deposits as “powder” is misleading. Please use the correct volcanological terms, defining them where appropriate.

7 Line 311
“The backscattered electron images, as presented in Figure 6, reveal that most of the pozzolanic aggregates are profoundly reacted with the binder.”
This sentence is very confusing. Which are the pozzolanic aggregates? ... only the pumice? What data justifies "pozzolanic" reaction? Did the pozzolanic products then react with the binding matrix? Please separate out the concepts and describe them one by one after first presenting the analytical data.

Each clast type should be identified in the caption for each panel (and in the graphic as well, to assist the reader). Please also add a label to each EDS analysis panel, stating the phase from which the compositional analysis is derived.

The term ”volcanic pozzolans” adds more confusion. It attributes reactive capabilities to these particles that have not been validated through analyses. These appear to be pumice fragments, possibly with accretionary ash rims. (The astute reader would also wonder why the ceramic particles have no pozzolanic attributes.)

8 319
Fig. 6(g) is a single EDS point analysis in a very low magnification SEM-BSE image. High magnification image(s) and analyses are needed to justify the interpretation of Mg-binding phase (s).
How can a "paracrystalline" hydrated phase be the "principal crystalline” phase?
aliquot: This means each smaller component of the sample. It does not make sense here.
An aliquot is a piece or a sub-sample of a larger whole, particularly one extracted for chemical analysis or other treatment. Each smaller component of the sample is referred to as an aliquot, and it has the same properties as the original sample.

8 Line 537

This is highly problematic. As previously noted, Vitruvius never used the term "pulvis puteolana". Furthermore, there is no "prodigious strength" – compressive strength measurements are uniformly low in the ancient concretes and their modern replicas, < 9 MPa (Oleson et al. 2006, DOI: 10.1017/S1047759400006255).

Why is *de Architectura* never mentioned in the text?

1) The photomicrographs and micrographs in the manuscript show many clasts greater than ash-sized (or sand-sized, < 2mm). Much of the tephra is lapilli-sized, 2- 64 mm. This is not a powder - but, rather, a medium lapilli-sized pumiceous tephra.

2)"prodigious" has no direct translation in the Latin of the ancient texts.

de Architectura 5.12.1-2

1. de opportunitate autem portuum non est praetermittendum, sed quibus rationibus tueantur naves in iis ab tempestatibus explicandum. hi autem naturaliter si sint bene positi habeantque acroteria sive promuntoria procurrentia, ex quibus introrsus curvaturae sive versurae ex loci natura fuerint conformatae, maximas utilitates videntur habere. circum enim porticus sive navalia sunt facienda sive ex porticibus aditus ad emporia, turresque ex utraque parte conlocandae, ex quibus catenae traduci per machinas possint.

2. Sin autem non naturalem locum neque idoneum ad tuendas ab tempestatibus naves habuerimus, ita videtur esse faciendum uti si nullum flumen in his locis inpedierit sed erit ex una parte statio, tunc ex altera parte structuris sive aggeribus expediantur progressus. et ita conformandae portuum conclusiones. eae autem structurae quae in aqua sunt futurae, videntur sic esse faciendae uti portetur pulvis a regionibus quae sunt a Cumis continuatae ad promuntorium Minervae, isque misceatur uti in mortario duo ad unum respondeant.

de Architectura 2.6.1-2

1. Est etiam genus pulveris quod efficit naturaliter res admirandas. nascitur in regionibus Baianis et in agris municipiorum quae sunt circa Vesuvium montem. quod commixtum cum calce et caemento non modo ceteris aedificiis praestat firmitatem, sed etiam moles cum struuntur in mari, sub aqua solidescunt. hoc autem fieri hac ratione videtur quod sub his montibus et terrae ferventes sunt et fontes crebri, qui non essent, si non in imo haberent aut e sulphure aut alumine aut bitumine ardentes maximos ignes. igitur penitus ignis et flammae vapor per intervenia permanans et ardens efficit levem eam terram, et ibi qui nascitur tofus turgens est sine liquore. ergo cum tres res consimili ratione ignis vehementia formatae in unam pervenerint mixtionem, repente recepto liquore una cohaerescunt et celeriter umore duratae solidantur, neque eas fluctus neque vis aquae potest dissolvere.

2. ardores autem esse in his locis etiam haec res potest indicare quod in montibus Cumanorum Baianis sunt loca sudationibus excavata, in quibus vapor fervidus ab imo nascens ignis vehementia perforat eam terram per eamque manando in his locis oritur et ita sudationum egregias efficit utilitates. non minus etiam memorantur antiquitus crevisse ardores et abundavisse sub Vesuvio monte et inde evoluisse circa agros flammam. ideoque tunc quae spongia sive pumex Pompeianus vocatur excocto ex alio genere lapidis in hanc redacta esse videtur generis qualitatem.
