# Supplementary material for: Vitruvian binders in Venice: First evidence of Phlegraean pozzolans in an underwater Roman construction in the Venice Lagoon
Source: PLoS One. 2024 Nov 22;19(11):e0313917. doi: 10.1371/journal.pone.0313917 (PMC11584134; doi:10.1371/journal.pone.0313917)
Supplement: Attachment — Submitted filename: PONE-D-23-36360_R1_reviewer.pdf [file pone.0313917.s012.pdf]

PLOS ONE

Vitruvian binders in Venice: first evidence of Phlegraean pozzolans in an underwater Roman construction in the Venice Lagoon

--Manuscript Draft--

**Manuscript Number:**	PONE-D-23-36360R1
**Article Type:**	Research Article
**Full Title:**	Vitruvian binders in Venice: first evidence of Phlegraean pozzolans in an underwater Roman construction in the Venice Lagoon
**Short Title:**	Phlegraean pozzolans in mortars from an underwater Roman construction in the Venice Lagoon
**Corresponding Author:**	Simone Dilaria<br>University of Padova: Universita degli Studi di Padova<br>Padova, ITALY
**Keywords:**	Phlegraean pozzolans; Roman mortars; underwater archaeology; LA-ICP-MS; QPA-XRPD; M-S-H; Venice Lagoon
**Abstract:**	Four mortar samples were collected from a submerged Roman well-cistern (1st c. CE) in the Northern part of the Lagoon of Venice, recently investigated during underwater surveys promoted by the team of maritime archaeology of the University Ca' Foscari of Venice. Samples were preliminary described following a standardized protocol of analytical techniques, including Polarized Light Optical Microscopy (PLM), Quantitative Phase Analysis - X-Ray Powder Diffraction (QPA-XRPD) and Scanning Electron Microscopy (SEM) coupled with Energy-Dispersive X-Ray Spectroscopy (EDS). Archaeometric analyses allowed the samples to be identified as lime-based mortars enriched with ceramic fragments and sand-sized particles compatible with local alluvial deposits. Moreover, pyroclastic aggregates, incompatible with the local geology, were added to the mortars to enhance their hydraulic capabilities. Their provenance was determined geochemically by analysing, via SEM-EDS and Laser Ablation-Inductively Coupled Plasma-Mass Spectrometry (LA-ICP-MS), areas of apparently fresh volcanic glass, outlining the actual geochemistry of magma's pristine composition within the analysed clasts. The resulting fingerprints were then compared with the geochemical distribution of the pyroclastic products of the main Italian Plio-Quaternary magmatic districts. The lithological source of the analysed tephra appears to be petrochemically congruent with the Phlegraean Fields volcanic district. Provenance determination was complicated by the significant reaction processes the analysed pyroclastic clasts were object of, seriously affecting their geogenic chemical and petro-mineralogical features. In fact, the strongly alkaline anoxic underwater environment of the Venetian lagoon likely fostered the kinetics, as the matrices showed a relevant development of M-S-H hydrates replacing the pristine Ca-bearing phases of the binder. On the other hand, the carbonation of the lime was almost null. It seems that the homogeneous blend of local sands, ceramic fragments and imported volcanic rocks with brackish water promoted the development of pozzolanic and para-pozzolanic processes, increasing the durability and preservation of the analysed mortars over time. This evidence shows, once again, how the Vitruvian prescriptions on the utilization of Phlegraean pozzolans (Vitr., 5.12.2) were thoroughly grounded in the outcomes of ancient engineering knowledge, to enhance the physical and mechanical properties of seawater concretes.
**Order of Authors:**	Simone Dilaria
	Giulia Ricci
	Michele Secco
	Carlo Beltrame
	Elisa Costa
	Tommaso Giovanardi
	Jacopo Bonetto
	Gilberto Artioli

Powered by Editorial Manager® and ProduXion Manager® from Aries Systems Corporation

**Response to Reviewers:**	Journal Requirements<br>Figures<br>-Figure 1 was edited and rearranged, with subfigures edited from open access journals (credits reported according to the PLOS form). The new satellite sketch map on the right of figure 1 is taken from Landsat portal, as reported in the caption.<br>-Regarding figure 2 the copyholder of the main image (orthophoto) is included as an author of the paper (Carlo Beltrame). Do we have to obtain a permission from one of the authors itself to reproduce in Open Access one of his own photos he deliberately wants to publish in open access with this submission?<br><br>-Role of Funder statement<br>As requested, we amend for the role of funder statement and we ask to edit it with the following paragraph.<br><br>For the study design of the research, SD was financed by a grant from the “Exceptional Laboratory Practices in Cultural Heritage: Upgrading Infrastructure and Extending Research Perspectives of the Laboratory of Archaeometry”, co-financed by Greece and the European Union project under the auspices of the program “Competitiveness, Entrepreneurship and Innovation” NSRF 2014–2020, SD (https://ec.europa.eu/regional_policy/in-your-country/programmes/2014-2020/el/2014gr16m2op001_en). For the data collection and investigation of the well-cistern of Canale San Felice, CB was financed in 2023 by the PNRR project CHANGES – Cultural Heritage Active Innovation for Sustainable Society (project code: PE00000020; https://sites.google.com/uniroma1.it/changes/); For the analytical investigations on samples, SD was financed from the project “Trade and use of volcanic pozzolans in the Roman world. A natural material for the production of eco-sustainable concrete of antiquity” (Principal investigator: Simone Dilaria, BIRD 2023 of the Department of Cultural Heritage of the University of Padova, project code BIRD230232/23), TG was partially financed by Habits PRIN 2022 project, code 2022BC2Z5F. The research was implemented and funded within the scopes of the University of Padova under the World Class Research Infrastructures (WCRI) programme – SYCURI (Synergic Strategies for Culture Heritage at Risk).<br><br>Reviewer 1<br>•On lines 69 and 594 the pozzolanic material is wrongly termed "pulvis puteolana" rather than the correct Latin "pulvis puteolanus" seen elsewhere in the text.<br>- Edited and corrected<br>-<br>•It might be useful to some readers if the previously documented pattern of trade in this material was outlined: most of it is found on the coast of Lazio and Toscana and in a few large harbours elsewhere, such as Caesarea Palaestinae and Alexandria<br>- The introduction was rearranged including a specification of the port evidences in Italy and Eastern Mediterranean<br><br>Reviewer 2<br><br>Introduction<br>•The Introduction, unfortunately, contains many inaccuracies. Vitruvius did not use the term “pulvis puteolana” in de Architectura and never referred to Puteoli. There is also confusion over the use of chemical terms, such as “compound”, “liquid solutions (and “aliquot” in the Results); aerial lime-based mortars; and archaeological context. (Vitruvius wrote de Architectura in 30 BCE, for Octavian (not Augustus in 1st C CE)).<br><br>AND<br><br>•“Vitruvius” and “Vitruvian” appear many times in the manuscript yet the text of the actual passages from de Architectura are not given – nor is the relevance of these passages to the Venice lagoon mortars directly justified by the material analyses. Instead, there are oblique references that leave the reader confused as to the nature of a “prodigious powder”:<br>Line 35: “Vitruvian mention to the prodigious effects of pulvis puteolana used in seawater concrete (Vitr., 5.12.2-3)”

Powered by Editorial Manager® and ProduXion Manager® from Aries Systems Corporation

	AND<br><br>•Line 68: Both Vitruvius (Vitr. 2.6.1-2; 5.12.2) and Pliny (Pliny 16.202; 35.166) exalted the outstanding properties of the pulvis puteolana, referred to a prodigious powder outcropping around the modern town of Pozzuoli (Bay of Naples<br>Line 505: “exploitation of the “Vitruvian pulvis puteolana” in the ancient Mediterranean.<br>Line 537: “Vitruvian book, regarding the prodigious strength granted by pulvis puteolana once employed in the making of underwater concretes”<br><br>AND<br><br>•In fact, Vitruvius never used the term “pulvis puteolana”. He simply states “pulvis” (or dust). The Latin text of 2.6.1-2 and 5.12.2-3 appears at the end of this review.<br>Attribution of an origin from Puteoli should correctly go to Seneca, Q Nat, or Pliny, HN: p. 2, Building for Eternity, 2014, Oxbow Books.<br>In Latin, Vitruvius’ term for the pumiceous, poorly consolidated volcanic ash that crops out “in the vicinity of Baiae and the territory of the municipalities around Mount Vesuvius” in the northwest sector of the Gulf of Naples was pulvis, “powder” or “dust” (De arch 2.6.1). This term refers to its finer grain size distribution, as compared with the granular scoriaceous ash or excavated sands (harenae fossiciae) of the region around Rome. Vitruvius thus indicates that the powdery ash used in first century BC came from either the Flegrean Fields near Puteoli or the Somma- Vesuvius volcanic districts (Fig. 7.2). The term, Puteolanus pulvis, or “dust (or powder) from Puteoli,” occurs in two of the three passages by ancient authors that mention pulvis (Seneca, Q Nat 3.20.3; p. 26, Passage 14; Pliny, HN 16.202; pp. 26–27, Passage 15). In Pliny HN 35.167 (p. 27, Passage 16) the phrase is a pulvere Puteolano. Vitruvius does not attach a locative adjective, but simply states pulvis.<br><br>AND<br><br>•These issues create a highly problematic foundation for the manuscript. Substantial revision is needed to resolve the textural errors, add the relevant descriptions from de Architectura, and state explicitly how these are validated by the analytical results.<br><br>AND<br><br>•“Both Vitruvius (Vitr. 2.6.1-2; 5.12.2) and Pliny (Pliny 16.202; 35.166) exalted the outstanding properties of the pulvis puteolana, referred to a prodigious powder outcropping around the modern town of Pozzuoli (Bay of Naples).” Puteoli and “pulvis puteolana” is never referenced in de Architectura. Also,<br>1)"exalted": definition involves a person and is not justified by the text passages: (of a person or their rank or status) placed at a high or powerful level; held in high regard; in a state of extreme happiness. Please revise.<br>2)"prodigious", "powder", "outcropping" : the definitions of these words are at odds with the actual behavior of the pyroclastic deposits. Without the text passages the reader will not have a context for this oblique description<br>3)“Preservation”: the activity or process of keeping something valued alive, intact, or free from damage or decay.<br><br>AND<br><br>•"prodigious" has no direct translation in the Latin of the ancient texts.<br>Although the Gulf of Naples tephra (and tuff) deposits have a prominent fine-ashed size fraction (de’Gennaro et al 1999, https://www.sciencedirect.com/science/article/abs/pii/S0377027399000402) they also contain a substantial coarse ash to lapilli sized fraction. Therefore, referring to these deposits as “powder” is misleading. Please use the correct volcanological terms, defining them where appropriate.<br><br>The revision of the manuscript carefully considered all aspects related to the Introduction and the specific Latin terminology. The introduction underwent a comprehensive restructuring, with particular attention to detail, resulting in an expanded paragraph. In the initial version of the manuscript, these elements received only brief treatment, despite their thorough exploration in a prior study that focused

more extensively on the literary components (Dilaria, S., Secco, M., Ghiotto, A.R. et al. Early exploitation of Neapolitan pozzolan (pulvis puteolana) in the Roman theatre of Aquileia, Northern Italy. Sci Rep 13, 4110 (2023). https://doi.org/10.1038/s41598-023-30692-y). Nonetheless, we found it necessary to delve deeper into this discussion, incorporating all reviewer feedback and introducing additional literary references to ensure the coherence and integrity of the text.
Moreover, terms like "prodigious" and "exalted" have been avoided in the revised draft of the introduction. Additionally, as pointed out in various circumstances, the grain size of the volcanic deposits, both in the samples under examination and generally, has been modified from "ash"/"powder" to "medium lapilli-sized pumiceous tephra."
References to "pulvis" have been retained only in connection with Vitruvian mentions. We believe that this term could indeed align with the construction knowledge of a Roman builder who used the term "powder" to describe a loosely cohesive material that could easily be reduced to dust, either naturally or through rapid mechanical grinding. Therefore, we preferred to maintain this ambivalence between Vitruvian "pulvis" and the documented "medium lapilli" size in the samples.

Kinetics

•this discussion is based on only four EDS point analyses in low magnification images (Figure 6 b2, c2, c3, d1) and no direct information regarding fluid compositions in the lagoon. To accurately describe “kinetics of pozzolanic and para-pozzolanic reaction processes” more data is needed, such as high resolution SEM-EDS maps of relevant microstructures, high magnification images of the phase morphologies, and a potential resolution of the AFm phases in X-ray diffraction analyses, as hydrotalcite, for example.

AND

•Section 4.2 has a lengthy discussion of magnesium-enriched phases in the binding matrix. This is of interest, yet there seem to be only four EDS point analyses in low magnification images related to this process (Figure 6). The XRD analyses of Figure 4 state AFm phases, but these are not identified separately, e.g. as hydrotalcite. More data is needed – for example, high resolution SEM-EDS maps of instructive microstructural environments distinguishing pozzolanic reaction processes from subsequent authigenic processes, occurring possibly after the full consumption of calcium-hydroxide. In its current form, this hypothesis remains speculative.

AND

•“The backscattered electron images, as presented in Figure 6, reveal that most of the pozzolanic aggregates are profoundly reacted with the binder.”
This sentence is very confusing. Which are the pozzolanic aggregates? ... only the pumice? What data justifies "pozzolanic" reaction? Did the pozzolanic products then react with the binding matrix? Please separate out the concepts and describe them one by one after first presenting the analytical data.
Each clast type should be identified in the caption for each panel (and in the graphic as well, to assist the reader). Please also add a label to each EDS analysis panel, stating the phase from which the compositional analysis is derived.

In the revision of the manuscript, more detailed information on the crystal structures of AFm phases determined by XRPD analyses has been reported. Furthermore, the microstructural and microchemical characteristics of the pozzolanic reaction products have been better described through more detailed sentences and a new supporting figure (Figure 7). A dedicated sentence on the type of pozzolanic aggregates interested by pozzolanic interaction phenomena has been added.

Cistern diagram

•Section 5.1 is difficult to follow since there are no diagrams outlining the well-cistern structure (possibly shown in Fig. S1?)and relationships with changing water level from ancient to modern time.

Powered by Editorial Manager® and ProduXion Manager® from Aries Systems Corporation

A new subfigure (fig 1, c) reporting the original reconstruction of the well-cistern reporting the height of local water level in Roman times compared with the situation at present was added in the edited draft.

Other aspects
•Results presented in the Introduction should be moved to the Conclusions section
These sections were rearranged and the draft reorganized according to the suggestions provided by the Reviewer, also considering the detailed comments to the draft in the PDF file.

• statements questioning the validity of the LA-ICP-MS analyses of “unreacted glass” should be fully resolved.
As already stated, “This technique has been already adopted as a rapid and efficient tool for a preliminary assessment of volcanic pozzolans’ geochemistry included in ancient mortar-based materials” as reported for example in Marra et al. 2013; Marra et al. 2016; D’Ambrosio et al. 2015.

•Abstract. This statement, “significant reaction processes the analysed pozzolans were object of, seriously affecting their geogenic chemical and petro-mineralogical features”, calls into question preceding sentence, a "conclusion", "pyroclastic aggregates from the Phlegraean fields (pulvis puteolana) were added to the mortars". Please revise and clarify.
First, present the analytical data, Second, temper the conclusion: for example, "even so, the lithological origin of the tephra seems to the Campi Flegrei volcanic district."

The abstract was rearranged according to the suggestions and reordered presenting in order the analytical data and tempering the conclusions

•“perfect” and “perfectly” appear 6 times in the manuscript, with no justification for what “perfect” actually means: “having all the required or desirable elements, qualities, or characteristics; as good as it is possible to be; without fault, faultless, flawless.”
“Perfect” is a qualitative assessment – not a scientific descriptor.
The mortars are not flawless materials, please use a different descriptor. Neither is the preservation “perfect”, since there has apparently been much “decay” of the components of the original mortar fabric. Jackson et al. use the term “beneficial corrosion.”

As rightly pointed out by the reviewer, these qualitative adjectives (e.g., "perfect," "peculiar," etc.) are misleading in the text and not suitable for discussing analytical data. They have been entirely removed in the revised draft.

•This is not correct. "Aerial lime-based mortars" develop their binding characteristics through carbonation. That process is antithetical to mortars with pozzolans that develop mainly silicate binding components.
Do you mean lime-based mortars in sub-aerial construction? or architectural applications? Revision is needed. See:
https://www.researchgate.net/publication/358625402_AERIAL_LIME_MORTARS_AN_INTRODUCTION_AND_A_BRI EF_DISCUSSION

As rightly pointed out by the reviewer, the original phrase was misleading, as “aerial” specification assumes that a mortar had completed the carbonation process, whereas we do not intend to discuss the physical and mechanical improvements of mortars that have undergone complete carbonation. Therefore, the misleading term "Aerial" has been removed from the sentence, which has been modified to "lime-based mortars," without delving into the details of lime setting processes.

•The term ”volcanic pozzolans” adds more confusion. It attributes reactive capabilities to these particles that have not been validated through analyses. These appear to be pumice fragments, possibly with accretionary ash rims. (The astute reader would also wonder why the ceramic particles have no pozzolanic attributes.)

Powered by Editorial Manager® and ProduXion Manager® from Aries Systems Corporation

	Edited in the revised draft according to the useful suggestion. In the revised manuscript volcanic pozzolans was substitute in most of the cases with the actual material under study (i.e. Pumice, pumiceous tephra, weakly lithified tuffs) in particular when describing the results of the analytical investigations. We maintained the reference to “pozzolanic materials” or “volcanic pozzolans” in general only in the discussion and conclusions sections as they refer to general consideration of these kinds of materials.<br><br>•The hypothesis stating the relevance of the materials to the descriptions in de Architectura is not directly tested and validated.<br><br>The revised draft now includes a more thorough comparison between the materials under study and the Vitruvian source, in particular in the discussion and conclusions. Further investigation into the disparities between Vitruvius's description of Neapolitan "pulvis" usage and its actual implementation in construction has been conducted in the discussion and conclusions. This exploration showcases the strong connection with traditional Roman construction practices and explores alternative applications of "pulvis" in coating contexts. However, the primary focus remains on highlighting the reactive properties of volcanic pozzolanic material, particularly its alkaline reaction with water, whether utilized in mixes or upon contact post-placement. This emphasis aligns seamlessly with the empirical understanding presented in De architectura regarding mortar hardening and durability in submerged environments.<br><br>•The photomicrographs and micrographs in the manuscript show many clasts greater than ash-sized (or sand- sized, < 2mm). Much of the tephra is lapilli-sized, 2- 64 mm. This is not a powder - but, rather, a medium lapilli- sized pumiceous tephra.<br><br>Edited throughout the revised manuscript according to this right observation.<br><br>•This is highly problematic. As previously noted, Vitruvius never used the term "pulvis puteolana". Furthermore, there is no "prodigious strength" – compressive strength measurements are uniformly low in the ancient concretes and their modern replicas, < 9 MPa (Oleson et al. 2006, DOI: 10.1017/S1047759400006255).<br>The sentence addressing this aspect in the discussion section has been refined to highlight the moderate durability of underwater concretes incorporating Neapolitan pozzolans when compared to traditional ancient mortars, albeit still falling short of the mechanical strength exhibited by modern concretes.
**Additional Information:**	
**Question**	**Response**
**Financial Disclosure**<br><br>Enter a financial disclosure statement that describes the sources of funding for the work included in this submission. Review the submission guidelines for detailed requirements. View published research articles from *PLOS ONE* for specific examples.<br><br>This statement is required for submission and **will appear in the published article** if the submission is accepted. Please make sure it is accurate.	Yes

**Funded studies**<br>Enter a statement with the following details:<br>• Initials of the authors who received each award<br>• Grant numbers awarded to each author<br>• The full name of each funder<br>• URL of each funder website<br>• Did the sponsors or funders play any role in the study design, data collection and analysis, decision to publish, or preparation of the manuscript?<br><br>Did you receive funding for this work?	
Please add funding details.<br>as follow-up to "**Financial Disclosure**<br><br>Enter a financial disclosure statement that describes the sources of funding for the work included in this submission. Review the submission guidelines for detailed requirements. View published research articles from *PLOS ONE* for specific examples.<br><br>This statement is required for submission and **will appear in the published article** if the submission is accepted. Please make sure it is accurate.<br><br>**Funded studies**<br>Enter a statement with the following details:<br>• Initials of the authors who received each award<br>• Grant numbers awarded to each author<br>• The full name of each funder<br>• URL of each funder website<br>• Did the sponsors or funders play any role in the study design, data collection and analysis, decision to publish, or preparation of the manuscript?<br><br>Did you receive funding for this work?"	This project was partially implemented within the scope of the “Exceptional Laboratory Practices in Cultural Heritage: Upgrading Infrastructure and Extending Research Perspectives of the Laboratory of Archaeometry”, co-financed by Greece and the European Union project under the auspices of the program “Competitiveness, Entrepreneurship and Innovation” NSRF 2014–2020 (https://ec.europa.eu/regional_policy/in-your-country/programmes/2014-2020/el/2014gr16m2op001_en). The investigation of the well-cistern of Canale San Felice in 2023 was financed by the PNRR project CHANGES - Cultural Heritage Active Innovation for Sustainable Society (project code: PE00000020; https://sites.google.com/uniroma1.it/changes/). The analyses on materials were financed from the project “Trade and use of volcanic pozzolans in the Roman world. A natural material for the production of eco-sustainable concrete of antiquity” (Principal investigator: Simone Dilaria, BIRD 2023 of the Department of Cultural Heritage of the University of Padova, project code BIRD230232/23). This work is funded by the University of Padova under the World Class Research Infrastructures (WCRI) programme - SYCURI (Synergic Strategies for Culture Heritage at Risk).
Please select the country of your main research funder (please select carefully as in some cases this is used in fee calculation).	ITALY - IT

as follow-up to "**Financial Disclosure**<br><br>Enter a financial disclosure statement that describes the sources of funding for the work included in this submission. Review the submission guidelines for detailed requirements. View published research articles from *PLOS ONE* for specific examples.<br><br>This statement is required for submission and **will appear in the published article** if the submission is accepted. Please make sure it is accurate.<br><br>**Funded studies**<br>Enter a statement with the following details:<br>• Initials of the authors who received each award<br>• Grant numbers awarded to each author<br>• The full name of each funder<br>• URL of each funder website<br>• Did the sponsors or funders play any role in the study design, data collection and analysis, decision to publish, or preparation of the manuscript?<br><br>Did you receive funding for this work?"	
**Competing Interests**<br><br>Use the instructions below to enter a competing interest statement for this submission. On behalf of all authors, disclose any competing interests that could be perceived to bias this work—acknowledging all financial support and any other relevant financial or non-financial competing interests.<br><br>This statement is required for submission and **will appear in the published article** if the submission is accepted. Please make sure it is accurate and that any funding sources listed in your Funding Information later in the submission form are also declared in your Financial Disclosure statement.	The authors have declared that no competing interests exist.

View published research articles from *PLOS ONE* for specific examples.<br><br>**NO authors have competing interests**<br><br>Enter: *The authors have declared that no competing interests exist.*<br><br>**Authors with competing interests**<br><br>Enter competing interest details beginning with this statement:<br><br>*I have read the journal's policy and the authors of this manuscript have the following competing interests: [insert competing interests here]*<br><br>* typeset	
**Ethics Statement**<br><br>Enter an ethics statement for this submission. This statement is required if the study involved:<br><br>• Human participants<br>• Human specimens or tissue<br>• Vertebrate animals or cephalopods<br>• Vertebrate embryos or tissues<br>• Field research<br><br>Write "N/A" if the submission does not require an ethics statement.<br><br>General guidance is provided below. Consult the submission guidelines for detailed instructions. **Make sure that all information entered here is included in the Methods section of the manuscript.**	N/A

**Format for specific study types**<br><br>**Human Subject Research (involving human participants and/or tissue)**<br>• Give the name of the institutional review board or ethics committee that approved the study<br>• Include the approval number and/or a statement indicating approval of this research<br>• Indicate the form of consent obtained (written/oral) or the reason that consent was not obtained (e.g. the data were analyzed anonymously)<br><br>**Animal Research (involving vertebrate animals, embryos or tissues)**<br>• Provide the name of the Institutional Animal Care and Use Committee (IACUC) or other relevant ethics board that reviewed the study protocol, and indicate whether they approved this research or granted a formal waiver of ethical approval<br>• Include an approval number if one was obtained<br>• If the study involved *non-human primates*, add *additional details* about animal welfare and steps taken to ameliorate suffering<br>• If anesthesia, euthanasia, or any kind of animal sacrifice is part of the study, include briefly which substances and/or methods were applied<br><br>**Field Research**<br><br>Include the following details if this study involves the collection of plant, animal, or other materials from a natural setting:<br>• Field permit number<br>• Name of the institution or relevant body that granted permission	
**Data Availability**<br><br>Authors are required to make all data underlying the findings described fully available, without restriction, and from the time of publication. PLOS allows rare exceptions to address legal and ethical concerns. See the PLOS Data Policy and FAQ for detailed information.	Yes - all data are fully available without restriction

A Data Availability Statement describing where the data can be found is required at submission. Your answers to this question constitute the Data Availability Statement and **will be published in the article**, if accepted.<br><br>**Important:** Stating 'data available on request from the author' is not sufficient. If your data are only available upon request, select 'No' for the first question and explain your exceptional situation in the text box.<br><br>Do the authors confirm that all data underlying the findings described in their manuscript are fully available without restriction?	
**Describe where the data may be found in full sentences. If you are copying our sample text, replace any instances of XXX with the appropriate details.**<br><br>• If the data are **held or will be held in a public repository**, include URLs, accession numbers or DOIs. If this information will only be available after acceptance, indicate this by ticking the box below. For example: *All XXX files are available from the XXX database (accession number(s) XXX, XXX.*).<br>• If the data are all contained **within the manuscript and/or Supporting Information files**, enter the following: *All relevant data are within the manuscript and its Supporting Information files.*<br>• If neither of these applies but you are able to provide **details of access elsewhere**, with or without limitations, please do so. For example:<br><br>*Data cannot be shared publicly because of [XXX]. Data are available from the XXX Institutional Data Access / Ethics Committee (contact via XXX) for researchers who meet the criteria for access to confidential data.*<br><br>*The data underlying the results presented in the study are available from (include the name of the third party*	All relevant data are within the manuscript and its Supporting Information files.

and contact information or URL).<br>• This text is appropriate if the data are owned by a third party and authors do not have permission to share the data.<br><br>* typeset	
Additional data availability information:	Tick here if the URLs/accession numbers/DOIs will be available only after acceptance of the manuscript for publication so that we can ensure their inclusion before publication.

Manuscript
Click here to access/download;Manuscript;Vitruvian binders in Venice_manuscript_rev1_Clean Copy.docx

Vitruvian binders in Venice: first evidence of Phlegraean pozzolans in an underwater Roman construction in the Venice Lagoon

Simone Dilaria[1,2,*], Giulia Ricci[2,3], Michele Secco[1,2], Carlo Beltrame[4], Elisa Costa[4], Tommaso Giovanardi[5], Jacopo Bonetto[1], Gilberto Artioli[2,3]

[1] Department of Cultural Heritage (DBC), University of Padova, Padua, Italy.

[2] Inter-Departmental Research Centre for the Study of Cement Materials and Hydraulic Binders (CIRCe), University of Padova, Padua, Italy.

[3] Department of Geosciences, University of Padova, Padua, Italy.

[4] Department of Humanities, Ca' Foscari University Venice, Venice, Italy

[5] Department of Chemical and Geological Sciences, University of Modena and Reggio Emilia, Modena, Italy

[*] Corresponding author (simone.dilaria@unipd.it)

Abstract

Four mortar samples were collected from a submerged Roman well-cistern (1st c. CE) in the Northern part of the Lagoon of Venice, recently investigated during underwater surveys promoted by the team of maritime archaeology of the University Ca' Foscari of Venice. Samples were preliminary described following a standardized protocol of analytical techniques, including Polarized Light Optical Microscopy (PLM), Quantitative Phase Analysis - X-Ray Powder Diffraction (QPA-XRPD) and Scanning Electron Microscopy (SEM) coupled with Energy-Dispersive X-Ray Spectroscopy (EDS). Archaeometric analyses allowed the samples to be identified as lime-based mortars enriched with ceramic fragments and sand-sized particles compatible with local alluvial deposits. Moreover, pyroclastic aggregates, incompatible with the local geology, were added to the mortars to enhance their hydraulic capabilities. Their provenance was determined geochemically by analysing, via SEM-EDS and Laser Ablation-Inductively Coupled Plasma-Mass Spectrometry (LA-ICP-MS), areas of apparently fresh volcanic glass, outlining the actual geochemistry of magma's pristine composition within the analysed clasts. The resulting

fingerprints were then compared with the geochemical distribution of the pyroclastic products of the main Italian Plio-Quaternary magmatic districts. The lithological source of the analysed tephra appears to be petrochemically congruent with the Phlegraean Fields volcanic district. Provenance determination was complicated by the significant reaction processes the analysed pyroclastic clasts were object of, seriously affecting their geogenic chemical and petro-mineralogical features. In fact, the strongly alkaline anoxic underwater environment of the Venetian lagoon likely fostered the kinetics, as the matrices showed a relevant development of M-S-H hydrates replacing the pristine Ca-bearing phases of the binder. On the other hand, the carbonation of the lime was almost null. It seems that the homogeneous blend of local sands, ceramic fragments and imported volcanic rocks with brackish water promoted the development of pozzolanic and para-pozzolanic processes, increasing the durability and preservation of the analysed mortars over time. This evidence shows, once again, how the Vitruvian prescriptions on the utilization of Phlegraean pozzolans (Vitr., 5.12.2) were thoroughly grounded in the outcomes of ancient engineering knowledge, to enhance the physical and mechanical properties of seawater concretes.

Introduction

The technological expertise achieved by humankind in the formulation of binding products from Antiquity throughout time represents one of the most intriguing aspects in contemporary research on ancient mortar-based materials [1–4]. A specific interest was devoted by scholars to the parametrization of the physical-mechanical properties of ancient recipes, in order to unveil the secrets of the relevant durability and longevity of such composite construction materials.

In this framework, the use of certain aggregates, such as crushed ceramics, highly-amorphous volcanic rocks, organic ashes, metal slags or calcined clays [5] represents one of the most brilliant achievements of ancient craftsmanship to improve the cohesive behaviour and waterproofing capabilities of lime-based mortars [6–11]. Under the term “pozzolanic reaction” we usually refer to the full set of chemical processes occurring when such materials – usually labelled as pozzolanic aggregates – are mixed with calcium hydroxide (portlandite) in pastes. During the reaction, the silica and alumina compounds

present in these reactive aggregates react with the portlandite. This results in the formation of an array of anthropogenic phases detected in ancient mortars [12], having very variable stoichiometries, ranging from pure calcium silicate hydrates (C-S-H) to calcium aluminate hydrates (C-A-H) or complex C-A-S-H phases at increased Al activity. Such newformed phases are the main binding agents responsible for the strength, durability and waterproofing capabilities of the resulting composite materials [13–18].

Nevertheless, the environmental conditions in which mortar-based mixtures are embedded also play an essential role in the development of reaction processes. As thoroughly investigated by [7], the latent reactivity of pozzolanic aggregates appears to be favored in saltwater environments, since the alkaline and anoxic conditions promote the reaction, whereas lime carbonation is minimized. Moreover, under certain conditions of alkali, magnesium and chlorine activity, the hydraulic reaction can deviate from the typical lime-silica-alumina ternary diagram, favoring the precipitation of other types of para-pozzolanic hydrate products, based on the interaction of magnesium with silica and, sometimes, also with alumina. These processes usually lead to the formation of M-S-H/M-A-S-H phases (where M stands for MgO in cement chemistry notation) with a structurally disordered phyllosilicate-like structure [19–22], whose formation in ancient mortars is now extensively recognized [12,23–28].

Ancient builders formalized their empirical knowledge on the reactions occurring in mortar-based materials enhanced with reactive aggregates in underwater environments. Vitruvius, in his *De Architectura* books on ancient architecture and building materials, made reference to a particular powder (*pulvis*) outcropping between *Baiae* in the Gulf of Naples and the territories surrounding Mount Vesuvius (*nascitur in regionibus Baianis et in agris municipiorum quae sunt circa Vesuvium montem*, Vitr. *De Arch*. 2.6.1). He observed that, once mixed with hydrated lime, such *pulvis* confers remarkable properties (*res admirandas*) to lime-based mortars, as underlined by the Latin author. He firstly highlighted its unique capability to enhance the cohesion of structural concretes, as observed in [29], (*commixtum [pulvis] cum calce et caemento non modo ceteris aedificiis praestat firmitatem*, Vitr. *De Arch*. 2.6.1). Furthermore (*sed etiam*), this material was recommended for the construction of concrete piers in harbour infrastructures, as it enables the setting of concrete even in underwater environments [30,31] (*moles cum struuntur in mari, sub aqua solidescunt*, Vitr. *De Arch*. 2.6.1). In another part of his treaty, the Roman author reaffirmed the outcropping area of the powder within an area ranging from

the territory of *Cumae* to the north, to the *promontorium Minervae* – to be identified with the Mounts Lattari in the Sorrentine peninsula [32]– to the south (*pulvis a regionibus quae sunt a Cumis continuatae ad promuntorium Minervae*, Vitr. *De Arch*. 5.12.2). Modern geological research suggests a correspondence between the Vitruvian *pulvis* and medium-sized pumiceous tephra and weakly-lithified tuff outcropping in the Neapolitan volcanic district and pertaining both to the last Phlegraean eruptions (Neapolitan Yellow Tuff and post-Neapolitan Yellow Tuff) and the pre-79 CE eruptive events of Mount Somma-Vesuvius [33].

After Vitruvius, references to the "*pulvis*" become indeed recurrent in ancient treaties. Pliny in his *Historia Naturalis* (published one or two years before the 79 CE eruption of Somma-Vesuvius), and Seneca in *Naturales quaestiones*, quoted the Vitruvian definition, adding the adjective *puteolanus* to the *pulvis*, thus identifying the main source deposits in the nearness of the ancient town of Puteoli, nowadays Pozzuoli in the Bay of Naples (*pulverem appellatam in Puteolanis collibus*, Plin. *NH* 35.166; *in ipsa turribus Puteolis e pulvere exaedificatis*, Plin. *NH* 16.202; *Quemadmodum Puteolanus pulvis*, Sen. *Q. Nat*. 3.20.3). Given the nearly century-long gap between the lives of Vitruvius and those of Pliny and Seneca, an hypothesis that has not yet evaluated by scholars is whether this specific reference to the sourcing area limited in the nearness of the *ager Puteolanus* could also indicate a centralization of the quarry sites for the material around the Phlegraean district, in the Gulf of Naples, rather than in the Somma-Vesuvius volcanic unit. Indeed, from the second half of the 1st c. BCE, the Phlegraean territory saw significant infrastructure development, implementing some of the Roman Empire's primary commercial and military harbours, such as those at Baia, Puteoli and Miseno [34,35]. These ports played a crucial role in facilitating the spread of the Neapolitan volcanic pozzolans for concrete constructions throughout the Mediterranean regions of the Roman Empire during the next two centuries. In fact, in agreement with the literary sources, from the second half of the 1st c. BCE, around the time when Vitruvius wrote the *De Architectura* for Octavian (~ 30 BCE), the Neapolitan *pulvis* started being traded out of the region, monopolizing the markets in the following decades as the pivotal material to produce durable hydraulic mortar-based materials. Recent evidence confirms the widespread use of "pulvis puteolanus" for the realization of underwater harbor piers, whose utilization was documented in Late Republican structures from both Tyrrhenian and Adriatic sea [33]. Since the end of the 1st c. BCE, evidence of Phlegraean

pozzolans utilization has been recorded in some of the major Roman harbors of the Eastern Mediterranean, such as that of Caesarea Marittima, built by Herod the Great around by the end of the 1st c. BCE, and that of Alexandria in Egypt [33,36,37]. Furthermore, evidence of utilization of the *pulvis puteolanus* for the structural reinforcement of masonry concretes for on-land buildings were documented in Roman constructions in the sites of the Gulf of Naples at least since the 2nd c. BCE [38–41]. The material was also traded and used in the Provinces of the Empire for the realization of concrete masonries and foundations of on-land urban public monuments, as indicated by recent findings from Aquileia in Northern Italy [42], Sardinia [29] and North Africa [43].

During the Late Imperial Age, mentions to the *pulvis puteolanus* were reported by Fulgenzio (*De Aet Mund*. 3.138) in the 5th c. CE, and by Isidorus of Seville (*Etym*. 16.1.8) in the 7th c. CE [44]. The frequent appearance of the term in ancient sources underlines its notoriety as building material in Roman times, persisting until the early Middle Ages. However, archaeological evidence of the trading of *pulvis puteolanus* after the first decades of the 4th c. CE (or even after the first Tetrarchy) are currently lacking. Proof of utilisation of imported Phlegraean pumices, used as pozzolanic and light-weighting materials on concrete vaults, has been found in the Diocletian Baths in Rome [45] and in the Great Baths of Aquileia [46], recently dated to the Diocletian period [47]. From the second half of the 4th c. CE, the economic and commercial crisis that affected the Empire likely led to a substantial drop in the exportation of Neapolitan volcanic pozzolans in the Mediterranean.

In this research, a new evidence of mortars enriched with volcanic tephra in submerged environments is reported. Samples were collected from a Roman well-cistern nowadays preserved in the northern Venice Lagoon, recently investigated by the team of maritime Archaeology of the University Ca' Foscari Venice, led by C. Beltrame [48]. This is a peculiar system for collecting and storing freshwater, an ancestor of the Venetian-style well ("Pozzo alla Veneziana") developed in Venice during the Medieval and Modern Ages [49], but originating in Roman times.

The archaeological context and mortar sampling

The site of the well-cistern is nowadays situated along the San Felice Channel in the northern part of the Venice Lagoon, a shallow submerged area characterized by salt marshes, tidal flats and tidal channels (**Fig 1a-b**). According to the most recent geomorphological reconstructions, the 'well-cistern' was originally positioned much closer to the coastal edge between the lagoon and the Adriatic Sea [50]. Currently, it lies at a depth ranging from -4.5 to -3.0 m MSL, rising around 1.5 m above the bottom of the channel. Nevertheless, on the basis of the photogrammetric survey and the quoted DEM of the structure (Digital Elevation Model), the depth of the structure suggests that it was partially placed underwater also in Roman times. In fact, even considering that the Roman relative sea level (RSL) in this part of the northern lagoon was about 2.15 m lower [51], the base of the well-cistern was surely positioned at a depth of around – 2.0 m MSL even in ancient times [48] (**Fig 1c**).

The historical framework and constructive technique of the structure was already accurately described in [48]: this is a flat quasi-squared box-like construction of around 6.7 × 7.7 m and entirely made, in both masonries and floors, of local bricks, measuring around 43.5–46 × 29.5–29 × 6 cm, which is the typical size of the *sesquipedali* of the Po Valley [52,53]. Due to its shape, in previous studies (for example [54]) the structure was interpreted as a “defensive tower” (Torrione) dating to the Roman Imperial period. In the summer 2020, after the underwater surveys conducted by the team of Ca’ Foscari University assisted by Venice's Idra company, the structure was reinterpreted as a Roman well-cistern. This consists of a circular well shaft lined with bricks or stones, included in a squared masonry structure. The space between the well and the squared structure is then filled with river sand, employed for filtering and draining clean rain water to the bottom, where it is tapped by the well (see **Fig 1c**).

The new interpretation of the San Felice structure as a well-cistern relies on several indicators. These include the preliminary identification of the use of waterproofing *cocciopesto* mortar renders, the assessment of the structure's base depth in relation to the relative sea level, the absence of groundwork poles in the soft lagoon mud – that are recurrent for building foundations of Roman period – and the strong similarities with the much better preserved well-cistern of Ca’ Ballarin, located along the same

channel not far from this site [48]. Various other pieces of evidence of such structures dated to Roman Times are present in the North-Eastern Adriatic territory (i.e. Aquileia and Musile di Piave, **S1 Fig**).

The dating of the structure to the 1st c. CE, or little later, was determined on the basis of associated pottery finds [48].

To characterize properties and composition of the hydraulic mortars employed in the structures, four mortar samples, labelled TSF (Torrione San Felice), were collected from coating renders and masonry joints of the structure during two underwater campaigns in 2020 and 2023 (**Fig 2).** All necessary permits were obtained for the described study, which complied with all relevant regulations. The sampling activity and the underwater archaeological survey in the Lio Piccolo site was authorized by the Soprintendenza Archeologia, Belle Arti e Paesaggio per l'area metropolitana di Venezia e le province di Belluno, Padova e Treviso. Samples were collected and stored in the laboratories of the Department of Geosciences of the University of Padova.

Fig 1. The site of Torrione San Felice in the Lagoon of Venice. (a) Reconstruction of the Lagoon of Venice with its main morphological features: salt marshes, tidal flats, and tidal channels (modified from [55] for illustrative purposes only); (b) Satellite image (taken from Landsat Image Gallery - https://landsat.visibleearth.nasa.gov) of the area highlighted in dashed black line in sub-figure (a), with positioning of the site of the San Felice well-cistern; (g) graphical hypothetical reconstruction of the original conformation of the San Felice well-cistern and relative sea-level in Roman times and at present.

Fig 2. Orthophoto of the submerged San Felice well-cistern at the end of 2020 survey, with indication of the mortars under study and their sampling points.

Multi-analytical protocol

TSF samples collected from the structure were analysed according to a multi-analytical protocol designed for defining the raw materials employed in the compounds, the reaction phenomena occurring in submerged environment and, finally, the provenance of the volcanic tephra included in the mortars (**S1 Analytical methods**).

189 All samples were firstly subjected to a full petro-mineralogical characterization, through detailed
190 Polarized Light Optical Microscopy (PLM) investigations of mortars prepared on 30 μm thin sections.
191 The characterization was accomplished in accordance with the standard UNI 11176:2006 "Cultural
192 heritage - Petrographic description of a mortar" [56]. Furthermore, samples were mineralogically
193 investigated by Quantitative Phase Analysis - X-Ray Powder Diffraction (QPA-XRPD). Bulk samples were
194 analysed after the addition of 20 wt% zincite as internal standard. Subsequently, the XRPD profiles were
195 refined through the Rietveld method [57].
196 After this first screening, the most representative sample (TSF_T9) was subjected to further in-detail
197 investigations aimed at parametrizing the reaction phenomena and the provenance of the volcanic
198 tephra included in the material.
199 Scanning Electron Microscopy (SEM) coupled with Energy-Dispersive X-Ray Spectroscopy (EDS)
200 analyses on both binder matrices and aggregates' reaction rims were functional at determining the
201 chemical activity of the hydrate products occurring in the alkali-rich brackish-water environment of the
202 Lagoon of Venice [58]. Moreover, QPA-XRPD analysis on the sole binder fraction of the sample allowed a
203 mineralogical characterization of the reaction products within the material. Binder-concentrated
204 aliquots were separated from the bulk mortar following the wet separation procedure [59,60]. The
205 quantification of the mineralogical phases was determined by the addition to the separated-binder
206 fraction of 20 wt% zincite as internal standard. Then, the profiles were processed adopting the same
207 procedure previously defined for the bulk mortars.
208 The provenance of the volcanic tephra was determined adopting a geochemical approach. After a
209 detailed mineral-petrographic characterization under PLM, the investigation of the geochemistry of
210 pyroclastic clasts was performed according to the analytical protocol reported in [42]. Firstly, their
211 major elements' profiles were determined by analysing fresh unreacted areas of volcanic glass. The
212 average values and standard deviations were calculated based on 5 up to 10 SEM-EDS semi-quantitative
213 punctual analyses, performed on a 30 μm thin section (TSF_T9A) and on two 1 mm thick sections
214 (TSF_T9B and TSF_T9C), prepared in order to be investigated via LA-ICP-MS. The sections were polished
215 and carbon-coated before the analysis. LA-ICP-MS was performed in order to collect the trace element
216 profiles of a selection of juveniles already investigated by SEM-EDS. To avoid as much as possible the

contamination with non-geogenic chemical elements (i.e. pozzolanic hydrated phases), several clasts preserving portions of fresh volcanic glass fitting within the 55 μm laser ablation spot-size were selected for this part of the research. For each clast, 4 up to 9 punctual LA-ICP-MS analyses were acquired, and the average values and standard deviations were calculated and reported. Then, according to an already scientifically-established methodology [33,61–63], a selection of major and trace elements collected from the observed pozzolans was plotted in discriminant scatterplots in relation with the distribution of already-analysed geological samples related to the main Plio-Quaternary magmatic activities of the Italian peninsula. Moreover, by plotting in explorative scatterplots and spider-diagrams the chemical elements one-by-one, an updated pattern of elements was selected among REE (Rare Earth Elements) and HFSE (High Field Strength Elements) to discriminate among the differentiated magmatic activities occurred within the Neapolitan volcanoes. Finally, in order to check and verify the results inferred from scatterplot and spider-diagram distributions, we processed the geochemical data by discriminant analysis (DA). This is a multivariate statistical data reduction method frequently adopted to validate the classification of known data into categories or to assign unknown observations into such categories. This useful technique has been already applied in archaeology for assessing the provenance of artifacts of unknown origin by matching their descriptive variables with already classified markers [29,64–67].

Results

Characterization of raw materials

Through PLM investigations, the TSF samples were characterized as lime-based mortars enriched with ceramic fragments and dust (*cocciopesto*), mixed with sands and medium lapilli-sized pumiceous tephra in variable concentrations (**Tab 1**).

Sample TSF_T9, collected from the joint coating between one wall and the floor of the well-cistern (socle), has the highest content of volcanic tephra and ceramic fragments, easily detectable even at naked eye (**Fig 3, a**). This is followed by sample TSF_LN, where the concentration of volcanic tephra and ceramic fragments appear less abundant than the previous one (**Fig 3, b**). Finally, the last two samples, TSF_SS and SN, show a low amount of these components while the concentration of sand, comprising

244 angular monocrystalline quartz, micritic limestone and dolostones clasts, small granitoids and scattered
245 plagioclases and micas of the muscovite type (**Fig 3, c-d**), is higher [68]. From a minero-petrographic
246 point of view, their lithology is compatible with that of the local alluvial deposits, reworked in lagoon
247 environments, with intermediate composition between the sediments of the lagoon bottom, seashore,
248 and Piave and Brenta rivers [68].
249 QPA-XRPD analyses highlighted these differences through a significant shift in the concentration of the
250 phases correlated with the silicate fraction of the sand, such as quartz and plagioclases (**Fig 4** and **Tab**
251 **1**). Another distinguishing marker is represented by the abundance of clinopyroxene of the diopside
252 type, that is almost twice in TSF_T9 (11.4 wt%) in respect to the other samples. Its occurrence is related
253 to the ceramic inclusions and it demonstrates the firing of Ca-rich clays at high temperature (>850 °C)
254 [69].
255 Among the newformed pozzolanic phases, hydrated calcic aluminates of the C-A-H type (AFm phases)
256 [70] with crystal structures referable to those of calcium monocarboaluminate [71,72] were detected in
257 all the samples apart from TSF_T9. Its absence in the latter probably rely on the fact that most of Si and
258 Al ions did not react with Ca in a traditional Ca-based pozzolanic system, but with Mg, precipitating Mg-
259 rich hydrated compounds (M-S-H and M-A-S-H), as detailed in paragraph 4.2. In fact, broad low-angle
260 peaks ascribable to poorly crystalline M-S-H/M-A-S-H phases presenting a phyllosilicate-like structure
261 of turbostratically-disordered smectite clays [12,19,26,73,74] were detected in high concentration in
262 sample TSF_T9. Finally, the presence of pyrite, detected by XRPD in most of the samples, is to be ascribed
263 to post-depositional alteration phenomena of the materials, as a consequence of the action of sulfate-
264 reducing bacteria, which promoted the dissolution of oxides and hydroxides in sulfur-rich alkaline
265 aqueous solutions. The conditions for the proliferation of these bacteria are coherent with that of buried
266 contexts of anoxic lagoon-like environments, having abundant organic matter and poor water circulation
267 [75–77].
268 As already stated, sample TSF_T9 was selected for a thorough analytical characterization, being the
269 mortar with the highest concentration of pozzolanic aggregates and that showing the more complex
270 reaction kinetics of the pozzolanic aggregates with the binder.

Under PLM observation, the binder of TSF_T9 appears extremely zoned, with a structure varying from micritic to microsparitic and diffused areas exhibiting very low birefringence colours (**Fig 5, b**).

Lime lumps are few and scattered (**Fig 5, c**), demonstrating a good mixing of the binding component. The aggregate is mainly constituted of poorly sorted ceramic elements (**Fig 5, d**), with a grain-size distribution ranging from angular pluri-millimetric fragments to finely dispersed micrometric powder, less than 40 μm in size. The relevant milling probably contributed to the reactivity of the ceramic fraction [78,79].

Volcanic clasts constitute a slightly subordinate fraction of the aggregate in respect to the ceramic ones. They primarily consist in rounded-subrounded vesicular pumices with elongated or round pores and weak to compact density (**Fig 5, e**). Under specific conditions, the reaction with the binder is pervasive to the extent that pozzolans become barely discernible, appearing as *ghosts* fully reacted within the matrix. Two families of pumices were distinguished, based on the texture of their groundmass: completely aphyric (glassy) juveniles (**Fig 5, f-g**) fairly prevailing over feeble porphyritic ones (**Fig 5, h-i**), presenting sparse feldspars microlites and biotite occasionally filling the intraclasts. The phenocrysts' assemblage is constituted by large crystals of K-feldspar (sanidine) prevailing over plagioclases, biotite and – occasionally – clinopyroxenes. Accessory minerals are composed of opaque minerals (iron oxides) and apatite (**S2 Fig, a**). These juveniles are characterized by high porosity, deriving from the quick outgassing of magmatic volatiles in the course of explosive events [80]. Angular chips of weakly-lithified tuffs (**Fig 5, j** and **k**) constitute a subordinate fraction of the volcanic aggregates. They are rich in mineral inclusions of biotite, k-feldspars (sanidine/orthoclase) and plagioclases, clay minerals, iron oxides, opaque minerals, glass shards and fragmented pumices embedded in a cineritic matrix (**S2 Fig, b**). Finally, compact vitrophyric obsidians with scattered feldspars microlites (**Fig 5, l**) were occasionally observed together with dense porphyritic lavas with k-feldspars and clinopyroxene phenocrysts (**Fig 5, m**). It must be remarked that in the other samples from the well-cistern, only pumices were detected among the volcanic components.

From a minero-petrographic point of view, the volcanic clasts detected in TSF samples appear incompatible with the magmatism of the volcanic rocks of the Veneto Region exploited in Roman times, substantially constituted by the dense effusive lava domes, dikes and explosive breccias of the Euganean

Hills district [81] and compact basalts and basanites of the Berici Hills districts [82]. Therefore, this component of the aggregate is not local, and it was imported from other regions, whose geographic position was tracked in paragraph 4.3.

Opaque millimetric shards and ground microclasts of oxidized iron slags (**Fig 5, n**), chemically characterized via SEM-EDS microanalysis (**S2 Fig, c**), were detected uniquely in TSF_T9 sample, while they are absent in the others. They could act as tensile strengthening agents of the binding composite, and they were likely added only to this sample to enhance the mechanical behaviour of the compound [83], according to a practice already attested in ancient Greek and Roman times [84,85]. Their abundance suggests an intentional reuse of discarded processing debris of metalwork that probably took place in the nearness of the site, but on which there are no pieces of evidence in the archaeological record.

Finally, fragments of carbonate rocks were identified and they consist in sub-millimetric to pluri-millimetric angular or sub-rounded chips of crystalline limestones (**Fig 5, o**), metamorphosed white marbles (**Fig 5, p**), and occasional altered dolostones/dolomitic limestones (**Fig 5, q**), indicating de-dolomitization processes [86]. Furthermore, apart from monomineralic quartz/carbonate sandy clasts, scattered monomineralic feldspars, clinopyroxenes, biotite and opaque minerals were observed, probably deriving from the weathering of volcanic aggregates.

Among the additives, dispersed charcoal (**Fig 5, r**) and organic unburnt fibres (**Fig 5, s**) were occasionally detected, both in samples TSF_T9 as well as in the other ones.

Fig 3. Low-magnification PLM micrographs of TSF samples (transmitted light, plane polars). (**a**) TSF_T9; (**b**) TSF_LN; (**c**) TSF_SN; (**d**) TSF_SS. Legend: Vp = Vesicular pumice; Cf = Ceramic fragments.

Fig 4. XRPD patterns of the four analysed TSF mortar samples. Mineral phases labelled according to [87] when mentioned: Clc = Clinochlore; Bt = Biotite; Ms = Muscovite; AFm = AFm phases of the Ca-rich calcium monocarboaluminate type (authigenic process); Php = Phillipsite; Anl = Analcime; Qz = Quartz; Kfs = K-Feldspar; Pl = Plagioclase; Cal = Calcite; Cpx = Clinopyroxenes; Hem = Hematite; Ilm= Ilmenite; Py = Pyrite (post-depositional component); Znc = Zincite (internal standard).

Fig 5. In-detail mineral-petrographic characterization of TSF_T9. (a) Cross section of the sample, with identification of the main aggregate components; PLM images of TSF_T9 sample, acquired in plane nicols (PN) and crossed nicols (XN); (b) low-birefringence lime binder in an area rich in poorly sorted ceramic fragments (XN); (c) a lime lump surrounded by an outer rim showing low birefringence (PN); (d) the ceramic fragments' assemblage, mainly constituted by poorly sorted grains (PN); (e) feebly sorted sub-rounded clasts of pumice, with evident reaction edges of pozzolanic dissolution and reaction (PN); (f) and (g) a clast of aphyric pumice, with scattered phenocrysts of k-feldspars (sanidine) and plagioclase (XN and PN respectively); (h) and (i) a clast of pumice with a feebly-porphyritic texture, containing scattered feldspar microlites in a primarily glassy groundmass (XN and PN respectively); (j) an angular chip of weakly-lithified tuff with cineritic groundmass (PN); (k) a coarse fragment of weakly-lithified tuff including volcanic shards, fragmented pumices and scattered biotite and feldspars (PN); (l) a small angular fragment of very dense vitrophyric obsidian, with sharp reaction rims (XN); (m) a clast of dense porphyritic lava with k-feldspars and clinopyroxenes phenocrysts (XN); (n) concentration of opaque fine-grained iron slags (PN); (o) an angular chip of crystalline limestone (PN); (p) a coarse clast of marble, composed of anhedral metamorphized crystals of calcite (PN); (q) a chip of dolostone with clear de-dolomitization edges (PN); (r) an organic charcoal inclusion (PN); (s) a small fragment of unburned organic fibre (PN). Legend – *Rock clasts and main aggregates*: P = pumice; aP = aphyric pumice; pP = porphyritic pumice; Lv = volcanic lava; wLT = weakly-lithified tuff; Obs = obsidian; Sh = volcanic shard; Mb = marble; Dol = dolostone; Cc = crystalline limestone; Cf = ceramic fragments; *Lime binder and reactions*: L = lime lump; Rr = reaction rim; *Minerals*: Bt = biotite; Fls = feldspar; Kfs = k-feldspar; Pl = plagioclase; Qz = quartz; Cpx = clinopyroxene; Op = opaque minerals; *Other inclusions*: Ch = charcoal; Of = organic fibre; Is = iron slag.

Table 1. Cumulative results of PLM and bulk QPA-XRPD analyses on the four mortar samples from San Felice well-cistern. Legend: b.d. = below detection; **- -** very low presence (<2%); **-** low presence (2-7.5%); **+** = moderate occurrence (7.5-20%); **++** = relevant occurrence (20-40%); **+++** = abundant occurrence (> 40%); LB = Lime binder.

**Sample**	**TSF_T9**	**TSF_LN**	**TSF_SN**	**TSF_SS**
**Function**	Coating (socle)	Joint mortar	Linings (?)	Linings (?)
**Description**	junction between masonry and floor	between two *sesquipedali* of the masonry	Flooring	Flooring
PLM				

**Binder texture**	LB	LB	LB	LB
**Lime Lumps**	-	+	-	-
**Voids**	-	+	++	+
**Local alluvial sands**	-	++	+++	+++
**Ceramic fragments**	+++	++	+	-
**Volcanic tephra**	++	+	-	-
**Crystalline Limestone chips**	+	+	-	-
**Iron slags**	+	b.d.	b.d.	b.d.
**Shells**	- -	-	- -	- -
**Fibers and charcoal**	- -	- -	-	- -
**estimated B:A prop.**	1:2	1:2	1:3	1:2
QPA-XRPD				
**Calcite**	19.5	19.2	11.7	26.1
**AFm**	b.d.	b.d.	0.3	1.3
**Dolomite**	b.d.	3.5	2.5	4.96
**Quartz**	2.4	21.7	19.4	25.8
**Plagioclase**	3.9	10.4	8.5	7.2
**K-feldspar**	4.2	4.7	4.1	3.5
**Microcline**	4.0	3.6	2.1	2.1
**Analcime**	0.9	0.4	0.4	0.2
**Phillipsite**	0.1	0.5	b.d.	0.6
**Clinopyroxene**	11.4	5.4	6.2	4.4
**Hematite**	0.5	0.3	0.3	0.3
**Ilmenite**	0.2	0.1	0.2	0.2
**Muscovite**	b.d.	2.3	2.3	3.0
**Biotite**	0.7	1.4	b.d.	0.7
**Clinochlore**	b.d.	3.7	2.2	4.1
**Pyrite**	b.d.	0.6	0.2	0.1

**Smectite/M-S-H**	26.0	b.d.	13.6	b.d.
**Amorphous**	26.1	22.4	26.1	15.6

Binder Characterization and Reaction in Submerged Environment

The reaction processes involving reactive aggregates and the lime-based binder in TSF_T9 were investigated in-detail by SEM-EDS and QPA-XRPD analyses of the separated binder fraction. The backscattered electron images, as presented in **Fig 6**, show relevant interfacial dissolution features in most of the pozzolanic aggregates, both pyroclastic clasts and ceramic fragments. Furthermore, pumice grains are consistently dissolved by the reaction processes even in the cores (**Fig. 7, a**), with few surviving portions of fresh volcanic glass enriched in interfacial dissolution pits (**Fig. 7, b, b1**), and relevant filling of the outgassing vesicles by compact clusters of anhedral nanostructured particles with aluminosilicate-magnesian composition (**Fig. 7, b2**). The binding matrix is constituted by a tight network of intergrown nanostructured phases with pseudo-lamellar crystal habits (**Fig. 7, c, d**). The chemical composition of the binding matrix clearly indicates the presence of Si, Mg, Al, and, subordinately, Ca and Fe (**Fig 6, a-c3, Fig. 7, d1**), apart from limited rounded sub-micrometric clusters enriched in calcium, to be considered as relicts of the original lime calcium-based binder of the mortar (**Fig. 7, d2**). Such articulated chemical profiles are primarily attributed to a complex array of hydraulic phases that ultimately formed after pozzolanic reaction. In general terms, the factors triggering the widespread reactivity of these materials derive from an interplay of parameters that are influenced both by the intrinsic physicochemical properties of the pozzolanic aggregates and the environmental conditions of precipitation.

QPA-XRPD analyses of the binder fraction showed the occurrence of very high aliquots of paracrystalline Mg-rich hydrated phases (M-S-H/M-A-S-H) as principal crystalline phase, up to 37.0 %wt (**Fig 6, g**), likely related to para-pozzolanic reactions between the lime-based binder and Mg-rich reactive aggregates [73] through a liquid medium [28]. These were mineralogically described using the structure of a turbostratically disordered smectite, as described at paragraph 4.1. The relevant amorphous phase (up to 52.5 %wt) may also be related to development of these hydrate phases. It has been already noticed

that salt-water plays a crucial role in facilitating the formation of M-S-H/M-A-S-H products. This is achieved by increasing the pH, which, in turn, promotes the dissolution of silicates and carbonates releasing Mg, Si, Al, ions in the system, which then combine to form Si-Al-Mg hydraulic phases [23,25,26,28,88]. As a matter of fact, the significant formation of M–S–H/M–A–S–H phyllosilicate gels is likely occurring when high concentrations of active magnesium, silica and alumina are present in the system. Whereas Al and Si were provided by the natural and artificial pozzolans, the sources of Mg in TSF_T9 within the aggregate fraction are less consistent and rely on the de-dolomitized dolostones and on certain Mg-rich ceramic fragments, exhibiting partially vitrified and reacted siliceous-aluminous matrices (**Fig 6, d-d1)**. However, the amount of active Mg occurring within the aggregate fraction is not sufficient to satisfy the abundant magnesium content detected by EDS analysis of matrices. The possible utilization of a magnesian binder must be rejected as confirmed by EDS analyses of the scattered lumps and calcination relicts, proving its original chemistry as eminently calcic (**Fig 6, e-e1, f-f1**).

Therefore, as already suggested in [28], it must be assumed that another relevant source of Mg was provided by the prolonged exposure to brackish water, having a significant concentration of magnesium ions (up to 3.7 %wt) in the overall amount of soluble salts [89].

Regarding the remaining phases, the QPA-XRPD shows the occurrence of reduced aliquots (2.2 %wt) of Mg-rich AFm phase of the hydrotalcite type [72] , confirming the high degree of Mg activity in the system, together with a very low amount of calcite (1.6 %wt), to be ascribed to the anthropogenic $CaCO_3$ included in the binder. This demonstrates that carbonation of the binder was minimized whereas the available portlandite primarily undergone pozzolanic interchanges, being the reactivity of the pozzolanic aggregates fostered in the alkaline and oxygen-depleted subaqueous conditions. Moreover, the sample appears clearly Ca-depleted. In fact, the low-Ca rates highlighted by EDS analyses in the matrixes prove that the development of ordinary Ca-based hydraulic phases (C-S-H; C-A-S-H) was completely subordinated to Mg-based ones in such chemical environment. Therefore, under this condition, the hydraulic reaction occurred in TSF_T9 sample deviated from the conventional calcium-silica-alumina ternary diagram due to specific conditions of alkali, magnesium and chlorine activity.

The remaining phases detected with the QPA-XRPD analysis of the TSF_T9 binder fraction are negligible and mainly consist in mineral residues from the aggregate, as goethite (2.3 %wt), representing fine liquid-suspended particles deriving from the disaggregation of the iron shards included in the sample.

Fig 6. Backscattered electron images and EDS microanalyses of sample TSF_T9. (a) SEM acquisition of an area of sample TSF_T9; (b) enlarged picture of the area highlighted by the white dashed line in figure (a); (b1) EDS analysis of unreacted volcanic glass within a pumice clast; (b2) Binder matrix of an area close to the pumice clast enriched in M-A-S-H; (c) pumice aggregate within the matrix of the sample; (c1) unreacted pumice clast; (c2 and c3) binder matrix enriched in M-A-S-H; (d) ceramic fragment; (d1) EDS analysis of the ceramic fragment; (e) lime lump; (e1) EDS analyses of the lime lump; (f) calcination relict; (f1) EDS analyses of the calcination relict; (g) high-magnification detail of the binder matrix's microstructure; (g1) EDS analysis of the investigated area; (h) XRPD pattern of the separated binder fraction of sample TSF_T9. Mineral phases labelled according to [87] when mentioned: Ms = Muscovite; AFm = AFM phases; Cal = Calcite; Cpx = Clinopyroxenes; Gth = Goethite. The quantification of mineral phases after QPA analysis is shown to the right of the XRPD profile.

Fig 7. Backscattered electron images and EDS microanalyses of the sample TSF_T9. (a) SEM acquisition of a highly reacted pumice clast; (b) detail of the area highlighted by the white dashed line in figure (b1); EDS analysis of unreacted volcanic glass within the pumice clast; (b2) EDS analysis of the reaction products filling an outgassing vescicle of the pumice clast; (c) SEM acquisition of a portion of binding matrix; (d) detail of the area highlighted by the white dashed line in figure (c); (d1) EDS analysis of the portion of binding matrix highlighted in figure (d); (d2) EDS analysis of the rounded cluster highlighted in figure (d).

Provenance of pyroclastic clasts

Preliminary minero-petrographic and microchemical investigation of the pyroclastic clasts included in TSF samples allowed to establish their incompatibility with the magmatic products of the Veneto region. In order to determine the provenance of these extra-regional juveniles on a geochemical basis, 29 volcanic clasts, comprising 23 aphyric and slightly porphyritic pumices, 5 weakly-lithified tuffs and 1 vitrophiric obsidian (**S1 Table**), were preliminary investigated via EDS microanalysis on apparently fresh glass, still preserving the geogenic chemical fingerprint. This technique has been already adopted

as a rapid and efficient tool for a preliminary assessment of volcanic pozzolans' geochemistry included in ancient mortar-based materials [40,61,66,90].

As a first comparison between the analysed clasts and the geochemical intervals of the volcanic products of the main Italian Plio-Quaternary magmatic districts [91,92], the resulting major elements' profiles were plotted in the TAS (Total Alkali vs Silica) diagram [93], reporting the relation between alkaline elements ($Na_2O + K_2O$) and silica (SiO_2). In this diagram, most of the TSF_T9 pyroclastic clasts can be chemically categorized as phonolites. However, some clasts (*a, c, z, 05*), primarily comprising weakly-lithified tuffs, exhibit compositions that fall between phonolites and trachytes. Specifically, the Si values in the pumice clasts range from 55.1 to 60.8 wt% with a standard deviation (st.dev.) typically < 1 wt% in aphyric samples and ≤ 1.5 in porphyritic ones. The concentration of Na + K in the pumice fragments is comprised around 14 ± 0.5 wt%, except for clast *c* (12.3 wt%), thus confirming their uniform geochemical composition.

On the other hand, st.dev. values exceeding 1.5 in Si concentration and 1 in Na + K concentrations were registered for weakly-lithified tuffs. This could be symptomatic of a higher heterogeneity, possibly determined by the occurrence of microcrystalline minerals in the cineritic matrices of these pyroclastic rock fragments, whose presence can be inferred also by the inhomogeneous interference colours observed in the SEM backscattered images (see **S2 Fig, b**).

The geochemical profile of the clasts is compatible with most of the volcanic products of the Campanian magmatic province, including the alkaline and highly-alkaline series of the main Phlegraean eruptions (pyroclastic products) [91,94], comprising the pre- and Campanian Ignimbrite (pre-CI/CI), pre- and Neapolitan Yellow Tuff (pre-NYT/NYT), post-NYT as well as the Phlegraean-correlated volcanoes of Ischia and Procida-Vivara islands (**Fig 8, a**). However, some of the clasts, characterized by low-Si concentrations, fall outside the intervals of the Phlegraean Fields.

Moreover, by only considering the TAS distribution, all the observed profiles except clast 09 appear geochemically compatible with the Somma-Vesuvius explosive events older than 79 CE (**Fig 8, b**). On the other hand, they are incompatible with the highly-alkaline pyroclastic deposits of the Roman volcanoes, i.e., the *harenae fossiciae* (Vitr. De Arch. 2.6.6), comprising Pozzolane Nere, Pozzolane Rosse and Pozzolanelle from the Colli Albani eruptive facies and *carbunculus* from Colli Sabatini, according to [95].

459 They appear incompatible also with most of the other products of the Roman and Tuscan magmatic
460 provinces [91,96,97], as well as with the rhyolites and rhyolitic breccias of the Oligocene eruptions of
461 the Euganean Hills in the Veneto region [98] (**Fig 8, c**). A very slight overlap can be only tracked with
462 certain pyroclastic phonolites of Vulcano in the Aeolian Arc Isles [99] (**Fig 8, d**).
463 These results demonstrate that TAS is non-conclusive for provenance determination of the pyroclastic
464 clasts under investigation. Therefore, trace elements, collected via LA-ICP-MS, were crucial to exactly
465 pinpoint their origin. Glassy/cineritic areas of 9 pumices and 2 weakly-lithified tuffs from sub-samples
466 TSF_T9B and TSF_T9C (**S2 Table**), presenting unreacted areas wide enough to be comprised within the
467 55-μm laser ablation's spot size, were selected and analysed avoiding as much as possible the altered
468 portions (**Fig 9**).
469 Good results were collected for most of the analysed pumices (both aphyric and porphyritic). In these
470 clasts, the quantification of REE and HFSE returned a low st.dev. (see the comments to the data reported
471 in the **S2 Table**), that, in particular, is coherent for the concentrations of Zr, Y, Nb, Yb, La, Th and Ta,
472 usually considered in literature for provenance discrimination [29,33,42,45,46,61,62,100]. Conversely,
473 the two analysed weakly-lithified tuff clasts (*a* and *s*) exhibited significantly high st.dev. and unusual
474 average values, confirming the geochemical inconsistency already evidenced after EDS investigations.
475 Therefore, while the LA-ICP-MS technique proved effective for tracking the geochemical fingerprint of
476 fresh and chemically homogeneous tephra, it is not appropriate for analysing volcanic rocks presenting
477 heterogeneous petro-mineralogical composition. Thus, these clasts were removed from the dataset
478 considered in the following analytical steps.
479 As suggested by the TAS, the discordancy with the volcanic products of Roman district (comprising Colli
480 Albani and Colli Sabatini pyroclastic products) and Aeolian Isles, is confirmed also by trace elements, as
481 demonstrated by Th/Ta vs Nb/Zr and Zr/Y vs Nb/Y scatterplots [61,101]. In the first one, the analysed
482 clasts plot within the field of the Neapolitan volcanoes (**S3 Fig, a**), characterized by the low ratio of Th/Ta
483 (usually < 15, except from Pomici di Mercato eruption of Mt Somma-Vesuvius), whereas, in the Zr/Y vs
484 Nb/Y scatterplot (**S3 Fig, b**), the clasts connoted by the high Nb/Y ratios (always > 1.7) overlap the field
485 of Campania and they are incompatible with both Tuscan/Roman Magmatic districts and Aeolian Isles.

To establish the provenance of the pyroclastic clasts within the Neapolitan district at high resolution, a selection of trace elements connoting their fingerprint was compared to that of around 950 pyroclastic products selected from literature, comprising the main eruptive events of the Phlegraean Fields, Mt. Somma-Vesuvius, Ischia and Procida-Vivara.

In the Zr/Y vs Nb/Y diagram (**Fig 10, a**), most of the samples overlap the Phlegraean Fields' intervals, showing strong correlations with the products of the post-NYT activities. However, some clasts with Nb/Y values < 2.0 (in particular *e* and *j*) present certain affinities with geological samples of the Ischia's pre- and post-Monte Epomeo Green Tuffs (MEGT). Additionally, other pumices (especially *k*) fall close to the intervals associated with certain pre-79 CE Mt. Somma-Vesuvius juveniles. The Nb/Zr vs La/Yb scatterplots, adopted in [33] for discrimination, do not provide any other relevant information (**Fig 10, b**).

Both the scatterplots indicate that the compatibility with the Phlegraean Fields of the analysed clasts is plausible, but it cannot be considered as ubiquitous. Consequently, to enhance the resolution of the analysis, non-fractional HSFE and REE from 950 geological markers constituting our in-house geochemical dataset were compared in exploratory bivariate scatterplots trace-by-trace. Notably, Yb concentrations show a relevant and coherent variability within the volcanic units of the Gulf of Naples, resulting extremely informative when plotted versus Zr, Nb and Th. A similar pattern is observed when Nd is plotted versus Rb.

In detail, in the Nd vs Rb scatterplot (**Fig 10, c**), and in the Yb vs Zr, Yb vs Nb, Yb vs Th ones (**Fig 11, a-c**), the 9 analysed pumices systematically overlap the intervals of distribution of the Phlegraean fields, connoted by positive correlation between Yb with Zr, Nb and Th, as well as between Nd with Rb. All the TSF_T9 clasts have Yb and Nd values higher than 4 and 60 ppm, respectively, making them incompatible with Mt. Somma-Vesuvius eruptions, characterized by Nd values < 50 ppm and Yb values < 2 ppm except for the older series, where Yb values can reach 5 ppm. The distribution of the Ischia pre-MEGT, MEGT and post-MEGT products appears somewhat inconstant. Although Yb and Nd are positively correlated with the other trace elements, when compared to the other Phlegraean products, these trends show slightly higher ratios, particularly evident in the Yb vs Th scatterplot (see **Fig 11, c**).

513 Having proved a ubiquitous compatibility with the Phlegraean Fields, the investigated clasts appear to
514 align in particular with the post-NYT eruptive events, characterized by high Zr, Th and Yb. However, in
515 most of the diagrams, the analysed pumices show a latent compatibility with pre-NYT, NYT and CI
516 juveniles too.
517 To prove with the highest degree of resolution the origin of the clasts within the Phlegraean domain,
518 discriminant analysis (DA) was carried out. The geological markers, subdivided according to the major
519 Phlegraean eruptive events, were used as classification factors, and the most informative trace elements
520 adopted for provenance discrimination in the scatterplots (Zr, Nb, Y, Th, Ta, Yb, La, Nd and Rb) were
521 selected as independent variables. The compatibility with possible sources was established considering
522 two probabilistic terms.
523 For all the analysed pumices, the DA reported a 1st probability correlation, ranging from 75 to 100%,
524 with the post-NYT volcanic products (see **Tab. 2**). This compatibility was eventually confirmed through
525 a spider-diagram reporting the trace element profiles of the analysed pumices, perfectly overlapping the
526 intervals of the geological marker samples associated with the post-NYT eruptions (**Fig 12**).
527 These outcomes confirm that the weakly-lithified deposits of the late Phlegraean eruptions, outcropping
528 around the Gulf of Naples' coastline, are the likely source of the volcanic material included in TSF
529 samples.
530
531 **Fig 8.** TAS scatterplots of pyroclastic clasts (pumices, obsidians, tuffs) included in TSF_T9 sample, overlapped to
532 the geochemical intervals of the main Plio-Quaternary volcanic districts of the Italian peninsula (reference for
533 geochemical fields taken from [42] and references therein), distributed as it follows: (a) the main eruptive events
534 of the Phlegraean Fields, comprising pre- and Campanian Ignimbrite (pre-CI/CI), pre- and Neapolitan Yellow Tuff
535 (pre-NYT/NYT), post-NYT, and Phlegraean-correlated volcanoes of Ischia and Procida-Vivara; (b) the three main
536 eruptive *facies* of Somma-Vesuvius; (c) the products of the Roman, Tuscan and Euganean Magmatic provinces; (d)
537 pyroclastic products of the Aeolian Arc Isles.
538
539 **Fig 9.** Identification of the pyroclastic clasts in the polished sections TSF_T9A, TSF_T9B and TSF_T9C samples
540 analysed by SEM-EDS (in yellow and light-blue dashed lines) and the nine pumices analysed by both SEM-EDS and

LA-ICP MS (in light-blue dashed line). The lower part of the figure reports the BSE acquisitions of the nine pumice clasts analysed by LA-ICP-MS, with indication of the Laser Ablation spot of analysis (the dimension of the red dots is scaled down to the instrumental 55 μm spot size).

Fig 10. Trace elements' scatterplot distribution of the pumices in TSF_T9A and TSF_T9B samples, in relation with that of juveniles' geological markers from the main eruptive facies of the volcanic units of the Gulf of Naples. A selection of discriminant trace elements for provenance recognition was adopted: (a) Nb/Y vs Zr/Y; (b) La/Yb vs Nb/Zr; (c) Nd vs Rd. Geochemical data of reference geological markers are taken from literature, as it follows: Phlegraean Fields (eruptive activities distinguished according to [102,103]: Pre-CI [102,104–107]; CI [102,104,107–109]; post-CI [104]; Pre-NYT [102,106,107]; NYT [102,110,111]; Post-NYT [61,102,110,112–117]; comprising Epoch I, II and III according to [117]; Somma-Vesuvius (eruptive activities distinguished according to [118]: Older series – Pomici di Base < 20 k.a. BP [105,119–124]; Greenish pumice [120,122,125]; Pomici di Mercato [120,122,124,126,127]; Avellino [119,120,124,127,128]; Protohistorical series – AP1/AP6 [120,122–124]; 79 CE [119,120,122,124,127,129–131]; post-79 CE – 472 CE [119,120,122–124]; Ischia, eruptive activities distinguished according to [132]: pre-MEGT [132–136]; MEGT [132,133]; post-MEGT [132–136]; Procida-Vivara [137].

Fig 11. Trace elements' scatterplot distribution of the pumices in TSF_T9A and TSF_T9B samples, in relation with that of juveniles' geological markers from the main eruptive *facies* of the volcanic units of the Gulf of Naples. A selection of discriminant trace elements for provenance recognition was adopted: (a) Yb vs Zr; (b) Yb vs Nd; (c) Yb vs Th. Geochemical data of reference geological markers are taken from literature, as it follows. Phlegraean Fields (eruptive activities distinguished according to [102,103]: Pre-CI [102,104–107]; CI [102,104,107–109]; post-CI [104]; Pre-NYT [102,106,107]; NYT [102,110,111]; Post-NYT [61,102,110,112–117]; comprising Epoch I, II and III according to [117]; Somma-Vesuvius (eruptive activities distinguished according to [118]: Older series – Pomici di Base < 20 k.a. BP [105,119–124]; Greenish pumice [120,122,125]; Pomici di Mercato [120,122,124,126,127]; Avellino [119,120,124,127,128]; Protohistorical series – AP1/AP6 [120,122–124]; 79 CE [119,120,122,124,127,129–131]; post-79 CE – 472 CE [119,120,122–124]; Ischia, eruptive activities distinguished according to [132]: pre-MEGT [132–136]; MEGT [132,133]; post-MEGT [132–136]; Procida-Vivara [137].

Fig 12. Comparative spider-diagram of trace elements (average values for each single clast) between the pumice glass measured by LA-ICP-MS in this study and the field of Post-NYT juveniles from literature (references for post-NYT are reported in the main text and captions of Figg 10 and 11). Data were normalized to the Primitive Mantle recalculated from pyrolite (PM, data from: [138]).

Table 2. Results of the discriminant analysis of pumice samples with probabilistic association to sources within the main Phlegraean eruptions (Classification Function Coefficients and Discriminant Function Coefficients are reported at the **S3. Table**).

Clast	Provenance (1st prob.)	Highest Group	Squared distance	Prob. (%)	Provenance (2nd prob.)	2nd Highest group	Squared distance	Prob. (%)	TOT prob. (%)
j	Post-NYT	44.3087	4.61778	90.32	pre-NYT	42.0199	9.1953	9.16	99.48
p	Post-NYT	47.6212	5.88543	75.17	pre-NYT	46.4381	8.25164	23.03	98.2
o	Post-NYT	54.886	8.51028	80.03	pre-NYT	53.3371	11.6079	17.01	97.04
e	Post-NYT	35.2316	20.086	97.76	pre-NYT	31.4485	27.6522	2.22	99.98
k	Post-NYT	63.3567	12.6565	91.23	NYT	60.8802	17.6097	7.67	98.9
n	Post-NYT	39.832	17.359	96.96	pre-NYT	36.3686	24.286	3.04	100
i	Post-NYT	16.6407	57.7087	98.12	pre-NYT	12.6858	65.6184	1.88	100
t	Post-NYT	40.453	46.5635	98.34	pre-NYT	36.3725	54.7244	1.66	100
v	Post-NYT	29.2316	76.88	99.92	pre-NYT	22.1365	91.0701	0.08	100

Discussion

The data obtained from this research are extremely interesting from an archaeological point of view, as they offer important information regarding the building technique and the environment in which the TSF well-cistern was placed, providing a new piece of evidence on the commerce and exploitation of the Vitruvian *pulvis* in the ancient Mediterranean.

The construction technique and environment of San Felice well-cistern

In a context-related perspective, the building technique of the well-cistern appears extremely complex. Nowadays, due to eustatic and subsidence phenomena, the first course of bricks positioned at the bottom of the structure lies at a depth of – 4.20/3.75 m MSL, but even in Roman times the base of the well-cistern was placed at a depth of around – 2.0 m MSL [48]. This implies that at least the foundations of the cistern, but more probably most of the structure, had to be built underwater. Therefore, it seems difficult to think that the Romans were able to build a structure made of local *sesquipedali* bricks laid on mortar joints and mortar-coated without using cofferdams [33,139], possibly constituted of a double bulkhead (Vitr., *De Arch*. 5.12.5). In order to work in an adequately dry space, it is even probable that machines for the lifting of the liquid infiltrations, as those described by Vitruvius (Vitr., *De Arch.* 10.6.1-4) [140,141], were employed avoiding as much as possible the intrusion of saltwater during construction. This technique was historically used in Venice to build underwater, but some pieces of evidence on the use of wooden scaffoldings to allow the setting of mortar-based materials underwater, dating back to the Roman times, were documented in other sites along the Northern Adriatic coast [142]. However, this solution does not deny the opportunity to use, whereas available, hydraulic mortars in order to fasten the bricks, improving the grip and bonding of the building materials. The natural and artificial pozzolans (volcanic tephra and crushed ceramics in particular) were processed and finely milled by the crafts to maximize their interfaces with the lime binder, thus increasing the overall waterproofing capability of the resulting mortars. In addition, the reactivity was certainly fostered by the brackish liquid medium the mortar was presumably mixed with and that came into contact with the binding materials shortly after the cofferdams were dismantled. In fact, when Phlegraean pozzolan-rich mortars are applied in masonry joints or *opus caementicium* casts for on-land buildings, where carbonation of lime-based binder is favoured by air circulation, the development of reaction processes in the matrices can be minimized. There are even cases where it appears almost null or just limited to the interface edges with the reactive elements [42,143]. Therefore, it is eventually clear how the buried, alkaline-rich submerged anoxic environment favoured the latent reactivity of certain aggregates over

aerial carbonation, influencing the development of pozzolanic or para-pozzolanic reaction processes. These complex reaction kinetics significantly contributed to the strength and durability of the compounds along time [7,26,73,100], as already recognized for underwater concrete piers of Roman harbour infrastructures [7,28,100,144]. However, in this case, a clear prevalence of Mg-based hydrated phases over traditional Ca-based aluminosilicate hydrates is observed. This opens new perspectives regarding the complex relationships between Ca and Mg in authigenic reaction products, currently under investigation our research group.

Regardless of the specific kinetics of the reactions, this result confirms – once again – the brilliant intuition of the Roman engineers, epitomized in the well-known Vitruvian book, regarding the waterproofing abilities and mechanical strength conferred by the combination of Neapolitan *pulvis* with lime pastes under highly alkaline and anoxic conditions. Recent studies, using replicas of ancient specimens, have demonstrated that mortar-based materials reinforced with volcanic tephra from the Neapolitan volcanic regions exhibit compressive strengths of approximately 7-8 MPa [33,145,146]. While not reaching the standards of contemporary cements, these findings indicate a significant improvement over the majority of ancient mortars and concretes. These crucial discoveries were initially embraced by the masons of the San Felice well-cistern and subsequently reinterpreted and adapted to the construction techniques and materials of the local tradition.

Commercialization and function of *pulvis puteolanus*

In general terms, the results obtained in this research offer a new piece on the diffusion of the Neapolitan pozzolans in the ancient Mediterranean. The ship-trading of the Neapolitan pumiceous tephra from Campania up to the northernmost Adriatic route seems to be substantiated by continuously expanding attestations, that now range from the High Imperial Age [42] to the Late Antiquity [46]. New scenarios emerge regarding the widespread distribution of Neapolitan pozzolans in this area, which were even utilized in a context previously seen as peripheral in the Roman world, such as the Venice Lagoon. This endorses the hypothesis that the material was transported as ship-ballast along the endo-lagunar route connecting Ravenna with *Altinum* and Aquileia [147]. In light of this data, the Lagoon of Venice looks

638 intricately innervated within the commercial networks of one of the most renowned and specialized
639 building materials of the authentic Roman tradition.
640 Considering the dimension of the well-cistern (6.7 × 7.7 m), which may have been intended to serve fresh
641 water to ships passing throughout the lagoon route, the quantity of mortar needed for the making of the
642 structure was surely significant and, as a consequence, the amount of volcanic pozzolan exploited.
643 Therefore, its use in the making of the mortars was not left to chance: as demonstrated by the different
644 concentrations of volcanic aggregates in the analyzed samples, the masons carefully dosed the imported
645 volcanic powder, by employing the largest quantity in the nodal joints of the well-cistern, to ensure an
646 optimal sealing.
647 The targeted utilization of *pulvis puteolanus* in the framework of waterproofing coatings is particularly
648 interesting. In contrast to the structural applications observed in both on-land buildings and underwater
649 piers in harbour structures, this employment of the volcanic powder seems to primarily entail the
650 material for waterproofing and coating purposes. In particular, the significant presence of crushed
651 ceramics in the examined mortars is a fundamental aspect to consider. In fact, the archaeological
652 evidence demonstrate that volcanic pozzolans-enriched mortars, presenting no ceramic inclusions,
653 were mainly employed in masonry joints and structural mortars and concretes [12,40,66,148,149],
654 possibly for improving the strength and the cohesive properties of the compounds, as recalled by
655 Vitruvius regarding both the *pulvis* (Vitr. *De Arch*. 2.6.1) and the *harenae fossiciae* (Vitr. *De Arch*. 2.4.1-2).
656 On the other hand, the concurrent utilization of both natural and artificial pozzolans (pyroclastic rocks
657 and crushed ceramics, respectively) represents a common construction tradition among Roman
658 artisans, frequently adopted for lime-based coatings of hydraulic infrastructures [26,41,149–151].

659

660 Conclusions

661 This study provides many insights in the current field of research on ancient hydraulic mortars. The
662 main results, summarized below, can be split into three categories, comprising: (a) highlights on the
663 kinetics of pozzolanic and para-pozzolanic reaction processes; (b) novel tools for the provenance

664 determination of volcanic pozzolans, and (c) crucial advances on the technology of ancient pozzolanic
665 mortars and on the trading of *pulvis puteolanus* in Northern Italy in Roman times.

666 • **Kinetics of pozzolanic and para-pozzolanic reaction processes**. The study has provided new
667 insights regarding the development of the reaction processes in ancient mortar-based materials
668 in submerged anoxic environments. Being clear the role of saltwater in fostering the latent
669 "pozzolanicity" of certain materials, the prevalent formation of Mg-based hydrate products (M-
670 S-H and M-A-S-H) over traditional Ca-based pozzolanic ones (C-S-H and C-A-S-H) was promoted
671 by the long-term evolution of the reactions, as the binders appear clearly Ca-depleted and feebly
672 carbonated. This opens new perspectives on the complex kinetics of reactions occurring in
673 ancient underwater mortars and on the crucial role played by Mg ions in these processes.

674 • **Tools for provenance determination of volcanic pozzolans**. A nodal point of the study was
675 the provenance determination of the pyroclastic clasts identified in the samples. From a
676 methodological point of view, the work offers new tools for measuring the geochemical
677 fingerprint of volcanic aggregates in ancient mortar-based materials and for tracking their
678 origin. In particular, an enriched set of discriminant diagrams was proposed, outlining the
679 crucial role of Yb and Nd for a geochemical differentiation of juveniles within the Neapolitan
680 magmatic district. The statistical processing of the descriptive geochemical elements with
681 multivariate techniques, such as the discriminant analysis (DA), can constitute another useful
682 tool to validate and crosscheck the information derived from scatterplot observations.

683 • **Technology of ancient pozzolanic mortars and trading of *pulvis puteolanus***. From a
684 historical-archaeological point of view, this study documented a new case of utilization of
685 Phlegraean volcanic pozzolans in Roman times and the first attestation of this material in the
686 lagoon of Venice. This evidence allows to reconsider this territory as strictly connected in the
687 ancient Northen Adriatic economic-commercial routes and deeply embedded in the Roman
688 technological awareness of genuine central-Italic tradition, reinterpreted according to the forms,
689 methods and construction materials of the local built environment.

690

Supporting information

S1 Analytical methods. Instrumental equipment and standards.

S1 Fig Roman well-cisterns. Examples of Roman well-cistern documented in the site of Ca' Ballarin in the Northern Lagoon of Venice (up) and from Aquileia (down). Images taken from [48] (see references in the main text).

S2 Fig SEM-EDS investigation of pyroclastic clasts' mineral chemistry and iron slags.

S3 Fig TAS diagram. Trace elements' scatterplots of the pumice clasts in TSF_T9B and TSF_T9C subsamples analyzed by LA-ICP-MS; a) Nb/Zr vs Th/Ta scatterplot of pumice clasts in relation to the fields occupied by the Roman, Tuscan and Campanian magmatic provinces and Aeolian Arc Islands' volcanic products (compositional fields from [42] and references therein); b) Nb/Y vs Zr/Y scatterplot of clasts' samples in relation to the fields occupied by the Roman, Tuscan and Campanian magmatic provinces, and the Aeolian Arc Island's products (compositional fields from [42] and references therein).

S1 Table. **Major elements composition of the pyroclastic clasts**. Clasts were analyzed by SEM-EDS and described as %wt in oxides (average values and standard deviation); b.d. = Components below the detection limit.

S2 Table. Trace elements of the pyroclastic clasts. Chemical results of the selected clasts of pumice analyzed by LA-ICP-MS (average values of multiple spot-size analyses are reported and related standard deviation). Values are described as ppm

S3 Table. Coefficients of the discriminant analysis.

Acknowledgments

We are grateful to Dr. Alessandro Asta (Soprintendenza Archeologia, Belle Arti e Paesaggio per l'area metropolitana di Venezia e le province di Belluno, Padova e Treviso) for the support in the underwater archaeological research and endorsement to excavation activities.

Author contributions

Conceptualization: Simone Dilaria, Michele Secco, Jacopo Bonetto, Gilberto Artioli

Data curation: Simone Dilaria, Giulia Ricci, Michele Secco, Tommaso Giovanardi

Formal analysis: Simone Dilaria, Giulia Ricci

Funding acquisition: Simone Dilaria, Carlo Beltrame, Jacopo Bonetto, Gilberto Artioli

Investigation: Simone Dilaria, Giulia Ricci

Methodology: Simone Dilaria, Michele Secco

Project administration: Simone Dilaria, Jacopo Bonetto, Carlo Beltrame

Resources: Carlo Beltrame, Elisa Costa

Software: Simone Dilaria

Supervision: Jacopo Bonetto, Gilberto Artioli

Validation: Simone Dilaria

Visualization: Simone Dilaria (figures 1a-b, 3, 4, 5, 6, 7, 8, 9, 10, 11, tables 1 and 2 and all the figures and tables in the supplementary materials), Elisa Costa (figure 1c and 2), Tommaso Giovanardi (figure 12)

Writing – original draft: Simone Dilaria (introduction, characterization of raw materials, provenance of pyroclastic clasts, Discussion, conclusions); Carlo Beltrame (The archaeological context and mortar sampling, The construction technique and environment of San Felice well-cistern), Elisa Costa (The archaeological context and mortar sampling), Giulia Ricci (multi-analytical protocol and Binder Characterization and Reaction in Submerged Environment), Michele Secco (Binder Characterization and Reaction in Submerged Environment), Jacopo Bonetto (Commercialization and function of *pulvis puteolanus*)

Writing – review & editing: all the authors contributed in the review and editing of the manuscript

References

1. Hobbs LW, Siddall R. Cementitious Materials of the Ancient World. In: Ringbom A, Hohlfelder RL, editors. Building Roma aeterna: current research on Roman mortar and concrete Proceedings of the conference, March 27-29, 2008. Helsinki, 2011: Societas Scientiarum Fennica; 2011. pp. 35–60.
2. Artioli G, Secco M, Addis A. The vitruvian legacy: Mortars and binders before and after the Roman world. European Mineralogical Union Notes in Mineralogy. 2019. pp. 151–202. doi:10.1180/EMU-notes.20.4
3. Dilaria S, Secco M. Mortar Recipes Through the Ages. A Brief Review of Data from Prehistory to Late Antiquity. Arheol i Prir Nauk. 2022;18: 113–126. doi:10.18485/arhe_apn.2022.18.9
4. Vasiliki Pachta, Maria Stefanidou, Stavroula Konopisi, Ioanna Papayianni. Technological Evolution of Historic Structural Mortars. J Civ Eng Archit. 2014;8: 846–854. doi:10.17265/1934-7359/2014.07.005
5. Lancaster LC. Pozzolans in Mortar in the Roman Empire: An Overview and Thoughts on Future Work. Mortiers et hydraulique en Méditerranée antique. Presses universitaires de Provence; 2019. pp. 31–39. doi:10.4000/books.pup.39835
6. Jackson MD, Logan JM, Scheetz BE, Deocampo DM, Cawood CG, Marra F, et al. Assessment of material characteristics of ancient concretes, Grande Aula, Markets of Trajan, Rome. J Archaeol Sci. 2009;36: 2481–2492. doi:10.1016/j.jas.2009.07.011
7. Jackson MD, Mulcahy SR, Chen H, Li Y, Li Q, Cappelletti P, et al. Phillipsite and Al-tobermorite mineral cements produced through low-temperature water-rock reactions in Roman marine concrete. Am

Mineral. 2017;102: 1435–1450. doi:10.2138/am-2017-5993CCBY

8. Borges C, Santos Silva A, Veiga R. Durability of ancient lime mortars in humid environment. Constr Build Mater. 2014;66: 606–620. doi:10.1016/j.conbuildmat.2014.05.019

9. Seymour LM, Maragh J, Sabatini P, Di Tommaso M, Weaver JC, Masic A. Hot mixing: Mechanistic insights into the durability of ancient Roman concrete. Sci Adv. 2023;9. doi:10.1126/sciadv.add1602

10. MacFarlane J, Vanorio T, Monteiro PJM. Multi-scale imaging, strength and permeability measurements: Understanding the durability of Roman marine concrete. Constr Build Mater. 2021;272: 121812. doi:10.1016/j.conbuildmat.2020.121812

11. Jackson MD, Vola G, Všianský D, Oleson JP, Scheetz BE, Brandon C, et al. Cement Microstructures and Durability in Ancient Roman Seawater Concretes. RILEM Bookseries. 2012. pp. 49–76. doi:10.1007/978-94-007-4635-0_5

12. Dilaria S, Secco M, Bonetto J, Ricci G, Artioli G. Making Ancient Mortars Hydraulic. How to Parametrize Type and Crystallinity of Reaction Products in Different Recipes. Conservation and Restoration of Historic Mortars and Masonry Structures HMC 2022 RILEM Bookseries, vol 42. 2023. pp. 36–52. doi:10.1007/978-3-031-31472-8_4

13. Richardson IG. The nature of C-S-H in hardened cements. Cem Concr Res. 1999;29: 1131–1147. doi:10.1016/S0008-8846(99)00168-4

14. Richardson IG. Tobermorite/jennite- and tobermorite/calcium hydroxide-based models for the structure of C-S-H: Applicability to hardened pastes of tricalcium silicate, β-dicalcium silicate, Portland cement, and blends of Portland cement with blast-furnace slag, metakaol. Cem Concr Res. 2004;34: 1733–1777. doi:10.1016/j.cemconres.2004.05.034

15. Arizzi A, Cultrone G. Mortars and plasters—how to characterise hydraulic mortars. Archaeol Anthropol Sci. 2021;13. doi:10.1007/s12520-021-01404-2

16. L'Hôpital E, Lothenbach B, Le Saout G, Kulik D, Scrivener K. Incorporation of aluminium in calcium-silicate-hydrates. Cem Concr Res. 2015;75: 91–103. doi:10.1016/j.cemconres.2015.04.007

17. L'Hôpital E, Lothenbach B, Scrivener K, Kulik DA. Alkali uptake in calcium alumina silicate hydrate (C-A-S-H). Cem Concr Res. 2016;85: 122–136. doi:10.1016/j.cemconres.2016.03.009

18. Hong S-Y, Glasser F. Alkali sorption by C-S-H and C-A-S-H gels. Cem Concr Res. 2002;32: 1101–1111. doi:10.1016/S0008-8846(02)00753-6

19. Roosz C, Grangeon S, Blanc P, Montouillout V, Lothenbach B, Henocq P, et al. Crystal structure of

magnesium silicate hydrates (M-S-H): The relation with 2:1 Mg-Si phyllosilicates. Cem Concr Res. 2015;73: 228–237. doi:10.1016/j.cemconres.2015.03.014

20. Nied D, Enemark-Rasmussen K, L'Hopital E, Skibsted J, Lothenbach B. Properties of magnesium silicate hydrates (M-S-H). Cem Concr Res. 2016;79: 323–332. doi:10.1016/j.cemconres.2015.10.003

21. Bernard E, Lothenbach B, Rentsch D, Pochard I, Dauzères A. Formation of magnesium silicate hydrates (M-S-H). Phys Chem Earth. 2017;99: 142–157. doi:10.1016/j.pce.2017.02.005

22. Brew DRM, Glasser FP. Synthesis and characterisation of magnesium silicate hydrate gels. Cem Concr Res. 2005;35: 85–98. doi:10.1016/j.cemconres.2004.06.022

23. Bechor B, Sivan D, Miko S, Hasan O, Grisonic M, Rossi IR, et al. Salt pans as a new archaeological sea-level proxy: A test case from Dalmatia, Croatia. Quat Sci Rev. 2020;250: 106680. doi:10.1016/j.quascirev.2020.106680

24. Alonso-Olazabal A, Ortega LA, Zuluaga MC, Ponce-Antón G, Echevarría JJ, Fernández CA. Compositional characterization and chronology of roman mortars from the archaeological site of arroyo de la dehesa de velasco (Burgo de osma-ciudad de osma, Soria, Spain). Minerals. 2020;10: 393. doi:10.3390/min10050393

25. Ponce-Antón G, Zuluaga MC, Ortega LA, Mauleon JA. Petrographic and chemical-mineralogical characterization of mortars from the cistern at amaiur castle (Navarre, Spain). Minerals. 2020;10. doi:10.3390/min10040311

26. Secco M, Dilaria S, Bonetto J, Addis A, Tamburini S, Preto N, et al. Technological transfers in the Mediterranean on the verge of Romanization: Insights from the waterproofing renders of Nora (Sardinia, Italy). J Cult Herit. 2020;44: 63–82. doi:10.1016/j.culher.2020.01.010

27. Ricci G, Secco M, Addis A, Pistilli A, Preto N, Brogiolo G Pietro, et al. Integrated multi‐analytical screening approach for reliable radiocarbon dating of ancient mortars. Sci Rep. 2022;12: 3339. doi:10.1038/s41598-022-07406-x

28. Secco M, Asscher Y, Ricci G, Tamburini S, Preto N, Sharvit J, et al. Cementation Processes of Roman Pozzolanic Binders from Caesarea Maritima (Israel). Constr Build Mater. 2022;355: 129128. doi:10.2139/ssrn.4087431

29. Dilaria S, Previato C, Bonetto J, Secco M, Zara A, De Luca R, et al. Volcanic Pozzolan from the Phlegraean Fields in the Structural Mortars of the Roman Temple of Nora (Sardinia). Heritage. 2023;6: 567–586. doi:10.3390/heritage6010030

30. Lancaster LC. Mortars and plasters—How mortars were made. The literary sources. Archaeol Anthropol Sci. 2021;13: 1–20. doi:10.1007/s12520-021-01395-0
31. Greco A V. “Virtutes materiae.” Il contributo delle fonti latine nello studio di malte, intonaci e rivestimenti nel mondo romano. Sandhi, Or. 2011.
32. Vitruvio. De Architectura, Vol. I-II. Gros P, Corso A, Rromano E, editors. Torino; 1997.
33. Brandon CJ, Hohlfelder RL, Jackson MD, Oleson JP. Building for Eternity: The History and Technology of Roman Concrete Engineering in the Sea. Oleson JP, editor. Oxford: Oxbow Books; 2014.
34. Gianfrotta PA. Questioni di pilae e di pulvis puteolanus. J Anc Topogr. 2009;19: 101–120.
35. Gianfrotta PA. I porti dell’area Flegrea. In: eds. Laudizi G, Marangio C, editors. Porti, approdi e linee di rotta nel Mediterraneo antico, Atti del seminario di studi Lecce 1996. Studi di filologia e letteratura dell’Università di Lecce 4; 1998. pp. 153–176.
36. Stanislao C, Rispoli C, Vola G, Cappelletti P, Morra V, De Gennaro M. Contribution to the knowledge of ancient Roman seawater concretes: Phlegrean pozzolan adopted in the construction of the harbour at Soli-Pompeiopolis (Mersin, Turkey). Period di Mineral. 2011;80: 471–488. doi:10.2451/2011PM0031
37. Vola G, Gotti E, Brandon C, Oleson JP, Hohlfelder RL. Chemical, mineralogical and petrographic characterization of Roman ancient hydraulic concretes cores from Santa Liberata, Italy, and Caesarea Palestinae, Israel. Period di Mineral. 2011;80: 317–338. doi:10.2451/2011PM0023
38. Paternoster G, Rinzivillo R, Maddalena P, Vitale A, De Rosa C, Paduano L, et al. La cronologia delle malte del Rione Terra di Pozzuoli (Napoli). In: PATRON EDITORE 2007, editor. Atti del IV congresso nazionale AIAR. Pisa; 2007. pp. 365–378.
39. Di Benedetto C, Graziano SF, Guarino V, Rispoli C, Munzi P, Morra V, et al. Romans’ established skills: Mortars from D46b mausoleum, porta mediana necropolis, Cuma (Naples). Mediterr Archaeol Archaeom. 2018;18: 131–146. doi:10.5281/zenodo.1285895
40. Rispoli C, De Bonis A, Guarino V, Graziano SF, Di Benedetto C, Esposito R, et al. The ancient pozzolanic mortars of the Thermal complex of Baia (Campi Flegrei, Italy). J Cult Herit. 2019;40: 143–154. doi:10.1016/j.culher.2019.05.010
41. Rispoli C, De Bonis A, Esposito R, Graziano SF, Langella A, Mercurio M, et al. Unveiling the secrets of Roman craftsmanship: mortars from Piscina Mirabilis (Campi Flegrei, Italy). Archaeol Anthropol Sci. 2020;12. doi:10.1007/s12520-019-00964-8
42. Dilaria S, Secco M, Ghiotto AR, Furlan G, Giovanardi T, Zorzi F, et al. Early exploitation of Neapolitan

pozzolan (pulvis puteolana) in the Roman theatre of Aquileia, Northern Italy. Sci Rep. 2023;13: 1–19. doi:10.1038/s41598-023-30692-y

43. Djerad MS, Boufenara K, Courtils J des, Cantin N, Lefrais Y. Multianalytical Characterisation and Provenance Investigation of Natural Pozzolana in Roman Lime Mortars From the Archaeological Site of Hippo Regius (Algeria). Mediterr Archaeol Archaeom. 2022;22: 231–248. doi:10.5281/zenodo.7412076

44. Bridi E. L'impiego delle polveri pozzolaniche nell'edilizia antica: studio archeologico ed archeometrico di alcune strutture ed infrastrutture di epoca romana. Master Thesis, University of Padova. 2015.

45. Lancaster L, Sottili G, Marra F, Ventura G. Provenancing of lightweight volcanic stones used in ancient Roman concrete vaulting: Evidence from Rome. Archaeometry. 2011;53: 707–727. doi:10.1111/j.1475-4754.2010.00565.x

46. Dilaria S, Secco M, Rubinich M, Bonetto J, Miriello D, Barca D, et al. High-performing mortar-based materials from the late imperial baths of Aquileia: An outstanding example of Roman building tradition in Northern Italy. Geoarchaeology. 2022;37: 637–657. doi:10.1002/gea.21908

47. Rubinich M, Bonetto J, Cadario M, Dilaria S, Martinelli N. Le 'Grandi Terme' di Aquileia: nuovi dati dai sondaggi geognostici sui metodi costruttivi e sulla cronologia di costruzione. Orizzonti. 2024;XXV: 21–43.

48. Beltrame C, Medas S, Mozzi P, Ricci G. Roman 'Well-cisterns', Navigational Routes, and Landscape Modifications in the Venice Lagoon and Northeastern Adriatic. Int J Naut Archaeol. 2023;0: 1–25. doi:10.1080/10572414.2023.2182984

49. Ciancioisi A. Il "pozzo alla veneziana": una soluzione complessa per un'esigenza quotidiana. Revisione dei dati archeologici sull'approvvigionamento idrico nei centri lagunari durante il medioevo. Il caso di Jesolo (VE). Archeol Mediev. 2021;48: 371–388.

50. Primon S, Mozzi P. Torcello e la morfologia della laguna tra l'età romana e il Medioevo. In: Calaon D, Zendri E, Biscontin G, editors. Torcello scavata Patrimonio condiviso, 2 – Lo scavo 2012–2013. Regione del Veneto; 2014. pp. 105–121.

51. Serandrei Barbero R, Albani AD, Zecchetto S. Palaeoenvironmental significance of a benthic foraminiferal fauna from an archaeological excavation in the Lagoon of Venice, Italy. Palaeogeogr Palaeoclimatol Palaeoecol. 1997;136: 41–52. doi:10.1016/S0031-0182(97)00090-4

52. Previato C. Modi d'uso e sistemi di messa in opera del laterizio in Italia settentrionale in età repubblicana. In: Bonetto J, Bukowiecki E, Volpe R, editors. Alle origini del laterizio romano Nascita e diffusione del mattone cotto nel Mediterraneo tra IV e I secolo aC Atti del II Convegno internazionale "laterizio" Padova

aprile 2016. Quasar; 2019. pp. 369–382.

53. Bonetto J. Maestranze greche e laterizio cotto: alle origini dell'architettura della Cisalpina. In: Bonetto J, Bukowiecki E, Vole R, editors. Alle origini del laterizio romano Nascita e diffusione del mattone cotto nel Mediterraneo tra IV e I secolo aC Atti del II Convegno internazionale "laterizio" Padova aprile 2016. Edizioni Quasar; 2019. pp. 317–334.

54. Canal E. Archeologia della laguna di Venezia, 1960-2010. Caselle, S. 2013.

55. D'alpaos C, D'alpaos A. The valuation of ecosystem services in the venice lagoon: A multicriteria approach. Sustain. 2021;13: 1–15. doi:10.3390/su13179485

56. Pecchioni E, Fratini F, Cantisani E. Atlante Malte Antiche. In sezione sottile al microscopio ottico. Nardini Editore; 2014.

57. Rietveld HM. A profile refinement method for nuclear and magnetic structures. J Appl Crystallogr. 1969;2: 65–71. doi:10.1107/s0021889869006558

58. Serandrei Barbero R, Albani AD, Bonardi M. Ancient and modern salt marshes in the Lagoon of Venice. Palaeogeogr Palaeoclimatol Palaeoecol. 2004;202: 229–244. doi:10.1016/S0031-0182(03)00636-9

59. Addis A, Secco M, Marzaioli F, Artioli G, Chavarría Arnau A, Passariello I, et al. Selecting the most reliable 14C dating material inside mortars: The origin of the Padua Cathedral. Radiocarbon. 2019;61: 375–393. doi:10.1017/RDC.2018.147

60. Ricci G, Secco M, Marzaioli F, Passariello I, Terrasi F, Artioli G. New strategies in mortar characterization and radiocarbon dating. 2019 IMEKO TC4 International Conference on Metrology for Archaeology and Cultural Heritage, MetroArchaeo 2019. 2019.

61. Marra F, D'Ambrosio E, Sottili G, Ventura G. Geochemical fingerprints of volcanic materials: Identification of a pumice trade route from Pompeii to Rome. Bull Geol Soc Am. 2013;125: 556–577. doi:10.1130/B30709.1

62. D'Ambrosio E, Marra F, Cavallo A, Gaeta M, Ventura G. Provenance materials for Vitruvius' harenae fossiciae and pulvis puteolanis: Geochemical signature and historical-archaeological implications. J Archaeol Sci Reports. 2015;2: 186–203. doi:10.1016/j.jasrep.2015.01.012

63. Marra F, Anzidei M, Benini A, D'Ambrosio E, Gaeta M, Ventura G, et al. Petro-chemical features and source areas of volcanic aggregates used in ancient Roman maritime concretes. J Volcanol Geotherm Res. 2016;328: 59–69. doi:10.1016/j.jvolgeores.2016.10.005

64. Dilaria S, Bonetto J, Germinario L, Previato C, Girotto C, Mazzoli C. The stone artifacts of the National

903 Archaeological Museum of Adria (Rovigo, Italy): a noteworthy example of heterogeneity. Archaeol
904 Anthropol Sci. 2024;16: 14. doi:10.1007/s12520-023-01911-4
905 65. Montana G, Cau Ontiveros MÁ, Polito AM, Azzaro E. Characterisation of clayey raw materials for ceramic
906 manufacture in ancient Sicily. Appl Clay Sci. 2011;53: 476–488. doi:10.1016/j.clay.2010.09.005
907 66. Columbu S, Garau AM, Lugliè C. Geochemical characterisation of pozzolanic obsidian glasses used in the
908 ancient mortars of Nora Roman theatre (Sardinia, Italy): provenance of raw materials and historical–
909 archaeological implications. Archaeol Anthropol Sci. 2019;11: 2121–2150. doi:10.1007/s12520-018-
910 0658-y
911 67. Prochaska W. The use of geochemical methods to pinpoint the origin of ancient white marbles. Mineral
912 Petrol. 2023. doi:10.1007/s00710-023-00833-2
913 68. De Lorenzi Pezzolo A, Colombi M, Mazzocchin GA. Where did Roman masons get their material from? A
914 preliminary DRIFTS/PCA investigation on mortar aggregates from X Regio buildings in the Veneto area
915 (NE Italy) and their potential sources. Environ Sci Pollut Res. 2018;25: 28798–28807.
916 doi:10.1007/s11356-018-2202-0
917 69. Maritan L, Nodari L, Mazzoli C, Milano a., Russo U. Influence of firing conditions on ceramic products:
918 Experimental study on clay rich in organic matter. Appl Clay Sci. 2006;31: 1–15.
919 doi:10.1016/j.clay.2005.08.007
920 70. Matschei T, Lothenbach B, Glasser FP. The AFm phase in Portland cement. Cem Concr Res. 2007;37: 118–
921 130. doi:10.1016/j.cemconres.2006.10.010
922 71. Ipavec A, Gabrovšek R, Vuk T, Kaučič V, Maček J, Meden A. Carboaluminate Phases Formation During the
923 Hydration of Calcite-Containing Portland Cement. J Am Ceram Soc. 2011;94: 1238–1242.
924 doi:10.1111/j.1551-2916.2010.04201.x
925 72. Artioli G, Secco M, Addis A, Bellotto M. Role of hydrotalcite-type layered double hydroxides in delayed
926 pozzolanic reactions and their bearing on mortar dating: Composition, Properties, Application. In:
927 Herbert P, editor. Cementitious Materials: Composition, Properties, Application. Berlin: Walter de Gruyter
928 GmbH; 2017. p. 500.
929 73. Bernard E, Lothenbach B, Pochard I, Cau-Dit-Coumes C. Alkali binding by magnesium silicate hydrates. J
930 Am Ceram Soc. 2019;102: 6322–6336. doi:10.1111/jace.16494
931 74. Bernard E. Magnesium silicate hydrate (M-S-H) characterization: temperature, calcium, aluminum and
932 alkali. Phd thesis. Université Bourgogne Franche-Comté. 2017.

933 75. Secco M, Maritan L, Mazzoli C, Lampronti GI, Zorzi F, Nodari L, et al. Alteration Processes of Pottery in
934 Lagoon-Like Environments. Archaeometry. 2011;53: 809–829. doi:10.1111/j.1475-4754.2010.00571.x
935 76. Secco M, Dilaria S, Addis A, Bonetto J, Artioli G, Salvadori M. The Evolution of the Vitruvian Recipes over
936 500Years of Floor-Making Techniques: The Case Studies of the Domus delle Bestie Ferite and the Domus
937 di Tito Macro (Aquileia, Italy). Archaeometry. 2018;60: 185–206. doi:10.1111/arcm.12305
938 77. Dilaria S, Secco M. Analisi archeometriche sulle miscele leganti (malte e calcestruzzi). L'anfiteatro di
939 Aquileia Ricerche d'archivio e nuove indagini di scavo, Scavi di Aquileia V, Quingentole. 2018. pp. 177–
940 186.
941 78. Teutonico JM, Ashall G, Garrod E, Yates T. A comparative study of hydraulic lime-based mortars. Int RILEM
942 Work Hist Mortars Charact Tests. 1999; 339–349.
943 79. Coutelas A. L'hydraulicité des mortiers antiques, entre préconçus et réalité: I'exemple des mor-tiers de
944 tuileau et autres matériaux de Gaule romaine. In: Ortega F, Bouffier S, editors. Mortiers et hydraulique en
945 Méditerranée antique, Archéologies Mé-diterranéennes 6, Aix-en-Provence. 2019. pp. 17–30.
946 80. Houghton BF, Wilson CJN. A vesicularity index for pyroclastic deposits. Bull Volcanol. 1989;51: 451–462.
947 doi:10.1007/BF01078811
948 81. Astolfi G, Colombara F. La geologia dei Colli Euganei. Programma. Padova; 1990.
949 82. Macera P, Gasperini D, Piromallo C, Blichert-Toft J, Bosch D, Del Moro A, et al. Geodynamic implications of
950 deep mantle upwelling in the source of Tertiary volcanics from the Veneto region (South-Eastern Alps). J
951 Geodyn. 2003;36: 563–590. doi:10.1016/j.jog.2003.08.004
952 83. Wu R, Zhang Y, Zhang G, An S. Enhancement effect and mechanism of iron tailings powder on concrete
953 strength. J Build Eng. 2022;57: 104954. doi:10.1016/j.jobe.2022.104954
954 84. Papadimitriou G, Kordatos J. The brown waterproofing plaster of the ancient cisterns in Laurion and its
955 weathering and degradation. In: Maniatis Y, Herz N, Basiakos Y, editors. Asmosia III Athens: Transactions
956 of the 3rd International Symposium of the Association for the Study of Marble and other Stones used in
957 Antiquity. 1995. pp. 277–284.
958 85. Böttger KG, Thiedig F, Knöfel D. Keltisch-römische Mörtel vom Magdalensberg in Kärnten / Celtic-Roman
959 Mortars from Magdalensberg in Carinthia. Restor Build Monum. 2002;8: 19–42. doi:10.1515/rbm-2002-
960 5638
961 86. Katayama T. The so-called alkali-carbonate reaction (ACR) — Its mineralogical and geochemical details,
962 with special reference to ASR. Cem Concr Res. 2010;40: 643–675. doi:10.1016/j.cemconres.2009.09.020

963 87. Whitney DL, Evans BW. Abbreviations for names of rock-forming minerals. Am Mineral. 2010;95: 185–
964 187.
965 88. Prinčič T, Štukovnik P, Pejovnik S, De Schutter G, Bokan Bosiljkov V. Observations on dedolomitization of
966 carbonate concrete aggregates, implications for ACR and expansion. Cem Concr Res. 2013;54: 151–160.
967 doi:10.1016/j.cemconres.2013.09.005
968 89. Dickson AG, Goyet C. Handbook of methods for the analysis of the various parameters of the carbon
969 dioxide system in sea water. Oak Ridge. 1994.
970 90. Miriello D, Barca D, Bloise A, Ciarallo A, Crisci GM, De Rose T, et al. Characterisation of archaeological
971 mortars from Pompeii (Campania, Italy) and identification of construction phases by compositional data
972 analysis. J Archaeol Sci. 2010;37: 2207–2223. doi:10.1016/j.jas.2010.03.019
973 91. Peccerillo A. Plio-Quaternary Volcanism in Italy: Petrology, Geochemistry. Berlin; 2005.
974 92. Branca S, Cinquegrani A, Cioni R, Conte AM, Conticelli S, De Astis G, et al. The Italian Quaternary
975 Volcanism. Alp Mediterr Quat. 2023;36: 221–284. doi:10.26382/AMQ.2023.09
976 93. Le Bas MJL, Maitre RWL, Streckeisen A, Zanettin B. A Chemical Classification of Volcanic Rocks Based on
977 the Total Alkali-Silica Diagram. J Petrol. 1986;27: 745–750. doi:10.1093/petrology/27.3.745
978 94. Morra V, Calcaterra D, Cappelletti P, Colella A, Fedele L, de Gennaro R, et al. Urban geology: Relationships
979 between geological setting and architectural heritage of the Neapolitan area. J Virtual Explor. 2010;36.
980 doi:10.3809/jvirtex.2010.00261
981 95. Jackson M, Marra F, Deocampo D, Vella A, Kosso C, Hay R. Geological observations of excavated sand
982 (harenae fossiciae) used as fine aggregate in Roman pozzolanic mortars. Journal of Roman Archaeology.
983 2007. pp. 25–54. doi:10.1017/s1047759400005304
984 96. Avanzinelli R, Lustrino M, Mattei M, Melluso L, Conticelli S. Potassic and ultrapotassic magmatism in the
985 circum-Tyrrhenian region: Significance of carbonated pelitic vs. pelitic sediment recycling at destructive
986 plate margins. Lithos. 2009;113: 213–227. doi:10.1016/j.lithos.2009.03.029
987 97. Boari E, Tommasini S, Laurenzi MA, Conticelli S. Transition from Ultrapotassic Kamafugitic to Sub-alkaline
988 Magmas: Sr, Nd, and Pb Isotope, Trace Element and 40Ar–39Ar Age Data from the Middle Latin Valley
989 Volcanic Field, Roman Magmatic Province, Central Italy. J Petrol. 2009;50: 1327–1357.
990 doi:10.1093/petrology/egp003
991 98. Milani L, Beccaluva L, Coltorti M. Petrogenesis and evolution of the Euganean Magmatic Complex, Veneto
992 Region, North-East Italy. Eur J Mineral. 1999;11: 379–400. doi:10.1127/ejm/11/2/0379

99. Albert PG, Tomlinson EL, Smith VC, Di Traglia F, Pistolesi M, Morris A, et al. Glass geochemistry of pyroclastic deposits from the Aeolian Islands in the last 50 ka: A proximal database for tephrochronology. J Volcanol Geotherm Res. 2017;336: 81–107. doi:10.1016/j.jvolgeores.2017.02.008

100. Jackson MD, Chae SR, Mulcahy SR, Meral C, Taylor R, Li P, et al. Unlocking the secrets of Al-tobermorite in Roman seawater concrete. Am Mineral. 2013;98: 1669–1687. doi:10.2138/am.2013.4484

101. Marra F, D'Ambrosio E, Gaeta M, Mattei M. Petrochemical Identification and Insights on Chronological Employment of the Volcanic Aggregates Used in Ancient Roman Mortars. Archaeometry. 2016;58: 177–200. doi:10.1111/arcm.12154

102. Tomlinson EL, Arienzo I, Civetta L, Wulf S, Smith VC, Hardiman M, et al. Geochemistry of the Phlegraean Fields (Italy) proximal sources for major Mediterranean tephras: Implications for the dispersal of Plinian and co-ignimbritic components of explosive eruptions. Geochim Cosmochim Acta. 2012;93: 102–128. doi:10.1016/j.gca.2012.05.043

103. Peccerillo A. Campania volcanoes. Vesuvius, Campi Flegrei, and Campanian Volcanism. Elsevier; 2020. pp. 79–120. doi:10.1016/B978-0-12-816454-9.00005-5

104. Di Renzo V, Di Vito MA, Arienzo I, Carandente A, Civetta L, D'antonio M, et al. Magmatic History of Somma–Vesuvius on the Basis of New Geochemical and Isotopic Data from a Deep Borehole (Camaldoli della Torre). J Petrol. 2007;48: 753–784. doi:10.1093/petrology/egl081

105. Di Vito MA, Sulpizio R, Zanchetta G, D'Orazio M. The late Pleistocene pyroclastic deposits of the Campanian Plain: New insights into the explosive activity of Neapolitan volcanoes. J Volcanol Geotherm Res. 2008;177: 19–48. doi:10.1016/j.jvolgeores.2007.11.019

106. Pabst S, Wörner G, Civetta L, Tesoro R. Magma chamber evolution prior to the Campanian Ignimbrite and Neapolitan Yellow Tuff eruptions (Campi Flegrei, Italy). Bull Volcanol. 2008;70: 961–976. doi:10.1007/s00445-007-0180-z

107. Pappalardo L, Civetta L, D'Antonio M, Deino A, Di Vito M, Orsi G, et al. Chemical and Sr-isotopical evolution of the Phlegraean magmatic system before the Campanian Ignimbrite and the Neapolitan Yellow Tuff eruptions. J Volcanol Geotherm Res. 1999;91: 141–166. doi:10.1016/S0377-0273(99)00033-5

108. Civetta L, Orsi G, Pappalardo L, Fisher RV, Heiken G, Ort M. Geochemical zoning, mingling, eruptive dynamics and depositional processes — the Campanian Ignimbrite, Campi Flegrei caldera, Italy. J Volcanol Geotherm Res. 1997;75: 183–219. doi:10.1016/S0377-0273(96)00027-3

109. Pappalardo L, Civetta L, de Vita S, Di Vito M, Orsi G, Carandente A, et al. Timing of magma extraction

during the Campanian Ignimbrite eruption (Campi Flegrei Caldera). J Volcanol Geotherm Res. 2002;114: 479–497. doi:10.1016/S0377-0273(01)00302-X

110. Civetta L, Gallo G, Orsi G. Sr- and Nd-isotope and trace-element constraints on the chemical evolution of the magmatic system of Ischia (Italy) in the last 55 ka. J Volcanol Geotherm Res. 1991;46: 213–230. doi:10.1016/0377-0273(91)90084-D

111. Orsi G, Civetta L, D'Antonio M, Di Girolamo P, Piochi M. Step-filling and development of a three-layer magma chamber: the Neapolitan Yellow Tuff case history. J Volcanol Geotherm Res. 1995;67: 291–312. doi:10.1016/0377-0273(94)00119-2

112. Arienzo I, Mazzeo FC, Moretti R, Cavallo A, D'Antonio M. Open-system magma evolution and fluid transfer at Campi Flegrei caldera (Southern Italy) during the past 5 ka as revealed by geochemical and isotopic data: The example of the Nisida eruption. Chem Geol. 2016;427: 109–124. doi:10.1016/j.chemgeo.2016.02.007

113. Cannatelli C, Lima A, Bodnar RJ, De Vivo B, Webster JD, Fedele L. Geochemistry of melt inclusions from the Fondo Riccio and Minopoli 1 eruptions at Campi Flegrei (Italy). Chem Geol. 2007;237: 418–432. doi:10.1016/j.chemgeo.2006.07.012

114. Di Vito MA, Arienzo I, Braia G, Civetta L, D'Antonio M, Di Renzo V, et al. The Averno 2 fissure eruption: a recent small-size explosive event at the Campi Flegrei Caldera (Italy). Bull Volcanol. 2011;73: 295–320. doi:10.1007/s00445-010-0417-0

115. Smith VC, Isaia R, Pearce NJG. Tephrostratigraphy and glass compositions of post-15 kyr Campi Flegrei eruptions: implications for eruption history and chronostratigraphic markers. Quat Sci Rev. 2011;30: 3638–3660. doi:10.1016/j.quascirev.2011.07.012

116. Tonarini S, D'Antonio M, Di Vito MA, Orsi G, Carandente A. Geochemical and B–Sr–Nd isotopic evidence for mingling and mixing processes in the magmatic system that fed the Astroni volcano (4.1–3.8 ka) within the Campi Flegrei caldera (southern Italy). Lithos. 2009;107: 135–151. doi:10.1016/j.lithos.2008.09.012

117. Fedele L, Insinga DD, Calvert AT, Morra V, Perrotta A, Scarpati C. 40Ar/39Ar dating of tuff vents in the Campi Flegrei caldera (southern Italy): Toward a new chronostratigraphic reconstruction of the Holocene volcanic activity. Bull Volcanol. 2011;73: 1323–1336. doi:10.1007/s00445-011-0478-8

118. Santacroce R, Cioni R, Marianelli P, Sbrana A, Sulpizio R, Zanchetta G, et al. Age and whole rock–glass compositions of proximal pyroclastics from the major explosive eruptions of Somma-Vesuvius: A review as a tool for distal tephrostratigraphy. J Volcanol Geotherm Res. 2008;177: 1–18.

doi:10.1016/j.jvolgeores.2008.06.009

119. Ayuso RA, De Vivo B, Rolandi G, Seal RR, Paone A. Geochemical and isotopic (Nd–Pb–Sr–O) variations bearing on the genesis of volcanic rocks from Vesuvius, Italy. J Volcanol Geotherm Res. 1998;82: 53–78. doi:10.1016/S0377-0273(97)00057-7

120. Paone A. The geochemical evolution of the Mt. Somma-Vesuvius volcano. Mineral Petrol. 2006;87: 53–80. doi:10.1007/s00710-005-0103-7

121. Sparice D, Scarpati C, Mazzeo FC, Petrosino P, Arienzo I, Gisbert G, et al. New proximal tephras at Somma-Vesuvius: evidences of a pre-caldera, large (?) explosive eruption. J Volcanol Geotherm Res. 2017;335: 71–81. doi:10.1016/j.jvolgeores.2017.02.004

122. Piochi M, Ayuso RA, De Vivo B, Somma R. Crustal contamination and crystal entrapment during polybaric magma evolution at Mt. Somma–Vesuvius volcano, Italy: Geochemical and Sr isotope evidence. Lithos. 2006;86: 303–329. doi:10.1016/j.lithos.2005.05.009

123. Somma R, Ayuso RA, De Vivo B, Rolandi G. Major, trace element and isotope geochemistry (Sr-Nd-Pb) of interplinian magmas from Mt. Somma-Vesuvius (Southern Italy). Mineral Petrol. 2001;73: 121–143. doi:10.1007/s007100170014

124. Joron J-L, Metrich N, Rosi M, Santacroce R, Sbrana A. Chapter 3. Chemistry and Petrography of Somma-Vesuvius. In: Santacroce R, editor. Monografie finali Somma-Vesuvius. 1987. pp. 105–174.

125. Cioni R, Sulpizio R, Garruccio N. Variability of the eruption dynamics during a Subplinian event: the Greenish Pumice eruption of Somma–Vesuvius (Italy). J Volcanol Geotherm Res. 2003;124: 89–114. doi:10.1016/S0377-0273(03)00070-2

126. Aulinas M, Civetta L, Di Vito MA, Orsi G, Gimeno D, Férnandez-Turiel JL. The “Pomici di Mercato” Plinian eruption of Somma-Vesuvius: magma chamber processes and eruption dynamics. Bull Volcanol. 2008;70: 825–840. doi:10.1007/s00445-007-0172-z

127. Wotzlaw J-F, Bastian L, Guillong M, Forni F, Laurent O, Neukampf J, et al. Garnet petrochronology reveals the lifetime and dynamics of phonolitic magma chambers at Somma-Vesuvius. Sci Adv. 2022;8. doi:10.1126/sciadv.abk2184

128. Sulpizio R, Cioni R, Di Vito MA, Mele D, Bonasia R, Dellino P. The Pomici di Avellino eruption of Somma-Vesuvius (3.9 ka bp). Part I: stratigraphy, compositional variability and eruptive dynamics. Bull Volcanol. 2010;72: 539–558. doi:10.1007/s00445-009-0339-x

129. Cioni R, Marianelli P, Sbrana A. Dynamics of the A.D. 79 eruption: stratigraphic, sedimentological and

geochemical data on the successions from the Somma-Vesuvius southern and eastern sectors. Acta Vulcanol. 1992;2: 109–123.

130. Cioni R, Civetta L, Marianelli P, Metrich N, Santacroce R, Sbrana A. Compositional Layering and Syn-eruptive Mixing of a Periodically Refilled Shallow Magma Chamber: the AD 79 Plinian Eruption of Vesuvius. J Petrol. 1995;36: 739–776. doi:10.1093/petrology/36.3.739

131. Marturano A, Aiello G, Barra D, Fedele L, Grifa C, Morra V, et al. Evidence for Holocenic uplift at Somma-Vesuvius. J Volcanol Geotherm Res. 2009;184: 451–461. doi:10.1016/j.jvolgeores.2009.05.020

132. Brown SK, Crosweller HS, Sparks RSJ, Cottrell E, Deligne NI, Guerrero NO, et al. Characterisation of the Quaternary eruption record: analysis of the Large Magnitude Explosive Volcanic Eruptions (LaMEVE) database. J Appl Volcanol. 2014;3: 5. doi:10.1186/2191-5040-3-5

133. Tomlinson EL, Albert PG, Wulf S, Brown RJ, Smith VC, Keller J, et al. Age and geochemistry of tephra layers from Ischia, Italy: constraints from proximal-distal correlations with Lago Grande di Monticchio. J Volcanol Geotherm Res. 2014;287: 22–39. doi:10.1016/j.jvolgeores.2014.09.006

134. Melluso L, Morra V, Guarino V, de' Gennaro R, Franciosi L, Grifa C. The crystallization of shoshonitic to peralkaline trachyphonolitic magmas in a H2O–Cl–F-rich environment at Ischia (Italy), with implications for the feeder system of the Campania Plain volcanoes. Lithos. 2014;210–211: 242–259. doi:10.1016/j.lithos.2014.10.002

135. Brown RJ, Orsi G, De Vita S. New insights into Late Pleistocene explosive volcanic activity and caldera formation on Ischia (southern Italy). Bull Volcanol. 2008;70: 583–603. doi:10.1007/s00445-007-0155-0

136. Poli S, Chiesa S, Gillot PY, Gregnanin A, Guichard F. Chemistry versus time in the volcanic complex of Ischia (Gulf of Naples, Italy): evidence of successive magmatic cycles. Contrib to Mineral Petrol. 1987;95: 322–335. doi:10.1007/BF00371846

137. De Astis G, Pappalardo L, Piochi M. Procida volcanic history: new insights into the evolution of the Phlegraean Volcanic District (Campania region, Italy). Bull Volcanol. 2004;66: 622–641. doi:10.1007/s00445-004-0345-y

138. McDonough WF, Sun S -s. The composition of the Earth. Chem Geol. 1995;120: 223–253. doi:10.1016/0009-2541(94)00140-4

139. Giuliani CF. L'edilizia nell'antichità. Ia ed. 199. Roma; 2006.

140. Wikander Ö. Handbook of Ancient Water Technology. Brill, Lei. 2000.

141. Antico Gallina M. Le pompe idrauliche di Mediolanum: dalle particolarità costruttive alla relazione con i

caratteri ambientali , n. Archeologi. In: Minonzio F, editor. Problemi di macchinismo in ambito romano Macchine idrauliche nella letteratura tecnica, nelle fonti storiografiche e nelle evidenze archeologiche di età imperiale. Archeologi. Como; 2004. pp. 125–142.

142. Magnani S, Bianchig P, P. V. Il ponte romano alla Mainizza e la via Aquileia-Emona. Aquielia Nostra LXXVI. 2005; 81–136.

143. Dilaria S, Marinello A, Zara A. Analisi archeometriche delle malte aeree e pozzolaniche del tempio di Esculapio. Risult Prelim Quad Norensi. 2022;9: 225–238.

144. Jackson MD, Vola G, Gotti E, Zanga B. Roman Sea-Water Concretes and their Material Characteristics. In: Oleson JP, editor. Building for Eternity: the History and Technology of Roman Concrete Engineering in the Sea. Oxbow Books; 2014. pp. 141–187.

145. Gotti E, Oleson JP, Bottalico L, Brandon C, Cucitore R, Hohlfelder RL. A comparison of the chemical and engineering characteristics of ancient roman hydraulic concrete with a modern reproduction of vitruvian hydraulic concrete. Archaeometry. 2008;50: 576–590. doi:10.1111/j.1475-4754.2007.00371.x

146. Oleson JP, Bottalico L, Brandon C, Cucitore R, Gotti E, Hohlfelder RL. Reproducing a Roman Maritime structure with Vitruvian pozzolanic concrete. J Rom Archaeol. 2006;19: 29–52. doi:10.1017/S1047759400006255

147. Rosada G. La direttrice endolagunare e per acque interne della DEcima Regio Maritima: tra risorsa naturale e organizzazione antropica. La venetia nell'area padano-danubiana Le vie di comunicazione Atti del convegno internazionale (Venezia, 6-10 aprile 1988). Padova; 1990. pp. 153–182.

148. Dilaria S, Previato C, Secco M, Busana MS, Bonetto J, Cappellato J, et al. Phasing the history of ancient buildings through PCA on mortars' mineralogical profiles: the example of the Sarno Baths (Pompeii). Archaeometry. 2022; 1–17. doi:10.1111/arcm.12746

149. Seymour LM, Tamura N, Jackson MD, Masic A. Reactive binder and aggregate interfacial zones in the mortar of Tomb of Caecilia Metella concrete, 1C BCE, Rome. J Am Ceram Soc. 2022;105: 1503–1518. doi:10.1111/jace.18133

150. Bugini R, Salvatori A, Capannesi G, Sedda AF, D'agostini C, Giuliani CF. Investigation of the characteristics and properties of the "cocciopesto" from the ancient Roman period, in Conservation of Stone and Other Materials. In: Thiel M-J, editor. Proceedings of the International RILEM/ UNESCO Congress. London; 1993. pp. 386–393.

151. Chiocchini U. The Roman cistern of road Cristoforo Colombo, Rome, Italy: operating mode and connection

1143 with the old Appian Way. Archaeol Anthropol Sci. 2019;11: 321–336. doi:10.1007/s12520-018-0606-x

1144

Figure

Click here to access/download;Figure;Fig_1.jpg

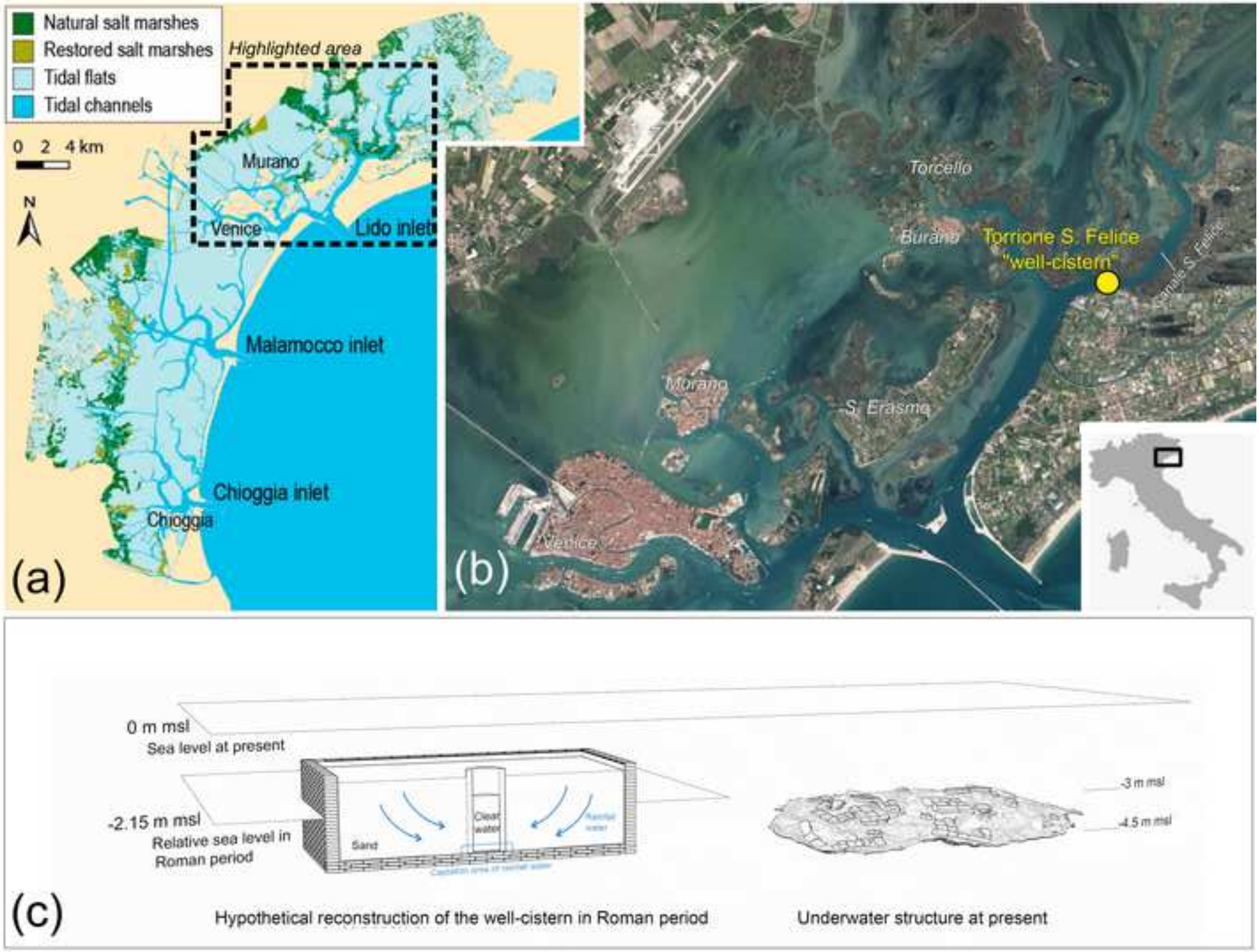

Figure

Click here to access/download;Figure;Fig_2.jpg

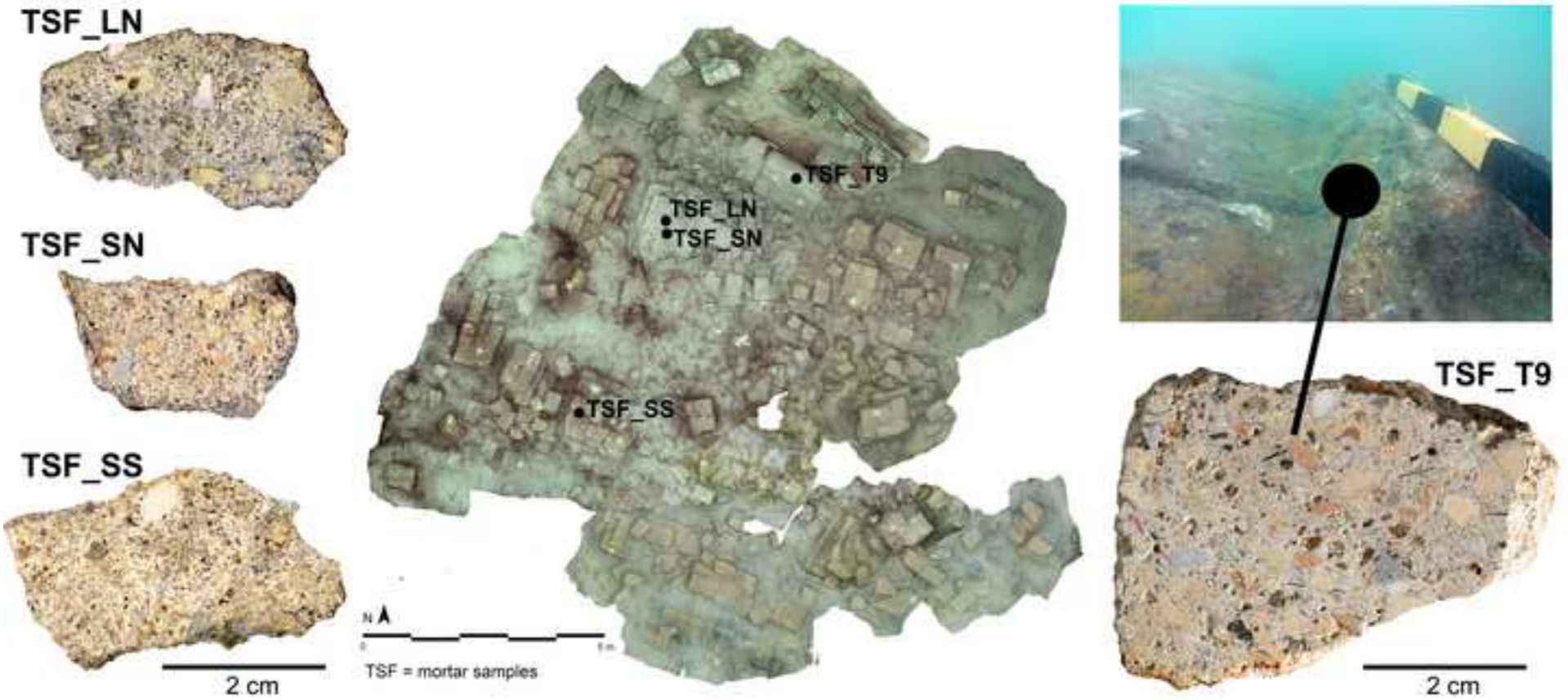

Figure

Click here to access/download;Figure;Fig_3.jpg

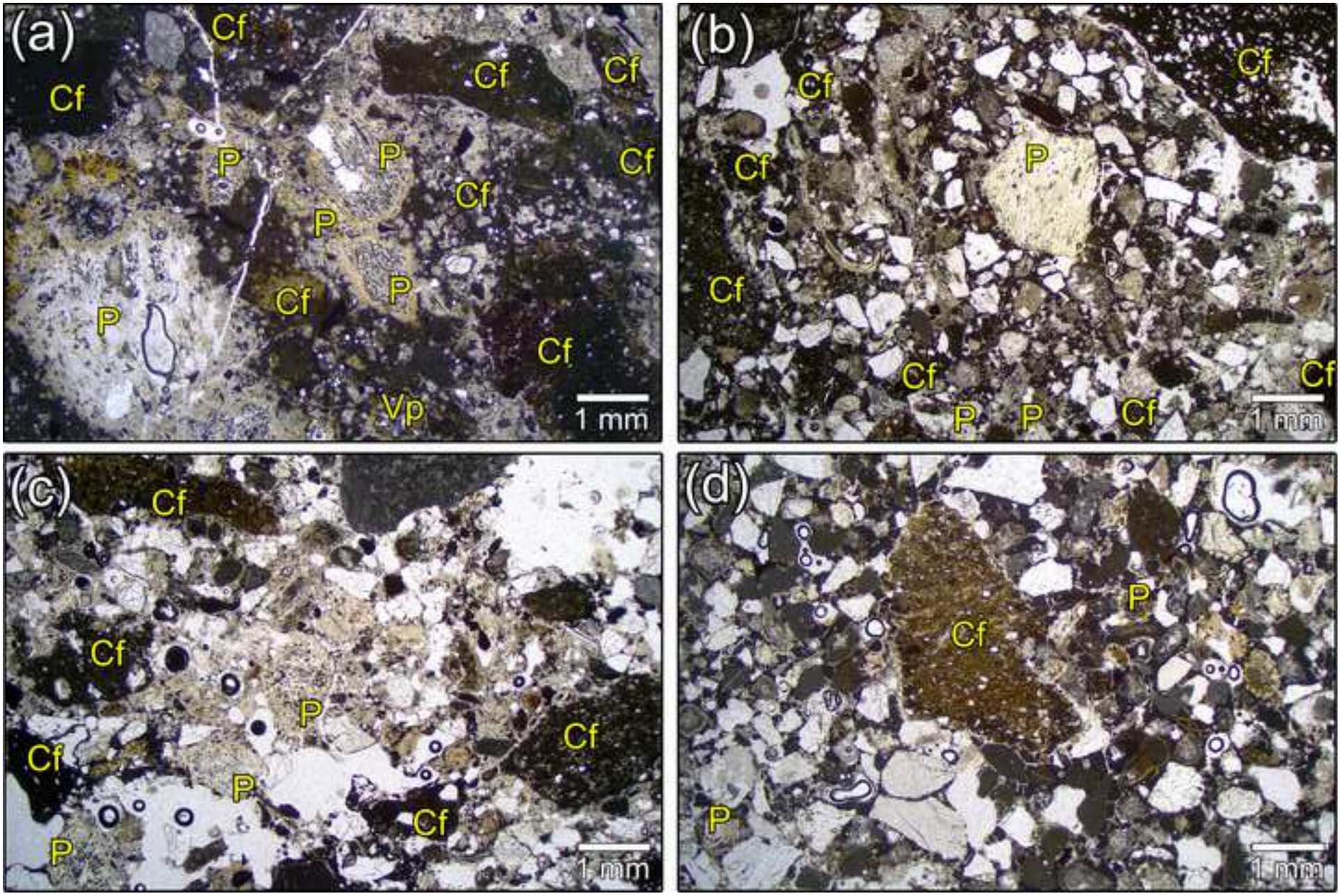

Figure

Click here to access/download;Figure;Fig_4.jpg

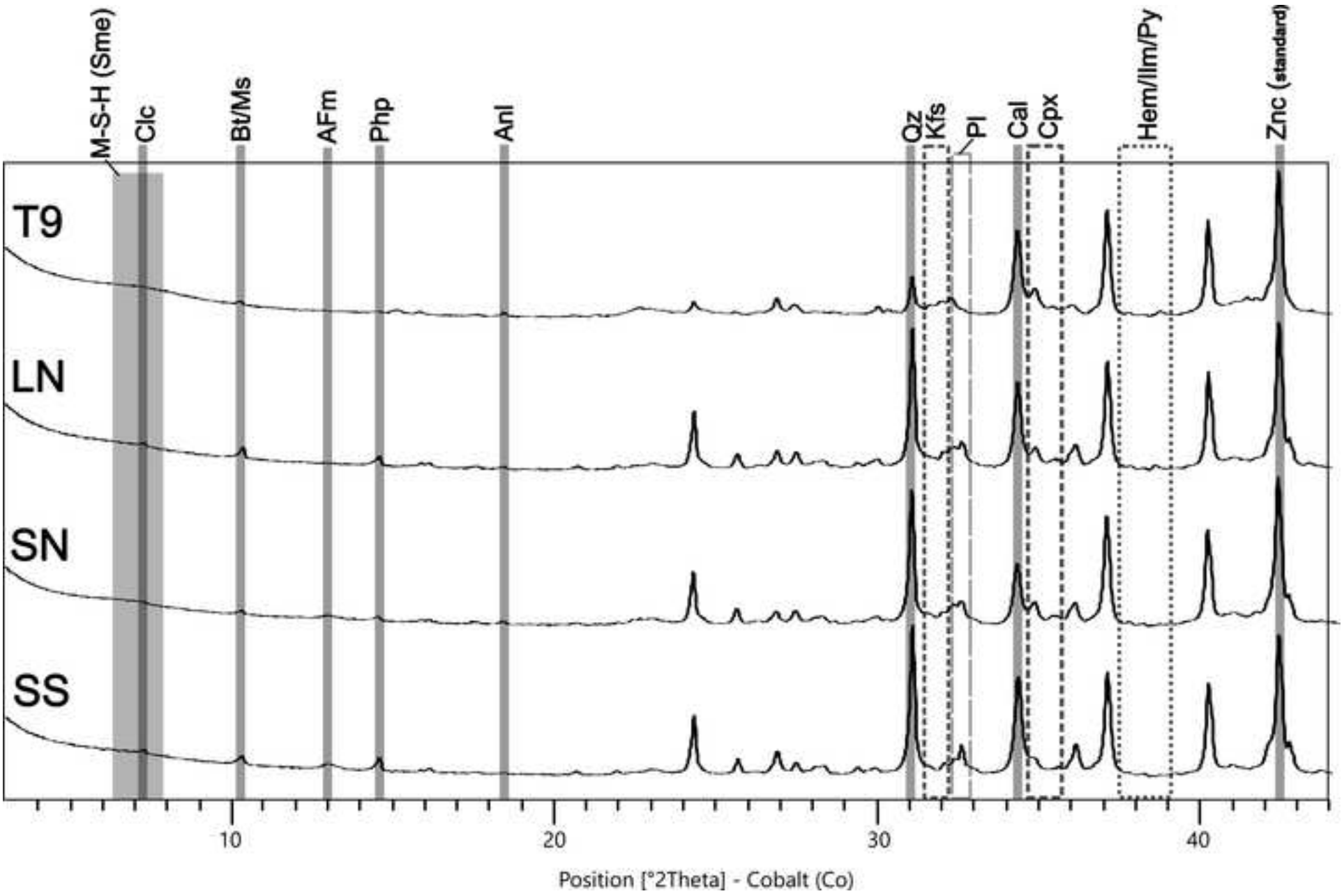

Figure

Click here to access/download;Figure;Fig_5.jpg

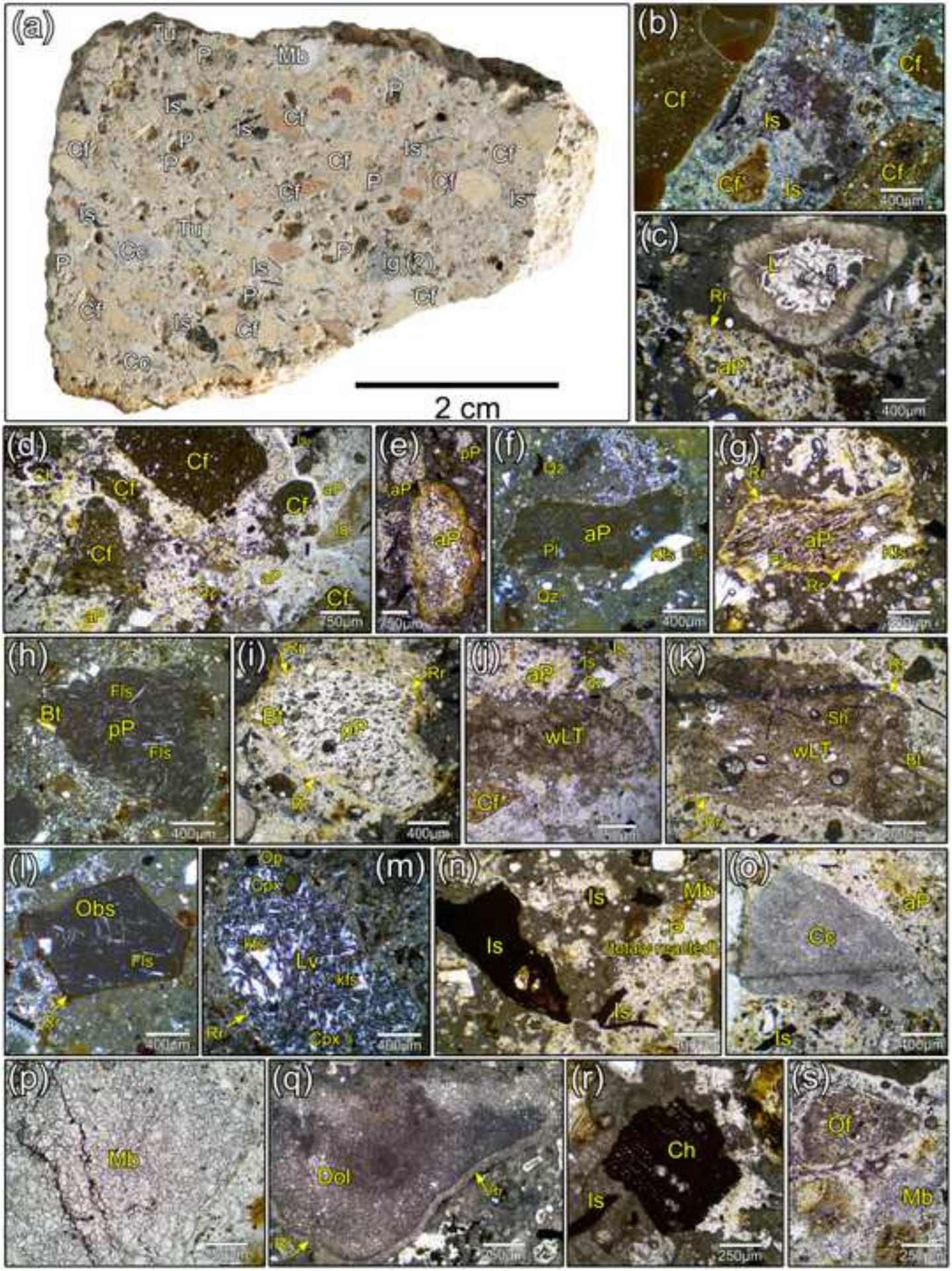

Figure

Click here to access/download;Figure;Fig_6.jpg

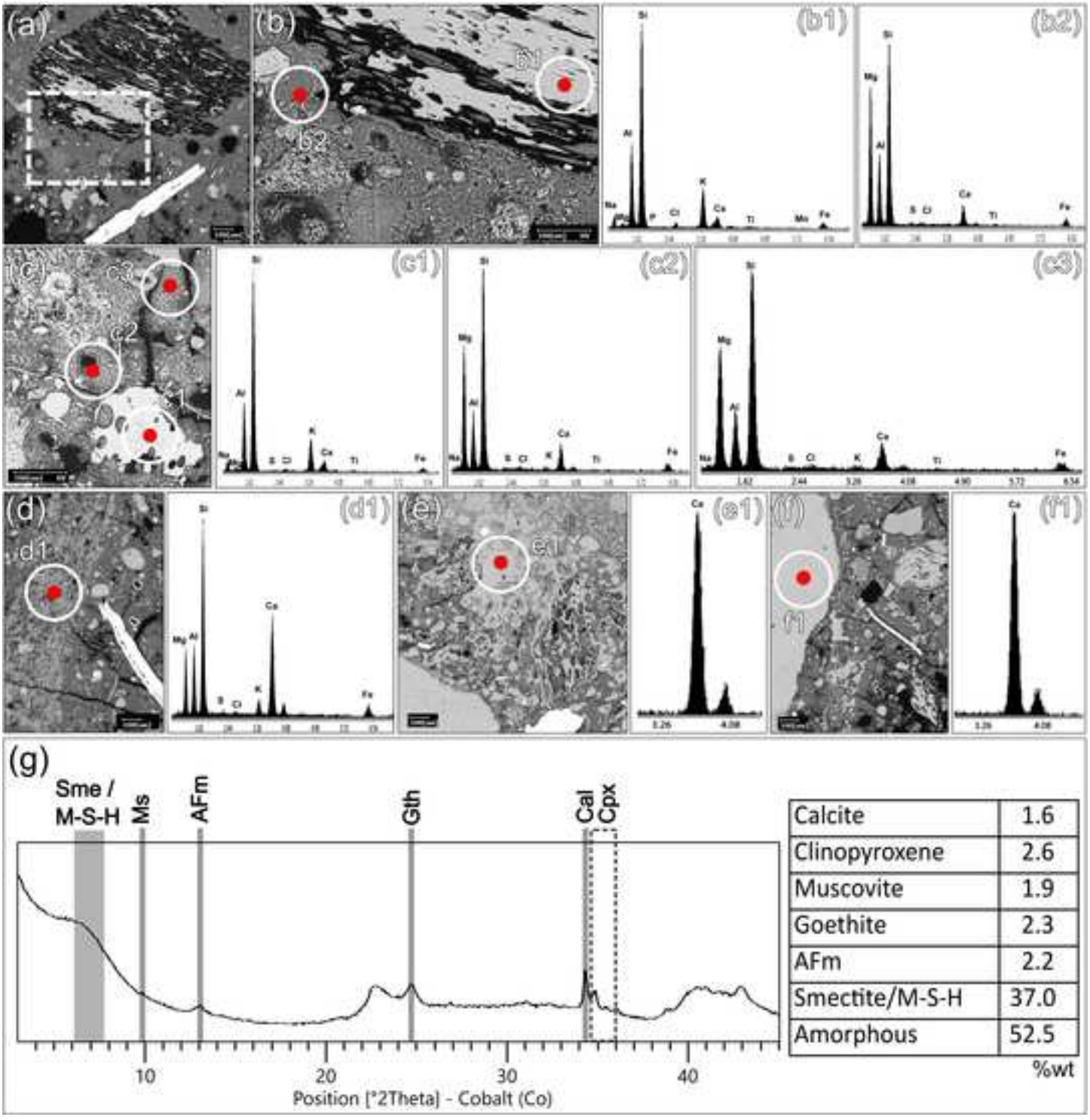

Calcite	1.6
Clinopyroxene	2.6
Muscovite	1.9
Goethite	2.3
AFm	2.2
Smectite/M-S-H	37.0
Amorphous	52.5

%wt

Figure

Click here to access/download;Figure;Fig_7.jpg

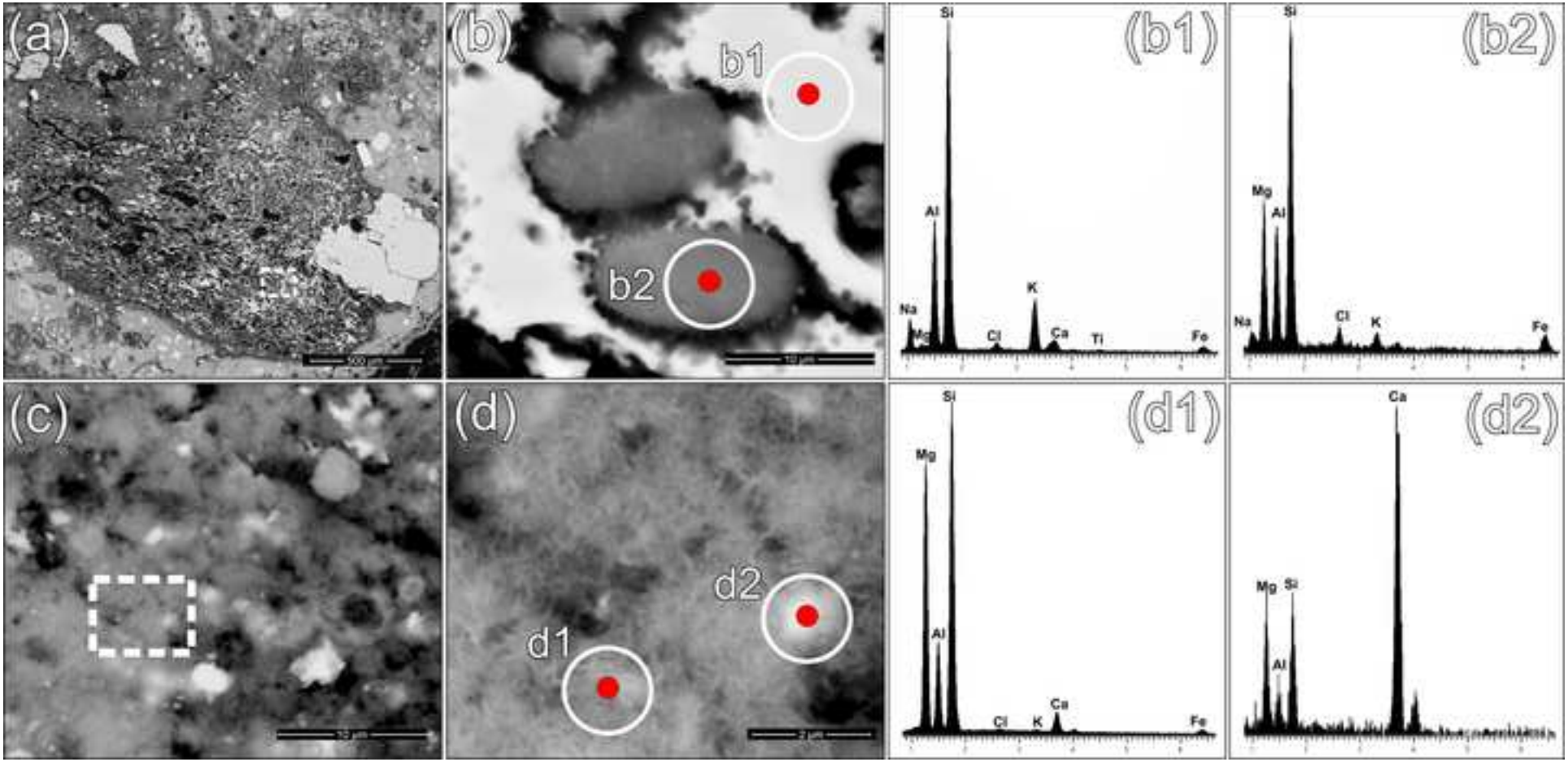

Figure

Click here to access/download;Figure;Fig_8.jpg

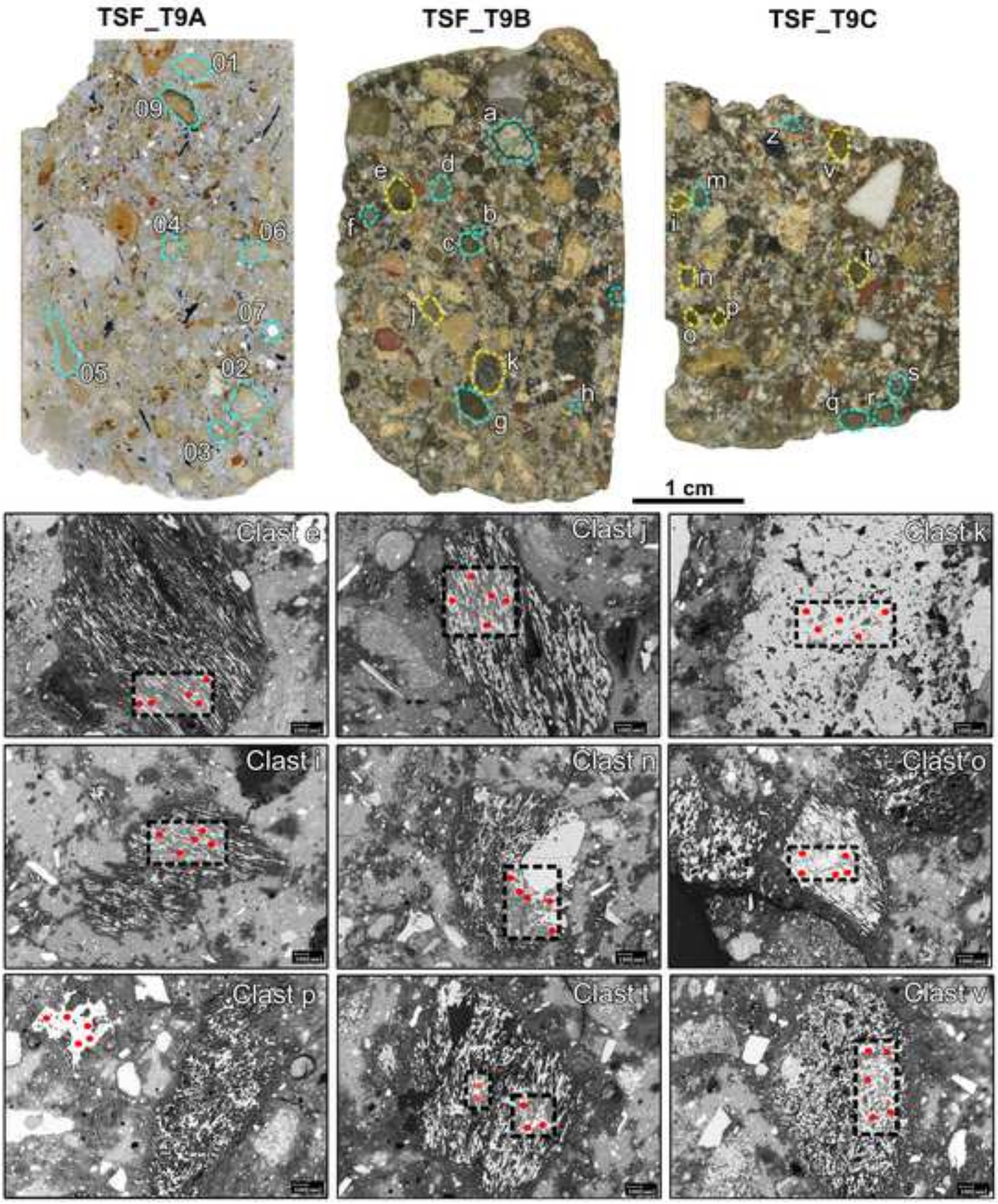

Figure

Click here to access/download;Figure;Fig_9.jpg

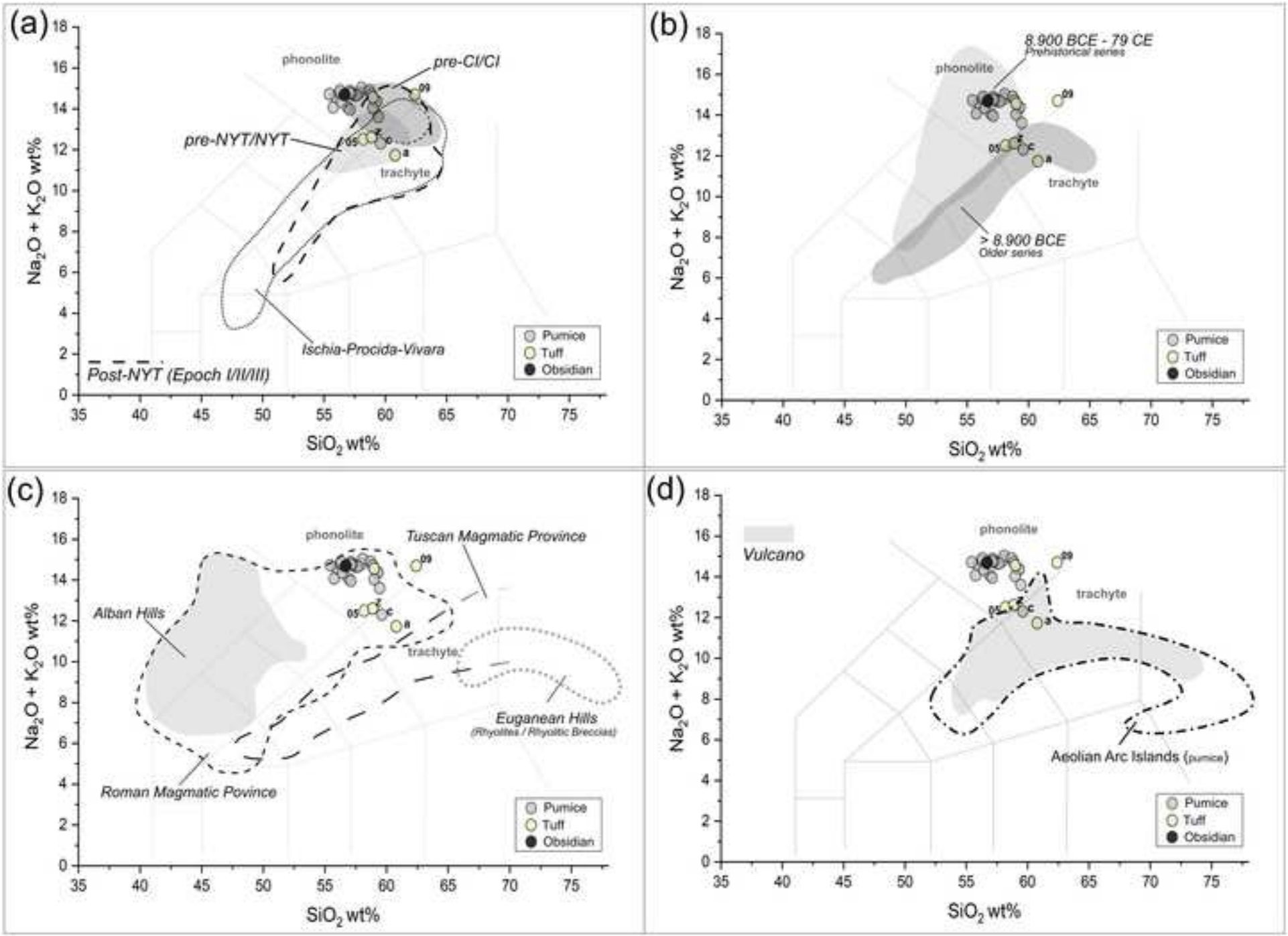

Figure

Click here to access/download;Figure;Fig_10.jpg

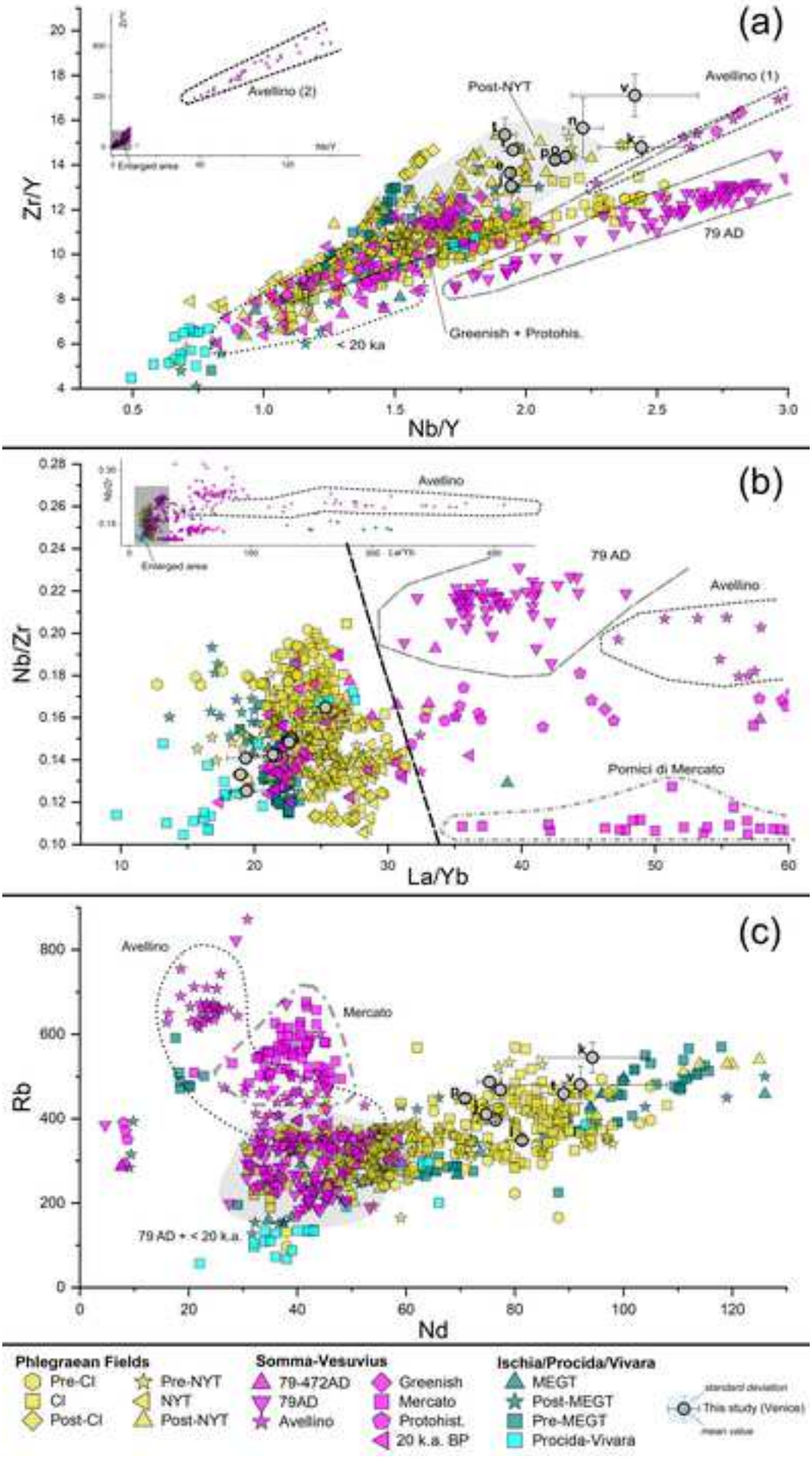

Figure

Click here to access/download;Figure;Fig_11.jpg

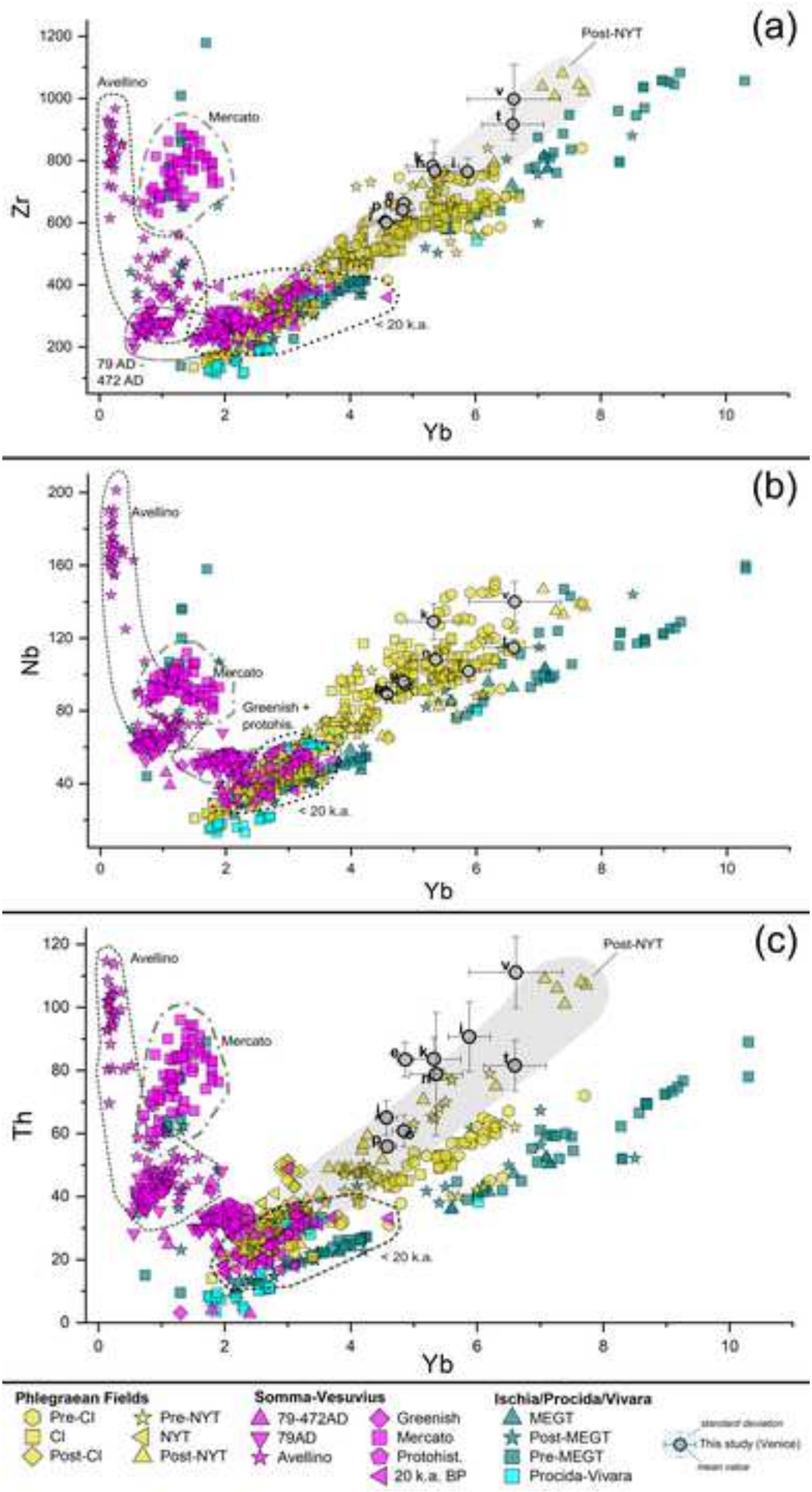

Figure

Click here to access/download;Figure;Fig_12.jpg

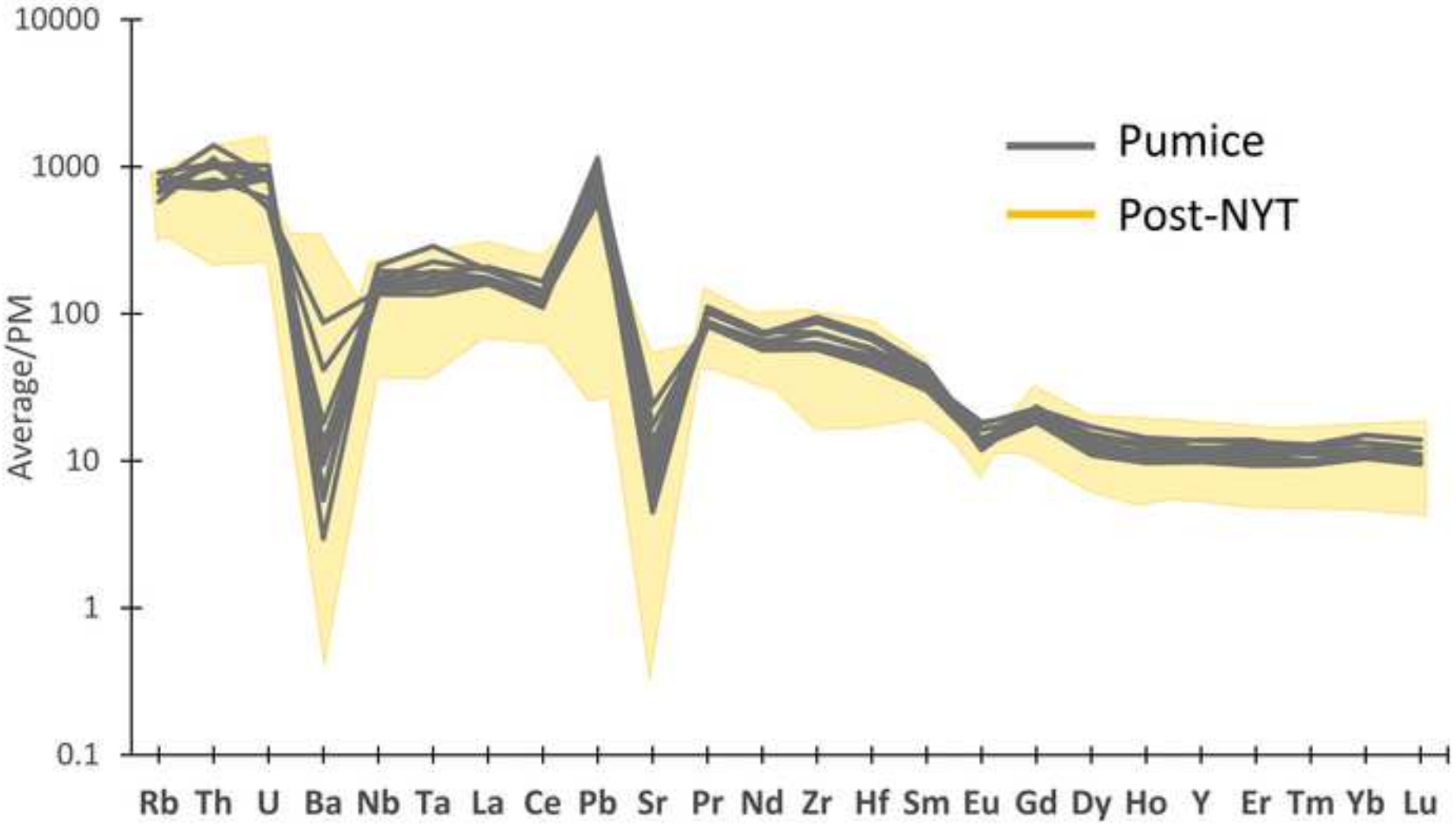

Click here to access/download
Supporting Information
Supporting_Information_Rev1.docx

Vitruvian binders in Venice: first evidence of Phlegraean pozzolans in an underwater Roman construction in the Venice Lagoon

Simone Dilaria[1,2,*], Giulia Ricci[2,3], Michele Secco[1,2], Carlo Beltrame[4], Elisa Costa[4], Tommaso Giovanardi[5], Jacopo Bonetto[1], Gilberto Artioli[2,3]

[1] Department of Cultural Heritage (DBC), University of Padova, ~~Piazza Capitaniato 7, 35139~~ Padua, Italy.

[2] Inter~~-~~Departmental Research Centre for the Study of Cement Materials and Hydraulic Binders (CIRCe), University of Padova, ~~Via Giovanni Gradenigo 6, 35131~~ Padua, Italy.

[3] Department of Geosciences, University of Padova, ~~Via Gradenigo 6, 35131~~ Padua, Italy.

[4] Department of Humanities, Ca' Foscari University Venice, ~~Dorsoduro 3484/D, 30123~~ Venice, Italy

[5] Department of Chemical and Geological Sciences, University of Modena and Reggio Emilia, ~~Via Campi 103, 411251~~ Modena, Italy

[*] Corresponding author (simone.dilaria@unipd.it)

Field Code Changed

Abstract

Four mortar samples were collected from a submerged Roman well-cistern (1st c. CE) in the N~~n~~northern part of the Lagoon of Venice, recently investigated during underwater surveys promoted by the team of maritime archaeology of the University Ca' Foscari of Venice. Samples were preliminary described following a standardized protocol of analytical techniques, including Polarized Light Optical Microscopy (PLM), Quantitative Phase Analysis - X-Ray Powder Diffraction (QPA-XRPD) and Scanning Electron Microscopy (SEM) coupled with Energy-Dispersive X-Ray Spectroscopy (EDS). Archaeometric analyses allowed the samples to be identified as lime-based mortars enriched with ceramic fragments and sand-sized particles compatible with local alluvial deposits~~local sands in variable concentrations.~~ . Moreover, pyroclastic aggregates, incompatible with the local geology, ~~from the Phlegraean fields (*pulvis puteolana*)~~ were added to the mortars to enhance their hydraulic capabilities~~ of the compounds~~. Their p~~P~~rovenance ~~determination of the volcanic pozzolans~~ was determined geochemically by analysing, via

SEM-EDS and Laser Ablation-Inductively Coupled Plasma-Mass Spectrometry (LA-ICP-MS), areas of apparently fresh volcanic glass, ~~unreacted cores of volcanic glass and by~~ outlining the actual geochemistry of magma's pristine composition within the analysed clasts. The resulting fingerprints were then compared~~ing the results~~ with the geochemical ~~profiles~~ distribution of the pyroclastic products of the main Italian Plio-Quaternary magmatic districts. ~~This operation was complicated by the significant reaction processes the analysed pozzolans were object of, seriously affecting their geogenic chemical and petro-mineralogical features.~~ The lithological source of the analysed tephra appears to be petrochemically congruent with the Phlegraean Fields volcanic district. Provenance determination was complicated by the significant reaction processes the analysed ~~pozzolans~~pyroclastic clasts were object of, seriously affecting their geogenic chemical and petro-mineralogical features. In fact, t~~T~~he strongly alkaline anoxic underwater environment of the Venetian lagoon likely fostered the kinetics, as the matrices showed a relevant development of M-S-H hydrates replacing the pristine Ca-bearing phases of the binder. On the other hand, ~~, suggesting that t~~the carbonation of the lime was almost null. It seems that ~~t~~T~~h~~he homogeneous ~~perfect~~ blend of local sands, ~~with~~ ceramic fragments and~~,~~ imported volcanic rocks ~~and~~ with brackish water promoted the development of pozzolanic and para-pozzolanic processes, ~~that influenced~~increasing the durability and ~~the perfect~~ preservation of the analysed mortars over time ~~to the present day~~. This evidence shows, once again, how the Vitruvian prescriptions on the utilization of Phlegraean pozzolans ~~as much as the literary passage by Vitruvius regarding Vitruvian mention to the prodigious effects of the "*pulvis puteolana* powder" (*pulvis*) to be used to enhance the physical and mechanical properties of used in seawater concretes~~ (Vitr., 5.12.2~~-3~~) were~~as~~ thoroughly grounded in the outcomes of ancient engineering knowledge, to enhance the physical and mechanical properties of seawater concretes.

~~1.~~ Introduction

The technological expertise achieved by humankind in the formulation of binding products from Antiquity ~~from the Prehistory~~ throughout time represents one of the most intriguing aspects in contemporary research on ancient mortar-based materials [1–4]. A specific interest was devoted by

Formatted: Highlight

Formatted: Normal, No bullets or numbering

scholars to the parametrization of the ~~hydraulic~~ physical-mechanical properties~~capabilities~~ of ancient recipes, in order to unveil the secrets of the ~~extreme~~ relevant durability and longevity of such composite construction materials.

In this framework, the use of certain aggregates, such as crushed ceramics, highly-amorphous volcanic rocks, organic ashes, metal slags or calcined clays [5] represents one of the most brilliant achievements of ~~the~~ ancient craftsmanship to improve the ~~hydraulic and~~ cohesive behaviour and waterproofing capabilities ~~of~~ of ~~aerial~~ lime-based mortars [6–11]. Under the term "pozzolanic reaction" we usually refer to the full set of chemical processes occurring when such materials – usually labelled as pozzolanic aggregates – are mixed with calcium hydroxide (portlandite) ~~(lime)~~ in ~~liquid solutions~~pastes. During the reaction, the silica and alumina compounds present in these ~~pozzolanic~~ reactive aggregates react with the ~~calcium hydroxide~~portlandite ~~of the lime pastes~~. This results in the formation of an array of anthropogenic phases ~~ordinarily~~ detected in ancient ~~hydraulic~~ mortars [12], having very variable stoichiometries, ranging from pure calcium silicate hydrates (C–S–H) to calcium aluminate hydrates (C–A–H) or complex C–A–S–H phases at increased Al activity. Such newformed phases are the main binding agents responsible for the strength, ~~and~~ durability and waterproofing capabilities of the resulting composite materials [13–18].

Nevertheless, the environmental conditions in which ~~the compounds~~ mortar-based mixtures are embedded also play an essential role in the development of reaction processes~~,~~ ~~as~~ As ~~the latent reactivity of pozzolanic aggregates appears to be favored in saltwater environments, since the alkaline and anoxic conditions promote the reaction, whereas lime carbonation is minimized.~~

~~Less t~~thoroughly investigated ~~in literatures~~ by [7], ~~is the fact that,~~ the latent reactivity of ~~pozzolanic~~pozzolanic aggregates appears to be favored in saltwater environments, since the alkaline and anoxic conditions promote the reaction, whereas lime carbonation is minimized. Moreover, under certain conditions of alkali, magnesium~~,~~ and chlorine activity, the hydraulic reaction can deviate from the typical lime-silica-alumina ternary diagram, favoring the precipitation of ~~to~~ other types of para-pozzolanic hydrate products, based on the interaction of magnesium with silica and~~or~~, sometimes, also with alumina. These processes usually ~~develop into~~lead to the formation of M-S-H/M-A-S-H phases (where M stands for MgO in cement chemistry notation)~~, having a~~ with a ~~variable~~ structurally

disordered phyllosilicate ~~to quasi gel~~-like structure [19–22], whose formation in ancient mortars is ~~being~~ now extensively recognized ~~properly recognized and parameterized only in the last years' research~~ [12,23–28].

Ancient builders formalized their empirical knowledge on the reactions occurring in mortar-based materials enhanced with ~~pozzolanic~~reactive aggregates in underwater environments. ~~In particular,~~ Vitruvius, in his *De Architectura* books on ancient architecture and building materials, made reference to a particular powder (*pulvis*)~~,~~ outcropping between~~between the vicinity of~~ *Baiae* in the Gulf of Naples and the territories surrounding Mount Vesuvius (*nascitur in regionibus Baianis et in agris municipiorum quae sunt circa Vesuvium montem*, Vitr. *De Arch*. 2.6.1). He observed that, once mixed with hydrated lime, such *pulvis* confers ~~lime-based mortars~~ remarkable properties (*res admirandas*) to lime-based mortars, as underlined by the Latin author. He firstly highlighted its unique capability to enhance the cohesion of structural concretes, as observed in [29], (*commixtum [pulvis] cum calce et caemento non modo ceteris aedificiis praestat firmitatem*, Vitr. *De Arch*. 2.6.1). Furthermore (*sed etiam*), this material was recommended for the construction of concrete piers in harbour infrastructures, as it enables the setting of concrete even in underwater environments [30,31] (*moles cum struuntur in mari, sub aqua solidescunt*, Vitr. *De Arch*. 2.6.1). In another part of his treaty, the Roman author reaffirmed the outcropping area of the powder within an area ranging from the territory of *Cumae* to the north, to the *promontorium Minervae* – to be identified with the Mounts Lattari in the Sorrentine peninsula [32] – to the south (*pulvis a regionibus quae sunt a Cumis continuatae ad promuntorium Minervae*, Vitr. *De Arch*. 5.12.2). ~~By not overly scrutinizing the slight spatial adjustments reiterated in the two sentences, m~~Modern geological research~~y~~ suggests a ~~geological~~ correspondence between the Vitruvian *pulvis* and medium-sized pumiceous tephra and weakly~~,~~ lithified tuff outcropping in the Neapolitan volcanic district and pertaining ~~to~~ both to the last Phlegraean eruptions (Neapolitan Yellow Tuff and post-Neapolitan Yellow Tuff) and the pre-79 CE eruptive events of Mount Somma-Vesuvius [33].

After Vitruvius, references to the "*pulvis*" become indeed recurrent in ~~the~~ ancient treaties. Pliny~~,~~ in his *Historia Naturalis* (~~,~~ published one or two years before the 79 CE eruption of Somma-Vesuvius), and Seneca~~,~~ in *Naturales quaestiones*, quoted the Vitruvian definition, ~~but~~ adding the adjective ~~of~~ *puteolanus* to the *pulvis*, thus identifying the main source deposits in the nearness of the ancient town of Puteoli,

Field Code Changed

Formatted: Font color: Auto

Formatted: Font color: Auto

Formatted: Font color: Auto

Formatted: Font: Italic

Formatted: Font: Italic

Formatted: Font color: Auto

Formatted: Font: Italic, Font color: Auto

Formatted: Font color: Auto

Formatted: Font: Italic

Formatted: Font: Italic

nowadays Pozzuoli in the Bay of Naples (*pulverem appellatam in Puteolanis collibus*, Plin. *NH* 35.166; *in ipsa turribus Puteolis e pulvere exaedificatis*, Plin. *NH* 16.202; *Quemadmodum Puteolanus pulvis*, Sen. *Q. Nat.* 3.20.3). Given the nearly century-long gap between the lives of Vitruvius and those of Pliny and Seneca, an hypothesis that has not yet evaluated by scholars is whether this specific reference to the sourcing area limited in the nearness of the *ager Puteolanus* could also indicate a centralization of the quarry sites for the material around the Phlegraean district, in the Gulf of Naples, rather than in the Somma-Vesuvius volcanic unit. Indeed, from the second half of the 1st c. BCE, the Phlegraean territory saw significant infrastructure development, implementing some of the Roman Empire's primary commercial and military harbours, such as those at Baia, Puteoli and Miseno [34,35]. These ports played a crucial role in facilitating the spread of the Neapolitan ~~pozzolanic material~~volcanic pozzolans for concrete constructions throughout the Mediterranean regions of the Roman Empire during the next two centuries. In fact, in agreement with the literary sources, from the second half of the 1st c. BCE, ~~actually~~ around the time when Vitruvius wrote the *De Architectura* for Octavian (~ 30 BCE), the Neapolitan *pulvis* started beinge~~n~~ traded out of the region ~~of natural procurement~~, monopolizing the markets in the following decades as the pivotal material ~~for the crafting of~~to produce durable ~~and~~ hydraulic mortar-based materials. Recent evidence confirms the widespread use of "pulvis puteolanus" for the realization of underwater harbor piers, whose utilization was documented in Late Republican ~~sports~~ tructures from both ~~the Tyrrenian~~Tyrrhenian and Adriatic sea~~s~~ [33]~~(Brandon et al. 2014; Marra et al. 2016)~~. Since the end of the 1st c. BCE, evidence of Phlegraean pozzolans utilization has been recorded in some of the major Roman harbors of the Eastern Mediterranean, such as that of Caesarea Marittima, built by Herod the Great around by the end of the 1st c. BCE, and that of Alexandria in Egypt [33,36,37]. Furthermore, evidence of utilization of the *pulvis puteolanus* for the structural reinforcement of masonry concretes for on-land buildings were documented in Roman constructions in the sites of the Gulf of Naples at least since the 2nd c. BCE [38–41]. The material was also traded and ~~utilized~~used in the Provinces of the Empire for the realization of concrete masonries and foundations of on-land ~~significant~~ urban public monuments, as indicated by recent findings from Aquileia in Northern Italy [42], Sardinia [29] and North Africa [43].

Formatted: Font: Italic
Formatted: Font: Italic
Formatted: Font color: Auto
Formatted: Font: Italic, Font color: Auto
Formatted: Font color: Auto
Formatted: Font: Italic, Font color: Auto
Formatted: Font color: Auto
Formatted: Font color: Auto
Formatted: Font: Italic, Font color: Auto
Formatted: Font color: Auto
Formatted: Font color: Auto
Formatted: Superscript
Formatted: Font color: Auto
Formatted: Font color: Auto
Formatted: Font color: Auto
Formatted: Font color: Auto
Formatted: Font color: Auto
Formatted: Font color: Auto
Formatted: Font color: Auto
Formatted: Font color: Auto
Formatted: Font color: Auto
Formatted: Font color: Auto
Formatted: Font color: Auto
Formatted: Font color: Auto
Formatted: Superscript
Formatted: Superscript
Formatted: Font color: Auto
Formatted: Font: Italic, Font color: Auto
Formatted: Font color: Auto
Formatted: Font color: Auto
Formatted: Font color: Auto
Formatted: Font color: Auto
Formatted: Superscript

During the Late Imperial Age, mentions to the ~~"~~*pulvis puteolanus*~~"~~ were reported by Fulgenzio (*De Aet Mund*. 3.138) ~~lived around~~in the 5th c. CE, and by Isidorus of Seville (*Etym*. 16.1.8)~~,~~ in the 7th c. CE [44]. The frequent appearance of the term in ancient sources underlines its notoriety as building material in Roman times, persisting ~~into~~until the early Middle Ages. However, archaeological evidence of the trading of *pulvis puteolanus* after the first decades of the 4th c. CE (or even after the first Tetrarchy) are currently lacking. Proof of utilisation of imported Phlegraean pumices, used as pozzolanic and light-weighting materials on~~of~~ concrete vaults, ~~come from of~~has been found in the ~~large~~ Diocletian Baths in Rome [45], and in~~from~~ the Great Baths of~~from~~ Aquileia [46], ~~whose foundation was~~ recently dated to the Diocletian period [47]. From the second half of the 4th c. CE, the economic and commercial crisis that affected the Empire likely led to a substantial drop in the exportation of Neapolitan volcanic pozzolans in the Mediterranean.

In this research, a new evidence of mortars enriched with volcanic tephra in submerged environments is reported. S~~s~~amples ~~under study~~ were collected from a Roman well-cistern nowadays preserved in the northern ~~Lagoon of~~ Venice Lagoon, recently investigated by the team of maritime Archaeology of the University Ca' Foscari Venice, led by C. Beltrame [48]. This is a peculiar system for collecting and storing freshwater, an ancestor of the Venetian-style well ("Pozzo alla Veneziana") developed in Venice during the Medieval and Modern Ages [49], but originating in Roman times.

Formatted: Font color: Auto, Italian (Italy)
Formatted: Font color: Auto, Italian (Italy)
Formatted: Font color: Auto, Italian (Italy)
Formatted: Font: Italic, Italian (Italy)
Formatted: Font: Italic, Italian (Italy)
Formatted: Font: Italic, Font color: Auto, Italian (Italy)
Formatted: Font: Italic, Italian (Italy)
Formatted: Font: Italic, Font color: Auto, Italian (Italy)
Formatted: Font: Italic, Italian (Italy)
Formatted: Italian (Italy), Superscript
Formatted: Font: Italic, Italian (Italy)
Formatted: Italian (Italy), Superscript
Formatted: Font color: Auto, Italian (Italy)
Formatted: Font color: Auto, Italian (Italy)
Formatted: Font color: Auto
Formatted: Font: Italic
Formatted: Superscript
Formatted: Font color: Auto
Formatted: Font color: Auto

that in geological terms relates to the mainly unlithified pyroclastic ash deposits (namely pumices and feebly lithified tuffs) of the Neapolitan volcanic district, comprising both the last Phlegraean (Neapolitan Yellow Tuff and post-Neapolitan Yellow Tuff events) and Mt. Somma-Vesuvius pre-79 CE eruptive events [29].and Pliny (Pliny 16.202; 35.166) exalted the outstanding properties of the *pulvis puteolana*, referred to a prodigious powder outcropping around the modern town of Pozzuoli (Bay of Naples) that in geological terms relates to the mainly unlithified pyroclastic ash deposits (namely pumices and feebly lithified tuffs) of the Neapolitan volcanic district, comprising both the last Phlegraean (Neapolitan Yellow Tuff and post-Neapolitan Yellow Tuff events) and Mt. Somma-Vesuvius pre-79 CE eruptive events [29]. These Latin authors indicated the use of *pulvis puteolana* for enhancing the longevity of seawater concrete [30,31]. In perfect agreement with the literary source, from the Augustan Age (beginning of the 1st c. CE) onwards, this product obtained the monopoly in the markets as the topmost material for the manufacture of long-lasting hydraulic mortar-based materials for underwater port infrastructures [28,32–36].

In this research, new evidence of mortars enriched with Neapolitan volcanic pozzolans in submerged environments is reported. Samples under study were collected from a Roman well-cistern nowadays preserved in the Norther Lagoon of Venice, recently investigated by the team of maritime Archaeology of the University Ca' Foscari Venice, led by C. Beltrame [37]. The samples were preliminary analysed adopting a multi-analytical protocol for the characterization of the main petro-mineralogical features of the compounds, including Polarized Optical Microscopy (PLM), Quantitative Phase Analysis – X-Ray Powder Diffraction (QPA-XRPD) and Scanning Electron Microscopy (SEM) coupled with Energy Dispersive Spectroscopy (EDS). Indeed, archaeometric analyses allowed the compounds to be identified as lime-based mortars enriched with ceramic fragments (*cocciopesto*), local sand and volcanic pozzolans in variable concentrations. Great interest concerned the investigation of the extraordinarily advanced reaction phenomena that significantly perturbed the original geochemical and petro-mineralogical characteristics of the pozzolanic aggregates. High aliquots of paracrystalline Mg-rich hydrated phases (M-S-H/M-A-S-H), likely related to para-pozzolanic reactions between the lime-based binder and Mg-

Formatted: English (United Kingdom)

Formatted: Font: (Default) Cambria

~~rich reactive aggregates [38] through a liquid medium [39], were detected. However, these complex reaction kinetics seriously complicated the determination of the provenance of the volcanic pozzolans. Their compatibility with the composition of the pyroclastic products of the Phlegraean Fields was geochemically established by analysing via SEM-EDS and Laser Ablation-Inductively Coupled Plasma-Mass Spectrometry (LA-ICP-MS) unreacted glassy cores of a selection of pozzolanic aggregates and by comparing the results with over 950 geochemical profiles of pyroclastic rocks of the Neapolitan volcanic districts selected from literature.~~

~~Our outcomes represent the first proof of Phlegraean pozzolans in the Veneto region during the Roman era, which, not coincidentally, has been reported in an extremely fragile geomorphological context, still barely archaeologically explored, such as the Lagoon of Venice.~~

The archaeological context and mortar sampling

The site of the ~~so-called 'tower'~~ well-cistern ~~nowadays~~ is nowadays situated along the San Felice Channel ~~(**Figure 1**)~~ in the northern part of the ~~lagoon of~~ Venice Lagoon, a shallow ~~lagoon~~ submerged area characterized by salt marshes, tidal flats and tidal channels (**Fig 1a-b**). ~~The~~ According to the most recent geomorphological reconstructions, ~~proposed in **Figure 1**, shows that, compared with nowadays,~~ the 'well-cistern' ~~, in antiquity,~~ was originally positioned much closer to the coastal edge between the lagoon and the Adriatic Sea [50]. ~~nowadays~~Currently, it lies at a depth ranging from -4.5 to -3.0 m MSL, rising around 1.5 m above the bottom of the channel ~~bottom~~. Nevertheless, ~~but~~, on the basis of the photogrammetric survey and the quoted DEM of the structure (Digital Elevation Model), the depth of the structure suggests that it was partially placed underwater also in Roman times. In fact, even considering that the Roman relative sea level (RSL) in this part of the northern lagoon was about 2.15 m lower [51], the base of the well-cistern was surely positioned at a depth of around – 2.0 m MSL even in ancient times [48] (**Fig 1c**). ~~Torrione San Felice (TSF), located by side of the S. Felice Channel in antiquity, was much closer to the edge of the lagoon and with the Adriatic Sea.~~

The historical framework and constructive technique of the ~~TSF~~ structure was already accurately described in [48]: ~~nowadays, it lies at a depth ranging from -4.5 to -3.0 m MSL, rising around 1.5 m above~~

Formatted: English (United States)

~~the channel bottom, but, on the basis of the photogrammetric survey and the quoted DEM of the structure (Digital Elevation Model), the depth of the structure suggests that it was partially placed underwater also in Roman times. In fact, even considering that the Roman relative sea level (RSL) in this part of the northern lagoon was about 2.15 m lower [40], the base of the well-cistern was surely positioned at a depth of around −2.0 m MSL [37].~~ ~~t~~This is a flat quasi-squared box-like construction of around 6.7 × 7.7 m and entirely made, in both masonries and floors, of local bricks, measuring around 43.5–46 × 29.5–29 × 6 cm, which is the typical size of the *sesquipedali* of the Po Valley [52,53].

-Due to its shape, in previous studies (for example [54]) the structure was interpreted as a "defensive tower" (Torrione) dating to the Roman Imperial period. In the summer 2020, after the underwater surveys conducted by the team of Ca' Foscari University assisted by Venice's Idra company, the structure was reinterpreted as a Roman well-cistern. ~~It~~ This ~~typically~~ consists of a circular well shaft lined with bricks or stones, included in a squared masonry structure. The space between the well and the squared structure is then filled with river sand, employed for filtering and draining clean rain water to the bottom, where it is tapped by the well~~e; the entire reservoir is then filled with river sand, and on top of the sand, boxes or narrow channels are built with masonry vaults and open bottoms~~ (see **Fig 1c**).

~~, a peculiar system for collecting and storing freshwater, an ancestor of the Venetian-style well ("Pozzo alla Veneziana") developed in Venice during the Medieval and Modern Ages [44], but originating in Roman times with various pieces of evidence from the North-Eastern Adriatic territory (Aquileia, Musile di Piave and Ca' Ballarin/Treporti, **S1 Figure**).~~ The new interpretation of the San Felice structure as a well-cistern relies on several indicators. These include the preliminary identification of the use of waterproofing *cocciopesto* mortar renders, the assessment of the structure's base depth in relation to the relative sea level, the absence of groundwork poles in the soft lagoon mud – that are recurrent for building foundations of Roman period – and the strong similarities with the much better preserved well-cistern of Ca' Ballarin, located along the same channel not far from this site [48]. Various other pieces of evidence of such structures dated to Roman Times are present in the North-Eastern Adriatic territory (i.e. Aquileia and Musile di Piave, **S1 Figure**).

The dating of the ~~TSF~~ structure to~~in~~ the 1st c. CE, or little later, was determined on the basis of associated pottery finds [48].

In order tTo characterize the properties and composition of the peculiar hydraulic mortars employed in the structures, four mortar samples, labelled TSF (Torrione San Felice), were collected from coating renders and masonry joints of the structure during two underwater campaigns in 2020 and 2023 (**Figure 2).** All necessary permits were obtained for the described study, which complied with all relevant regulations. The sampling activity and the underwater archaeological survey in the Lio Piccolo site was authorized by the Soprintendenza Archeologia, Belle Arti e Paesaggio per l'area metropolitana di Venezia e le province di Belluno, Padova e Treviso. Samples were collected and stored in the laboratories of the Department of Geosciences of the University of Padova.

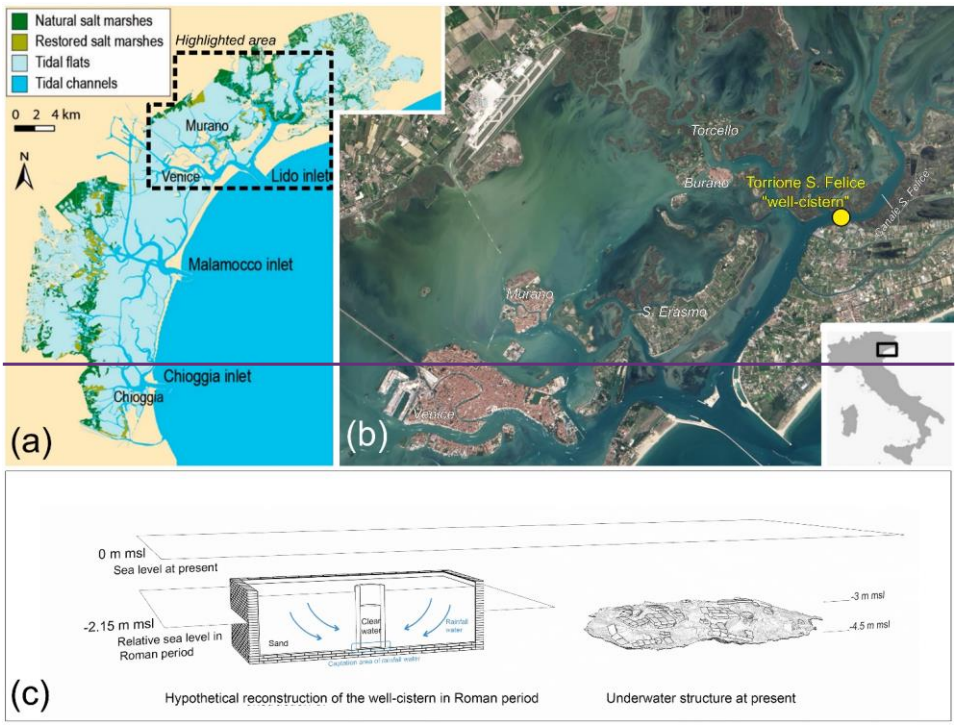

Fig ure 1. The site of Torrione San Felice in the Lagoon of Venice. (a) Reconstruction of the Lagoon of Venice with its main morphological features: salt marshes, tidal flats, and tidal channels (modified from [55] for illustrative purposes only); (b) Satellite image (taken from Landsat Image Gallery - https://landsat.visibleearth.nasa.gov) of the area highlighted in dashed black line in sub-figure (a), with positioning of the site of the Torrione San Felice well-cisternGeomorphological map of the northern lagoon of Venice (modified from [37]) with indication of the

~~site of the Torrione San Felice well-cistern. In the inset map, the site is georeferenced on a satellite sketch-map taken from Google Earth~~; (g) graphical hypothetical reconstruction of the original conformation of the San Felice well-cistern and relative sea-level in Roman times and at present.~~.~~

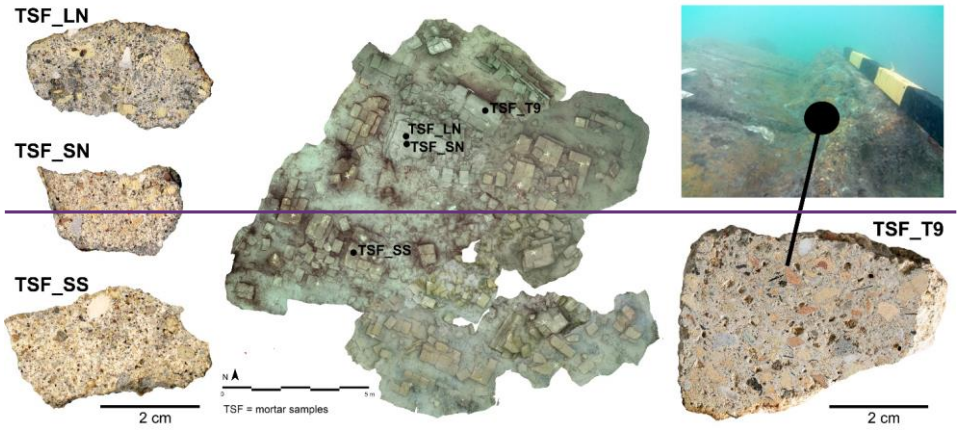

Fig~~ure~~ 2. Orthophoto of the submerged ~~Torrione~~ San Felice well-cistern at the end of 2020 survey, with indication of the mortars under study and their sampling points.

Multi-analytical protocol

TSF s~~S~~amples collected from the ~~TSF~~ structure were analysed according to a multi-analytical protocol designed for defining the raw materials employed in the compounds, the ~~peculiar~~ reaction phenomena occurring in submerged environment and, finally, the provenance of the volcanic tephra ~~volcanic pozzolans~~ included in the mortars (**S1 Analytical methods**).

All samples were firstly subjected to a full petro-mineralogical characterization, through detailed Polarized Light Optical Microscopy (PLM) investigations of mortars prepared on 30 μm thin sections. The characterization was accomplished in accordance with the standard UNI 11176:2006 “Cultural heritage - Petrographic description of a mortar” [56]. Furthermore, samples were mineralogically investigated by Quantitative Phase Analysis - X-Ray Powder Diffraction (QPA-XRPD). Bulk s~~S~~amples were

analysed after the addition ~~to the bulk fraction~~ of 20 wt% zincite as internal standard. Subsequently, the XRPD profiles were refined through the Rietveld method [57].

After this first screening, the most representative sample (TSF_T9) was subjected to further in-detail investigations aimed at parametrizing the reaction phenomena and the provenance of the volcanic tephra ~~natural pozzolans~~ included in the ~~compound~~material.

Scanning Electron Microscopy (SEM) coupled with Energy-Dispersive X-Ray Spectroscopy (EDS) analyses on both~~f the~~ binder matrices~~x~~ and aggregates' ~~aggregate's~~ reaction rims ~~of the sample~~ were functional at determining the ~~chemism~~ chemical activity of the ~~secondary~~ hydrate products occurring in the ~~peculiar~~ alkali-rich brackish-water ~~marsh water~~ environment of the Lagoon of Venice [58]. Moreover, QPA-XRPD analysis on the sole binder fraction of the sample allowed a mineralogical characterization of the reaction products ~~of~~ within the material ~~to be characterized also at a mineralogical level~~. Binder-concentrated aliquots were separated from the bulk mortar following the wet separation procedure [59,60]. The quantification of the mineralogical phases was determined by the addition to the separated-binder fraction of 20 wt% zincite as internal standard. Then, the profiles were processed adopting the same procedure previously defined for the bulk mortars.

The provenance of the volcanic ~~pozzolans~~ tephra was determined adopting a geochemical approach. After a detailed mineral-petrographic characterization under PLM, the investigation of the geochemistry of pyroclastic clasts was performed according to the analytical protocol reported in [42]. Firstly, their major elements' profiles were determined by analysing fresh unreacted areas of volcanic glass. The average values and standard deviations were calculated ~~on the basis of~~based on 5 up to 10 SEM-EDS semi-quantitative punctual analyses, performed on a 30 µm thin section (TSF_T9A) and on two 1 mm thick sections (TSF_T9B and TSF_T9C), prepared in order to be investigated via LA-ICP-MS ~~analysis~~. The sections were polished and carbon-coated before the analysis. LA-ICP-MS ~~analyses were~~was performed in order to collect the trace element profiles of a selection of ~~volcanic pozzolans~~juveniles already investigated by SEM-EDS. ~~In order to~~To avoid~~,~~ as much as possible~~,~~ the contamination with non-geogenic chemical elements (i.e. ~~reaction~~ pozzolanic hydrated phases), ~~a set of~~several clasts preserving portions of fresh volcanic glass fitting within the 55 µm laser ablation spot-size were selected for this part of the

research. For each clast, 4 up to 9 punctual LA-ICP-MS analyses were acquired, and the average values and standard deviations were calculated and reported.

~~The actual provenance of the clasts was determined by comparing on discriminant scatterplots.~~ Then, according to an already scientifically-established methodology [33,61–63], ~~a~~a selection of major and trace elements collected from the observed pozzolans was plotted in discriminant scatterplots in relation with the distribution of already-analysed geological samples related~~ing~~ to the main Plio-Quaternary magmatic activities of the Italian peninsula~~, according to an already scientifically-established methodology [29,36,50,51]~~. Moreover, by plotting in explorative scatterplots and spider-diagrams the chemical elements one-by-one, an updated pattern of elements was selected among REE (Rare Earth Elements) and HFSE (High Field Strength Elements) to discriminate among the differentiated magmatic activities occurred within the Neapolitan volcanoes. ~~In addition~~Finally, in order to check and verify the results inferred from scatterplot and spider-diagram distributions, we processed the geochemical data by discriminant analysis (DA). This is a multivariate statistical data reduction method frequently adopted to validate the classification of known data into categories or to assign unknown observations into such categories. This useful technique has been already applied in archaeology for assessing the provenance of artifacts of unknown origin by matching their descriptive variables with already classified markers [29,64–67].

Results ~~and Discussion~~

Characterization of raw materials

Formatted: Font: (Default) Cambria, 16 pt, Bold

Through PLM investigations, the TSF samples were characterized as lime-based mortars enriched with ceramic fragments and dust (*cocciopesto*), mixed with sands and ~~with~~ medium lapilli-sized pumiceous tephra in variable concentrations ~~local sands and volcanic pozzolans in variable concentrations~~ (**Tab 1**)~~.~~ Sample TSF_T9, collected from the joint coating between one wall and the floor of the well-cistern (socle), has the highest content of volcanic ~~pozzolans~~ tephra and ceramic fragments, easily detectable even at naked eye (**Fig~~ure~~ 3, a**). This is followed by sample TSF_LN, where the concentration of volcanic tephra and ceramic fragments ~~pozzolanic aggregates~~ appear~~s~~ less abundant than the previous one

(~~Figure~~Fig **3, b**). Finally, the last two samples, TSF_SS and SN, show ~~few traces~~a low amount of these components ~~of pozzolanic aggregates and~~ while the~~a~~ ~~higher~~ concentration of ~~alluvial~~ sand~~s~~, comprising angular monocrystalline quartz, micritic limestone and dolostones clasts, small granitoids and scattered plagioclases and micas of the muscovite type (**Fig 3, c-d**), is higher [68]. From a minero-petrographic point of view, their lithology is compatible with that of the local alluvial deposits, reworked in lagoon environments, with intermediate composition between the sediments of the lagoon bottom, seashore, and Piave and Brenta rivers [68]. ~~and other non-pozzolanic elements (**Figure 3, c-d**), mainly comprising angular monocrystalline quartz, micritic limestone and dolostones clasts, small granitoids and scattered plagioclases and micas of the muscovite type.~~

QPA-XRPD analyses highlighted these differences through a significant shift in the concentration of the phases correlated with the silicate fraction of the sand~~s~~, such as quartz and plagioclases (~~Figure~~Fig **4** and **Tab 1**). ~~-~~Another distinguishing marker is represented by the abundance of clinopyroxene of the diopside type, that is almost twice in TSF_T9 (11.4 wt%) in respect to the other samples. Its occurrence is related to the ceramic inclusions and it demonstrates the firing of Ca-rich clays at high temperature (>850 °C) [69].

Among the newformed pozzolanic phases, hydrated calcic ~~ferro-~~aluminates of the C-A-H type (AFm phases) [70] with crystal structures referable to those of calcium monocarboaluminate [71,72], ~~REF~~were detected in all the samples apart from TSF_T9. Its absence in ~~the most hydraulic-suspected sample of the dataset is intriguing and~~the latter probably rely on the fact that most of Si and Al ions did not react with Ca in a traditional Ca-based pozzolanic system, but with Mg, precipitating ~~into~~ Mg-rich hydrated compounds (M-S-H and M-A-S-H), as detailed in paragraph 4.2. In fact, broad low-angle peaks ascribable to poorly crystalline M-S-H/M-A-S-H phases presenting ~~the~~ a ~~peculiar~~ phyllosilicate-like structure of turbostratically-disordered smectite clays [12,19,26,73,74] were detected in high concentration in ~~the~~ sample TSF_T9. Finally, the presence of pyrite, detected by XRPD in most of the samples, is to be ascribed to post-depositional alteration phenomena of the materials, as a consequence of the action of sulfate-reducing bacteria, which promoted the dissolution of oxides and hydroxides in sulfur-rich alkaline aqueous solutions. The conditions for the proliferation of these bacteria are coherent with that of buried

Formatted: Highlight

contexts of anoxic lagoon-like environments, having abundant organic matter and poor water circulation [75–77].

As already stated, sample TSF_T9 was selected for a thorough analytical characterization, being the mortar with the highest concentration of pozzolanic aggregates and that showing the more complex reaction kinetics of the pozzolanic aggregates with the binder.

Under PLM observation, the binder of TSF_T9 appears extremely zoned, with a structure varying from micritic to microsparitic and ~~, with~~ diffused areas exhibiting very low birefringence colours (~~Figure~~**Fig 5, b**)~~, likely ascribable to the relevant precipitation of hydrate phases.~~

Lime lumps are few and scattered (~~Figure~~**Fig 5, c**), demonstrating a good mixing of the binding component. The aggregate is mainly constituted of poorly sorted ceramic elements (~~Figure~~**Fig 5, d**), with a grain-size distribution ranging from angular pluri-millimetric fragments to finely dispersed micrometric powder, less than 40 μm in size. The relevant milling probably contributed to the reactivity of the ceramic fraction [78,79].

Volcanic ~~rock~~ clasts constitute a slightly subordinate fraction of the aggregate in respect to the ceramic ones. They primarily consist in rounded-subrounded vesicular pumices with elongated or round pores and weak to compact density (~~Figure~~**Fig 5, e**). Under specific conditions, the reaction with the binder is pervasive to the extent that pozzolans become barely discernible, appearing as *ghosts* fully reacted within the matrix. Two families of pumices were distinguished, based on the texture of their groundmass: completely aphyric (glassy) juveniles (~~Figure~~**Fig 5, f-g**) fairly prevailing over feeble porphyritic ones (~~Figure~~**Fig 5, h-i**), presenting sparse feldspars microlites and biotite occasionally filling the intraclasts. The phenocrysts' assemblage is constituted by large crystals of ~~minerals of~~ K-feldspar~~s~~ (sanidine) prevailing over plagioclases, biotite and – occasionally – clinopyroxenes. Accessory minerals are composed of opaque minerals (iron oxides) and apatite (**S2** ~~Figure~~**Fig, a**). These juveniles are characterized by high porosity, deriving from the quick outgassing of magmatic volatiles in the course of explosive events [80]. Angular chips of weakly-lithified tuffs ~~ignimbrite tuffs~~ (~~Figure~~**Fig 5, j** and **k**) ~~and cutaxitic ignimbrites (Figure 5, k)~~ constitute a ~~totally~~ subordinate fraction of the volcanic aggregates. They are rich in mineral inclusions of biotite, k-feldspars (sanidine/orthoclase) and plagioclases, clay minerals, iron oxides, opaque minerals, glass shards and fragmented pumices

384 embedded in a cineritic matrix (**S2 ~~Figure~~Fig, b**). Finally, ~~elements of~~ compact vitrophyric obsidians
385 with scattered feldspars microlites (**~~Figure~~Fig 5, l**) were ~~very~~ occasionally observed together with dense
386 porphyritic lavas with k-feldspars and clinopyroxene phenocrysts (**~~Figure~~Fig 5, m**). It must be remarked
387 that in the other samples from the well-cistern, only pumices were detected among the volcanic ~~pozzolan~~
388 components.
389 From a minero-petrographic point of view, the volcanic ~~pozzolans~~ clasts detected in TSF samples appear
390 incompatible with the magmatism ~~local volcanic rocks~~ of the volcanic rocks of the Veneto Region
391 exploited in Roman times, substantially constituted by the dense effusive lava domes, dikes and
392 explosive breccias of the Euganean Hills ~~volcanic units~~district [81] and compact basalts and basanites of
393 the Berici Hills districts [82]. Therefore, this component of the aggregate is not local, and it was imported
394 from other regions, whose geographic position was tracked in paragraph 4.3.
395 Opaque millimetric shards and ground microclasts of ~~pure Fe~~oxidized iron slags (**~~Figure~~Fig 5, n**),
396 chemically characterized via SEM-EDS microanalysis (**S2 ~~Figure~~Fig, c**), were detected uniquely in
397 TSF_T9 sample, while they are absent in the others. They could act as tensile strengthening agents of the
398 binding composite, and they were likely added only to this sample to enhance the mechanical behaviour
399 of the compound [83], according to a practice already attested in ancient Greek and Roman times [84,85].
400 Their abundance suggests an intentional reuse of discarded processing debris of metalwork that
401 probably took place in the nearness of the site, but on which there are no pieces of evidence in the
402 archaeological record.
403 Finally, fragments of carbonate rocks were identified and they consist in sub-millimetric to pluri-
404 millimetric angular or sub-rounded chips of crystalline limestones (**~~Figure~~Fig 5, o**), metamorphosed
405 white marbles (**~~Figure~~Fig 5, p**), and occasional altered dolostones/dolomitic limestones (**~~Figure~~Fig 5,**
406 **q**), indicating de-dolomitization processes [86]. Furthermore, apart from monomineralic
407 quartz/carbonate sandy clasts, scattered monomineralic feldspars, clinopyroxenes, biotite and opaque
408 minerals were observed, probably deriving from the weathering of volcanic aggregates~~, were~~
409 ~~detected~~~~observed~~.
410 Among the additives, dispersed charcoal (**~~Figure~~Fig 5, r**) and organic unburnted ~~ted~~ fibres (**~~Figure~~Fig 5, s**)
411 were occasionally detected, both in samples TSF_T9 as well as in the other ones.

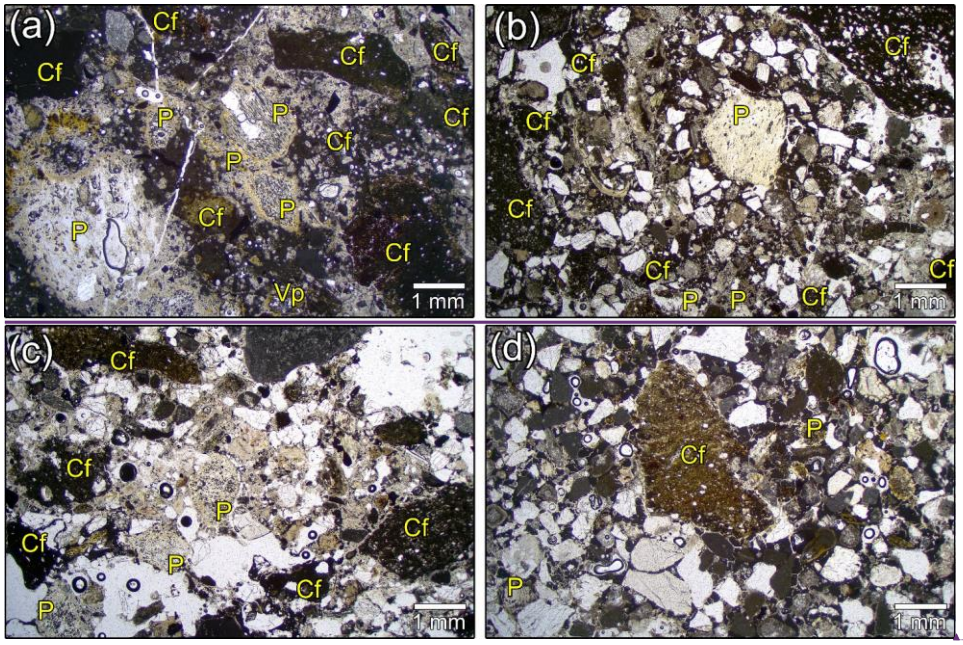

~~Figure~~Fig 3. Low-magnification PLM micrographs of TSF samples (transmitted light, plane polars). (**a**) TSF_T9; (**b**) TSF_LN; (**c**) TSF_SN; (**d**) TSF_SS. Legend: Vp = ~~Volcanic pozzolan~~Vesicular pumice; Cf = Ceramic fragments.

Formatted: Italian (Italy)

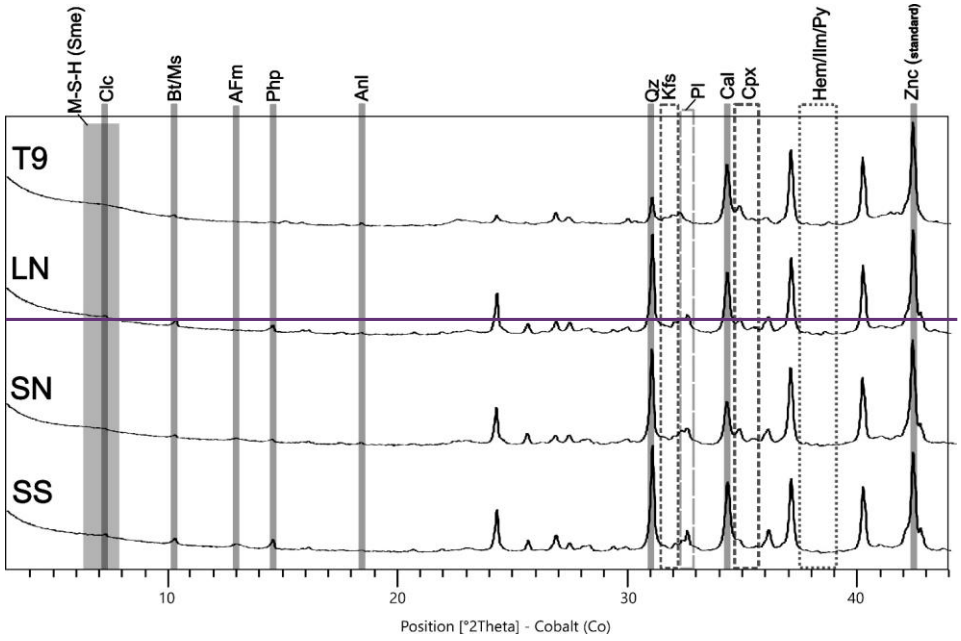

~~Figure~~Fig **4**. XRPD patterns of the four analysed TSF mortar samples. Mineral phases labelled according to [87] when mentioned: Clc = Celinochlore; Bt = Biotite; Ms = Muscovite; AFm = AFm phases of the Ca~~Mg~~-rich calcium monocarboaluminate~~hydrotalcite~~ type (authigenic process); Php = Phillipsite; Anl = Analcime; Qz = Quartz; Kfs = K-Feldspar; Pl = Plagioclase; Cal = Calcite; Cpx = Clinopyroxenes; Hem = Hematite; Ilm= Ilmenite; Py = Pyrite (post-depositional component); Znc = Zincite (~~used as~~ internal standard).

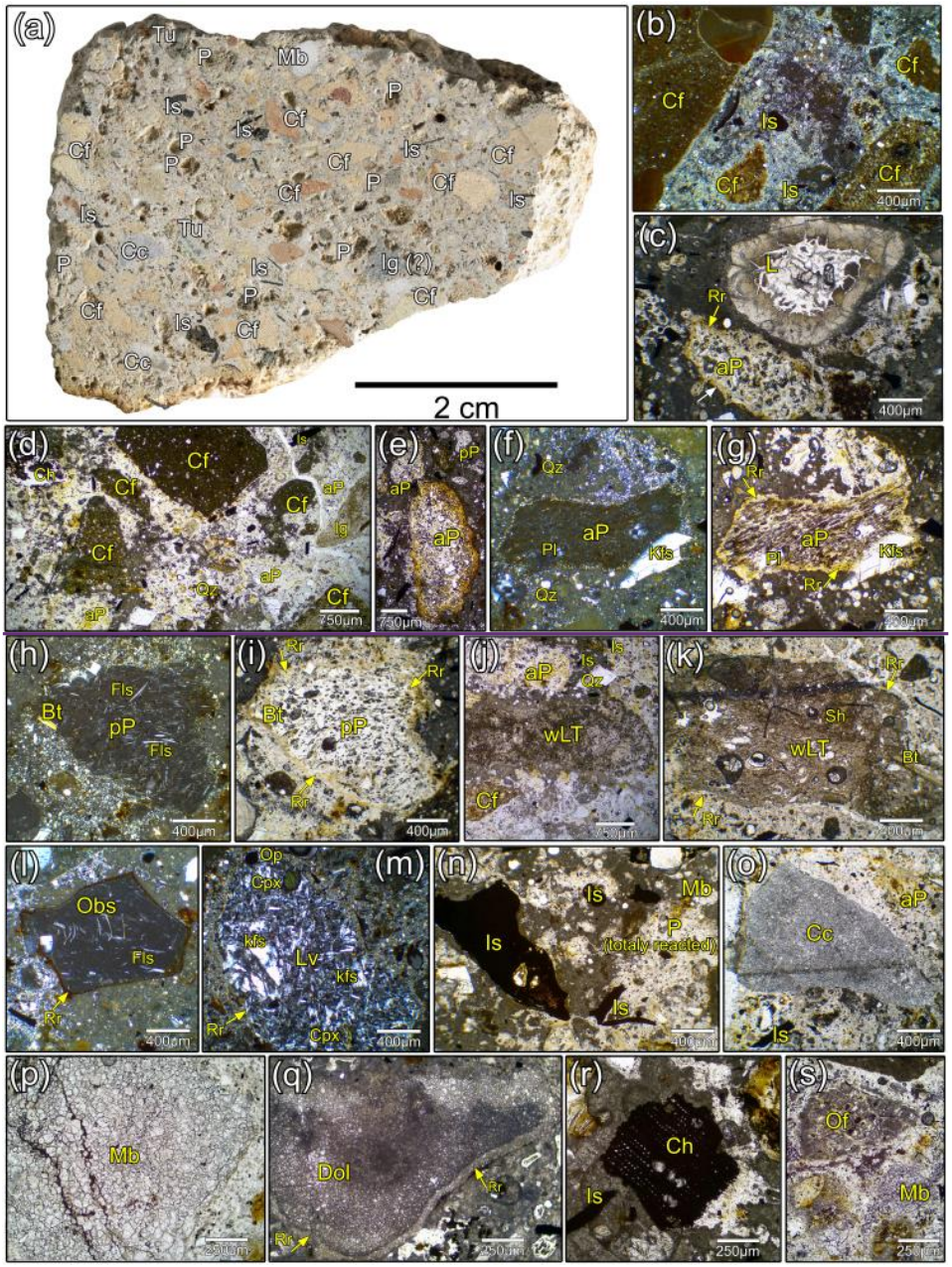

425

426 **~~Figure~~Fig 5.** In-detail mineral-petrographic characterization of TSF_T9. (a) Cross section of the sample, with

427 identification of the main aggregate components; PLM images of TSF_T9 sample, acquired in plane nicols (PN) and

crossed nicols (XN); (b) low-birefringence lime binder in an area rich in poorly sorted ceramic fragments (XN); (c) a lime lump surrounded by an outer rim showing low birefringence (PN); (d) the ceramic fragments' assemblage, mainly constituted by poorly sorted grains (PN); (e) feebly sorted sub-rounded clasts of pumice, with evident reaction edges of pozzolanic dissolution and reaction (PN); (f) and (g) a clast of aphyric pumice, with scattered phenocrysts of k-feldspars (sanidine) and plagioclase (XN and PN respectively); (h) and (i) a clast of pumice with a feebly-porphyritic texture, containing scattered feldspar microlites in a primarily glassy groundmass (XN and PN respectively); (j) an angular chip of weakly-lithified tuffs ~~tuff~~ with cineritic groundmass (PN); (k) a coarse fragment of weakly-lithified tuffs ~~eutaxitic ignimbrite~~ including volcanic shards, fragmented pumices and scattered biotite and feldspars (PN); (l) a small angular fragment of very dense vitrophyric obsidian, with sharp reaction rims (XN); (m) a clast of dense porphyritic lava with k-feldspars and clinopyroxenes phenocrysts (XN); (n) concentration of opaque fine-grained iron slags (PN); (o) an angular chip of crystalline limestone (PN); (p) a coarse clast of marble, composed of anhedral metamorphized crystals of calcite (PN); (q) a chip of dolostone with clear de-dolomitization edges (PN); (r) an organic charcoal inclusion (PN); (s) a small fragment of unburned organic fibre (PN). Legend – *Rock clasts and main aggregates*: P = pumice; aP = aphyric pumice; pP = porphyritic pumice; Lv = volcanic lava; wLT = weakly-lithified tuff; Obs = obsidian; ~~Tu = tuffs; Ig = ignimbrite;~~ Sh = volcanic shard; Mb = marble; Dol = dolostone; Cc = crystalline limestone; Cf = ceramic fragments; *Lime binder and reactions*: L = lime lump; Rr = reaction rim; *Minerals*: Bt = biotite; Fls = feldspar; Kfs = k-feldspar; Pl = plagioclase; Qz = quartz; Cpx = clinopyroxene; Op = opaque minerals; *Other inclusions*: Ch = charcoal; Of = organic fibre; Is = iron slag.

Table 1. Cumulative results of PLM and bulk QPA-XRPD analyses on the four mortar samples from ~~Torrione~~ San Felice well-cistern. Legend: b.d. = below detection; **- -** very low presence (<2%); **-** low presence (2-7.5%); **+** = moderate occurrence (7.5-20%); **++** = relevant occurrence (20-40%); **+++** = abundant occurrence (> 40%); LB = Lime binder.

**Sample**	**TSF_T9**	**TSF_LN**	**TSF_SN**	**TSF_SS**
**Function**	Coating (socle)	Joint mortar	Linings (?)	Linings (?)
**Description**	junction between masonry and floor	between two *sesquipedali* of the masonry	Flooring	Flooring
PLM				
**Binder texture**	LB	LB	LB	LB
**Lime Lumps**	**-**	**+**	**-**	**-**

**Voids**	-	+	++	+
**Local alluvial sands**	-	++	+++	+++
**Ceramic fragments**	+++	++	+	-
**Volcanic tephra~~Volcanic pozzolans~~**	++	+	-	-
**Crystalline Limestone chips**	+	+	-	-
**Iron slags**	+	b.d.	b.d.	b.d.
**Shells**	- -	-	- -	- -
**Fibers and charcoal**	- -	- -	-	- -
**estimated B:A prop.**	1:2	1:2	1:3	1:2
QPA-XRPD				
**Calcite**	19.5	19.2	11.7	26.1
**AFm**	b.d.	b.d.	0.3	1.3
**Dolomite**	b.d.	3.5	2.5	4.96
**Quartz**	2.4	21.7	19.4	25.8
**Plagioclase**	3.9	10.4	8.5	7.2
**K-feldspar**	4.2	4.7	4.1	3.5
**Microcline**	4.0	3.6	2.1	2.1
**Analcime**	0.9	0.4	0.4	0.2
**Phillipsite**	0.1	0.5	b.d.	0.6
**Clinopyroxene**	11.4	5.4	6.2	4.4
**Hematite**	0.5	0.3	0.3	0.3
**Ilmenite**	0.2	0.1	0.2	0.2
**Muscovite**	b.d.	2.3	2.3	3.0
**Biotite**	0.7	1.4	b.d.	0.7
**Clinochlore**	b.d.	3.7	2.2	4.1
**Pyrite**	b.d.	0.6	0.2	0.1

**Smectite/M-S-H**	26.0	b.d.	13.6	b.d.
**Amorphous**	26.1	22.4	26.1	15.6

Binder Characterization and Reaction in Submerged Environment

The reaction processes involving ~~pozzolanic~~ reactive aggregates and the lime-based binder ~~matrix~~ in TSF_T9 were investigated in-detail by SEM-EDS and QPA-XRPD analyses of the separated binder fraction. The backscattered electron images, as presented in ~~**Figure**~~**Fig 6**, show relevant interfacial dissolution features in most of the pozzolanic aggregates, both pyroclastic clasts ~~pumice~~ and ceramic fragments. Furthermore, pumice~~ous~~ grains are consistently dissolved by the reaction processes even in the cores (**Fig. 7, a**), with few surviving portions of fresh volcanic glass ~~volcanic glass~~ enriched in interfacial dissolution pits (**Fig. 7, b, b1**), and relevant filling of the outgassing vesicles by compact clusters of anhedral nanostructured particles with aluminosilicate-magnesian composition (**Fig. 7, b2**). The binding matrix is constituted by a tight network of intergrown nanostructured phases with pseudo-lamellar crystal habits (**Fig. 7, c, d**). ~~reveal that most of the pozzolanic aggregates are profoundly reacted with the binder.~~ The chemical composition of the binding~~er~~ matrix clearly indicates the presence of Si, Mg, Al, and, subordinately, Ca and Fe (~~**Figure**~~**Fig 6, a--c3**, **Fig. 7, d1**), apart from limited rounded sub-micrometric clusters enriched in calcium, to be ~~taken~~considered as relicts of the original lime calcium-based binder of the mortar (**Fig. 7, d2**). Such articulated chemical profiles~~which~~ are primarily attributed to a complex array of hydraulic phases that ultimately formed after pozzolanic reaction. In general terms, the factors triggering the widespread reactivity of these materials derive from a ~~complex~~n interplay of parameters that are influenced both by the intrinsic physicochemical properties of the pozzolanic aggregates and the environmental conditions of precipitation.

~~The backscattered electron images, as presented in **Figure 6**, reveal that most of the pozzolanic aggregates are profoundly reacted with the binder. The chemical composition of the binder matrix clearly indicates the presence of Si, Mg, Al, and, subordinately, Ca and Fe (**Figure 6, a--c3**), which are primarily attributed to a complex array of hydraulic phases that ultimately formed after pozzolanic~~

~~reaction. In general terms, the factors triggering the widespread reactivity of these materials derive from a complex interplay of parameters that are influenced by the intrinsic physicochemical properties of the pozzolanic aggregates.~~

QPA-XRPD analyses of the binder fraction showed the occurrence of very high aliquots of paracrystalline Mg-rich hydrated phases (M-S-H/M-A-S-H) as principal crystalline phase, up to 37.0 %wt (~~Figure~~Fig **6, g**), likely related to para-pozzolanic reactions between the lime-based binder and Mg-rich reactive aggregates [73]~~,~~ through a liquid medium [28]. These were mineralogically described using the structure of a turbostratically disordered smectite, as described at paragraph 4.1. ~~Moreover, t~~The relevant amorphous phase (up to 52.5 %wt)~~, displaying no diffraction reflexes,~~ may also be ~~mainly~~ related to development of these hydrate phases. It has been already noticed that salt-water plays a crucial role in facilitating the formation of M-S-H/M-A-S-H products. This is achieved by increasing the pH, which, in turn, promotes the dissolution of silicates and carbonates releasing Mg, Si, Al, ions in the system, which then combine to form Si-Al-Mg hydraulic phases [23,25,26,28,88]. As a matter of fact, the significant formation of M–S–H/M–A–S–H phyllosilicate gels is likely occurring when high concentrations of active magnesium, silica and alumina are present in the system. Whereas Al and Si were provided by the natural and artificial pozzolans, the sources of Mg in TSF_T9 within the aggregate fraction are less consistent and rely on the de-dolomitized dolostones and on certain Mg-rich ceramic fragments, exhibiting partially vitrified and reacted siliceous-aluminous matrices (~~Figure~~Fig **6, d-d1)**. However, the amount of active Mg occurring within the aggregate fraction is not sufficient to satisfy the abundant magnesium content detected by EDS analysis of matrices. The possible utilization of a magnesian binder must be rejected as confirmed by EDS analyses of the scattered lumps and calcination relicts, proving its original chemistry as eminently calcic (~~Figure~~Fig **6, e-e1, f-f1**).

Therefore, as already suggested in [28], it must be assumed that another relevant source of Mg was provided by the prolonged exposure to brackish water, having a significant concentration of magnesium ions (up to 3.7 %wt) in the overall amount of soluble salts [89].

Regarding the remaining phases, the QPA-XRPD shows the occurrence of reduced aliquots (2.2 %wt) of Mg-rich AFm phase of the hydrotalcite type [72], confirming the high degree of Mg activity in the system, together with a very low amount of calcite (1.6 %wt), to be ascribed to the anthropogenic $CaCO_3$ included

in the binder. This demonstrates that carbona~~ta~~tion of the binder was minimized whereas the available portlandite primarily undergone pozzolanic interchanges, being the reactivity of the ~~pozzolanic~~ ~~reactive~~pozzolanic aggregates fostered in the alkaline and oxygen-depleted subaqueous conditions. Moreover, the sample appears clearly Ca-depleted. In fact, the low-Ca rates ~~returned~~ highlighted by EDS analyses in the matrixes prove that the development of ordinary Ca-based hydraulic phases (C-S-H; C-A-S-H) was completely subordinated to Mg-based ones in such chemical environment. Therefore, under this condition, the hydraulic reaction occurred in TSF_T9 sample deviated from the conventional calcium-silica-alumina ternary diagram due to specific conditions of alkali, magnesium and chlorine activity.

The remaining phases detected with the QPA-XRPD analysis of the TSF_T9 binder fraction are negligible and mainly consist in mineral residues from the aggregate, as goethite (2.3 %wt), representing fine liquid-suspended particles deriving from the disaggregation of the iron shards included in the sample.

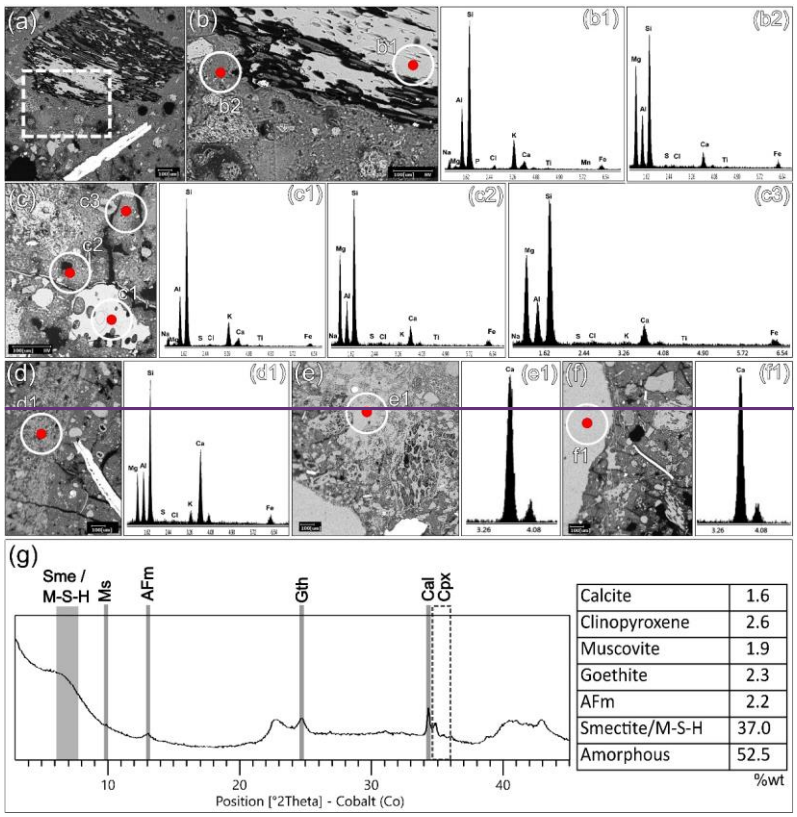

~~Figure~~Fig 6. Backscattered electron images and EDS microanalyses of ~~the~~ sample TSF_T9. (a) SEM acquisition of an area of sample TSF_T9; (b) enlarged picture of the area highlighted by the white dashed line in figure (a); (b1) EDS analysis of ~~an unreacted core~~apparently fresh unreacted volcanic glass within~~of~~ a pumice clast; (b2) Binder matrix of an area close to the pumice clast enriched in M-A-S-H; (c) pumice aggregate within the matrix of the sample; (c1) unreacted pumice clast; (c2 and c3) binder matrix enriched in M-A-S-H; (d) ceramic fragment; (d1) EDS analysis of the ceramic fragment; (e) lime lump; (e1) EDS analyses of the lime lump; (f) calcination relict; (f1) EDS analyses of the calcination relict; (g) high-magnification detail of the binder matrix's microstructure; (g1) EDS analysis of the investigated area; (hg) XRPD pattern of the separated binder fraction of sample TSF_T9. Mineral phases labelled according to [87] when mentioned: Ms = Muscovite; AFm = AFM phases; Cal = Calcite; Cpx = Clinopyroxenes; Gth = Goethite. The quantification of mineral phases after QPA analysis is shown to the right of the XRPD profile.

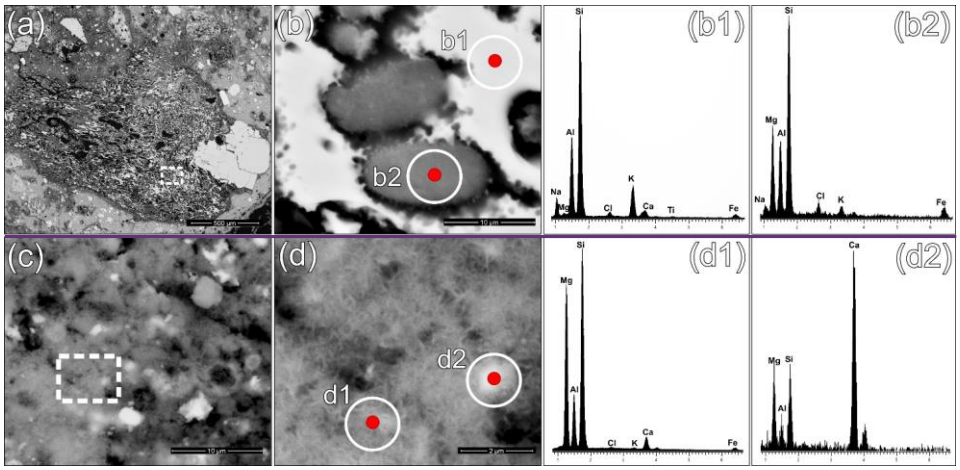

Fig 7. Backscattered electron images and EDS microanalyses of the sample TSF_T9. (a) SEM acquisition of a highly reacted pumice clast; (b) detail of the area highlighted by the white dashed line in figure (b1); EDS analysis of unreacted volcanic glass within the pumice clast; (b2) EDS analysis of the reaction products filling an outgassing vescicle of the pumice clast; (c) SEM acquisition of a portion of binding matrix; (d) detail of the area highlighted by the white dashed line in figure (c); (d1) EDS analysis of the portion of binding matrix highlighted in figure (d); (d2) EDS analysis of the rounded cluster highlighted in figure (d).

Provenance of ~~volcanic~~ pyroclastic clasts ~~pozzolans~~

Preliminary minero-petrographic and microchemical investigation of the ~~volcanic pozzolans~~pyroclastic clasts included in TSF samples allowed to establish their incompatibility with the ~~geology~~ magmatic products~~sm~~ of the Veneto region.

In order to determine the provenance of these extra-regional ~~pozzolans~~ juveniles on a geochemical basis, 29 volcanic clasts, comprising 23 aphyric and slightly porphyritic pumices, 54 weakly-lithified tuffs~~ignimbrite tuffs~~ and~~,~~ 1 vitrophiric obsidian ~~and 1 eutaxitic ignimbrite~~ (**S1 Table**), were preliminary investigated via EDS microanalysis on apparently fresh glass~~unreacted glassy/cineritic areas~~, still preserving the geogenic chemical fingerprint. This technique has been already adopted as a rapid and

efficient tool for a preliminary assessment of volcanic pozzolans' geochemistry included in ancient mortar-based materials [40,61,66,90].

~~For~~ As a first comparison between the analysed clasts and the geochemical intervals of the volcanic products of the main Italian Plio-Quaternary magmatic districts [91,92], the resulting major elements' profiles were plotted in the TAS (Total Alkali vs Silica) diagram [93], reporting the relation between alkaline elements (Na_2O + K_2O) and silica (SiO_2). In this diagram, most of the TSF_T9 ~~volcanic pozzolans~~pyroclastic clasts can be chemically categorized as phonolites. However, some clasts (*a, c, z, 05*), primarily comprising ~~ignimbrites and ignimbrite tuffs~~weakly-lithified tuffs, exhibit compositions that fall between phonolites and trachytes. Specifically, the Si values in the pumice clasts range from 55.1 to 60.8 wt% with a standard deviation (st.dev.) typically < 1 wt% in aphyric samples and ≤ 1.5 in porphyritic ones. The concentration of Na + K in the pumice fragments is comprised around 14 ± 0.5 wt%, except for clast *c* (12.3 wt%), thus confirming their uniform geochemical composition.

On the other hand, st.dev. values exceeding 1.5 in Si concentration and 1 in Na + K concentrations were registered for weakly-lithified tuffs~~tuffs and ignimbrite clasts~~. This could be symptomatic of a higher heterogeneity, possibly determined by the occurrence of microcrystalline minerals in the cineritic matrices of these pyroclastic rock fragments~~stone chips~~, whose presence can be inferred also by the inhomogeneous interference colours observed in the SEM backscattered images (see **S2 ~~Figure~~Fig, b**).

The geochemical profile of the clasts is compatible with most of the volcanic products of the Campanian magmatic province, including the alkaline and highly-alkaline series of the main Phlegraean eruptions (pyroclastic products) [91,94], comprising the pre- and Campanian Ignimbrite (pre-CI/CI), pre- and Neapolitan Yellow Tuff (pre-NYT/NYT), post-NYT as well as the Phlegraean-correlated volcanoes of Ischia and Procida-Vivara islands (**~~Figure~~Fig 8~~7~~, a**). However, some of the clasts, characterized by low-Si concentrations, fall outside the intervals of the Phlegraean Fields.

~~The Si-depletion could be attributable to a slight alteration of the clasts after pozzolanic/para-pozzolanic reactions that may have affected even the innermost cores of the less-reacted clasts.~~

~~On the basis of~~Moreover, by only considering the TAS distribution, all the observed profiles except clast 09 appear geochemically compatible with the Somma-Vesuvius explosive events older than 79 CE (**~~Figure~~Fig 8~~7~~, b**). On the other hand, they are incompatible with the highly-alkaline pyroclastic deposits

~~scoriaceous cinerites~~ of the Roman volcanoes, i.e., ~~Colli Albani~~the ~~(~~*harenae fossiciae* (Vitr. De Arch. 2.6.6), comprising Pozzolane Nere, Pozzolane Rosse and Pozzolanelle from the Colli Albani eruptive facies a~~)~~nd *carbunculus* from Colli Sabatini, according to [95]. They appear ~~and~~ incompatible also with most of the other products of the Roman and Tuscan magmatic provinces [91,96,97], as well as with the rhyolites and rhyolitic breccias of the Oligocene eruptions of the Euganean Hills in the Veneto region [98] (~~**Figure**~~**Fig 8~~7~~, c**). ~~Finally,~~ A~~a~~ very ~~feeble~~ slight overlap can be only tracked with certain pyroclastic phonolites of Vulcano in the Aeolian Arc Isles [99] (~~**Figure**~~**Fig 8, d**).

These results demonstrate that TAS is non-conclusive for provenance determination of the ~~volcanic pozzolans~~pyroclastic clasts under investigation. Therefore, trace elements, collected via LA-ICP-MS, were crucial to exactly pinpoint their origin. Glassy/cineritic areas of 9 pumices and 2 weakly-lithified tuffs ~~tuffs~~ from sub-samples TSF_T9B and TSF_T9C (**S2 Table**), presenting unreacted areas wide enough to be comprised within the 55-µm laser ablation's spot size, were selected and analysed avoiding as much as possible the altered portions (~~**Figure**~~**Fig 9~~8~~**).

Good results were collected for most of the analysed pumices (both aphyric and porphyritic). In these clasts, the quantification of REE and HFSE returned a low st.dev. (see the comments to the data reported in the **S2 Table**), that, in particular, is coherent for the concentrations of Zr, Y, Nb, Yb, La, Th and Ta, usually considered in literature for provenance discrimination [29,33,42,45,46,61,62,100]. Conversely, the two analysed weakly-lithified tuff ~~tuff samples~~clasts (~~clasts~~ *a* and *s*) exhibited significantly high st.dev. and unusual average values, confirming the geochemical inconsistency already evidenced after EDS investigations. Therefore, while the LA-ICP-MS technique proved effective for tracking the geochemical fingerprint of fresh and chemically homogeneous tephra, it is not appropriate for analysing volcanic rocks presenting heterogeneous petro-mineralogical composition. Thus, these clasts were removed from the dataset considered in the following analytical steps.

As suggested by the TAS, the discordancy with the volcanic products of Roman district (comprising Colli Albani and Colli Sabatini pyroclastic ~~produces~~products~~'s *harenae fossiciae*~~) and Aeolian Isles, is confirmed also by trace elements, as demonstrated by Th/Ta vs Nb/Zr and Zr/Y vs Nb/Y scatterplots [61,101]. In the first one, the analysed clasts plot within the field of the Neapolitan volcanoes (**S3**~~.~~ ~~**Figure**~~**Fig, a**), characterized by the low ratio of Th/Ta (usually < 15, except from Pomici di Mercato eruption of Mt

Somma-Vesuvius), whereas, in the Zr/Y vs Nb/Y scatterplot (**S3 ~~Figure~~Fig, b**), the clasts, connoted by the high Nb/Y ratios (always > 1.7), overlap the field of Campania and they are incompatible with both Tuscan/Roman Magmatic districts and Aeolian Isles.

To establish the provenance of the ~~volcanic pozzolans~~ pyroclastic clasts within the Neapolitan district at high resolution, a selection of trace elements connoting their fingerprint was compared to that of around 950 pyroclastic products selected from literature, comprising the main eruptive events of the Phlegraean Fields, Mt. Somma-Vesuvius, Ischia and Procida-Vivara.

In the Zr/Y vs Nb/Y diagram (**~~Figure~~Fig 10~~9~~, a**), most of the samples overlap the Phlegraean Fields' intervals, showing strong correlations with the products of the post-NYT activities. However, some clasts with Nb/Y values < 2.0 (in particular *e* and *j*) present certain affinities with geological samples of the Ischia's pre- and post-Monte Epomeo Green Tuffs (MEGT). Additionally, other pumices (especially *k*) fall close to the intervals associated with certain pre-79 CE Mt. Somma-Vesuvius juveniles.

The Nb/Zr vs La/Yb scatterplots, adopted in [33] for discrimination, do~~es~~ not provide any other relevant information (**~~Figure~~Fig 10~~9~~, b**).

Both the scatterplots indicate that the compatibility with the Phlegraean Fields of the analysed clasts is plausible, but it cannot be considered as ubiquitous. Consequently, to enhance the resolution of the analysis, non-fractional HSFE and REE from ~~the~~ 950 geological markers constituting our in-house geochemical dataset were compared in exploratory bivariate scatterplots trace-by-trace. Notably, Yb concentrations show a relevant and coherent variability within the volcanic units of the Gulf of Naples, resulting extremely informative when plotted versus Zr, Nb and Th. A similar pattern is observed when Nd is plotted versus Rb.

In ~~fact~~detail, in the Nd vs Rb scatterplot (**Fig 10~~9~~, c**), and in the Yb vs Zr, Yb vs Nb, Yb vs Th ones (**Fig 11~~0~~, a-c**) ~~Yb vs Zr, Yb vs Nb, Yb vs Th and Nd vs Rb scatterplots (**Figure 9, c-f**)~~, the 9 analysed pumices systematically overlap the intervals of distribution of the Phlegraean fields, connoted by positive correlation between Yb with Zr, Nb and Th, as well as between Nd with Rb. All the TSF_T9 clasts have Yb and Nd values higher than 4 and 60 ppm, respectively, making them incompatible with Mt. Somma-Vesuvius eruptions, characterized by Nd values < 50 ppm and Yb values < 2 ppm except for the older

Formatted: Font: Bold

series, where Yb values can reach 5 ppm. The distribution of the Ischia pre-MEGT, MEGT and post-MEGT products appears somewhat inconstant. Although Yb and Nd are positively correlated with the other trace elements, when compared to the other Phlegraean products, these trends show slightly higher ratios, particularly evident in the Yb vs Th scatterplot (see ~~Figure~~Fig 1109**,** ~~c~~e).

Having proved a ubiquitous compatibility with the Phlegraean Fields, the investigated clasts appear to align in particular with the post-NYT eruptive events, characterized by high Zr, Th and Yb. However, in most of the diagrams, the analysed pumices show a latent compatibility with pre-NYT, NYT and CI juveniles too.

To prove with the highest degree of resolution the origin of the clasts within the Phlegraean domain, discriminant analysis (DA) was carried out. The geological markers, subdivided according to the major Phlegraean eruptive events, were used as classification factors, and the most informative trace elements adopted for provenance discrimination in the scatterplots (Zr, Nb, Y, Th, Ta, Yb, La, Nd and Rb) were selected as independent variables. The compatibility with possible sources was established considering two probabilistic terms.

For all the analysed pumices, the DA reported a 1st probability correlation, ranging from 75 to 100%, with the post-NYT volcanic products (see **Tab. 2**). This compatibility was eventually confirmed through a spider-diagram reporting the trace element profiles of the analysed pumices, perfectly overlapping the intervals of the geological marker samples associated with the post-NYT eruptions (~~Figure~~Fig **12**10).

These outcomes confirm that the weakly-lithified deposits of the late Phlegraean eruptions, outcropping around the Gulf of Naples' coastline, are the likely source of the volcanic material included in TSF samples.

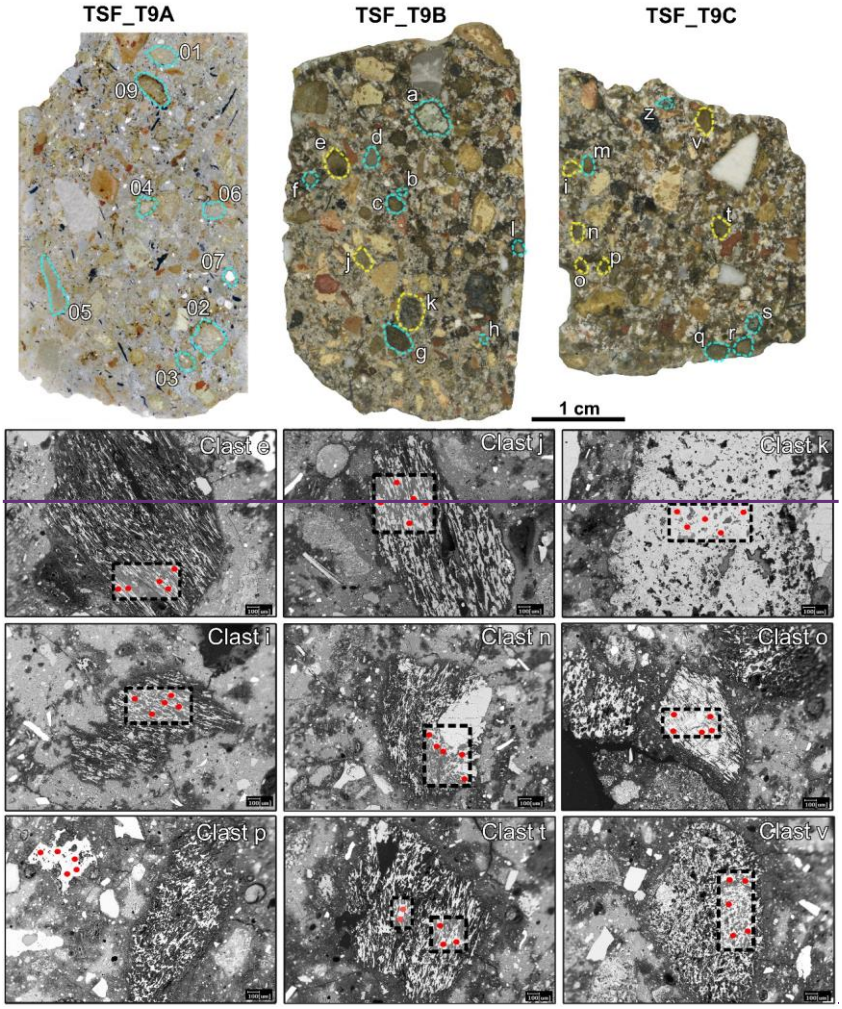

~~Figure~~Fig ~~8~~7. TAS scatterplots of ~~volcanic pozzolans~~pyroclastic clasts (pumices, obsidians, tuffs ~~and ignimbrites~~) included in TSF_T9 sample, overlapped to the geochemical intervals of the main Plio-Quaternary volcanic districts of the Italian peninsula (reference for geochemical fields taken from [42] and references therein), distributed as it follows: (a) the main eruptive events of the Phlegraean Fields, comprising pre- and Campanian Ignimbrite (pre-CI/CI), pre- and Neapolitan Yellow Tuff (pre-NYT/NYT), post-NYT, and Phlegraean-correlated volcanoes of Ischia and Procida-Vivara; (b) the three main eruptive *facies* of Somma-Vesuvius; (c) the products of the Roman, Tuscan and Euganean Magmatic provinces; (d) pyroclastic products of the Aeolian Arc Isles.

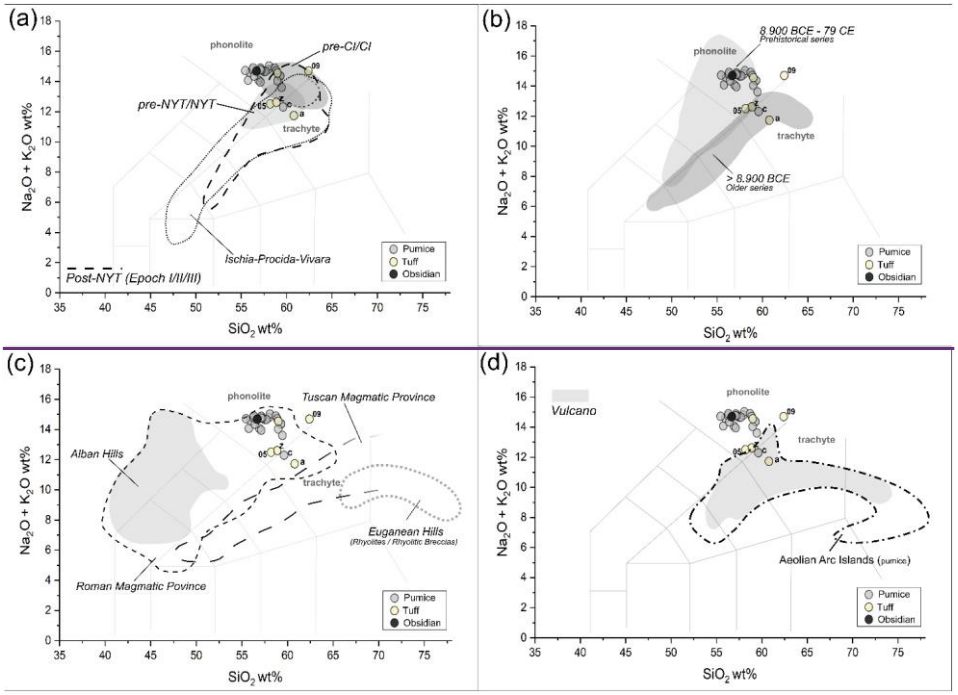

~~Figure~~Fig ~~9~~8. Identification of the ~~volcanic pozzolans~~pyroclastic clasts in the polished sections TSF_T9A, TSF_T9B and TSF_T9C samples analysed by SEM-EDS (in yellow and light-blue dashed lines) and the nine pumices analysed by both SEM-EDS and LA-ICP MS (in light-blue dashed line). ~~In t~~The lower part of the figure reports the BSE acquisitions of the nine ~~LA-ICP-MS analysed~~ pumices~~s~~ clasts analysed by LA-ICP-MS, with indication of the Laser Ablation spot of analysis (the dimension of the red dots is scaled down to the instrumental 55 μm spot size).

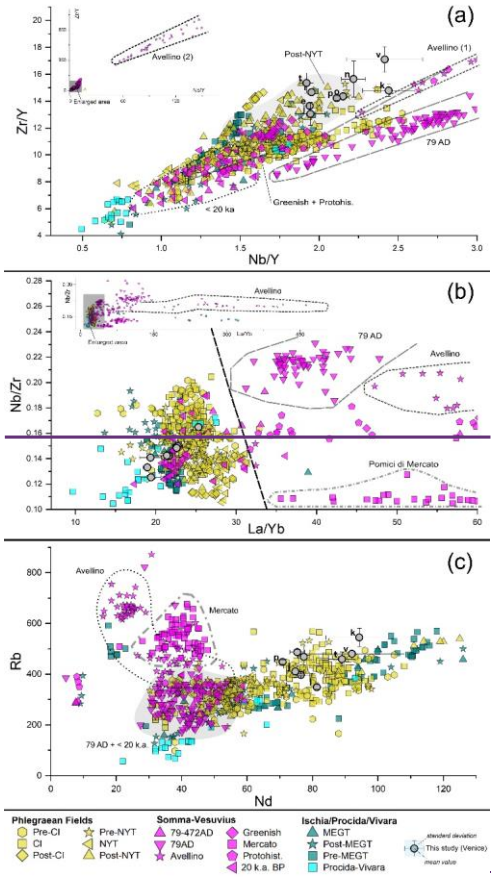

669

670 **~~Figure~~Fig 109**. Trace elements' scatterplot distribution of the pumices in TSF T9A and TSF T9B samples, in
671 relation with that of juveniles' geological markers from the main eruptive facies of the volcanic units of the Gulf of
672 Naples. A selection of discriminant trace elements for provenance recognition was adopted: (a) Nb/Y vs Zr/Y; (b)
673 La/Yb vs Nb/Zr; (c) Nd vs Rd. Geochemical data of reference geological markers are taken from literature, as it
674 follows: Phlegraean Fields (eruptive activities distinguished according to [102,103]; Pre-CI [102,104–107]; CI
675 [102,104,107–109]; post-CI [104]; Pre-NYT [102,106,107]; NYT [102,110,111]; Post-NYT [61,102,110,112–117];
676 comprising Epoch I, II and III according to [117]; Somma-Vesuvius (eruptive activities distinguished according to
677 [118]; Older series – Pomici di Base < 20 k.a. BP [105,119–124]; Greenish pumice [120,122,125]; Pomici di Mercato
678 [120,122,124,126,127]; Avellino [119,120,124,127,128]; Protohistorical series – AP1/AP6 [120,122–124]; 79 CE
679 [119,120,122,124,127,129–131]; post-79 CE – 472 CE [119,120,122–124]; Ischia, eruptive activities distinguished
680 according to [132]; pre-MEGT [132–136]; MEGT [132,133]; post-MEGT [132–136]; Procida-Vivara [137].

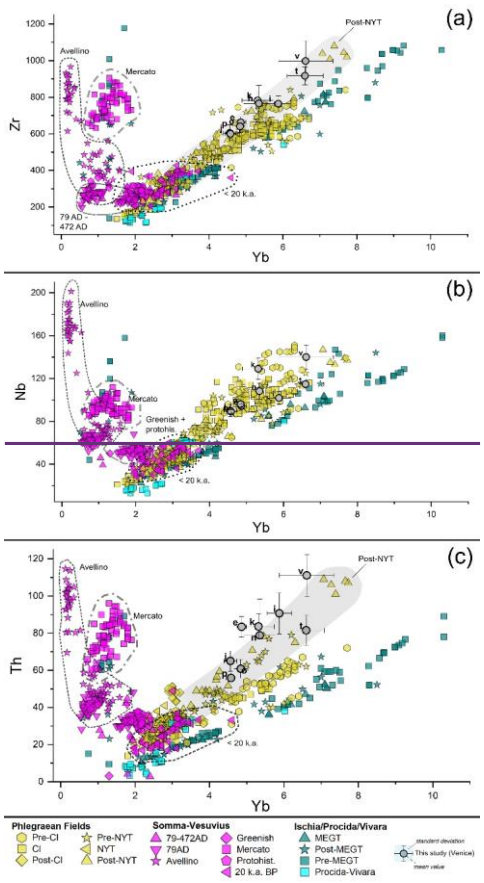

Fig 110. Trace elements' scatterplot distribution of the pumices in TSF_T9A and TSF_T9B samples, in relation with that of juveniles' geological markers from the main eruptive *facies* of the volcanic units of the Gulf of Naples. A selection of discriminant trace elements for provenance recognition was adopted: ~~(a) Nb/Y vs Zr/Y; (b) La/Yb vs Nb/Zr;~~ (ae) Yb vs Zr; (bd) Yb vs Nd; (ce) Yb vs Th~~; (f) Nd vs Rd~~. Geochemical data of reference geological markers are taken from literature, as it follows. Phlegraean Fields (eruptive activities distinguished according to [102,103]: Pre-CI [102,104–107]; CI [102,104,107–109]; post-CI [104]; Pre-NYT [102,106,107]; NYT [102,110,111]; Post-NYT [61,102,110,112–117]; comprising Epoch I, II and III according to [117]; Somma-Vesuvius (eruptive activities distinguished according to [118]: Older series – Pomici di Base < 20 k.a. BP [105,119–124]; Greenish pumice [120,122,125]; Pomici di Mercato [120,122,124,126,127]; Avellino [119,120,124,127,128]; Protohistorical series

Formatted: Font: Bold

– AP1/AP6 [120,122–124]; 79 CE [119,120,122,124,127,129–131]; post-79 CE – 472 CE [119,120,122–124]; Ischia, eruptive activities distinguished according to [132]: pre-MEGT [132–136]; MEGT [132,133]; post-MEGT [132–136]; Procida-Vivara [137].

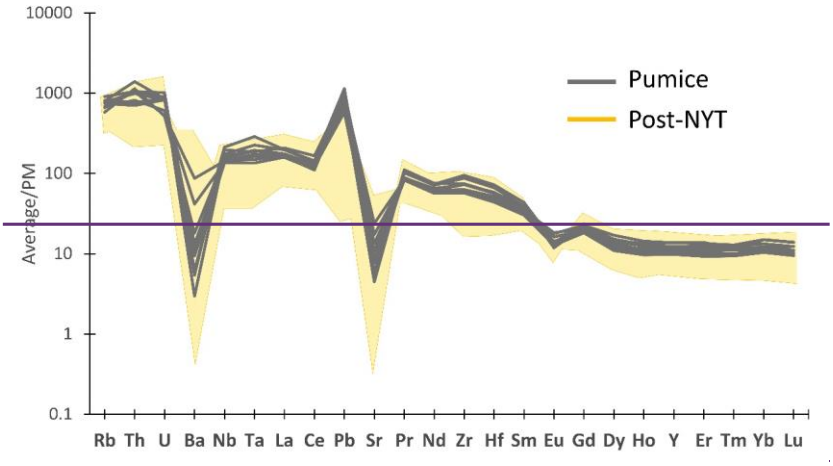

Figure 12. – Comparative spider-diagram of trace elements (average values for each single clast) between the pumice glass measured by LA-ICP-MS in this study and the field of Post-NYT juveniles from literature (references for post-NYT are reported in the main text and captions of Figure 10 and 11). Data were normalized to the Primitive Mantle recalculated from pyrolite (PM, data from: [138]).

Table 2. Results of the discriminant analysis of pumice samples with probabilistic association to sources within the main Phlegraean eruptions (Classification Function Coefficients and Discriminant Function Coefficients are reported at the **S3. Table**).

Clast	Provenance (1st prob.)	Highest Group	Squared distance	Prob. (%)	Provenance (2nd prob.)	2nd Highest group	Squared distance	Prob. (%)	TOT prob. (%)
j	Post-NYT	44.3087	4.61778	90.32	pre-NYT	42.0199	9.1953	9.16	99.48
p	Post-NYT	47.6212	5.88543	75.17	pre-NYT	46.4381	8.25164	23.03	98.2
o	Post-NYT	54.886	8.51028	80.03	pre-NYT	53.3371	11.6079	17.01	97.04
e	Post-NYT	35.2316	20.086	97.76	pre-NYT	31.4485	27.6522	2.22	99.98

k	Post-NYT	63.3567	12.6565	91.23	NYT	60.8802	17.6097	7.67	98.9
n	Post-NYT	39.832	17.359	96.96	pre-NYT	36.3686	24.286	3.04	100
i	Post-NYT	16.6407	57.7087	98.12	pre-NYT	12.6858	65.6184	1.88	100
t	Post-NYT	40.453	46.5635	98.34	pre-NYT	36.3725	54.7244	1.66	100
v	Post-NYT	29.2316	76.88	99.92	pre-NYT	22.1365	91.0701	0.08	100

~~Archaeological inferences~~Discussion

The data obtained from this research are extremely interesting from an archaeological point of view, as they offer important information regarding the building technique and the environment in which the TSF well-cistern was placed, providing a new piece of evidence on the commerce and exploitation of the Vitruvian *pulvis ~~puteolana~~* in the ancient Mediterranean.

~~5.1~~ The construction technique and environment of ~~'Torrione'~~ San Felice well-c~~C~~istern

Formatted: Font: 16 pt

In a context-related perspective, the building technique of the well-cistern appears extremely complex. Nowadays, due to eustatic and subsidence phenomena, the first course of bricks positioned at the bottom of the structure lies at a depth of – 4.20/3.75 m MSL, but even in Roman times the base of the well-cistern was placed at a depth of around – 2.0 m MSL [48]. This implies that at least the foundations of the cistern, but more probably most of the structure, had to be ~~realized~~ built underwater. Therefore, it seems difficult to think that the Romans were able to build a structure made of local *sesquipedali* bricks laid on mortar joints and mortar-coated without using cofferdams [33,139], possibly constituted of a double bulkhead (Vitr., *De Arch*. 5.12.5). In order to work in an adequately dry space, it is even probable that machines for the lifting of the liquid infiltrations, as those described by Vitruvius (Vitr., *De Arch.* 10.6.1-4) [140,141], were employed avoiding as much as possible the intrusion of saltwater during construction. This technique was historically used in Venice to build underwater, but some pieces of evidence o~~f~~n the use of wooden scaffoldings to allow the setting of mortar-based materials underwater, dating back to the Roman times, were documented in other sites along the Northern Adriatic coast [142].

However, this solution does not deny the opportunity to use, whereas available, hydraulic mortars~~,~~ in order to fasten the bricks, improving the grip and bonding of the building materials. The natural and artificial pozzolans (volcanic ~~ashes~~ tephra and crushed ceramics in particular) were processed and finely milled by the crafts to maximize their interfaces with the lime binder, thus increasing the overall ~~hydraulic~~ waterproofing capability of the resulting mortars. In addition, the reactivity was certainly fostered by the brackish liquid medium the mortar was presumably mixed with and that came into contact with the binding materials shortly after the cofferdams were dismantled. In fact, when Phlegraean pozzolan-rich mortars are applied in masonry joints or *opus caementicium* casts for on-land buildings, where carbonation of lime-based binder is favoured by air circulation, the development of reaction processes in the matrices can be minimized. There are even cases where it appears almost null or just limited to the interface edges with the reactive elements [42,143]. Therefore, it is eventually clear how the buried, alkaline-rich submerged anoxic environment favoured the latent reactivity of certain aggregates over aerial carbonation, influencing the development of pozzolanic or para-pozzolanic reaction processes. Thesee~~is~~ complex reaction kinetics significantly contributed to the strength and durability of the compounds along time [7,26,73,100], as already recognized for underwater concrete piers of Roman ~~port~~ harbour infrastructures [7,28,100,144]. ~~-~~However, in this case, a clear prevalence of Mg-based hydrated ~~systems~~phases over traditional Ca-based aluminosilicate hydrates is observed. This ~~opens up~~opens new perspectives regarding the complex relationships between Ca and Mg in authigenic reaction products~~, to be investigated in detail~~currently under investigation our research group.

Regardless of the specific kinetics of the reactions, t~~T~~his result confirms – once again – the brilliant intuition of the Roman engineers, epitomized in the well-known Vitruvian book, regarding the waterproofing abilities and mechanical strength conferred by the combination of Neapolitan *pulvis* with lime pastes under highly alkaline and anoxic conditions. Recent studies, using replicas of ancient specimens, have demonstrated that mortar-based materials reinforced with volcanic tephra from the Neapolitan volcanic regions exhibit compressive strengths of approximately 7-8 MPa [33,145,146]. While not reaching the standards of contemporary cements, these findings indicate~~signify~~ a significant improvement over the majority of ancient mortars and concretes. These crucial discoveries were initially

embraced by the masons of the San Felice well-cistern and subsequently reinterpreted and adapted to the construction techniques and materials of the local tradition.

~~regarding the prodigious strength granted by *pulvis* puteolana once employed in the making of underwater concretes. These important findings were firstly assimilated by the indigenous workers and then reinterpreted and adapted to the construction methods and materials of the local tradition.~~

~~5.2~~ Commercialization and ~~use~~ function of *pulvis puteolanus~~na~~*

In general terms, the results obtained in this research offer a new piece on the diffusion of the Neapolitan pozzolans in the ancient Mediterranean. The ship-trading of the Neapolitan "~~prodigious powder~~pumiceous tephra ~~,"~~ from Campania up to the northernmost Adriatic route seems to be substantiated ~~in an ever~~ by continuously expanding ~~landscape of~~ attestations, that now range from the High Imperial Age [42] to the Late Antiquity [46]. New scenarios emerge regarding the widespread distribution of Neapolitan pozzolans in this area, which were even utilized in a context previously seen as peripheral in the Roman world, such as the Venice~~tian~~ Lagoon~~opened on the wide distribution of the Neapolitan pozzolans in this region, that were even employed in a context hitherto considered as marginal in the Roman world, such as the Lagoon of Venice~~. Indeed, from Aquileia, located around 11 km inland from the Adriatic coastline and around 80 km north of the Lagoon of Venice, another crucial proof of expoitation of Neapolitan pozzolans was evidenced by recent research [49]. This endorses the hypothesis that the material was transported as ship-ballast along the endo-lagunar route connecting Ravenna with *Altinum* and Aquileia [147]. In light of this data, the Lagoon of Venice ~~emerges~~ looks ~~as~~ intricately innervated within the commercial networks of one of the most renowned and specialized building materials of the authentic Roman tradition~~that, in the light of these data, now appears as deeply rooted in the commercial networks of one of the most esteemed and specialized building materials of the genuine Roman tradition. Indeed, from Aquileia, located around 11 km inland from the Adriatic coastline and around 80 km north of the Lagoon of Venice, another crucial proof of expoitation of Neapolitan pozzolans was evidenced by recent research [49]. This endorses the hypothesis that the~~

Formatted: Font: Not Italic

Formatted: Font: 16 pt

Formatted: Font: Italic

~~transport of the material was operated probably as ship ballast along the endolagunar route connecting Ravenna with *Altinum* and Aquileia [131].~~

Considering the dimension of the well-cistern (6.7 × 7.7 m), which may have been intended to serve fresh water to ships passing throughout the lagoon route, the quantity of mortar needed for the making of the structure was surely significant and, as a consequence, the amount of volcanic pozzolan exploited. Therefore, its use in the making of the mortars was not left to chance: as demonstrated by the different concentrations of volcanic aggregates in the analyzed samples, the masons carefully dosed the imported volcanic powder, by employing the largest quantity in the nodal joints of the well-cistern, to ensure an optimal sealing.

-The targeted utilization of *pulvis puteolanus* in the framework of waterproofing coatings ~~warrants attention~~is particularly interesting. In contrast to the structural applications observed in both on-land buildings and underwater piers in ~~port environments~~harbour structures, this ~~way of use~~employment of the volcanic powder seems to primarily entail the material for waterproofing and coating purposes. In particular, the significant presence of crushed ceramics in the examined mortars is a fundamental aspect to consider. In fact, the archaeological evidence demonstrate that volcanic pozzolans-enriched mortars, presenting no ceramic inclusions, were mainly employed in masonry joints and structural mortars and concretes [12,40,66,148,149], possibly for improving the strength and the cohesive properties of the compounds, as recalled by Vitruvius regarding both the *pulvis* (Vitr. *De Arch*. 2.6.1) and the *harenae fossiciae* (Vitr. *De Arch*. 2.4.1-2). On the other hand, the concurrent utilization of both natural and artificial pozzolans (pyroclastic rocks and crushed ceramics, respectively) represents a common construction tradition among Roman artisans, frequently adopted for lime-based coatings of hydraulic infrastructures ~~As demonstrated by the different concentrations of volcanic aggregates in the analyzed samples, the local builders carefully dosed the imported material, employing the largest amounts of pozzolans in the nodal joints of the well-cistern, to ensure an optimal waterproofing. Crushed ceramics were also added for this purpose, according to the common *modus operandi* of Roman crafts of combining natural and artificial pozzolanas (pyroclastic rocks and crushed ceramics, respectively) for waterproofing the coatings of hydraulic infrastructures~~ [26,41,149–151].

Conclusions

This study provides many insights in the current field of research on ancient hydraulic mortars. The main results, summarized below, can be split into three categories, comprising: (a) highlights on the kinetics of pozzolanic and para-pozzolanic reaction processes; (b) novel tools for the provenance determination of volcanic pozzolans, and (c) crucial advances on the technology of ancient pozzolanic mortars and on the trading of *pulvis puteolan*~~*a*~~*us* in Northern Italy in Roman times.

- **Kinetics of pozzolanic and para-pozzolanic reaction processes**. The study has provided new insights regarding the development of the ~~peculiar~~ reaction processes in ancient mortar-based materials in submerged anoxic environments. Being clear the role of saltwater in fostering the latent "pozzolanicity" of certain materials, the prevalent formation of Mg-based hydrate products (M-S-H and M-A-S-H) over traditional Ca-based pozzolanic ones (C-S-H and C-A-S-H) was promoted by the long-term evolution of the reactions, as the binders appear clearly Ca-depleted and feebly carbonated. This opens new perspectives on the complex kinetics of reactions occurring in ancient underwater mortars and on the crucial role played by Mg ions in these processes.
- **Tools for provenance determination of volcanic pozzolans**. A nodal point of the study was the provenance determination of the ~~natural pozzolans~~pyroclastic clasts identified in the samples. From a methodological point of view, the work offers new tools for measuring the geochemical fingerprint of volcanic aggregates in ancient mortar-based materials and for tracking their origin. In particular, an enriched set of discriminant diagrams was proposed, outlining the crucial role of Yb and Nd for a geochemical differentiation of juveniles within the Neapolitan magmatic district. The statistical processing of the descriptive geochemical elements with multivariate techniques, such as the discriminant analysis (DA), can constitute another useful tool to validate and crosscheck the information derived from scatterplot observations.

- **Technology of ancient pozzolanic mortars and trading of *pulvis puteolanusa***. From a historical-archaeological point of view, this study documented a new ~~peculiar~~ case of utilization of Phlegraean volcanic pozzolans in Roman times and the first attestation of this material in the lagoon of Venice. This evidence allows to reconsider this territory as strictly connected in the ancient Northen Adriatic economic-commercial routes and deeply embedded in the Roman technological awareness of genuine central-Italic tradition, ~~thought redesigned~~reinterpreted according to the forms, methods and construction materials of the local built environment.

Supporting information

S1 Analytical methods. Instrumental equipment and standards.

S1 ~~Figure~~Fig Roman well-cisterns. Examples of Roman well-cistern documented in the site of Ca' Ballarin in the Northern Lagoon of Venice (up) and from Aquileia (down). Images taken from [48] (see references in the main text).

S2 ~~Figure~~Fig SEM-EDS investigation of ~~volcanic~~ pyroclastic clasts~~pozzolans~~' mineral chemistry and iron slags.

S3 ~~Figure~~Fig TAS diagram. Trace elements' scatterplots of the pumice clasts in TSF_T9B and TSF_T9C subsamples analyzed by LA-ICP-MS; a) Nb/Zr vs Th/Ta scatterplot of pumice clasts in relation to the fields occupied by the Roman, Tuscan and Campanian magmatic provinces and Aeolian Arc Islands' volcanic products (compositional fields from [42] and references therein); b) Nb/Y vs Zr/Y scatterplot of clasts' samples in relation to the fields occupied by the Roman, Tuscan and Campanian magmatic provinces, and the Aeolian Arc Island's products (compositional fields from [42] and references therein).

S1 Table. **Major elements composition of the pyroclastic clasts~~volcanic pozzolans~~**. Clasts were analyzed by SEM-EDS and described as %wt in oxides (average values and standard deviation); b.d. = Components below the detection limit.

S2 Table. Trace elements of the pyroclastic clasts~~volcanic pozzolans~~. Chemical results of the selected clasts of pumice analyzed by LA-ICP-MS (average values of multiple spot-size analyses are reported and related standard deviation). Values are described as ppm

S3 Table. Coefficients of the discriminant analysis.

Acknowledgments

We are grateful to Dr. Alessandro Asta (Soprintendenza Archeologia, Belle Arti e Paesaggio per l'area metropolitana di Venezia e le province di Belluno, Padova e Treviso) for the support in the underwater archaeological research and endorsement to excavation activities.
