# Supplementary material for: Vitruvian binders in Venice: First evidence of Phlegraean pozzolans in an underwater Roman construction in the Venice Lagoon
Source: PLoS One. 2024 Nov 22;19(11):e0313917. doi: 10.1371/journal.pone.0313917 (PMC11584134; doi:10.1371/journal.pone.0313917)
Supplement: Attachment — Submitted filename: Response to Reviewers_rev2.pdf [file pone.0313917.s013.pdf]

dBC

UNIVERSITÀ DEGLI STUDI DI PADOVA

DIPARTIMENTO DEI BENI CULTURALI:
ARCHEOLOGIA, STORIA DELL'ARTE,
DEL CINEMA E DELLA MUSICA

Piazza Capitaniato 7
35139 Padova

Padova, October 2024

Abstract

- 23 “to enhance their hydraulic capabilities” What specific results from the data validate this hypothesis? In the abstract, this should be stated as, for example, ‘as shown by”

AND

The interpretations stated in the Abstract have not been addressed with explicit data. For example, “to enhance their hydraulic capabilities”

The sentence was rephrased, omitting the final part mentioning the improved hydraulic capabilities.

- 25 “(LA-ICP-MS) areas of apparently fresh volcanic glass, outlining the actual geochemistry of magma’s pristine composition within the analysed clasts.” The emphasis is on the “magma’s pristine composition”. Magma, defined molten or semi-molten material beneath the surface of Earth, also contains crystals so that “magma’ is not directly correlative with “apparently fresh volcanic glass”. This is not geologically correct.

Rephrased into: outlining the original geochemistry of analysed clasts

- 29 “Provenance determination was complicated by the significant reaction processes the analyzed pyroclastic clasts were object of, seriously affecting their geogenic chemical and petro- mineralogical features” There is a conflict here with the “apparently fresh glass” stated above. The reader is now seriously confused. This confusion must be resolved.

The paragraph was revised to emphasize that, although most volcanic pyroclastic clasts appeared to be significantly altered at the interface with the binder, some larger clasts (typically exceeding 450 microns to plurimillimetric in size) still retained areas of volcanic glass that remained unaltered. These preserved zones were thoroughly analyzed and mapped using LA-ICP-MS and SEM-EDS techniques.

- 38 “to enhance the physical and mechanical properties of seawater concretes” Again, as in comment 23, the reader does not have any indication of the data validating or justifying of statement.

AND

“enhance the physical and mechanical properties of seawater concretes”

The sentence was rephrases highlighting the indication of Vitruvius who suggested the use of Phlegraean pozzolans to improve the physical and mechanical properties of seawater concretes, noting that when mixed with lime and cement, Neapolitan pozzolans not only provide greater strength to structures but also harden underwater during the construction of concrete structures and masonry “quod commixtum cum calce et caemento non modo ceteris aedificiis praestat firmitatem, sed etiam moles cum struuntur in mari, sub aqua solidescunt…”

Main Text

- 53 “are mixed with calcium hydroxide (portlandite) in pastes”. Not all cementitious materials are prepared with paste mixtures.

In pastes removed from the text.

- 253 diopside, sample TSF_T9 (Fig. 4 XRPD): “it demonstrates the firing of Ca-rich clays at high temperature (>850 °C) [69].” This is very confusing. In Fig. 3, the petrographic image of TSF-T9 shows no labels for crystals of clinopyroxene or diopside. Ceramic fragments (CF) are labelled and vesicular pumice fragments are labelled, as well, as P. (Note that the legend erroneously gives Vp as the label.) Yet there is no specific validation for diopside in the ceramic fragments. Diopside is a phenocryst phase in some Gulf of Naples pyroclastic deposits. The solidus temperature of diopside at 1 atmosphere pressure is 1,278°C, this is the horizontal line at the eutectic temperature in the diopside-anorthite system. https://agupubs.onlinelibrary.wiley.com/doi/10.1029/JB094iB07p09508 Problematically, the reference cited [69] is for modern firing temperatures. Ancient ceramic firing temperatures are 700-1100, substantially lower that the diopside solidus: <1080 degrees C https://www.nature.com/articles/222081a0.pdf <1150 degrees C

  https://www.researchgate.net/publication/229590301 Determination of the Firing Temperature of Ancient Ceramics by Measurement of Thermal Expansion

  Magnetic susceptibility testing allowed for determination of the maximum firing temperatures, showing that the pottery were fired predominantly at 780°C, with some samples being fired at temperatures up to 860°C. https://onlinelibrary.wiley.com/doi/abs/10.1002/gea.21770

  Thus, absence of petrographic validation of diopside in Fig. 3, the problematic citation for “firing temperatures”, and the recognition of diopside as a volcanic phase, damages the overall credibility of the analytical investigations and inferences.

  AND

  The sole label of Cpx in the petrographic images seems to be in a volcanic fragment (Fig. 5m). This, again, calls line 253 into question.

We thank the reviewer for the useful comments regarding the occurrence of diopside in the analyzed materials, but we are confident about the reported conclusions. We are aware that the theoretical solidus temperature of diopside is well above that stated in our manuscripts, nevertheless this evidence is not valid in ceramic systems far from equilibrium conditions, with relevant fluxing agents (especially calcium and magnesium carbonates) such as those commonly used for the production of ancient ceramics. This is demonstrated by the experimental study reported in the citation [69], that is not related to modern ceramic production processes, but it is “…reproducing two ancient firing techniques: pit firing, characterised by a high heating rate and short residence time in a reducing atmosphere; kiln firing, with a low heating rate and long residence time in an oxidising atmosphere”. We are reporting a couple of further sentences stated in the paper corroborating our hypotheses: 1) “Further increases in temperature caused the decarbonation of calcite and the crystallization of gehlenite, diopside and spinel between 850 and 900 °C”; 2) Calcite and illite devolatilisation was observed between 800 and 850 °C, and was associated with the formation of gehlenite and diopside. Apart from the cited paper, the crystallization of diopside during high temperature (>850°C) firing processes in oxidized conditions within ancient ceramics is widely attested in the specific scientific literature (see as an example the following paper: Maria Daghmehchi, Adriano Guido, Adelaide Mastandrea, Mir Amir Salahi, Mehdi Omrani, Jebrael Nokandeh, Thermal analysis of ancient ceramics using the microchemical and microstructural alterations of foraminifera, Materials Characterization, Volume 130, 2017, Pages 81-91, “Decomposition of calcite leads to the crystallization of gehlenite (Ca2Al2SiO7) and diopside (Ca(Mg,Fe) Si2O6) by reacting with silicates in the temperature range of 800–950 °C). This reference was added to the edited text. Finally, we think that the absence of euhedral diopside crystals within ceramic fragments, associated with the scarce occurrence of clinopyroxene phenocrysts in the pyroclastic fraction of the samples and the abundant mass fraction of the phase determined through XRPD-QPA in the sample richest in ceramic fragments (TSF_T9), is a further confirmation of its correlation with the firing process of ceramics. Indeed, it has been commonly observed the absence of well-formed firing-related diopside crystals within ancient ceramics, due to their micro-cryptocrystalline and state and consequent incorporation in the sintered matrix of ceramics related to scarce crystal growth during anthropogenic firing processes (see as a reference the following paper and related SEM micrographs: Maria Daghmehchi, Chiara Coletti, Dong Hyeok Moon, Mohammad Esmaeil Esmaeili Jelodar, Hadi Omrani, Arianit A. Reka, Ali Nematollahzadeh, Mohammadamin Emami, Mineralogical and microstructural characterization of ceramics from the fifth and fourth millennium BC in the central plateau of Iran, Open Ceramics, Volume 15, 2023, 100427). A clarifying sentence regarding clinopyroxene phenocryst inclusion in volcanic juveniles was even added to further corroborate assumptions “Scattered clinopyroxene phenocrysts were embedded in the volcanic juveniles too (e.g. ***Fig 6, m****). However, their concentration is so diluted that they would not be detectable in XRPD analyses, making it improbable for them alone to account for the significant fraction of clinopyroxene identified by XRPD”.*

- Figure 5: TSF-9. the petrographic images are very small and the yellow labels very difficult to decipher against the background patterns. White or black bold text would be more readable.

The labels were modified to include a white fill color and bold text. Additionally, Figure 3 was updated to ensure consistency across the OM figures.

- Perhaps a single Cpx label is legible in Fig.5m? If so, this does not resolve the problematic statement of line 253, since the label seems to be located in a volcanic clast.

Yes it is a Cpx (probably augite). Statement at line 253 rearranged to avoid misinterpretation, as discussed above.

- Figure 6: TSF-9. Seven EDS point analyses of diverse components are shown. The reader needs further assistance in deciphering the mix of components shown in the images.

AND

The labels of the point analyses on the BSE images are very difficult to decipher against the patterned backgrounds. The labels of elements on the EDS analyses are so small that they are impossible to read. Each EDS point analysis should have a label stating the phase analyzed. Only one additional point analysis shows gives a composition for the "binder matrix".

AND

The lettering of the figure 7 scale bars is so small that the values are impossible to decipher.

AND

- b) detail of the area highlighted by the white dashed line in figure (b1) [I HAVE FINALLY FOUND THE "WHITE DASHED LINE" LOCATED IN FIG. 7A. A LABEL IS NEEDED FOR THIS AND THE COMPONENTS OF THE CLAST]; c) SEM acquisition of a portion of binding matrix [IS THIS AREA SHOWN IN FIGURE 7A?]; (d) detail of the area highlighted by the white dashed line in figure (c); (di) EDS analysis of the portion of binding matrix highlighted in figure (d);

The sizes of the element labels in EDS were enlarged in both figures 6 and 7, and labels indicating the analyzed points were included. Additionally, the bar values were increased.

- There seems to be no data table in the main text or supplemental section showing the results of the (total) 11 EDS analyses (Figs. 6, 7).

AND

813: Kinetics of pozzolanic and para-pozzolanic reaction processes In Figures 6, 7 there are 3? EDS point analyses of the "binder matrix". There is no apparent table showing the data. It is a long stretch to extrapolate this minimal fabric analysis of one sample (TSH-9) to the "prevalent formation of Mg-based hydrate products M-S-H and M-A-S-H) over traditional Ca-based pozzolanic ones (C-S-H and C-A-S-H) was promoted by the long-term evolution of the reactions".

AND

(d2) EDS analysis of the rounded cluster highlighted in figure (d) . [THESE ARE THE TWO POINT ANALYSES, FROM A SINGLE SAMPLE, THAT ARE USED TO INFER BROAD PROCESSES IN THE "BINDER MATRIX".]

The edited draft includes a table (New supplementary table 1) reporting the cumulative results of various EDS analyses on binder matrices, detailing the quantification of the analyzed elements along with the overall standard deviation. We appreciate the reviewer for this valuable suggestion.

- 262 "pyrite, detected by XRPD in most of the samples, is to be ascribed to post-depositional alteration phenomena of the materials". There should be a statement here, justified by reference citations, that the sedimentary source deposits for the "aggregate" do not contain detrital pyrite crystals and that the compositions of the lagoonal water(s) are enriched in sulfur. Then, the hypothesis could be made that the crystals possibly derive from sulfate-reducing bacteria.

The sediments do not contain pyrite crystals. Local sands are primarily composed of carbonates (calcite and dolomite) and silicates (quartz) (De Lorenzi Pezzolo A, Colombi M, Mazzocchin GA. Where did Roman masons get their material from? A preliminary DRIFTS/PCA investigation on mortar aggregates from X Regio buildings in the Veneto area (NE Italy) and their potential sources. Environ Sci Pollut Res. 2018;25: 28798–28807. doi:10.1007/s11356-018-2202-0). Thus, the presence of pyrite is attributed to the secondary activity of sulfate-reducing bacteria. Sentence rephrased accordingly.

- 269 Here, the reader learns that TSF-T9 is "the mortar with the highest concentration of pozzolanic aggregates". "The aggregate is mainly constituted of poorly sorted ceramic elements (Fig 5, d),"

The sentence was rephrased indicating that the mortar "is also constituted by a relevant component of poorly sorted ceramic elements"

- 358 "surviving portions of fresh volcanic glass enriched in interfacial dissolution pits (Fig. 7, b, b1)," This statement calls into question the nature of the "unreacted volcanic glass". The caption is difficult to decipher. Microscopy and a legible scale bar should demonstrate that the 50 micron spot size for the "glass" phase of the LA-ICP-MS analyses is satisfied.

  AND

  (b2) EDS analysis of the reaction products filling an outgassing vescicle [misspelling] (corrected) of the pumice clast [WHAT IS THIS PHASE OR PHASE ASSEMBLAGE? THIS, TOO, DEMONSTRATES THE COMPLEX NATURE OF GLASS ALTERATION IN THE VOLCANIC AGGREGATE];

The authors propose that intact volcanic glass is preserved only in the cores of coarser pumice clasts, which are defined as larger than 450 microns. In contrast, the interface edges frequently display pronounced reaction profiles with the binding matrix, and the vesicles within the pyroclasts are often filled with hydrated reaction products, predominantly of the "MASH" type. A spot size of 55 microns is shown in Figure 8, with the caption confirming that the diameter of the spot is indeed 55 microns.

- Figure 7. Backscattered electron images and EDS microanalyses of the sample TSF_T9. (a) SEM acquisition of a highly reacted pumice clast [WHY IS THIS PUMICE CLAST SELECTED TO SHOW "UNRREACTED VOLCANIC GLASS"?]

Rephrased to avoid misinterpretation

- EDS analysis of unreacted volcanic glass within the pumice clast [THERE IS ETCHING AND DISSOLUTION OF THE PERIMETERS OF THE BRIGHT AREAS - and perhaps in three dimensions, as well. WHY IS THIS DESCRIBED AS "UNREACTED"?];

The core remains unreacted, while the perimeter displays clearly visible reaction edges. The sentence has been rephrased in a clearer way

Conclusions

- 822: "the work offers new tools for measuring the geochemical fingerprint of volcanic aggregates in ancient mortar-based materials" These methods may prove to be useful. However, the conflicted information given for the "highly reacted pumice clast" and "unreacted volcanic glass within the pumice clast" for Figure 7 and elsewhere in the text and figures creates real confusion as to what phase is analyzed and its fidelity in terms of recording primary glass compositions (in pyroclastic deposits that experienced authigenic alteration before excavation by Roman workers).

This confusion was clarified by rephrasing the sentences. Specifically, we aimed to convey that volcanic pozzolans often exhibit considerable reactivity with the binder, leading to notable dissolution at the interfacial boundaries, as demonstrated by SEM-EDS analysis in the interfacial zones of the coarser clasts. However, the coarser clasts, typically larger than 450 microns, retain unreacted volcanic glass cores. These cores remain intact and are sufficiently large (at least 55 microns) to be analyzed using SEM-EDS and LA-ICP-MS techniques.

- A careful, line-by-line revision of the text is needed to alleviate confusion throughout the text and figures and to bring clarity in ambiguous material descriptions and interpretations. Further revision by a native English speaker to streamline the text and use terms would also improve clarity.

Done

- References do not always follow a standardized format, for example, #4, #142

All bibliographic references have been thoroughly reviewed and standardized according to the journal's editorial guidelines.

Simone Dilaria, Ph.D. (corresponding author)

Assistant Professor (RTD-A) - L/ANT 07 (Classical Archaeology)

University of Padova, Department of Cultural Heritage

Piazza Capitaniato, 7 - 35139 Padova (Italy)

simone.dilaria@unipd.it
