# Supplementary material for: Vitruvian binders in Venice: First evidence of Phlegraean pozzolans in an underwater Roman construction in the Venice Lagoon
Source: PLoS One. 2024 Nov 22;19(11):e0313917. doi: 10.1371/journal.pone.0313917 (PMC11584134; doi:10.1371/journal.pone.0313917)
Supplement: Attachment — Submitted filename: Response to Reviewers.docx [file pone.0313917.s011.docx]

| **­** |  |  |
| --- | --- | --- |
|  |  |  |

**DIPARTIMENTO DEI BENI CULTURALI:**

ARCHEOLOGIA, STORIA DELL'ARTE,

DEL CINEMA E DELLA MUSICA

| Piazza Capitaniato 7  35139 Padova |  |
| --- | --- |
| Padova, March 2024 | |

To the Editors of PLoS ONE Journal,

please find attached our revised draft of the manuscript titled “Vitruvian binders in Venice: First Evidence of Phlegraean Pozzolans in an underwater Roman Construction in the Venice Lagoon”, by myself and co-authors, for publication in PLoS ONE journal.

The revised submitted draft has considered all the revisions suggested by the reviewer 2. Part of the answers were included as reply to the comments in the attached “PONE-D-23-36360_PDF Review_answers.pdf” file.

We include here the comments to the Journal Requirements reported in the response email for the original PONE-D-23-36360 submission of the 02/02/2024.

**Journal Requirements**

*Figures*

- *Figure 1 was edited and rearranged, with subfigures edited from open access journals (credits reported according to the PLOS form). The new satellite sketch map on the right of figure 1 is taken from Landsat portal, as reported in the caption.*
- *Regarding figure 2 the copyholder of the main image (orthophoto) is included as an author of the paper (Carlo Beltrame). Do we have to obtain a permission from one of the authors itself to reproduce in Open Access one of his own photos he deliberately wants to publish in open access with this submission?*

*Role of Funder statement*

As requested, we amend for the role of funder statement and we ask to edit it with the following paragraph.

- *For the study design of the research, SD was financed by a grant from the “Exceptional Laboratory Practices in Cultural Heritage: Upgrading Infrastructure and Extending Research Perspectives of the Laboratory of Archaeometry”, co-financed by Greece and the European Union project under the auspices of the program “Competitiveness, Entrepreneurship and Innovation” NSRF 2014–2020, SD (*[*https://ec.europa.eu/regional_policy/in-your-country/programmes/2014-2020/el/2014gr16m2op001_en*](https://ec.europa.eu/regional_policy/in-your-country/programmes/2014-2020/el/2014gr16m2op001_en)*). For the data collection and investigation of the well-cistern of Canale San Felice, CB was financed in 2023 by the PNRR project CHANGES – Cultural Heritage Active Innovation for Sustainable Society (project code: PE00000020;* [*https://sites.google.com/uniroma1.it/changes/*](https://sites.google.com/uniroma1.it/changes/)*); For the analytical investigations on samples, SD was financed from the project “Trade and use of volcanic pozzolans in the Roman world. A natural material for the production of eco-sustainable concrete of antiquity” (Principal investigator: Simone Dilaria, BIRD 2023 of the Department of Cultural Heritage of the University of Padova, project code BIRD230232/23), TG was partially financed by Habits PRIN 2022 project, code 2022BC2Z5F. The research was implemented and funded within the scopes of the University of Padova under the World Class Research Infrastructures (WCRI) programme – SYCURI (Synergic Strategies for Culture Heritage at Risk).*

**Reviewer 1**

- On lines 69 and 594 the pozzolanic material is wrongly termed "pulvis puteolana" rather than the correct Latin "pulvis puteolanus" seen elsewhere in the text.

*Edited and corrected*

- It might be useful to some readers if the previously documented pattern of trade in this material was outlined: most of it is found on the coast of Lazio and Toscana and in a few large harbours elsewhere, such as Caesarea Palaestinae and Alexandria

*The introduction was rearranged including a specification of the port evidences in Italy and Eastern Mediterranean*

**Reviewer 2**

**Introduction**

- The Introduction, unfortunately, contains many inaccuracies. Vitruvius did not use the term “pulvis puteolana” in *de Architectura* and never referred to Puteoli. There is also confusion over the use of chemical terms, such as “compound”, “liquid solutions (and “aliquot” in the Results); aerial lime-based mortars; and archaeological context. (Vitruvius wrote *de Architectura* in 30 BCE, for Octavian (not Augustus in 1st C CE)).

AND

- “Vitruvius” and “Vitruvian” appear many times in the manuscript yet the text of the actual passages from de Architectura are not given – nor is the relevance of these passages to the Venice lagoon mortars directly justified by the material analyses. Instead, there are oblique references that leave the reader confused as to the nature of a “prodigious powder”:

Line 35: “Vitruvian mention to the prodigious eﬀects of pulvis puteolana used in seawater concrete (Vitr., 5.12.2-3)”

AND

- Line 68: Both Vitruvius (Vitr. 2.6.1-2; 5.12.2) and Pliny (Pliny 16.202; 35.166) exalted the outstanding properties of the pulvis puteolana, referred to a prodigious powder outcropping around the modern town of Pozzuoli (Bay of Naples

Line 505: “exploitation of the “Vitruvian pulvis puteolana” in the ancient Mediterranean.

Line 537: “Vitruvian book, regarding the prodigious strength granted by pulvis puteolana once employed in the making of underwater concretes”

AND

- In fact, Vitruvius never used the term “pulvis puteolana”. He simply states “pulvis” (or dust). The Latin text of 2.6.1-2 and 5.12.2-3 appears at the end of this review. Attribution of an origin from Puteoli should correctly go to Seneca, Q Nat, or Pliny, HN: p. 2, Building for Eternity, 2014, Oxbow Books.

In Latin, Vitruvius’ term for the pumiceous, poorly consolidated volcanic ash that crops out “in the vicinity of Baiae and the territory of the municipalities around Mount Vesuvius” in the northwest sector of the Gulf of Naples was pulvis, “powder” or “dust” (De arch 2.6.1). This term refers to its finer grain size distribution, as compared with the granular scoriaceous ash or excavated sands (harenae fossiciae) of the region around Rome. Vitruvius thus indicates that the powdery ash used in first century BC came from either the Flegrean Fields near Puteoli or the Somma- Vesuvius volcanic districts (Fig. 7.2). The term, Puteolanus pulvis, or “dust (or powder) from Puteoli,” occurs in two of the three passages by ancient authors that mention pulvis (Seneca, Q Nat 3.20.3; p. 26, Passage 14; Pliny, HN 16.202; pp. 26–27, Passage 15). In Pliny HN 35.167 (p. 27, Passage 16) the phrase is a pulvere Puteolano. Vitruvius does not attach a locative adjective, but simply states pulvis.

AND

- These issues create a highly problematic foundation for the manuscript. Substantial revision is needed to resolve the textural errors, add the relevant descriptions from de Architectura, and state explicitly how these are validated by the analytical results.

AND

- “Both Vitruvius (Vitr. 2.6.1-2; 5.12.2) and Pliny (Pliny 16.202; 35.166) exalted the outstanding properties of the *pulvis puteolana*, referred to a prodigious powder outcropping around the modern town of Pozzuoli (Bay of Naples).” Puteoli and “pulvis puteolana” is never referenced in *de Architectura*. Also,

1) "exalted": definition involves a person and is not justified by the text passages: (of a person or their rank or status) placed at a high or powerful level; held in high regard; in a state of extreme happiness. Please revise.
2) "prodigious", "powder", "outcropping" : the definitions of these words are at odds with the actual behavior of the pyroclastic deposits. Without the text passages the reader will not have a context for this oblique description
3) “Preservation”: the activity or process of keeping something valued alive, intact, or free from damage or decay.

AND

- "prodigious" has no direct translation in the Latin of the ancient texts.

Although the Gulf of Naples tephra (and tuﬀ) deposits have a prominent fine-ashed size fraction (de’Gennaro et al 1999, https://[www.sciencedirect.com/science/article/abs/pii/S0377027399000402)](http://www.sciencedirect.com/science/article/abs/pii/S0377027399000402)) they also contain a substantial coarse ash to lapilli sized fraction. Therefore, referring to these deposits as “powder” is misleading. Please use the correct volcanological terms, defining them where appropriate.

*The revision of the manuscript carefully considered all aspects related to the Introduction and the specific Latin terminology. The introduction underwent a comprehensive restructuring, with particular attention to detail, resulting in an expanded paragraph. In the initial version of the manuscript, these elements received only brief treatment, despite their thorough exploration in a prior study that focused more extensively on the literary components (Dilaria, S., Secco, M., Ghiotto, A.R. et al. Early exploitation of Neapolitan pozzolan (pulvis puteolana) in the Roman theatre of Aquileia, Northern Italy. Sci Rep 13, 4110 (2023).* [*https://doi.org/10.1038/s41598-023-30692-y*](https://doi.org/10.1038/s41598-023-30692-y) *). Nonetheless, we found it necessary to delve deeper into this discussion, incorporating all reviewer feedback and introducing additional literary references to ensure the coherence and integrity of the text.*

*Moreover, terms like "prodigious" and "exalted" have been avoided in the revised draft of the introduction. Additionally, as pointed out in various circumstances, the grain size of the volcanic deposits, both in the samples under examination and generally, has been modified from "ash"/”powder” to "medium lapilli-sized pumiceous tephra." References to "pulvis" have been retained only in connection with Vitruvian mentions. We believe that this term could indeed align with the construction knowledge of a Roman builder who used the term "powder" to describe a loosely cohesive material that could easily be reduced to dust, either naturally or through rapid mechanical grinding. Therefore, we preferred to maintain this ambivalence between Vitruvian "pulvis" and the documented "medium lapilli" size in the samples.*

**Kinetics**

- this discussion is based on only four EDS point analyses in low magnification images (Figure 6 b2, c2, c3, d1) and no direct information regarding ﬂuid compositions in the lagoon. To accurately describe “kinetics of pozzolanic and para-pozzolanic reaction processes” more data is needed, such as high resolution SEM-EDS maps of relevant microstructures, high magnification images of the phase morphologies, and a potential resolution of the AFm phases in X-ray diﬀraction analyses, as hydrotalcite, for example.

AND

- Section 4.2 has a lengthy discussion of magnesium-enriched phases in the binding matrix. This is of interest, yet there seem to be only four EDS point analyses in low magnification images related to this process (Figure 6). The XRD analyses of Figure 4 state AFm phases, but these are not identified separately, e.g. as hydrotalcite. More data is needed – for example, high resolution SEM-EDS maps of instructive microstructural environments distinguishing pozzolanic reaction processes from subsequent authigenic processes, occurring possibly after the full consumption of calcium-hydroxide. In its current form, this hypothesis remains speculative.

AND

- “The backscattered electron images, as presented in Figure 6, reveal that most of the pozzolanic aggregates are profoundly reacted with the binder.”

This sentence is very confusing. Which are the pozzolanic aggregates? ... only the pumice? What data justifies "pozzolanic" reaction? Did the pozzolanic products then react with the binding matrix? Please separate out the concepts and describe them one by one after first presenting the analytical data.

Each clast type should be identified in the caption for each panel (and in the graphic as well, to assist the reader). Please also add a label to each EDS analysis panel, stating the phase from which the compositional analysis is derived.

*In the revision of the manuscript, more detailed information on the crystal structures of AFm phases determined by XRPD analyses has been reported. Furthermore, the microstructural and microchemical characteristics of the pozzolanic reaction products have been better described through more detailed sentences and a new supporting figure (Figure 7). A dedicated sentence on the type of pozzolanic aggregates interested by pozzolanic interaction phenomena has been added.*

**Cistern diagram**

- Section 5.1 is difficult to follow since there are no diagrams outlining the well-cistern structure (possibly shown in Fig. S1?)and relationships with changing water level from ancient to modern time.

*A new subfigure (fig 1, c) reporting the original reconstruction of the well-cistern reporting the height of local water level in Roman times compared with the situation at present was added in the edited draft.*

**Other aspects**

- Results presented in the Introduction should be moved to the Conclusions section

*These sections were rearranged and the draft reorganized according to the suggestions provided by the Reviewer, also considering the detailed comments to the draft in the PDF file.*

- statements questioning the validity of the LA-ICP-MS analyses of “unreacted glass” should be fully resolved.

*As already stated, “This technique has been already adopted as a rapid and efficient tool for a preliminary assessment of volcanic pozzolans’ geochemistry included in ancient mortar-based materials” as reported for example in Marra et al. 2013; Marra et al. 2016; D’Ambrosio et al. 2015.*

- Abstract. This statement, “significant reaction processes the analysed pozzolans were object of, seriously aﬀecting their geogenic chemical and petro-mineralogical features”, calls into question preceding sentence, a "conclusion", "pyroclastic aggregates from the Phlegraean fields (pulvis puteolana) were added to the mortars". Please revise and clarify.

First, present the analytical data, Second, temper the conclusion: for example, "even so, the lithological origin of the tephra seems to the Campi Flegrei volcanic district."

*The abstract was rearranged according to the suggestions and reordered presenting in order the analytical data and tempering the conclusions*

- “perfect” and “perfectly” appear 6 times in the manuscript, with no justification for what “perfect” actually means: “having all the required or desirable elements, qualities, or characteristics; as good as it is possible to be; without fault, faultless, ﬂawless.”

“Perfect” is a qualitative assessment – not a scientific descriptor.

The mortars are not ﬂawless materials, please use a diﬀerent descriptor. Neither is the preservation “perfect”, since there has apparently been much “decay” of the components of the original mortar fabric. Jackson et al. use the term “beneficial corrosion.”

*As rightly pointed out by the reviewer, these qualitative adjectives (e.g., "perfect," "peculiar," etc.) are misleading in the text and not suitable for discussing analytical data. They have been entirely removed in the revised draft.*

- This is not correct. "Aerial lime-based mortars" develop their binding characteristics through carbonation. That process is antithetical to mortars with pozzolans that develop mainly silicate binding components.

Do you mean lime-based mortars in sub-aerial construction? or architectural applications? Revision is needed. See:

https://www.researchgate.net/publication/358625402_AERIAL_LIME_MORTARS_AN_INTRODUCTION_AND_A_BRI EF_DISCUSSION

*As rightly pointed out by the reviewer, the original phrase was misleading, as “aerial” specification assumes that a mortar had completed the carbonation process, whereas we do not intend to discuss the physical and mechanical improvements of mortars that have undergone complete carbonation. Therefore, the misleading term "Aerial" has been removed from the sentence, which has been modified to "lime-based mortars," without delving into the details of lime setting processes.*

- The term ”volcanic pozzolans” adds more confusion. It attributes reactive capabilities to these particles that have not been validated through analyses. These appear to be pumice fragments, possibly with accretionary ash rims. (The astute reader would also wonder why the ceramic particles have no pozzolanic attributes.)

*Edited in the revised draft according to the useful suggestion. In the revised manuscript volcanic pozzolans was substitute in most of the cases with the actual material under study (i.e. Pumice, pumiceous tephra, weakly lithified tuffs) in particular when describing the results of the analytical investigations. We maintained the reference to “pozzolanic materials” or “volcanic pozzolans” in general only in the discussion and conclusions sections as they refer to general consideration of these kinds of materials.*

- The hypothesis stating the relevance of the materials to the descriptions in *de Architectura* is not directly tested and validated.

*The revised draft now includes a more thorough comparison between the materials under study and the Vitruvian source, in particular in the discussion and conclusions. Further investigation into the disparities between Vitruvius's description of Neapolitan "pulvis" usage and its actual implementation in construction has been conducted in the discussion and conclusions. This exploration showcases the strong connection with traditional Roman construction practices and explores alternative applications of "pulvis" in coating contexts. However, the primary focus remains on highlighting the reactive properties of volcanic pozzolanic material, particularly its alkaline reaction with water, whether utilized in mixes or upon contact post-placement. This emphasis aligns seamlessly with the empirical understanding presented in De architectura regarding mortar hardening and durability in submerged environments.*

- The photomicrographs and micrographs in the manuscript show many clasts greater than ash-sized (or sand- sized, < 2mm). Much of the tephra is lapilli-sized, 2- 64 mm. This is not a powder - but, rather, a medium lapilli- sized pumiceous tephra.

*Edited throughout the revised manuscript according to this right observation.*

- This is highly problematic. As previously noted, Vitruvius never used the term "pulvis puteolana". Furthermore, there is no "prodigious strength" – compressive strength measurements are uniformly low in the ancient concretes and their modern replicas, < 9 MPa (Oleson et al. 2006, DOI: 10.1017/S1047759400006255).

*The sentence addressing this aspect in the discussion section has been refined to highlight the moderate durability of underwater concretes incorporating Neapolitan pozzolans when compared to traditional ancient mortars, albeit still falling short of the mechanical strength exhibited by modern concretes.*

Sincerely,

Simone Dilaria, Ph.D. (corresponding author)

Assistant Professor (RTD-A) - L/ANT 07 (Classical Archaeology)

University of Padova, Department of Cultural Heritage

Piazza Capitaniato, 7 - 35139 Padova (Italy)

[simone.dilaria@unipd.it](mailto:simone.dilaria@unipd.it)
